# Brønsted acid-enhanced copper-catalyzed atroposelective cycloisomerization to axially chiral arylquinolizones via dearomatization of pyridine

**DOI:** 10.1038/s41467-022-27989-3

**Published:** 2022-01-18

**Authors:** Xiao-Long Min, Xiu-Lian Zhang, Wenbin Yi, Ying He

**Affiliations:** School of Chemistry and Chemical Engineering, Nanjing University of Science & Technology, Nanjing, 210094 China

**Keywords:** Synthetic chemistry methodology, Asymmetric catalysis

## Abstract

The construction of axially chiral *N*-heterobiaryls is of great interest as a result of their occurrence in organocatalysts, chiral ligands, natural products, and biologically active molecules. Despite remarkable achievements in this area, strategies for the preparation of new classes of axially chiral *N*-heterobiaryls remain to be further explored. Herein, we report the enantioselective synthesis of axially chiral arylquinolizones through an intramolecular atroposelective cycloisomerization. The reaction proceeds via the Brønsted acid-enhanced dearomatization of pyridine by a copper catalyst that allows for the formation of the desired products in excellent yields and enantioselectivities. The utility of this methodology is illustrated by a synthesis on gram scale production and transformation of the products into chiral thiourea catalysts. Mechanistic studies demonstrate that Brønsted acid plays a significant role in promoting the reactivity of the reaction, while both the steric and electronic effects of aryl substituents in substrate play a role in controlling the stereoselectivity.

## Abstract (web summary)

Methods to assemble axially chiral biaryl molecules with nitrogen-containing heterocycles can have implications in ligand development for homogeneous catalysis. Here the authors report enantioselective syntheses of axially chiral arylquinolizones, via an intramolecular atroposelective cycloisomerization.

## Introduction

**Fig. 1:**
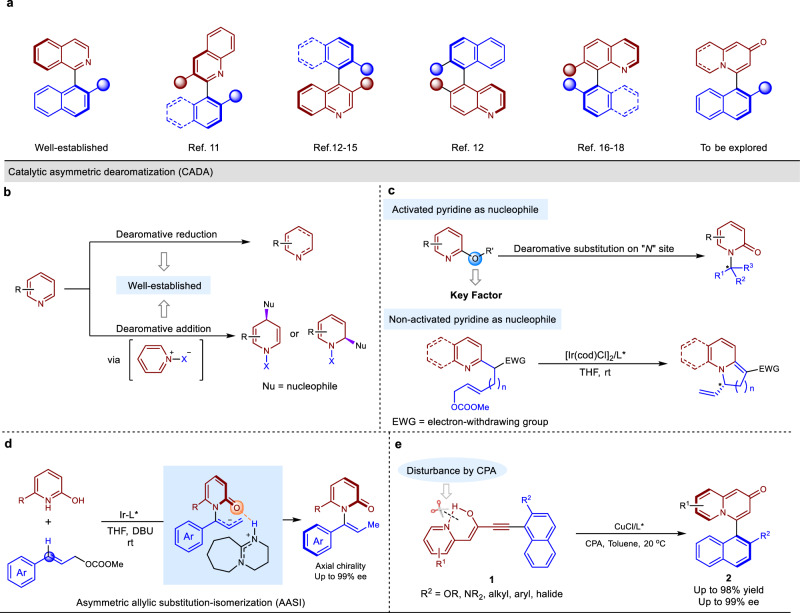
Background and concept design. **a** Representative examples of axially chiral axially chiral *N*-heterobiaryls. **b** State of the art of CADA using pyridine as an electrophile. **c** State of the art of CADA using pyridine as a nucleophile. **d** AASI for the synthesis of axially chiral enamides. **e** Atroposelective cycloisomerization to axially chiral arylquinolizones.

In recent years, substantial effort has been devoted to the efficient and modular catalytic synthesis of axially chiral *N*-heterobiaryls because of their broad applicability to pharmaceuticals, functional materials and catalysis (Fig. 1a)^1–5^. 1-(Isoquinolin-1-yl)naphthalene-2-ol (QUINOL), a representative example of an axially chiral *N*-heterobiaryl, has been studied extensively as a precursor to accessing a variety of ligands or catalysts, including QUINAP, IAN, and QUINOX^6–10^. Thus, there has been increased interest in the development of methods for the facile construction of axially chiral arylquinolines and arylisoquinolines^11–18^. Various strategies for the synthesis of these compounds, including dynamic kinetic resolution, enantioselective functionalization of prochiral *N*-heterobiaryls and enantioselective cross-coupling, have been developed^1–5^. However, to the best of our knowledge, there was no catalytic approach for accessing axially chiral arylquinolizidine skeletons so far, despite the fact that quinolizidines are widespread components of pharmaceuticals^19–21^.

On the basis of the previous reports on the preparation of quinolizidine analogs, we considered the catalytic asymmetric dearomatization (CADA) reaction of pyridines as a challenging but potentially efficient strategy to address this synthetic problem^22–28^. Enantioselective hydrogenation represents a direct and well-established approach for the CADA of pyridines (Fig. 1b)^29–31^. More recently, the enantioselective nucleophilic addition to pyridinium salts that are preactivated at the nitrogen site has been reported as well (Fig. 1b)^32–41^. However, in spite of this catalytic system, a significant drawback of this strategy is the need to remove the protecting group from the nitrogen at a later stage. Nevertheless, pyridine analogs in these cases are used as electrophiles. A brief survey of the literature reveals that the direct CADA of pyridine derivatives that behave as nucleophiles is much less explored. In this context, activated 2-hydroxy- and 2-alkoxypyridines have been employed (Fig. 1c)^42–46^. A unique example of the enantioselective dearomatization of unactivated nucleophilic pyridines was reported by the You group in 2014 (Fig. 1c)^47,48^. In their study, the iridium-catalyzed enantioselective allylic substitution was applied to generate the centrally chiral products using pyridine nitrogen as the nucleophilic site. The only report on the efficient CADA of nucleophilic pyridine derivatives to give axially chiral compounds was disclosed by our group, but the substrates were still limited to 2-hydroxypyridine analogs (Fig. 1d)^49^.

As part of our continuing efforts in axially chiral compounds^26,49–51^, we envisioned that CADA of nucleophilic pyridines might also be used for the atroposelective cycloisomerization to axially chiral arylquinolizones. If successful, it would represent the first example of their facile construction from readily prepared alkyne starting materials. At the outset of our studies, we anticipated that several challenges that may arise during our efforts to develop this cycloisomerization process: (1) The sp^2^ nitrogen lone pair of the pyridine directed outward the ring skeleton which is well directed to have overlap with vacant metal orbital in producing an σ bonding interaction. This may cause pyridine to be a good ligand, resulting in poisoning and deactivation of the metal catalyst. (2) The lack of assistance by -OR or -NHR group and bulky *ortho*-substituents in arene **1** may lead to the low reactivity of π-activation by TM-catalysis. (3) The ratio of *enol* and *keto* isomers of starting materials may cause difficulty in balancing the enantioselectivity and reactivity.

In this work, we report a Brønsted acid-enhanced copper-catalyzed atroposelective cycloisomerization to access axially chiral arylquinolizones via dearomatization of pyridines **1** (Fig. 1e).

## Results

### Reaction optimizations

To commence our study, we evaluated the reaction conditions by using **1a** as the model substrate for the synthesis of axially chiral arylquinolizones. At first, the use of AgOTf^26^ with L1 gave product **2a** in low 29% ee, albeit with a good yield of 70% (Table 1, entry 1). The low ee of **2a** can be attributed to the strong background reaction since the reaction could proceed rapidly in the presence of silver catalyst without the ligand^26^. Next, we examined the copper catalyst for the reaction. While the use of CuBF_4_ afforded the product in 50% yield with 36% ee (entry 2), changing the catalyst from CuBF_4_ to CuCl resulted in a substantial improvement in the ee of **2a** (73%) (entry 3). For both copper sources, the reaction rate was slower compared to AgOTf, and the background reaction was less significant. The solvent was also evaluated, and toluene was found to give a small improvement of enantioselectivity to 76% ee, though the yield was still moderate (entries 4–6). The ee of **2a** was decreased to 20–69% when L2–L5 were used as ligands for the catalyst (entries 7–10).

**Table 1:** Optimized conditions for the reaction^a^.


Entry	Catalyst	Ligand	Solvent	Additives	*t* (h)	Yield (%)^b^	Ee (%)^c^
1	AgOTf	L1	DCE	–	0.5	70	29
2	CuBF_4_	L1	DCE	–	72	50	36
3	CuCl	L1	DCE	–	72	51	73
4	CuCl	L1	THF	–	72	38	69
5	CuCl	L1	MeCN	–	72	34	56
6	CuCl	L1	Toluene	–	72	45	76
7	CuCl	L2	Toluene	–	72	30	20
8	CuCl	L3	Toluene	–	72	35	46
9	CuCl	L4	Toluene	–	72	56	69
10	CuCl	L5	Toluene	–	72	30	35
11^d^	CuCl	L1	Toluene	–	72	51	90
12^d^	CuCl	L1	Toluene (2.0 mL)	–	72	70	90
13^d^	CuCl	L1	Toluene (3.0 mL)	–	72	58	90
14^d^	CuCl	L1	Toluene	(*R*)-CPA	36	88	92
15^d^	CuCl	L1	Toluene	TsOH	36	67	90
16^d^	CuCl	L1	Toluene	PhCOOH	36	57	90
17^d^	CuCl	L1	Toluene	(*R*)-CPA (0.5 eq)	36	83	91
18^d^	CuCl	L1	Toluene	(*R*)-CPA (0.2 eq)	36	76	90
19^d^	CuCl	L1	Toluene	NaBAr_F_	72	26	13
20^d^	CuCl	L1	Toluene	DBU	72	N.D.^e^	N.D.^e^


^a^Reaction conditions: **1a** (0.1 mmol), catalyst (10 mol%), ligand (15 mol%), solvent (1.0 mL), 20 °C.

^b^Yields of isolated products.

^c^Ee values were determined by chiral HPLC.

^d^**1b** was used as the substrate.

^e^N.D. = Not detected. Compound **1b** was decomposed.

To further optimize the reaction conditions, we reasoned that a bulkier group on the naphthalene ring may be useful for improving the level of enantiocontrol. As expected, the use of **1b** bearing an -OEt instead of -OMe at the 2-position of the naphthalene ring significantly improves the enantioselectivity of **2b** (90% ee), although the yield was still moderate (51%) (Table 1, entry 11). Conducting the reaction at lower concentrations afforded improved levels of reactivity (entries 12 and 13). Given the existence of enol and pyridine groups in substrate **1**, we hypothesized chiral phosphoric acid (CPA) may be beneficial for not only the reactivity but enantioselectivity because of their hydrogen-bonding interactions^52–56^. With inexpensive BINOL phosphoric acid as the CPA, the reaction reached completion within 36 h, providing **2b** in higher yield (88%) and ee (92%) (entry 14). Examination of other acid additives showed that (*R*)-CPA was superior in this system (entries 14–18). Lower yield and a slight decrease in ee were obtained by decreasing the amount of (*R*)-CPA (entries 19 and 20). On the other hand, the use of additives NaBArF and DBU resulted in detrimental effects on the reaction (entries 19 and 20). It should be noted that π-activation by a low-cost copper catalyst for the enantioselective construction of atropisomers is rare and the example was only reported very recently^57^.

### Substrate scope

**Fig. 2:**
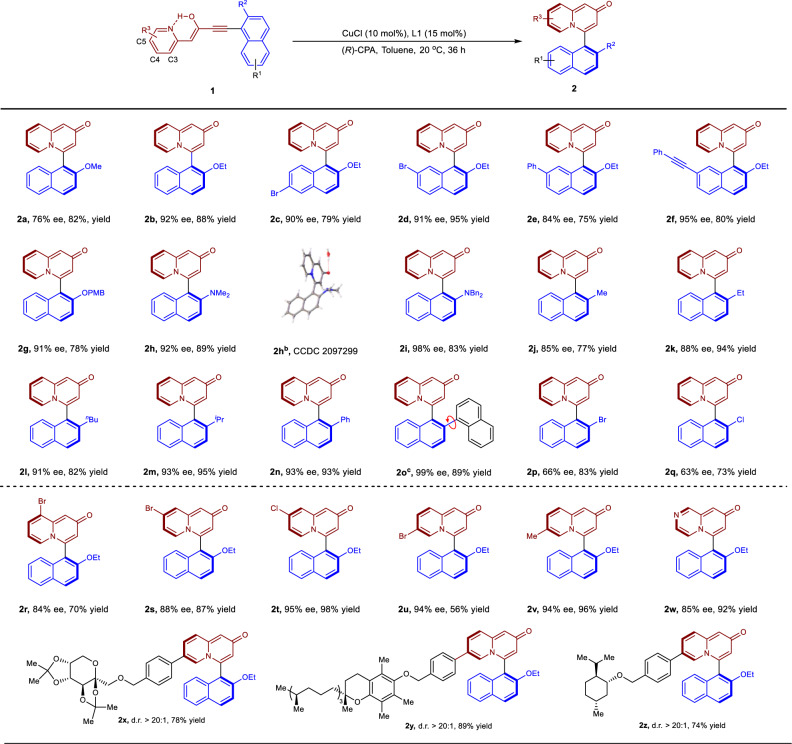
Substrate scope of the reaction. Reaction conditions: **1** (0.1 mmol), CuCl (10 mol%), L1 (15 mol%), (*R*)-CPA (0.1 mmol), toluene (2.0 mL), 20 °C for 36 h. The yield of isolated products. *Ee* values were determined by chiral HPLC. OPMB = *p*-methoxybenzyl^b^. The single-crystal X-ray of **2h** contains one molecule of H_2_O^c^. There are conformers that existed around the red arrow.

Having identified optimized conditions for the reaction, we next explored the substrate scope. In general, the substituent R^1^ on naphthol core such as bromo-, phenyl-, and alkyne- group has little effect on the yields and enantioselectivities, and the products could be isolated in 75–95% yield with 84–95% ee (Fig. 2, compounds **2c**–**2f**). Variation of *R*^2^ group in **1** proved to have dramatic effects on the enantioselectivity of the reaction (**2a**, **2g**–**2q**). Replacement of -OEt with -OPMB provided the product in 91% ee and 78% yield (compound **2g**). Notably, protected amines were also compatible at this position, leading to the desired **2h** and **2i** in excellent yields with high enantioselectivities of 92 and 98% ee, respectively. The absolute configuration of **2h** was determined by single-crystal X-ray analysis, and others were assigned by analogy to **2h**. It should be noted that, unlike previous enantioselective addition of alkynes to axially chiral heterobiayls^11,57–63^, substituent *R*^2^ is not limited to -OH, ether or amine. In fact, it was found to be amenable to both alkyl and aryl groups, providing the products in 85–99% ee (compounds **2j–2o**). However, alteration of -OEt to a halogen group such as -Br and -Cl resulted in moderate ee of 66 and 63%, respectively (compounds **2p** and **2q**). We speculated that the low ee of **2p** and **2q** may be attributed to their smaller size compared to other substituents.

To further demonstrate the generality of this protocol, the cyclization of a variety of substituted pyridines was explored. Substrates **1** bearing substituents at C3, C4, or C5 positions of pyridine were well tolerated, affording products in 84–94% ee with 56–96% yield (Fig. 2, compounds **2r–2v**). Modification of the central core of pyridine to pyrazine afforded the product **2w** in 85% ee and 92% yield. Since quinolizone derivatives are indispensable frameworks of numerous natural products and pharmaceuticals, the decoration of the substrate with complex bioactive molecules was also investigated. For instance, diacetonefructose derivative **1x** could undergo atroposelective cyclization to generate product **2x** in 78% yield with >20: 1 dr. The reaction also tolerated tocopherol and (*L*)-menthol derivatives, which delivered **2y** and **2z** in excellent yield (89 and 74% yield) and dr value (>20:1).

### Transformations and applications of the products

**Fig. 3:**
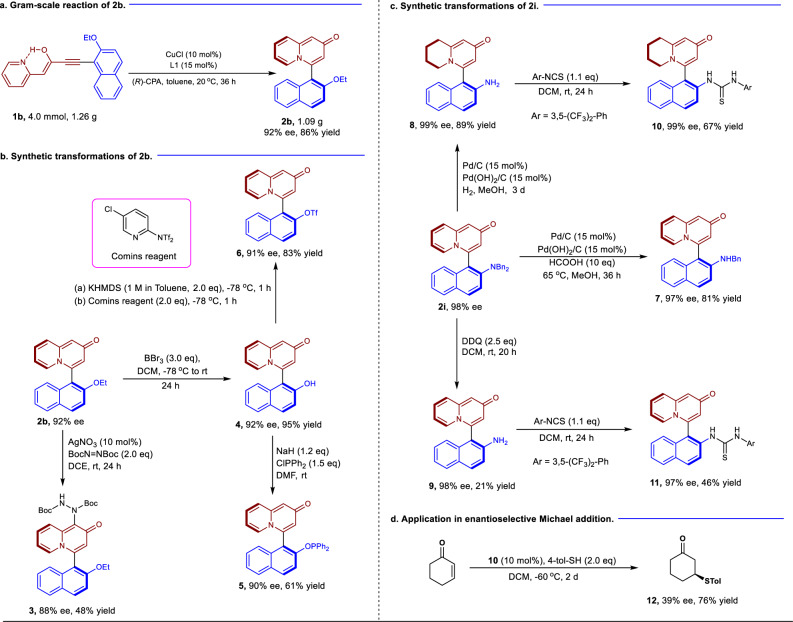
Large-scale experiments and synthetic applications. **a** Gram-scale reaction of **2b**. **b** Synthetic transformations of **2b**. **c** Synthetic transformations of **2i**. **d** Enantioselective Michael addition using compound **10** as the catalyst.

Having established these robust and general reaction conditions, we next explored the scale-up of the procedure to gram-scale. The synthesis of **2b** was conducted on a 4.0 mmol scale, and the product was obtained in 92% ee with 86% yield (1.09 g) (Fig. 3a). Subsequently, a series of synthetic transformations were performed based on products **2b** and **2i**. As can be seen from Fig. 3b, direct amination by silver catalysis led to product **3** in 88% ee with 48% yield. Next, deprotection of **2b** by BBr_3_ delivered compound **4** in 92% ee and 95% yield. Using **4** as a substrate, the axially chiral phosphinite **5** could be readily generated in 90% ee. Importantly, the coupling precursor **6** could be easily obtained in 83% yield while maintaining the enantioselectivity in the presence of Comins reagent.

The selective reductions of **2i** in the different reductive systems were then carried out (Fig. 3c). Interestingly, the use of HCOOH as a reductant under palladium catalysis afforded compound **7** in 97% ee with 81% yield, whereas using H_2_ as a reductant, compound **8** was generated using the same catalyst. The direct deprotection of the dibenzyl group of **2i** was finally achieved through oxidative conditions without any loss in enantioselectivity, albeit with a yield of 21%. Nevertheless, the axially chiral thiourea catalysts **10** and **11** which derived from **8** and **9** respectively were generated in both high enantioselectivities. To probe the reactivity of the catalyst, the enantioselective Michael addition of thiophenol with cyclohexenone was chosen as the model reaction. The 1,4-addition product **12** was obtained in a good yield of 76% with 39% ee. These results demonstrate the potential utility of this approach for the synthesis of new organocatalysts for asymmetric catalysis. (Fig. 3d).

### Mechanistic studies

**Fig. 4:**
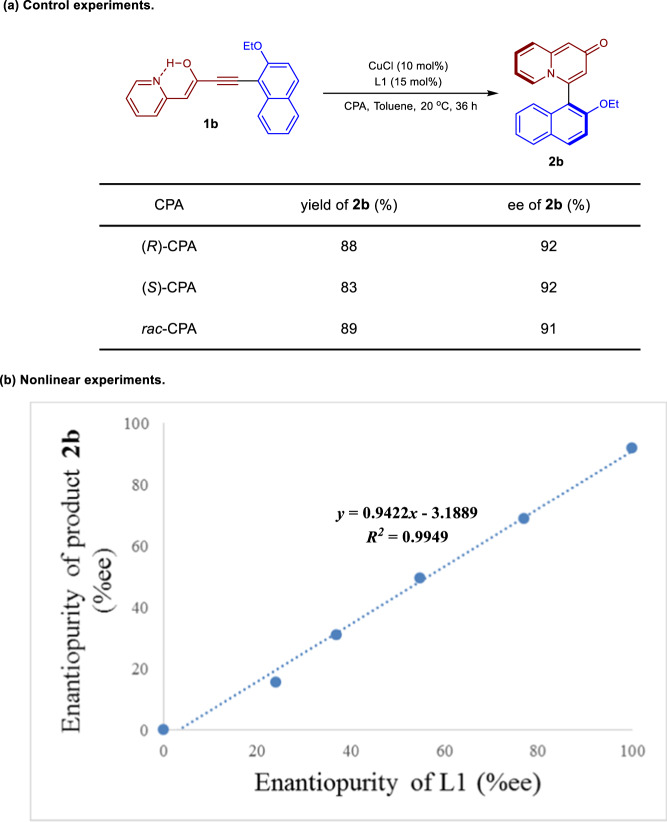
Mechanistic studies of enantioselectivities of reaction. **a** Control experiments. **b** Nonlinear experiments.

To probe the reaction mechanism, a series of control experiments were conducted. First, the effect of the additive was investigated by comparing the two enantiomers and racemate of CPA. As can be seen from Fig. 4a, there is no obvious change in either yield or enantioselectivity when the (*R*) isomer of the CPA was replaced with the enantiomer of the racemic mixture. Similar results were also obtained by using other representative substrates for the reaction (see Supplementary Table 3 for details). In addition, the reaction displays a clear linear relationship between the enantiopurity of phosphoramidite L1 and that of product **2b** (Fig. 4b). These observations indicate that only one molecule of L1 is likely to be ligated to the copper catalyst during the cycloisomerization process, whereas CPA is either not involved in the enantiodetermining transition state or far from the site of reactivity and not responsible for the enantiocontrol of the reaction.

**Fig. 5:**
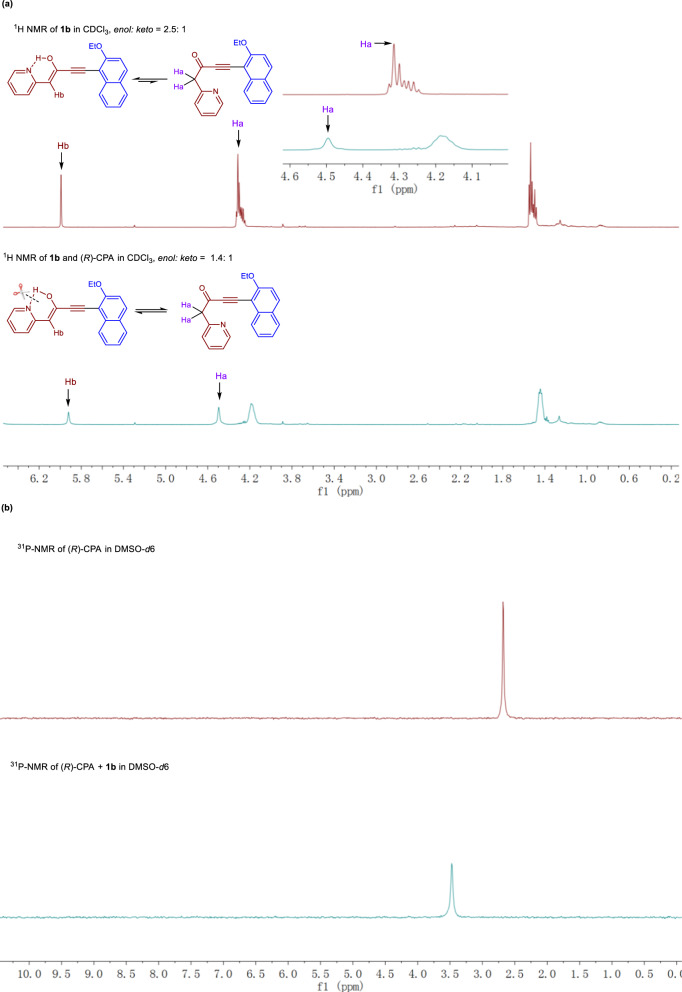
Mechanistic studies of the reactivity of the cycloisomerization. **a** ^1^H NMR studies of a mixture of **1b** and (*R*)-CPA. **b** ^31^P NMR studies of mixture of **1b** and (*R*)-CPA.

Next, we investigated how the CPA affects the reactivity of cycloisomerization. Considering the co-existence of the *enol* and *keto* equivalent of **1b**, we investigated the interactions of (*R*)-CPA and **1b** by ^1^H-NMR and ^31^P-NMR. As can be seen from Fig. 5a, the original ratio of *enol/keto* was calculated as 2.5:1 by ^1^H-NMR spectroscopy, and the *H*_a_ and *H*_b_ signals were recorded as *δ* 4.31 and *δ* 5.99. In the case of the addition of (*R*)-CPA, the ratio of *enol*/*keto* was decreased to 1.4/1. Moreover, the mixture of (*R*)-CPA (1.0 equiv) and **1b** (1.0 equiv) exhibited, two signals (*δ* 4.50 and *δ* 5.92) for *H*_a_ and *H*_b_, respectively, at a significantly different chemical shift compared to **1b** in the absence of the CPA additive. The interactions were further confirmed by ^31^P-NMR studies in DMSO-*d*6 (Fig. 5b). It is worth mentioning that mixture of CPA and **1b** in CDCl_3_ was homogeneous, while the CPA itself forms a suspension at the same concentration. These observations all point to the formation of a hydrogen-bond complex between the CPA and the starting material **1b**, which disrupts the intramolecular hydrogen-bonding in the *enol* form of **1b**, leading to greater amounts of the *keto* form.

**Fig. 6:**
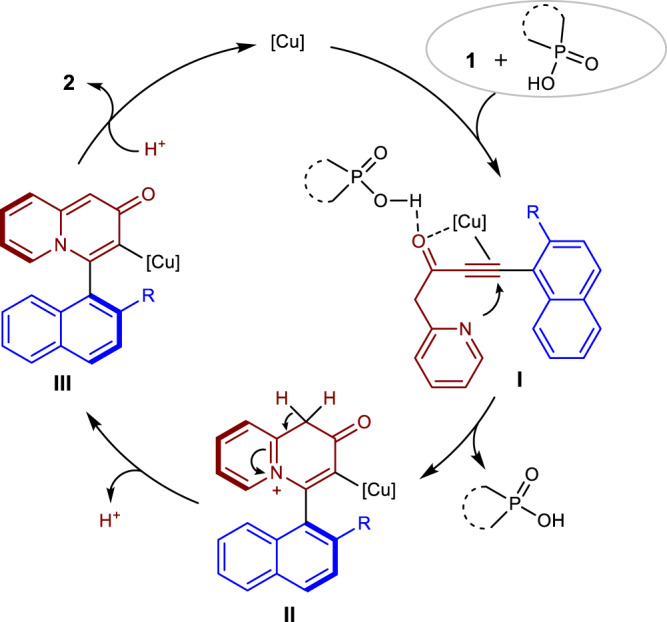
Proposed catalytic cycle of the reaction. Copper catalyst plays a role in controlling the stereoselectivity, while CPA plays a significant role in promoting the reactivity of the reaction.

On the basis of the results above, a proposed mechanism is outlined in Fig. 6. First, substrate **1** coordinating with CPA increases the ratio of *keto*/*enol* because of the hydrogen-bonding interactions. Meanwhile, the π-activation by copper catalyst with the direction of carbonyl occurred (see Supplementary Fig. 6 for details). The CPA hydrogen-bonded to the carbonyl in **I** making the alkyne more electrophilic which facilitated the intramolecular nucleophilic addition to the alkyne to form chiral intermediate **II**. The following isomerization and protonation of **III** would then occur to yield product **2** and regenerate the copper catalyst.

**Fig. 7:**
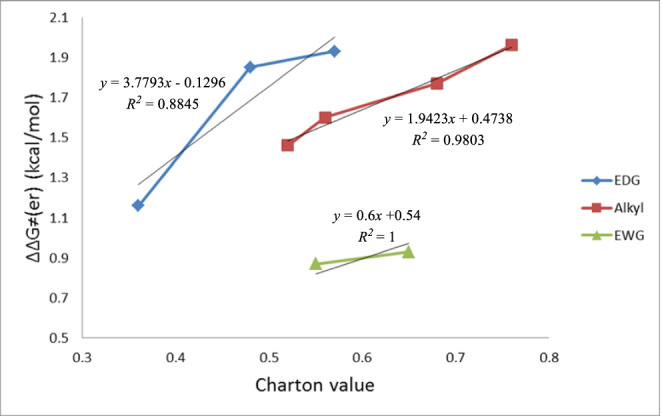
Correlation of the representative substrate parameters to enantioselectivities. EDG electron-donating group, EWG electron-withdrawing group.

Finally, linear free energy relationships (LFERs) were studied to probe the trends in enantioinduction by substrates^64–70^. Firstly, Charton values^71–73^, which are related to the van der Waals radius of the functional group, was used to relate the enantioselectivities. Unfortunately, no correlation was found when selected products were taken into consideration (Table 2). In addition, a non-linear correlation was also observed when Sterimol parameter^65,70^ was exploited (see Supplementary Tables 4 and 5 for details). However, when the electronic effect was divided into an electron-donating group (Table 2, entries 1–3), alkyl group (entries 4–7), and electron-withdrawing group (entries 8 and 9), good correlations were obtained (Fig. 7)^74–77^. Therefore, it seems that (a) the steric effect may play a role in enantioselectivity albeit that Charton values can not describe this influence accurately; (b) other factors such as electronic effect may also participate in the control of stereoselectivity.

**Table 2:** Examination of steric effect on enantioselectivity^a^.


Entry	*R*	er	Charton value	△△*G*^≠^ (kcal K^−1^ mol^−1^)^b^
1	OMe	88.06:11.94	0.36	1.16
2	OEt	96.0:4.0	0.48	1.85
3	Ph	96.52:3.48	0.57	1.93
4	Me	92.42:7.58	0.52	1.46
5	Et	94.02:5.98	0.56	1.60
6	^*n*^Bu	95.45:4.55	0.68	1.77
7	^*i*^Pr	96.66:3.34	0.76	1.96
8	Cl	18.32:81.68	0.55	0.87
9	Br	16.95:83.05	0.65	0.93

^a^Reaction conditions: **1** (0.1 mmol), CuCl (10 mol%), L1 (15 mol%), (*R*)-CPA (0.1 mmol), toluene (2.0 mL), 20 °C for 36 h. Yields of isolated products, er values were determined by chiral HPLC.

^b^ΔΔ*G*^≠^ = RTln(*er*), *R* = 0.001986 kcal K^−1^ mol^−1^, *T* = 293.15 K.

**Fig. 8:**
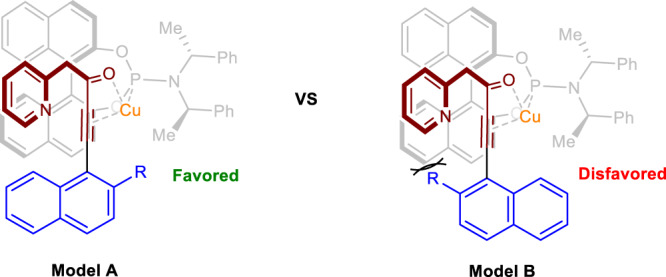
Proposed stereoinduction modes. Cl^−^ is omitted for simplicity.

Based on the mechanistic studies and absolute configuration of product **2**, we proposed two possible stereoinduction models. As shown in Fig. 8, the transition-state model **A** accounts for the observed stereochemistry of axial chirality. It is obvious that model **B** is disfavored relative to **A** since the steric repulsion between the R group and the backbone of the ligand. Thus, the bulky substituents of the R group are crucial to suppress model **B** which contributes to the obvious improvement in the enantioselectivity. This result is also consistent with our substrate scope investigations illustrated in Fig. 2.

## Discussion

In conclusion, we have developed a strategy for the synthesis of axially chiral arylquinolizones. This transformation was achieved by a copper-catalyzed enantioselective dearomatization of nucleophilic pyridines. Control experiments indicated that this transformation was enhanced by Brønsted acid by changing the enol/keto ratio of the substrate. The synthetic utility of the strategy was demonstrated by obtaining a series of axially chiral arylquinolizone analogues. Moreover, the axially chiral quinolizone-type thiourea could be used as a new catalyst for enantioselective Michael addition, albeit with moderate ee. LFERs analysis indicated that both a steric hindrance and electronic effect participate in the control of stereoselectivity. Further studies on the reaction mechanism are currently in progress in our laboratory.

## Methods

### General procedure for the synthesis of enantioenriched compounds 2

To a vial was added CuCl (1.0 mg, 10 mol%), L1 (8.5 mg, 15 mol%), toluene (2.0 mL), and stirring bar. The vial was wrapped with Teflon tape and fitted with a corresponding cap. The vial was stirred at room temperature for 30 min. Then compound **1** (0.1 mmol, 1.0 eq) and phosphoric acid (34.8 mg, 0.1 mmol, 1.0 eq) were added into the reaction mixture. The reaction was stirred at 20 °C for 36 h. The reaction was then diluted with EA and washed with water three times. The extracts were dried and the product was purified by column chromatography over silica gel. Full experimental details and characterization of new compounds can be found in the Supplementary Information.

## Supplementary information

Supplementary Information

## Data Availability

The authors declare that the data supporting the findings of this study are available within the article and Supplementary Information file, or from the corresponding author upon request. The X-ray crystallographic coordinates for structures reported in this study have been deposited at the Cambridge Crystallographic Data Centre (CCDC), under deposition number CCDC 2097299 (**2h**). The data can be obtained free of charge from The Cambridge Crystallographic Data Centre via www.ccdc.cam.ac.uk/data_request/cif.
