# Supplementary material for: Brønsted acid-enhanced copper-catalyzed atroposelective cycloisomerization to axially chiral arylquinolizones via dearomatization of pyridine
Source: Nat Commun. 2022 Jan 18;13:373. doi: 10.1038/s41467-022-27989-3 (PMC8766466; doi:10.1038/s41467-022-27989-3)
Supplement: Supplementary file 1 — Supplementary Information [file 41467_2022_27989_MOESM1_ESM.pdf]

Supplementary Information

Brønsted Acid-Enhanced Copper-Catalyzed Atroposelective Cycloisomerization to Axially Chiral Arylquinolizones via Dearomatization of Pyridine

Xiao-Long Min, Xiu-Lian Zhang, Wenbin Yi* & Ying He*

School of Chemistry and Chemical Engineering, Nanjing University of Science & Technology, Nanjing 210094, China

Table of Contents

Supplementary Methods S3
General Information S3
General procedure A: Synthesis of substrate 1 from 2-substituted-1-ethynylnaphthalene[1] S3
General procedure B: Synthesis of 2-alkoxy-1-ethynylnaphthalene from 2-naphthol S4
General procedure C: Synthesis of 1-ethynyl-*N,N*-dialkylnaphthalen-2-amine from 2-naphthylamine S5
General procedure D: Synthesis of 2-halide-1-ethynylnaphthalene from 1-Naphthylamine S7
General procedure E: Synthesis of 1-ethynyl-2-alkylnaphthalene from 2-alkylnaphthalene S8
General procedure F: Initial studies for the synthesis of **2a** S23
Optimization of reaction conditions S23
General procedure G: Comparison of $(PhO)_2POOH$ and CPA for the reaction S25
General procedure H: Synthesis of the enantioenriched products **2** S26
Racemization experiments S39
Procedure for gram-scale reaction of **2b**. S44
Synthetic transformations. S44
Application of **10** for enantioselective Michael addition. S52
^{1}H NMR and ^{31}P NMR spectrum of **1b** and CPA S53
Dynamic ^{1}H NMR spectrum of **1k** and CPA S55
Linear effect of ligand and product S56
The linear free energy relationship analysis [a] S57
Spectral data S60
HPLC data S125
Supplementary References S158

Supplementary Methods

General Information

Unless otherwise noted, all starting materials were purchased from commercial sources and used without any further purification. Anhydrous THF is obtained by distillation over sodium and benzophenone ketyl immediately before use. The analytical data for the known compounds were found to match with the literature data and stored at -30 °C under an inert atmosphere. Thin layer chromatograph plates were visualized under UV light (254 nm) or by staining with phosphomolybdic acid or $KMnO_4$ followed by heating. Abbreviations are reported as follows: DCM = dichloromethane, DCE = 1,2-dichloroethane, THF = tetrahydrofuran, DMF = *N*, *N*-dimethylformamide, EA = ethyl acetate, TLC = thin layer chromatograph, PMB = *p*-methoxybenzyl. Nuclear magnetic resonance (NMR) spectra were recorded using an AVANCE 500 Bruker spectrometer and chemical shifts were reported in ppm. Multiplicities are reported using the following abbreviations: s = singlet, d = doublet, t = triplet, q = quartet, m = multiplet. Melting points were measured on INESA SGW® X-4A. The optical rotation values were measured on Hanon instruments P850. High resolution mass spectral data were acquired on Thermo Fisher Q-Exactive-Focus. Enantiomeric excesses were determined on a Thermo Fisher UltiMate 3000 Chiral HPLC. X-ray crystallographic analysis was carried out by Bruker APEX-II CCD. Reflections were merged by SHEXL according to the crystal class for calculation of statistics and refinement.

General procedure A: Synthesis of substrate 1 from 2-substituted-1-ethynylnaphthalene[1]

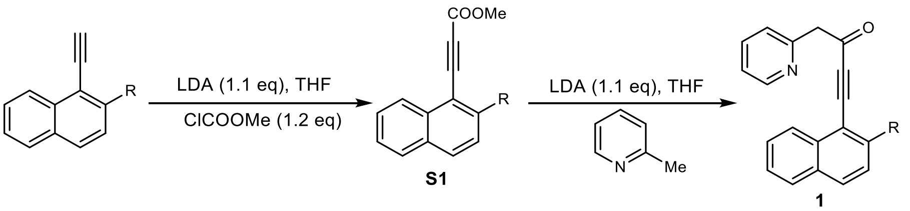

Under an argon atmosphere, to a flame-dried round-bottom flask equipped with a stirring bar was added corresponding 1-ethynylnaphthalene (1.0 eq) and dry THF (0.5 M). After the mixture was cooled down to -78 ºC, LDA (2 M in toluene) (1.1 eq) was added dropwise. The reaction was stirred at same temperature for 30 min. Methyl chloroformate (1.2 eq) was then added into the mixture. When the alkyne reacted completely, the mixture was quenched with saturated NH_4Cl

aqueous. The aqueous layer was extracted with EA (20 mL×3). The combined extracts were washed with brine, dried over anhydrous Na_2SO_4, filtered and concentrated in vacuo. The crude product was purified by column chromatograph over silica gel to afford the corresponding ester **S1**.

Under an argon atmosphere, to a flame-dried round-bottom flask equipped with a stirring bar was added 2-methylpyridine (1.0 eq) and anhydrous THF (0.3 M). After the mixture was cooled down to -78 °C, LDA (2 M in toluene) (1.1 eq) was added dropwise. The reaction mixture was stirred at same temperature for 30 min. Then the above ester **S1** dissolved in THF was added to the reaction dropwise *via* syringe. When the raw material reacted completely, the mixture was quenched with saturated NH_4Cl aqueous. The aqueous layer was extracted with EA (20 mL×3). The combined extracts were washed with brine, dried over anhydrous Na_2SO_4, filtered and concentrated in vacuo. The crude product was purified by column chromatograph over silica gel to afford the substrate.

General procedure B: Synthesis of 2-alkoxy-1-ethynylnaphthalene from 2-naphthol

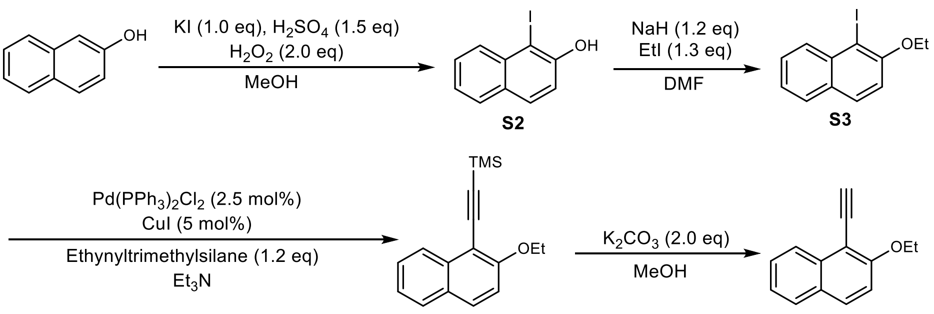

Under an argon atmosphere, to a flame-dried round-bottom flask equipped with a stirring bar was added 2-naphthol (1.0 eq), KI (1.0 eq) and MeOH (0.2 M). The reaction was cooled to 0 °C. Then concentrated H_2SO_4 (1.5 eq) was added at the same temperature. Subsequently, H_2O_2 (30 % in H_2O, 2.0 eq) was added dropwise before the mixture slowly warmed up to room temperature. When the raw material reacted completely, the mixture was filtered. The filtrate was concentrated in vacuo. Then the residue was re-dissolved in water. The aqueous layer was extracted with EA (20 mL×2). The combined extracts were dried over anhydrous Na_2SO_4, filtered and concentrated in vacuo. The crude product was purified by column chromatograph over silica gel to afford **S2**.

Under an argon atmosphere, to a flame-dried round-bottom flask equipped with a stirring bar was added **S2** (1.0 eq) and DMF (0.3 M). The reaction was cooled to 0 °C before NaH (60%

dispersion in mineral oil) (1.2 eq) was added in portions. When the reaction was stirred at the same temperature for 1.0 h, EtI (1.3 eq) was added *via* syringe. The reaction was stirred for another 1.0 h at room temperature. When the raw material reacted completely, the mixture was quenched with saturated NH_4Cl aqueous. The aqueous layer was extracted with EA (20 mL×3). The combined extracts were washed with brine, dried over anhydrous Na_2SO_4, filtered and concentrated in vacuo. The crude product was purified by column chromatograph over silica gel to afford the **S3**.

Under an argon atmosphere, to a flame-dried round-bottom flask equipped with a stirring bar was added **S3** (1.0 eq), ethynyltrimethylsilane (1.2 eq), CuI (5.0 mol%), $Pd(PPh_3)_2Cl_2$ (2.5 mol%) and Et_3N (0.5 M). The mixture was stirred at room temperature overnight. Then the reaction was quenched with saturated NH_4Cl aqueous. The aqueous layer was extracted with EA (20 mL×3). The combined extracts were dried over anhydrous Na_2SO_4, filtered and concentrated in vacuo. The crude product was used directly for next step without purification.

To a flame-dried round-bottom flask equipped with a stirring bar was added above crude product and K_2CO_3 (2.0 eq). The mixture was dissolved in MeOH (0.5 M) and stirred at room temperature for 2.0 h before the solvent was removed in vacuo. Then the residue was re-dissolved in water. The aqueous layer was extracted with EA (20 mL×2). The combined extracts were dried over anhydrous Na_2SO_4, filtered and concentrated in vacuo. The crude product was purified by column chromatograph over silica gel to afford the 1-ethynylnaphthalene. **1a, 1b, 1c, 1d, 1e, 1f, 1g**, **1r, 1s, 1t, 1u, 1v, 1w, 1x, 1y** and **1z** were synthesized from this procedure and general procedure **A**.

General procedure C: Synthesis of 1-ethynyl-*N,N*-dialkylnaphthalen-2-amine from 2-naphthylamine

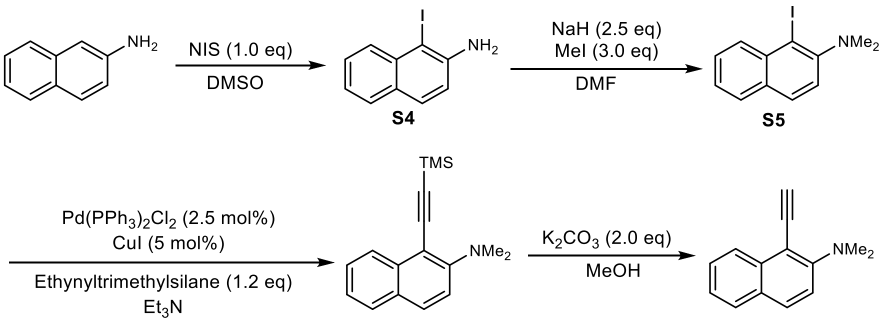

Under an argon atmosphere, to a flame-dried round-bottom flask equipped with a stirring bar was added 2-naphthylamine (1.0 eq), NIS (1.0 eq) and anhydrous DMSO (0.3 M). Then the reaction was stirred at room temperature for 2.0 h before the reaction was quenched with saturated NH_4Cl aqueous. The aqueous layer was extracted with EA (20 mL×3). The combined extracts were dried over anhydrous Na_2SO_4, filtered and concentrated in vacuo. The crude product was purified by column chromatograph over silica gel to afford the **S4**.

Under an argon atmosphere, to a flame-dried round-bottom flask equipped with a stirring bar was added **S4** (1.0 eq) and DMF (0.3 M). The reaction was cooled to 0 ^{o}C before NaH (60% dispersion in mineral oil) (2.5 eq) was added in portions. When the reaction was stirred at the same temperature for 1.0 h, MeI (3.0 eq) was added *via* syringe. The reaction was stirred for another 1.0 h at room temperature. When the raw material reacted completely, the mixture was quenched with saturated NH_4Cl aqueous. The aqueous layer was extracted with EA (20 mL×3). The combined extracts were washed with brine, dried over anhydrous Na_2SO_4, filtered and concentrated in vacuo. The crude product was purified by column chromatograph over silica gel to afford the **S5**.

Under an argon atmosphere, to a flame-dried round-bottom flask equipped with a stirring bar was added **S5** (1.0 eq), ethynyltrimethylsilane (1.2 eq), CuI (5.0 mol%), $Pd(PPh_3)_2Cl_2$ (2.5 mol%) and Et_3N (0.5 M). The mixture was stirred at room temperature overnight. The reaction was then quenched with saturated NH_4Cl aqueous. The aqueous layer was extracted with EA (20 mL×3). The combined extracts were dried over anhydrous Na_2SO_4, filtered and concentrated in vacuo. The crude product was used directly for next step without purification.

To a flame-dried round-bottom flask equipped with a stirring bar was added above crude product and K_2CO_3 (2.0 eq). The mixture was dissolved in MeOH (0.5 M) and stirred at room temperature for 2.0 h before the solvent was removed in vacuo. Then the residue was re-dissolved in water. The aqueous layer was extracted with EA (20 mL×2). The combined extracts were dried over anhydrous Na_2SO_4, filtered and concentrated in vacuo. The crude product was purified by column chromatograph over silica gel to afford the 1-ethynylnaphthalene. **1h** and **1i** were synthesized from this procedure and general procedure **A**.

General procedure D: Synthesis of 2-halide-1-ethynylnaphthalene from 1-Naphthylamine

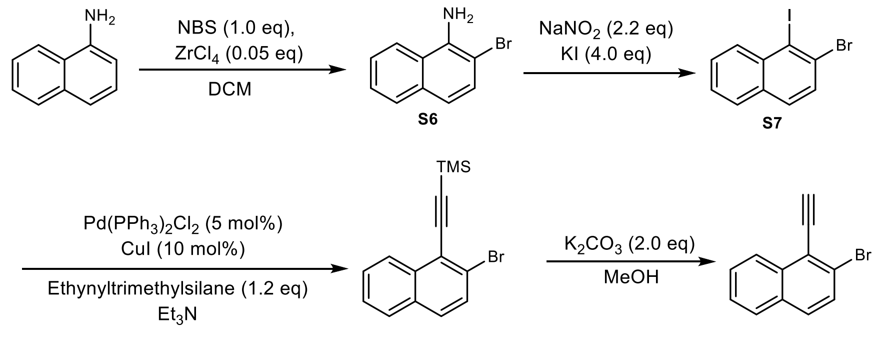

Under an argon atmosphere, to a flame-dried round-bottom flask equipped with a stirring bar was added NBS (1.0 eq) and DCM (0.2 M). The reaction was cooled to -78 °C before $ZrCl_4$ (0.05 eq) and 1-naphthylamine (1.0 eq) was added. The reaction was stirred at the same temperature for 1.0 h before saturated $NaHCO_3$ was added to quench the reaction. The aqueous layer was extracted with DCM (50 mL×2). The combined extracts were dried over anhydrous Na_2SO_4, filtered and concentrated in vacuo. The crude product was purified by column chromatograph over silica gel to afford the **S6**. [2]

To a flame-dried round-bottom flask equipped with a stirring bar was added **S6** (1.0 eq). Then concentrated HCl (4.0 eq) was added slowly. The mixture was stirred at room temperature for 20 min before the reaction was cooled to 0 °C. $NaNO_2$ (2.2 eq) was added into the mixture in portions while keep the temperature of the mixture low than 4 °C. The mixture was stirred at 0 °C for another 30 min before KI (4.0 eq) was added in one portion. The reaction was stirred at room temperature for 1.0 h and 60 °C for another 1.0 h. Then saturated $Na_2S_2O_3$ aqueous was added to quench the reaction. The aqueous layer was extracted with PE (50 mL×2). The combined extracts were dried over anhydrous Na_2SO_4, filtered and concentrated in vacuo. The crude product was purified by column chromatograph over silica gel to afford the **S7**.

Under an argon atmosphere, to a flame-dried round-bottom flask equipped with a stirring bar was added **S7** (1.0 eq), ethynyltrimethylsilane (1.2 eq), CuI (10 mol%), $Pd(PPh_3)_2Cl_2$ (5 mol%) and Et_3N (0.5 M). The mixture was stirred at room temperature overnight. The reaction was then quenched with saturated NH_4Cl aqueous. The aqueous layer was extracted with EA (20 mL×3). The combined extracts were dried over anhydrous Na_2SO_4, filtered and concentrated in vacuo. The crude product was used directly for next step without purification.

To a flame-dried round-bottom flask equipped with a stirring bar was added above crude product and K_2CO_3 (2.0 eq). The mixture was dissolved in MeOH (0.5 M) and stirred at room temperature for 2.0 h before the solvent was removed in vacuo. Then the residue was re-dissolved in water. The aqueous layer was extracted with EA (20 mL×2). The combined extracts were dried over anhydrous Na_2SO_4, filtered and concentrated in vacuo. The crude product was purified by column chromatograph over silica gel to afford the 1-ethynylnaphthalene. **1n, 1o, 1p** and **1q** were synthesized from this procedure and general procedure **A**.

General procedure E: Synthesis of 1-ethynyl-2-alkylnaphthalene from 2-alkylnaphthalene

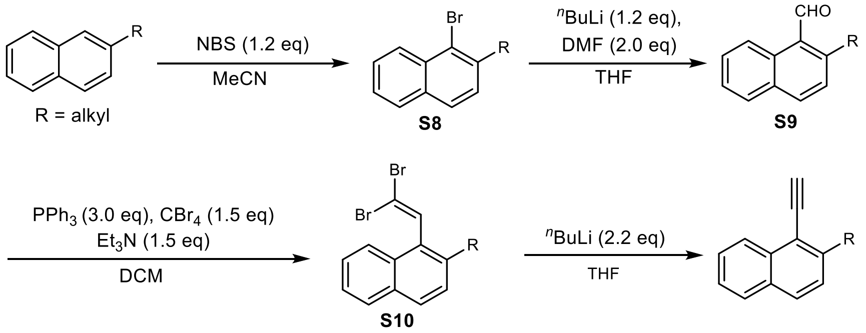

To a flame-dried round-bottom flask equipped with a stirring bar was added 2-alkylnaphthalene (1.0 eq), NBS (1.2 eq) and MeCN (0.5 M). The mixture was stirred at room temperature for 24 h. Then the reaction was quenched with saturated NH_4Cl aqueous. The aqueous layer was extracted with EA (20 mL×3). The combined extracts were dried over anhydrous Na_2SO_4, filtered and concentrated in vacuo. The crude product was purified by column chromatograph over silica gel to afford **S8**.

To a flame-dried round-bottom flask equipped with a stirring bar was added **S8** and THF (0.3 M). The reaction was cooled to -78 °C before ^{n}BuLi (2.5 M in THF) (1.2 eq) was added dropwise. The reaction was stirred at the same temperature for 1.0 h. Then anhydrous DMF (2.0 eq) was added. When the raw material reacted completely, the mixture was quenched with saturated NH_4Cl aqueous. The aqueous layer was extracted with EA (20 mL×3). The combined extracts were washed with brine, dried over anhydrous Na_2SO_4, filtered and concentrated in vacuo. The crude product was purified by column chromatograph over silica gel to afford the **S9**.

To a flame-dried round-bottom flask equipped with a stirring bar was added PPh_3 (3.0 eq), CBr_4 (1.5 eq), Et_3N (1.5 eq) and DCM (0.3 M). The mixture was cooled to 0 °C. Then **S9** (1.0 eq)

dissolved in DCM (2.0 M) was added dropwise. The reaction was stirred at the same temperature for another 1.0 h. Hexane (0.1 M) was then added and the reaction was stirred overnight. When the raw material reacted completely, the mixture was filtered and concentrated in vacuo. The crude product was purified by column chromatograph over silica gel to afford the **S10**.

To a flame-dried round-bottom flask equipped with a stirring bar was added **S10** and THF (0.3 M). The reaction was cooled to -78 °C before ^{n}BuLi (2.5 M in THF) (2.2 eq) was added dropwise. The reaction was stirred at the same temperature for 2.0 h. When the raw material reacted completely, the mixture was quenched with saturated NH_4Cl aqueous. The aqueous layer was extracted with EA (20 mL×3). The combined extracts were washed with brine, dried over anhydrous Na_2SO_4, filtered and concentrated in vacuo. The crude product was purified by column chromatograph over silica gel to afford the 1-ethynylnaphthalene. **1j, 1k, 1l** and **1m** were synthesized from this procedure and general procedure **A**.

1a, 4-(2-methoxynaphthalen-1-yl)-1-(pyridin-2-yl)but-3-yn-2-one / (*Z*)-4-(2-methoxynaphthalen-1-yl)-1-(pyridin-2-yl)but-1-en-3-yn-2-ol

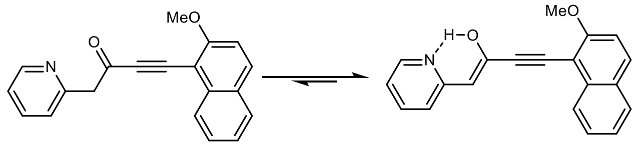

Yellow solid, m.p. 70-71 °C. **^{1}H NMR (500 MHz, Chloroform-*d*) δ:** 8.37 (d, *J* = 8.5 Hz, 1H), 8.34 (s, 1H), 7.90 (d, *J* = 9.1 Hz, 1H), 7.83 (d, *J* = 8.1 Hz, 1H), 7.67 (td, *J* = 7.9, 1.6 Hz, 1H), 7.64 – 7.60 (m, 1H), 7.46 – 7.43 (m, 1H), 7.31 – 7.29 (m, 1H), 7.08 (d, *J* = 8.2 Hz, 1H), 7.05 (dd, *J* = 7.4, 5.5 Hz, 1H), 6.07 (s, 1H, *enol*), 4.10 (s, 3H). (*enol: keto* = 2.7 :1) **^{13}C NMR (126 MHz, Chloroform-*d*) δ**: 160.85, 158.66, 156.81, 148.81, 148.76, 143.19, 136.39, 135.68, 133.51, 132.53, 130.08, 127.39, 127.28, 127.25, 127.11, 126.62, 124.26, 123.79, 123.67, 123.58, 123.32, 120.40, 118.00, 111.53, 111.18, 103.98, 102.78, 94.88, 83.82, 55.61, 55.50, 53.58. **HRMS(ESI) m/z**: calculated for $[C_{20}H_{15}NO_2 + H]^+$ 302.1181, found 302.1179.

1b, 4-(2-ethoxynaphthalen-1-yl)-1-(pyridin-2-yl)but-3-yn-2-one / (*Z*)-4-(2-ethoxynaphthalen-1-yl)-1-(pyridin-2-yl)but-1-en-3-yn-2-ol

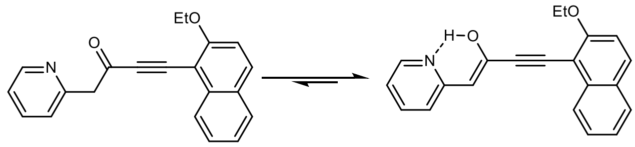

Yellow solid, m.p. 75-76 °C. **^{1}H NMR (500 MHz, Chloroform-*d*) δ**: 8.38 (d, *J* = 8.5 Hz, 1H), 8.33 (d, *J* = 5.3 Hz, 1H), 7.87 (d, *J* = 9.1 Hz, 1H), 7.84 – 7.80 (m, 1H), 7.67 (td, *J* = 7.8, 1.7 Hz, 1H), 7.61 (t, *J* = 7.6 Hz, 1H), 7.44 (dt, *J* = 13.3, 7.0 Hz, 2H), 7.28 (d, *J* = 8.9 Hz, 1H), 7.08 (d, *J* = 8.1 Hz, 1H), 7.05 (dd, *J* = 7.3, 5.5 Hz, 1H), 6.04 (s, 1H, *enol*), 4.36 – 4.34 (m, 2H), 1.59 (t, *J* = 7.0 Hz, 3H). (*enol: keto* = 2.5 :1) **^{13}C NMR (126 MHz, Chloroform-*d*) δ**: 158.23, 156.84, 148.95, 148.76, 143.19, 136.37, 135.65, 133.91, 133.53, 132.40, 129.92, 127.47, 127.24, 127.16, 127.06, 126.50, 124.35, 123.86, 123.61, 123.56, 123.34, 121.23, 120.39, 117.97, 113.29, 112.48, 104.76, 102.70, 96.67, 94.78, 84.02, 64.43, 64.20, 53.62, 14.10, 13.96. **HRMS(ESI) m/z**: calculated for $[C_{21}H_{17}NO_2 + H]^+$ 316.1138 , found 316.1128.

1c, 4-(6-bromo-2-ethoxynaphthalen-1-yl)-1-(pyridin-2-yl)but-3-yn-2-one / (*Z*)-4-(6-bromo-2-ethoxynaphthalen-1-yl)-1-(pyridin-2-yl)but-1-en-3 -yn-2-ol.

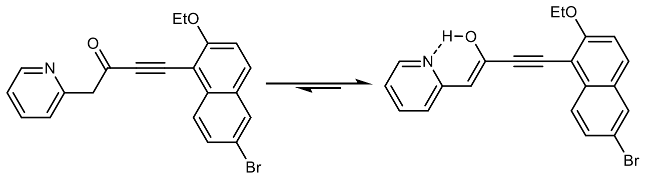

Yellow solid, m.p. 56-57 °C. **^{1}H NMR (500 MHz, Chloroform-*d*) δ:** 8.34 – 8.27 (m, 1H), 8.20 (d, *J* = 9.0 Hz, 1H), 7.91 (d, *J* = 2.0 Hz, 1H), 7.70 (d, *J* = 9.1 Hz, 1H), 7.63 (ddd, *J* = 8.8, 6.0, 1.9 Hz, 2H), 7.23 (d, *J* = 9.1 Hz, 1H), 7.06 (d, *J* = 8.1 Hz, 1H), 7.04 – 6.99 (m, 1H), 6.01 (s, 1H, *enol*), 4.29 (q, *J* = 6.9 Hz, 2H), 1.55 (t, *J* = 7.0 Hz, 3H). (*enol: keto* = 2.6 :1) **^{13}C NMR (126 MHz, Chloroform-*d*) δ:** 158.82, 156.76, 148.93, 148.74, 143.17, 136.40, 134.70, 129.74, 128.80, 128.66, 127.09, 126.75, 126.47, 125.96, 125.79, 123.47, 121.34, 121.25, 120.46, 118.06, 113.34, 103.98, 102.90, 95.10, 83.14, 64.36, 64.24, 53.70, 14.01, 13.88. **HRMS(ESI) m/z**: calculated for $[C_{21}H_{16}BrNO_2 + H]^+$ 394.0443 , found 394.0442.

1d, 4-(7-bromo-2-ethoxynaphthalen-1-yl)-1-(pyridin-2-yl)but-3-yn-2-one / (*Z*)-4-(7-bromo-2 -ethoxynaphthalen-1-yl)-1-(pyridin-2-yl)but-1-en-3- yn -2-ol

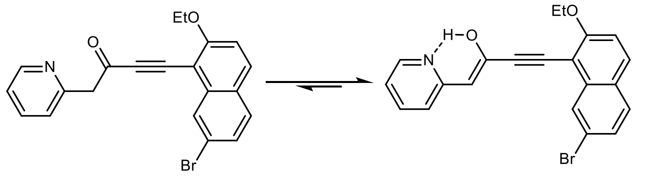

Yellow solid, m.p. 100-101 °C. **^{1}H NMR (500 MHz, Chloroform-*d*) δ:** 8.48 (d, *J* = 1.9 Hz, 1H), 8.34 (dt, *J* = 5.2, 1.2 Hz, 1H), 7.81 (d, *J* = 9.0 Hz, 1H), 7.70 – 7.64 (m, 2H), 7.49 (dd, *J* = 8.7, 2.0 Hz, 1H), 7.27 (d, *J* = 9.0 Hz, 1H), 7.10 (dt, *J* = 8.1, 1.1 Hz, 1H), 7.07 (ddd, *J* = 7.4, 5.2, 1.1 Hz, 1H), 6.04 (s, 1H, *enol*), 4.37 – 4.31 (m, 2H), 1.58 (t, *J* = 6.9 Hz, 3H). (*enol: keto* = 3.8 :1) **^{13}C NMR (126 MHz, Chloroform-*d*) δ:** 158.82, 156.76, 148.93, 148.74, 143.17, 136.40, 134.70, 129.74, 128.80, 128.66, 127.09, 126.75, 126.47, 125.96, 125.79, 123.47, 121.34, 121.25, 120.46, 118.06, 113.34, 103.98, 102.90, 95.10, 83.14, 64.36, 64.24, 53.70, 14.01, 13.88. **HRMS(ESI) m/z**: calculated for $[C_{21}H_{16}BrNO_2 + H]^+$ 394.0443 , found 394.0440.

1e, 4-(2-ethoxy-7-phenylnaphthalen-1-yl)-1-(pyridin-2-yl)but-3-yn-2-one / (*Z*)-4-(2-ethoxy-7-phenylnaphthalen-1-yl)-1-(pyridin-2-yl)but-1-en-3- yn-2-ol

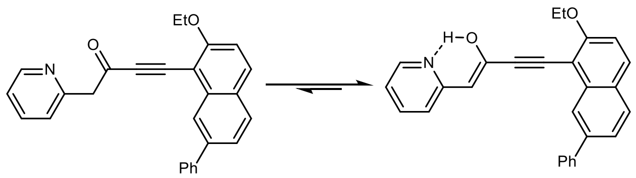

Yellow solid, m.p. 62-63 °C. **^{1}H NMR (500 MHz, Chloroform-*d*) δ**: 8.62 (d, *J* = 1.8 Hz, 1H), 8.29 (d, *J* = 5.4 Hz, 1H), 7.89 – 7.81 (m, 3H), 7.81 (d, *J* = 7.7 Hz, 1H), 7.71 (dd, *J* = 8.4, 1.8 Hz, 1H), 7.63 – 7.56 (m, 3H), 7.47 (t, *J* = 7.4 Hz, 1H), 7.25 (d, *J* = 9.0 Hz, 1H), 7.03 (d, *J* = 8.1 Hz, 1H), 7.01 – 6.99 (m, 1H), 6.06 (s, 1H, *enol*), 4.33 (q, *J* = 7.0 Hz, 2H), 1.60 (t, *J* = 7.0 Hz, 3H). (*enol: keto* = 2.8 :1) **^{13}C NMR (126 MHz, Chloroform-*d*) δ**: 160.83, 158.70, 156.71, 153.03, 149.23, 148.71, 143.02, 140.10, 139.96, 139.79, 139.13, 136.46, 135.64, 134.16, 133.85, 132.19, 129.75, 127.97, 127.92, 127.77, 126.81, 126.69, 126.66, 126.60, 126.32, 123.51, 123.34, 123.06, 122.15, 121.57, 121.22, 120.43, 118.00, 113.10, 112.28, 104.86, 102.70, 101.99, 96.22, 88.05, 84.13, 64.34, 64.14, 53.73, 14.14, 13.98. **HRMS(ESI) m/z**: calculated for $[C_{27}H_{11}NO_2 + H]^+$ 392.1651 , found 392.1644.

1f, 4-(2-ethoxy-7-(phenylethynyl)naphthalen-1-yl)-1-(pyridin-2-yl)but-3-yn-2-one / (*Z*)-4-(2 -ethoxy-7-(phenylethynyl)naphthalen-1-yl)-1-(pyridin-2-yl)but-1-en-3-yn-2-ol

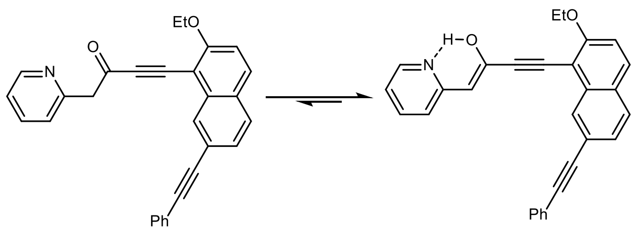

Yellow solid, m.p. 119-120 °C. ^{1}**H NMR (500 MHz, Chloroform-*d*) δ**: 8.54 (d, *J* = 1.4 Hz, 1H), 8.35 – 8.34 (m, 1H), 7.84 (d, *J* = 9.0 Hz, 1H), 7.79 (d, *J* = 8.5 Hz, 1H), 7.68 (ddt, *J* = 9.7, 4.4, 2.0 Hz, 3H), 7.54 (dd, *J* = 8.3, 1.8 Hz, 1H), 7.45 – 7.41 (m, 3H), 7.30 (d, *J* = 10.9 Hz, 1H), 7.10 (d, *J* = 8.2 Hz, 1H), 7.07 – 7.05 (m, 1H), 6.08 (s, 1H, *enol*), 4.38 – 4.35 (m, 3H), 1.60 (t, *J* = 6.9 Hz, 3H), 1.56 (t, *J* = 7.0 Hz, 1H). (*enol: keto* = 2.5 :1) ^{13}**C NMR (126 MHz, Chloroform-*d*) δ**: 160.84, 158.78, 156.82, 148.90, 143.19, 136.37, 135.71, 133.21, 131.97, 130.77, 130.75, 129.60, 127.75, 127.55, 127.45, 127.34, 127.16, 127.11, 126.77, 126.34, 126.04, 123.52, 122.26, 121.28, 120.45, 118.01, 113.70, 102.92, 95.19, 89.55, 89.08, 83.52, 64.40, 64.23, 53.71, 14.06, 13.93. **HRMS(ESI) m/z**: calculated for $[C_{29}H_{21}NO_2 + H]^+$ 416.1651 , found 416.1649.

1g, 4-(2-((4-methoxybenzyl)oxy)naphthalen-1-yl)-1-(pyridin-2-yl)but-3-yn-2-one / (*Z*)-4-(2-((4-methoxybenzyl)oxy)naphthalen-1-yl)-1-(pyridin-2-yl)but -1-en-3-yn-2-ol.

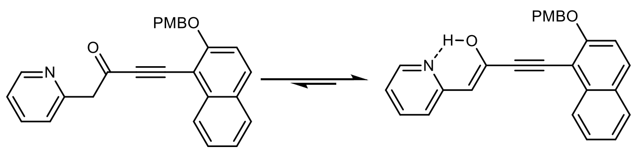

Yellow solid, m.p. 85-86 °C. ^{1}**H NMR (500 MHz, Chloroform-*d*) δ**: 8.41 (d, *J* = 8.4 Hz, 1H), 8.30 (d, *J* = 5.1 Hz, 1H), 7.81 (dd, *J* = 8.7, 3.4 Hz, 2H), 7.63 (dd, *J* = 7.9, 1.6 Hz, 2H), 7.54 (d, *J* = 8.3 Hz, 2H), 7.46 (d, *J* = 7.9 Hz, 1H), 7.28 (d, *J* = 9.0 Hz, 1H), 7.03 – 6.99 (m, 2H), 6.96 (t, *J* = 8.1 Hz, 2H), 5.99 (s, 1H, *enol*), 5.31 (s, 2H), 3.83 (s, 3H). (*enol: keto* = 3.2 :1) ^{13}**C NMR (126 MHz, Chloroform-*d*) δ**: 160.20, 158.46, 158.31, 158.13, 156.77, 153.08, 148.90, 143.20, 136.43, 135.69, 133.82, 133.40, 132.39, 129.91, 128.05, 127.83, 127.81, 127.69, 127.41, 127.32, 127.20, 127.15, 126.58, 124.39, 123.86, 123.75, 123.67, 123.57, 121.24, 120.39, 118.05, 114.05, 113.17, 113.03, 112.91, 105.51, 102.93, 96.82, 95.18, 87.85, 84.13, 70.32, 70.00, 54.27, 53.60. **HRMS(ESI) m/z**: calculated for $[C_{27}H_{11}NO_3 + H]^+$ 408.1600, found 408.1591.

1h, 4-(2-(dimethylamino)naphthalen-1-yl)-1-(pyridin-2-yl)but-3-yn-2-one / (*Z*)-4-(2-(dimethyl amino)naphthalen-1-yl)-1-(pyridin-2-yl)but-1-en-3 -yn-2-ol

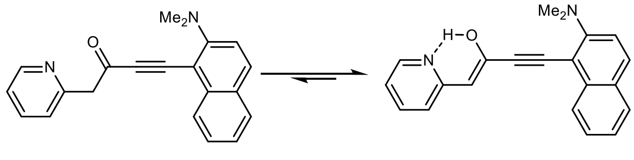

Yellow solid, m.p. 78-79 °C. ^{1}**H NMR (500 MHz, Chloroform-*d*) δ**: 8.69 (dd, *J* = 5.0, 1.8 Hz, 1H), 8.42 (d, *J* = 8.4 Hz, 1H), 8.32 – 8.20 (m, 1H), 7.96 (d, *J* = 8.5 Hz, 1H), 7.74 (ddd, *J* = 16.4,

7.0, 3.2 Hz, 3H), 7.59 (dtd, *J* = 12.1, 7.3, 6.7, 1.5 Hz, 2H), 7.44 – 7.35 (m, 2H), 7.22 (d, *J* = 9.1 Hz, 1H), 7.08 (d, *J* = 9.2 Hz, 1H), 7.03 (d, *J* = 8.1 Hz, 1H), 7.01 – 6.95 (m, 1H), 6.00 (s, 1H, *enol*), 4.30 (s, 1H, *keto*), 3.21 (s, 6H). (*enol: keto* = 1.4 :1) **^{13}C NMR (126 MHz, Chloroform-*d*) δ**: 156.79, 155.56, 153.48, 153.39, 149.60, 148.75, 142.95, 136.42, 135.72, 135.07, 134.29, 131.69, 129.26, 127.09, 127.02, 126.98, 126.97, 126.42, 126.21, 123.85, 123.60, 123.19, 122.74, 122.62, 121.25, 120.32, 117.81, 116.73, 116.25, 103.95, 101.81, 99.50, 99.21, 96.71, 92.95, 87.48, 53.11, 42.63, 42.50, 28.75. **HRMS(ESI) m/z**: calculated for $[C_{21}H_{18}N_2O + H]^+$ 315.1497, found 315.1487.

1i, 4-(2-(dibenzylamino)naphthalen-1-yl)-1-(pyridin-2-yl)but-3-yn-2-one / (*Z*)-4-(2-(dibenzyl amino)naphthalen-1-yl)-1-(pyridin-2-yl)but-1-en-3- yn-2-ol

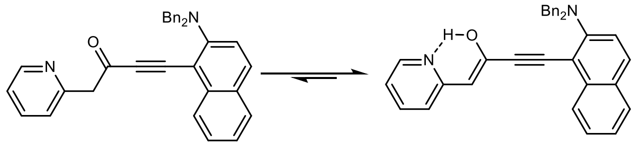

Yellow solid, m.p. 56-57 °C. **^{1}H NMR (500 MHz, Chloroform-*d*) δ**: 8.49 (d, *J* = 8.4 Hz, 1H), 8.35 – 8.28 (m, 1H), 7.77 (d, *J* = 8.2 Hz, 1H), 7.70 (d, *J* = 9.0 Hz, 1H), 7.46 (d, *J* = 7.4 Hz, 5H), 7.41 – 7.34 (m, 4H), 7.32 – 7.29 (m, 4H), 7.21 (d, *J* = 9.0 Hz, 1H), 7.04 (ddd, *J* = 7.4, 5.2, 1.1 Hz, 1H), 6.93 (d, *J* = 8.1 Hz, 1H), 5.49 (s, 1H, *enol*), 4.74 (s, 4H). (*enol: keto* = 3.2 :1) **^{13}C NMR (126 MHz, Chloroform-*d*) δ**: 156.92, 154.16, 153.21, 151.67, 148.75, 143.29, 137.64, 136.98, 136.25, 135.59, 134.81, 134.17, 131.27, 129.00, 128.91, 128.26, 127.72, 127.51, 127.40, 127.26, 127.15, 127.06, 127.00, 126.91, 126.89, 126.86, 126.39, 126.22, 126.02, 125.93, 124.38, 123.80, 123.46, 123.37, 121.16, 120.39, 119.46, 119.12, 117.89, 107.33, 102.38, 98.97, 96.76, 90.41, 86.77, 55.43, 55.27, 52.92. **HRMS(ESI) m/z**: calculated for $[C_{33}H_{26}N_2O + H]^+$ 467.2123, found 467.2116.

1j, 4-(2-methylnaphthalen-1-yl)-1-(pyridin-2-yl)but-3-yn-2-one / (Z)-4-(2-methylnaphthalen-1-yl) -1-(pyridin-2-yl)but-1-en-3-yn-2-ol

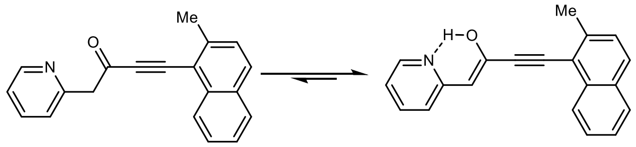

Yellow solid, m.p. 69-70 °C. **^{1}H NMR (500 MHz, Chloroform-*d*) δ**: 8.45 (dd, *J* = 8.4, 1.0 Hz, 1H), 8.34 (dt, *J* = 5.3, 1.3 Hz, 1H), 7.86 (dd, *J* = 8.1, 4.5 Hz, 1H), 7.81 (d, *J* = 8.3 Hz, 1H), 7.68 (td, *J* = 7.8, 1.8 Hz, 1H), 7.63 (ddd, *J* = 8.3, 6.8, 1.3 Hz, 1H), 7.52 (ddd, *J* = 8.0, 6.8, 1.2 Hz, 1H),

7.43 (dd, *J* = 12.4, 8.2 Hz, 1H), 7.09 (dt, *J* = 8.1, 1.1 Hz, 1H), 7.06 (ddd, *J* = 7.4, 5.3, 1.2 Hz, 1H), 6.06 (s, 1H, *enol*), 2.77 (s, 3H). (*enol: keto* = 3.9 :1) **^{13}C NMR (126 MHz, Chloroform-*d*) δ**: 156.72, 153.04, 148.89, 148.80, 143.15, 142.10, 139.25, 136.45, 136.00, 135.78, 132.99, 132.57, 130.46, 130.30, 130.06, 128.33, 127.95, 127.83, 127.21, 127.06, 126.96, 126.88, 126.59, 126.03, 124.14, 123.58, 123.30, 121.36, 120.42, 118.10, 117.09, 102.78, 96.01, 94.68, 85.96, 53.63, 20.44, 20.32. **HRMS(ESI) m/z**: calculated for $[C_{20}H_{15}NO + H]^+$ 286.1232, found 286.1227.

1k, 4-(2-ethylnaphthalen-1-yl)-1-(pyridin-2-yl)but-3-yn-2-one / (*Z*)-4-(2-ethylnaphthalen-1-yl)-1-(pyridin-2-yl)but-1-en-3-yn-2-ol

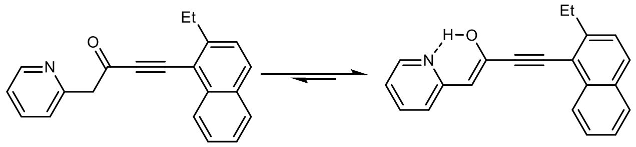

Yellow solid, m.p. 77-78 °C. **^{1}H NMR (500 MHz, Chloroform-*d*) δ**: 8.49 (d, *J* = 8.4 Hz, 1H), 8.31 (dd, *J* = 5.4, 1.7 Hz, 1H), 7.86 (dd, *J* = 10.1, 8.2 Hz, 2H), 7.65 (td, *J* = 7.6, 1.5 Hz, 2H), 7.54 (ddd, *J* = 8.1, 6.8, 1.2 Hz, 1H), 7.45 (d, *J* = 8.5 Hz, 1H), 7.07 (d, *J* = 8.1 Hz, 1H), 7.03 (ddd, *J* = 6.7, 5.4, 1.1 Hz, 1H), 6.05 (s, 1H, *enol*), 3.16 (q, *J* = 7.6 Hz, 2H), 1.44 (t, *J* = 7.6 Hz, 3H). (*enol: keto* = 3.8 :1) **^{13}C NMR (126 MHz, Chloroform-*d*) δ**: 156.66, 153.04, 148.94, 148.21, 145.36, 143.06, 136.50, 135.81, 133.08, 130.57, 130.49, 130.39, 128.37, 127.24, 127.10, 126.60, 126.05, 125.61, 125.08, 125.03, 124.72, 124.62, 123.58, 121.38, 120.44, 118.11, 116.32, 113.96, 102.76, 94.30, 85.68, 53.65, 27.54, 27.37, 14.36, 14.26. **HRMS(ESI) m/z**: calculated for $[C_{21}H_{17}NO + H]^+$ 300.1388, found 300.1385.

1l, 4-(2-butylnaphthalen-1-yl)-1-(pyridin-2-yl)but-3-yn-2-one / (*Z*)-4-(2-butylnaphthalen-1-yl) -1-(pyridin-2-yl)but-1-en-3-yn-2-ol

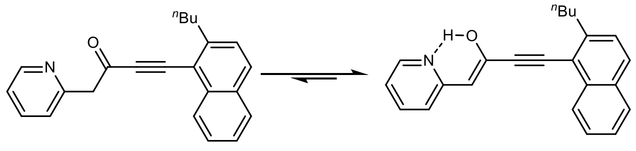

Yellow solid, m.p. 48-49 °C. **^{1}H NMR (500 MHz, Chloroform-*d*) δ**: 8.47 (d, *J* = 8.4 Hz, 1H), 8.37 – 8.34 (m, 1H), 7.85 (dd, *J* = 15.2, 8.4 Hz, 2H), 7.69 (td, *J* = 7.8, 1.7 Hz, 1H), 7.64 (ddd, *J* = 8.3, 6.8, 1.2 Hz, 1H), 7.53 (td, *J* = 7.4, 6.6, 1.2 Hz, 1H), 7.43 (d, *J* = 8.5 Hz, 1H), 7.10 (d, *J* = 8.1 Hz, 1H), 7.07 – 7.05 (m, 1H), 6.03 (s, 1H, *enol*), 3.12 (t, *J* = 7.7 Hz, 2H), 1.84 – 1.78 (m, 2H), 1.54 – 1.50 (m, 2H), 1.05 (t, *J* = 7.4 Hz, 3H). (*enol: keto* = 3.8:1) **^{13}C NMR (126 MHz, Chloroform-*d*) δ**:

156.75, 148.90, 148.85, 144.15, 143.17, 136.45, 135.76, 133.06, 132.64, 130.53, 130.18, 128.08, 127.20, 127.04, 126.54, 126.27, 125.99, 125.11, 124.67, 123.53, 121.34, 120.42, 118.10, 116.70, 102.72, 94.14, 85.91, 53.61, 34.02, 33.89, 32.20, 32.14, 21.61, 21.43, 13.08, 13.05. **HRMS(ESI) m/z**: calculated for $[C_{23}H_{21}NO + H]^+$ 328.1701, found 328.1699.

1m, 4-(2-isopropylnaphthalen-1-yl)-1-(pyridin-2-yl)but-3-yn-2-one / (*Z*)-4-(2-isopropylnaphthalen-1-yl)-1-(pyridin-2-yl)but-1-en-3-yn-2-ol

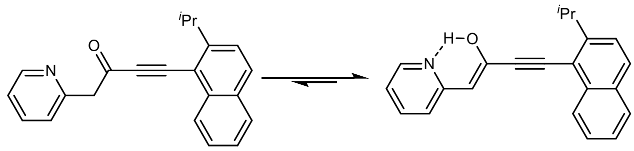

Yellow oil. **^{1}H NMR (500 MHz, Chloroform-*d*) δ**: 8.52 (d, *J* = 8.4 Hz, 1H), 8.32 – 8.30(m, 1H), 7.88 (dd, *J* = 10.8, 8.4 Hz, 2H), 7.66 – 7.63 (m, 2H), 7.53 (d, *J* = 8.4 Hz, 2H), 7.15 – 7.06 (m, 1H), 7.03 (ddd, *J* = 7.4, 5.3, 1.2 Hz, 1H), 6.06 (s, 1H, *enol*), 3.92 (p, *J* = 6.9 Hz, 1H), 1.45 (d, *J* = 7.0 Hz, 6H). (*enol: keto* = 3.8 :1) **^{13}C NMR (126 MHz, Chloroform-*d*) δ**: 156.66, 153.07, 152.26, 149.37, 148.98, 148.92, 145.36, 143.03, 136.51, 135.79, 133.00, 130.75, 130.62, 128.63, 127.08, 126.63, 126.08, 125.62, 125.29, 124.80, 123.59, 122.27, 122.17, 121.37, 120.44, 118.11, 115.81, 102.75, 95.97, 94.66, 88.85, 85.61, 53.65, 52.55, 31.35, 31.29, 27.54, 22.99, 22.34, 14.27. **HRMS(ESI) m/z**: calculated for $[C_{22}H_{19}NO + H]^+$ 314.1545, found 314.1541.

1n, 4-(2-phenylnaphthalen-1-yl)-1-(pyridin-2-yl)but-3-yn-2-one / (*Z*)-4-(2-phenylnaphthalen-1-yl)-1-(pyridin-2-yl)but-1-en-3-yn-2-ol

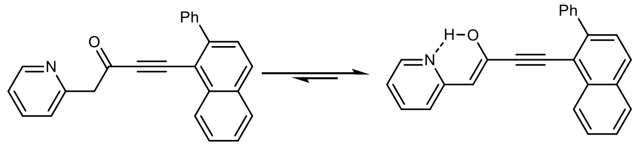

Yellow solid, m.p. 70-71 °C. **^{1}H NMR (500 MHz, Chloroform-*d*) δ**: 8.62 (d, *J* = 8.6 Hz, 1H), 8.31 (dd, *J* = 5.4, 1.7 Hz, 1H), 7.97 (d, *J* = 8.5 Hz, 1H), 7.94 (d, *J* = 8.3 Hz, 1H), 7.82 (dd, *J* = 7.4, 1.7 Hz, 2H), 7.73 – 7.71 (m, 1H), 7.68 – 7.62 (m, 2H), 7.62 (d, *J* = 4.5 Hz, 1H), 7.61 – 7.55 (m, 2H), 7.50 (dd, *J* = 8.5, 6.3 Hz, 1H), 7.04 (dd, *J* = 7.4, 5.4 Hz, 1H), 7.01 (d, *J* = 8.1 Hz, 1H), 5.73 (s, 1H, *enol*). (*enol: keto* = 4.2 :1) **^{13}C NMR (126 MHz, Chloroform-*d*) δ**: 156.68, 148.73, 148.51, 143.21, 142.34, 139.74, 136.36, 135.62, 132.69, 131.10, 130.36, 128.83, 128.66, 128.35, 127.26, 127.20, 127.07, 127.02, 126.66, 126.47, 126.43, 126.38, 125.83, 125.52, 125.33, 123.46, 121.19,

120.40, 118.10, 116.18, 103.10, 93.54, 86.92, 53.28. **HRMS(ESI) m/z**: calculated for [$C_{25}H_{17}NO$ + H]$^+$ 348.1388, found 348.1386.

1o, 4-([1,2'-binaphthalen]-1'-yl)-1-(pyridin-2-yl)but-3-yn-2-one / (Z)-4-([1,2'-binaphthalen]-1'-yl)-1-(pyridin-2-yl)but-1-en-3-yn-2-ol

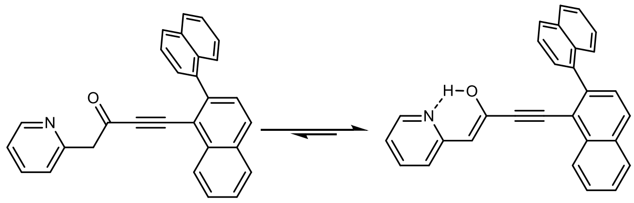

Yellow solid, m.p. 76-77 °C. **^{1}H NMR (500 MHz, Chloroform-*d*) δ**: 8.66 (d, *J* = 8.3 Hz, 1H), 8.19 (d, *J* = 5.3 Hz, 1H), 8.03 (dt, *J* = 13.4, 7.2 Hz, 4H), 7.81 (d, *J* = 8.5 Hz, 1H), 7.74 (q, *J* = 7.1 Hz, 1H), 7.72 – 7.63 (m, 4H), 7.62 – 7.53 (m, 2H), 7.50 (t, *J* = 7.7 Hz, 1H), 7.95 – 6.93 (m, 1H), 6.79 (d, *J* = 8.1 Hz, 1H), 4.95 (d, *J* = 1.6 Hz, 1H, *enol*). (*enol: keto* = 3.3 :1) **^{13}C NMR (126 MHz, Chloroform-*d*) δ**: 156.46, 152.38, 148.54, 148.08, 144.04, 143.09, 141.66, 137.94, 137.18, 136.25, 135.41, 132.69, 132.67, 132.39, 131.37, 130.87, 129.96, 127.87, 127.65, 127.52, 127.46, 127.43, 127.23, 127.17, 127.15, 127.02, 126.98, 126.54, 126.10, 125.82, 125.76, 125.54, 125.39, 125.32, 125.15, 125.11, 125.06, 124.81, 124.30, 124.27, 123.25, 121.01, 120.31, 118.51, 117.99, 103.17, 94.78, 94.32, 86.68, 53.03. **HRMS(ESI) m/z**: calculated for [$C_{29}H_{19}NO$ + H]$^+$ 398.1545, found 398.1546.

1p, 4-(2-bromonaphthalen-1-yl)-1-(pyridin-2-yl)but-3-yn-2-one / (Z)-4-(2-bromonaphthalen-1-yl)-1-(pyridin-2-yl)but-1-en-3-yn-2-ol

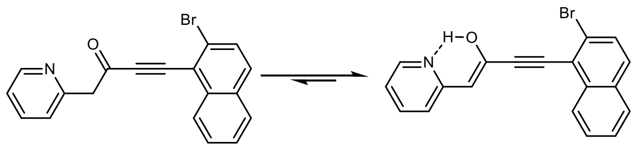

Yellow solid, m.p. 106-107 °C. **^{1}H NMR (500 MHz, Chloroform-*d*) δ**: 8.40 (dd, *J* = 8.3, 1.1 Hz, 1H), 8.31 – 8.30 (m, 1H), 7.81 (d, *J* = 8.0 Hz, 1H), 7.75 (d, *J* = 8.8 Hz, 1H), 7.71 (d, *J* = 3.5 Hz, 1H), 7.70 – 7.65 (m, 2H), 7.62 – 7.57 (m, 1H), 7.13 – 7.11 (m, 1H), 7.10 – 7.07 (m, 1H), 6.06 (s, 1H, *enol*). (*enol: keto* = 10.5 :1) **^{13}C NMR (126 MHz, Chloroform-*d*) δ**: 156.57, 148.24, 143.19, 136.54, 133.42, 130.60, 129.10, 128.55, 127.24, 126.97, 125.79, 125.39, 124.30, 120.60, 120.29, 118.35, 103.59, 94.85, 85.57, 53.55. **HRMS(ESI) m/z**: calculated for [$C_{19}H_{12}BrNO$ + H]$^+$ 350.0181, found 350.0171.

1q, 4-(2-chloronaphthalen-1-yl)-1-(pyridin-2-yl)but-3-yn-2-one / (*Z*)-4-(2-chloronaphthalen-1-yl) -1-(pyridin-2-yl)but-1-en-3-yn-2-ol

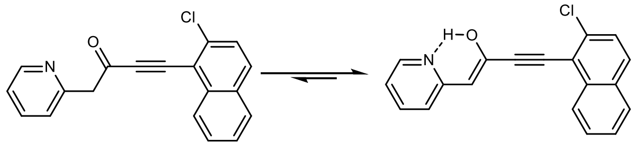

Yellow solid, m.p. 111-112 °C. **^{1}H NMR (500 MHz, Chloroform-*d*) δ**: 8.43 (dd, *J* = 8.4, 1.0 Hz, 1H), 8.36 (dt, *J* = 5.2, 1.2 Hz, 1H), 7.88 (d, *J* = 8.1 Hz, 1H), 7.82 (d, *J* = 8.7 Hz, 1H), 7.72 – 7.66 (m, 2H), 7.58 (ddd, *J* = 8.2, 6.9, 1.2 Hz, 1H), 7.55 (d, *J* = 8.7 Hz, 1H), 7.13 – 7.09 (m, 2H), 6.11 (s, 1H, *enol*). (*enol: keto* = 6.3 :1) **^{13}C NMR (126 MHz, Chloroform-*d*) δ**: 156.59, 148.14, 143.24, 136.52, 134.45, 133.09, 130.32, 129.09, 127.20, 126.96, 125.83, 125.65, 125.19, 120.60, 118.36, 117.77, 103.62, 98.94, 95.55, 83.71. **HRMS(ESI) m/z**: calculated for $[C_{19}H_{12}ClNO + Na]^+$ 328.0505, found 328.0503.

1r, 1-(3-bromopyridin-2-yl)-4-(2-ethoxynaphthalen-1-yl)but-3-yn-2-one / (*Z*)-1-(3-bromopyridin -2-yl)-4-(2-ethoxynaphthalen-1-yl)but-1-en-3- yn-2-ol

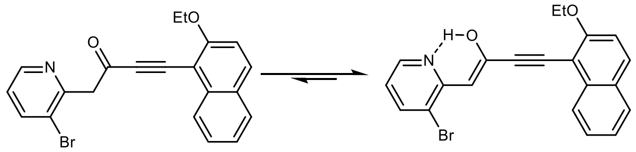

Yellow solid, m.p. 86-87 °C. **^{1}H NMR (500 MHz, Chloroform-*d*) δ**: 8.36 (d, *J* = 8.5 Hz, 1H), 8.34 (dd, *J* = 4.9, 1.4 Hz, 1H), 7.92 (dd, *J* = 7.9, 1.4 Hz, 1H), 7.89 (d, *J* = 9.0 Hz, 1H), 7.83 (d, *J* = 8.2 Hz, 1H), 7.63 (ddd, *J* = 8.3, 6.8, 1.2 Hz, 1H), 7.46 – 7.43 (m, 2H), 7.29 (d, *J* = 9.0 Hz, 1H), 6.53 (s, 1H, *enol*), 4.55 (s, 1H, *keto*), 4.37 (t, *J* = 7.0 Hz, 2H), 1.61 (t, *J* = 7.0 Hz, 3H). (*enol:keto* = 2.7 :1) **^{13}C NMR (126 MHz, Chloroform-*d*) δ**: 160.44, 158.53, 155.46, 149.17, 147.10, 142.78, 140.07, 139.35, 133.89, 133.45, 132.37, 130.20, 127.46, 127.25, 127.12, 127.08, 126.61, 124.30, 123.83, 123.55, 123.39, 122.71, 121.63, 119.05, 115.71, 113.24, 112.58, 104.53, 101.47, 94.50, 85.41, 64.47, 64.2, 53.11, 52.43, 14.08, 13.95. **HRMS(ESI) m/z**: calculated for $[C_{21}H_{16}BrNO_2 + H]^+$ 394.0443, found 394.0442.

1s, 1-(4-bromopyridin-2-yl)-4-(2-ethoxynaphthalen-1-yl)but-3-yn-2-one / (*Z*)-1-(4-bromopyridin -2-yl)-4-(2-ethoxynaphthalen-1-yl)but-1-en-3- yn-2-ol

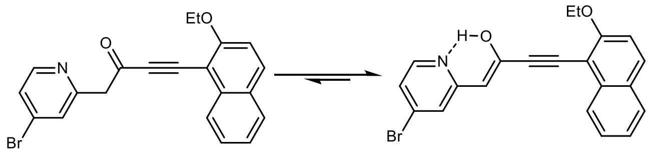

Yellow solid, m.p. 81-82 °C. **^{1}H NMR (500 MHz, Chloroform-*d*) δ**: 8.35 – 8.33 (m, 1H), 8.20 (d, *J* = 5.6 Hz, 1H), 7.88 (d, *J* = 9.0 Hz, 1H), 7.84 – 7.82 (m, 1H), 7.62 (ddd, *J* = 8.3, 6.8, 1.2 Hz, 1H), 7.45 (ddd, *J* = 8.1, 6.7, 1.2 Hz, 2H), 7.29 – 7.28 (m, 1H), 7.23 (dd, *J* = 5.6, 1.8 Hz, 1H), 5.98 (s, 1H, *enol*), 4.38 – 4.35 (m, 2H), 1.60 – 1.56 (m, 3H). (*enol: keto* = 2.2 :1) **^{13}C NMR (126 MHz, Chloroform-*d*) δ**: 160.52, 158.40, 158.02, 154.55, 149.39, 148.69, 144.89, 133.90, 133.49, 132.73, 132.57, 130.19, 127.44, 127.31, 127.28, 127.15, 127.09, 126.92, 126.61, 124.66, 124.23, 123.75, 123.62, 123.38, 123.10, 121.54, 113.16, 112.41, 104.44, 102.43, 96.54, 94.12, 88.50, 64.40, 64.20, 53.05, 14.08, 13.96. **HRMS(ESI) m/z**: calculated for $[C_{21}H_{16}BrNO_2 + H]^+$ 416.0262, found 416.0257.

1t, 1-(4-chloropyridin-2-yl)-4-(2-ethoxynaphthalen-1-yl)but-3-yn-2-one / (*Z*)-1-(4-chloropyridin -2-yl)-4-(2-ethoxynaphthalen-1-yl)but-1-en-3- yn-2-ol

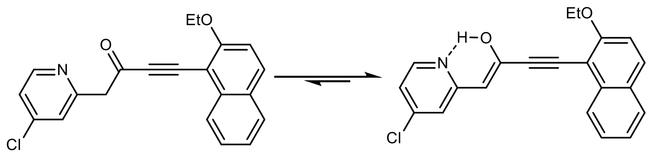

Yellow solid, m.p. 69-70 °C. **^{1}H NMR (500 MHz, Chloroform-*d*) δ**: 8.34 (d, *J* = 8.4 Hz, 1H), 8.29 (d, *J* = 5.6 Hz, 1H), 7.89 (d, *J* = 9.0 Hz, 1H), 7.83 (t, *J* = 7.2 Hz, 1H), 7.66 – 7.58 (m, 1H), 7.45 (ddd, *J* = 8.0, 6.8, 1.1 Hz, 1H), 7.29 (d, *J* = 9.2 Hz, 1H), 7.11 (d, *J* = 2.0 Hz, 1H), 7.08 (dd, *J* = 5.6, 2.0 Hz, 1H), 6.00 (s, 1H, *enol*), 4.39 – 4.34 (m, 2H), 1.58 (dt, *J* = 15.2, 6.9 Hz, 3H). (*enol: keto* = 2.3 :1). **^{13}C NMR (126 MHz, Chloroform-*d*) δ**: 160.50, 158.40, 158.20, 154.68, 149.53, 148.68, 145.14, 143.98, 143.59, 133.91, 133.50, 132.55, 130.17, 127.45, 127.28, 127.09, 126.60, 124.24, 123.93, 123.76, 123.62, 123.38, 121.68, 119.97, 118.67, 113.20, 112.43, 104.49, 102.58, 94.11, 85.16, 64.42, 64.21, 53.13, 14.08, 13.94. **HRMS(ESI) m/z**: calculated for $[C_{21}H_{16}ClNO_2 + H]^+$ 350.0948, found 350.0945.

1u, 1-(5-bromopyridin-2-yl)-4-(2-ethoxynaphthalen-1-yl)but-3-yn-2-one / (*Z*)-1-(5-bromopyridin -2-yl)-4-(2-ethoxynaphthalen-1-yl)but-1-en-3- yn-2-ol

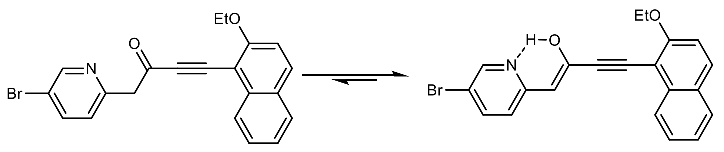

Yellow solid, m.p. 103-104 °C. **^{1}H NMR (500 MHz, Chloroform-*d*) δ**: 8.49 (d, *J* = 2.3 Hz, 1H), 8.34 (d, *J* = 8.4 Hz, 1H), 7.87 (dd, *J* = 8.7, 3.4 Hz, 1H), 7.83 (d, *J* = 7.9 Hz, 1H), 7.78 (dd, *J* = 8.5, 2.4 Hz, 1H), 7.65 – 7.58 (m, 1H), 7.44 (t, *J* = 7.5 Hz, 2H), 7.28 (d, *J* = 9.1 Hz, 1H), 7.00 (d, *J* = 8.5 Hz, 1H), 6.03 (s, 1H, *enol*), 4.35 (q, *J* = 7.0 Hz, 2H), 1.58 (t, *J* = 7.0 Hz, 3H). (*enol: keto* = 2.4 :1) **^{13}C NMR (126 MHz, Chloroform-*d*) δ**: 160.45, 158.33, 155.53, 149.79, 146.54, 146.00, 138.77, 138.19, 133.89, 133.47, 132.55, 130.11, 127.45, 127.28, 127.26, 127.10, 126.58, 124.89, 124.22, 123.73, 123.63, 123.37, 121.62, 118.52, 114.10, 113.20, 112.42, 104.53, 103.32, 94.00, 88.45, 85.25, 64.39, 64.18, 52.84, 14.08, 13.95. **HRMS(ESI) m/z**: calculated for $[C_{21}H_{16}BrNO_2 + H]^+$ 394.0443, found 394.0433.

1v, 4-(2-ethoxynaphthalen-1-yl)-1-(5-methylpyridin-2-yl)but-3-yn-2-one / (*Z*)-4-(2-ethoxynaphthalen-1-yl)-1-(5-methylpyridin-2-yl)but-1-en-3- yn-2-ol

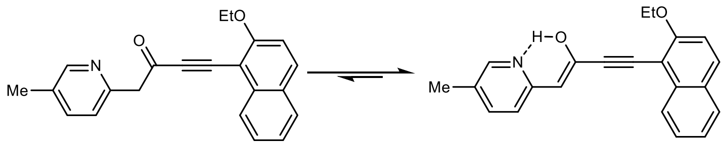

Yellow solid, m.p. 75-76 °C. **^{1}H NMR (500 MHz, Chloroform-*d*) δ**: 8.37 (dd, *J* = 8.4, 1.0 Hz, 1H), 8.21 (d, *J* = 2.0 Hz, 1H), 7.87 (d, *J* = 9.0 Hz, 1H), 7.85 – 7.77 (m, 1H), 7.61 (ddd, *J* = 8.3, 6.8, 1.3 Hz, 1H), 7.57 – 7.54 (m, 1H), 7.44 (ddd, *J* = 8.2, 6.8, 1.2 Hz, 1H), 7.29 (d, *J* = 9.1 Hz, 1H), 7.02 (d, *J* = 8.2 Hz, 1H), 6.03 (s, 1H, *enol*), 4.36 (q, *J* = 7.0 Hz, 2H), 2.38 (s, 3H), 1.59 (t, *J* = 7.0 Hz, 3H). (*enol: keto* = 2 :1) **^{13}C NMR (126 MHz, Chloroform-*d*) δ**: 158.12, 154.44, 149.14, 147.05, 143.59, 137.19, 136.21, 133.53, 132.29, 129.74, 127.94, 127.50, 127.19, 127.07, 127.01, 126.44, 124.41, 123.94, 123.55, 123.31, 123.03, 119.99, 113.41, 112.56, 103.08, 96.65, 94.72, 83.82, 64.48, 64.23, 53.20, 17.30, 17.18, 14.10, 13.94. **HRMS(ESI) m/z**: calculated for $[C_{22}H_{19}NO_2 + H]^+$ 330.1494, found 330.1492.

1w, 4-(2-ethoxynaphthalen-1-yl)-1-(pyrazin-2-yl)but-3-yn-2-one / (*Z*)-4-(2-ethoxynaphthalen-1-yl) -1-(pyrazin-2-yl)but-1-en-3-yn-2-ol

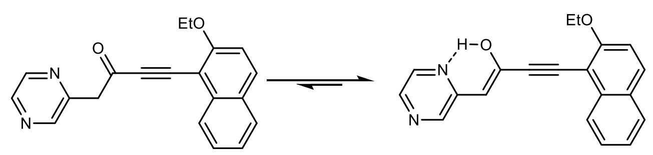

Yellow solid, m.p. 85-86 °C. **^{1}H NMR (500 MHz, Chloroform-*d*) δ**: 8.47 (d, *J* = 1.5 Hz, 1H), 8.36 (d, *J* = 2.8 Hz, 1H), 8.35 – 8.33 (m, 2H), 7.88 (d, *J* = 9.0 Hz, 1H), 7.82 (t, *J* = 8.3 Hz, 1H), 7.61 (dddd, *J* = 12.2, 8.4, 6.9, 1.3 Hz, 1H), 7.44 (dddd, *J* = 8.1, 6.9, 2.9, 1.2 Hz, 1H), 7.28 (d, *J* = 9.1 Hz, 1H), 6.12 (s, 1H, *enol*), 4.39 – 4.33 (m, 2H), 1.61 – 1.56 (m, 3H), (*enol: keto* = 1.1 :1) **^{13}C NMR (126 MHz, Chloroform-*d*) δ**: 160.55, 158.51, 152.54, 149.11, 148.20, 144.92, 143.45, 142.77, 142.28, 139.05, 138.98, 133.89, 133.44, 132.72, 130.41, 127.40, 127.34, 127.32, 127.15, 127.12, 126.70, 124.11, 123.67, 123.65, 123.42, 113.03, 112.27, 104.12, 101.55, 100.95, 96.43, 93.55, 88.99, 86.04, 64.34, 64.13, 50.82, 14.06, 13.92. **HRMS(ESI) m/z**: calculated for $[C_{22}H_{16}N_2O_2 + H]^+$ 317.1290, found 317.1282.

1x, 4-(2-ethoxynaphthalen-1-yl)-1-(5-(4-((((3a*S*,5a*R*,8a*R*,8b*S*)-2,2,7,7-tetramethyltetrahydro-3a*H*-bis([1,3]dioxolo)[4,5-b:4',5'-d]pyran-3a-yl)methoxy)methyl)phenyl)pyridin-2-yl)but-3-yn-2-one / (*Z*)-4-(2-ethoxynaphthalen-1-yl)-1-(5-(4-((((3a*S*,5a*R*,8a*R*,8b*S*)-2,2,7,7-tetramethyltetrahydro-3a*H*-bis([1,3]dioxolo)[4,5-b:4',5'-d]pyran-3a-yl)methoxy)methyl)phenyl)pyridin-2-yl)but-1-en-3-yn-2-ol

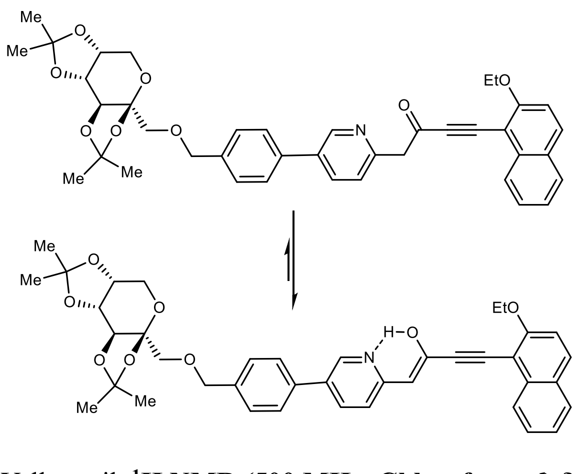

Yellow oil. **^{1}H NMR (500 MHz, Chloroform-*d*) δ**: 8.64 (d, *J* = 2.2 Hz, 1H), 8.38 (dd, *J* = 8.6, 1.0 Hz, 1H), 7.91 (dd, *J* = 8.4, 2.3 Hz, 1H), 7.88 (d, *J* = 9.0 Hz, 1H), 7.84 (d, *J* = 8.1 Hz, 1H), 7.63 – 7.60 (m, 4H), 7.53 – 7.50 (m, 4H), 7.46 – 7.45 (m, 1H), 7.31 (s, 1H), 7.29 (d, *J* = 9.0 Hz, 1H), 7.18 (dd, *J* = 8.4, 0.7 Hz, 1H), 6.11 (s, 1H, *enol*), 4.78 (s, 1H), 4.71 (d, *J* = 1.9 Hz, 1H), 4.68 – 4.66 (m, 2H), 4.51 (t, *J* = 2.6 Hz, 1H), 4.39 – 4.36 (m, 3H), 3.99 (dd, *J* = 13.0, 2.0 Hz, 1H), 3.81

(dd, *J* = 13.0, 0.8 Hz, 1H), 3.74 – 3.68 (m, 3H), 1.62 (s, 3H), 1.61 (s, 3H), 1.50 (s, 3H), 1.48 (s, 3H), 1.40 (s, 3H). (*enol: keto* = 2.8 :1) **^{13}C NMR (126 MHz, Chloroform-*d*) δ**: 158.24, 155.67, 147.68, 142.18, 137.09, 135.44, 134.49, 133.94, 133.52, 132.40, 131.12, 129.93, 127.48, 127.32, 127.23, 127.06, 126.52, 126.01, 125.54, 124.36, 123.85, 123.58, 123.35, 120.38, 113.30, 107.92, 107.59, 103.16, 101.68, 94.60, 84.58, 72.25, 70.68, 70.00, 69.20, 69.16, 64.45, 64.22, 60.02, 53.27, 25.60, 24.85, 24.46, 23.04, 14.11, 13.97. **HRMS(ESI) m/z**: calculated for $[C_{40}H_{41}NO_8 + H]^+$ 664.2910 , found 664.2906.

1y, 4-(2-ethoxynaphthalen-1-yl)-1-(5-(4-((((*R*)-2,5,7,8-tetramethyl-2-((4*S*,8*R*)-4,8,12-trimethyltridecyl)chroman-6-yl)oxy)methyl)phenyl)pyridin-2-yl)but-3-yn-2-one / (*Z*)-4-(2-ethoxynaphthalen-1-yl)-1-(5-(4-((((*R*)-2,5,7,8-tetramethyl-2-((4*S*,8*R*)-4,8,12-trimethyltridecyl)chroman-6-yl)oxy)methyl)phenyl)pyridin-2-yl)but-1-en-3-yn-2-ol

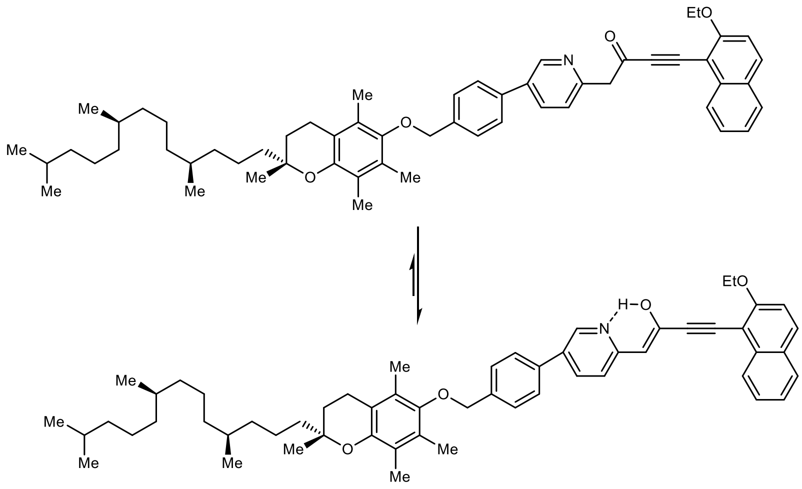

Yellow oil. **^{1}H NMR (500 MHz, Chloroform-*d*) δ**: 8.66 (d, *J* = 2.3 Hz, 1H), 8.44 (d, *J* = 2.8 Hz, 1H), 7.95 – 7.90 (m, 1H), 7.88 (d, *J* = 9.0 Hz, 1H), 7.85 (d, *J* = 8.1 Hz, 1H), 7.72 – 7.63 (m, 5H), 7.47 (t, *J* = 7.5 Hz, 1H), 7.29 (d, *J* = 9.1 Hz, 1H), 7.17 (d, *J* = 8.4 Hz, 1H), 6.14 (s, 1H, *enol*), 4.83 (d, *J* = 2.5 Hz, 2H), 4.37 (q, *J* = 7.0 Hz, 2H), 2.69 (t, *J* = 6.8 Hz, 2H), 2.34 (s, 3H), 2.29 (s, 3H), 2.23 (s, 3H), 1.99 – 1.82 (m, 2H), 1.63 (dt, *J* = 13.7, 6.8 Hz, 4H), 1.57 (d, *J* = 6.9 Hz, 3H), 1.54 – 1.46 (m, 2H), 1.37 (d, *J* = 20.0 Hz, 12H), 1.25 (ddt, *J* = 9.3, 6.6, 4.0 Hz, 2H), 1.22 – 1.11 (m, 4H), 1.06 – 0.93 (m, 11H), 0.90 (d, *J* = 6.9 Hz, 1H). (*enol: keto* = 4 :1) **^{13}C NMR (126 MHz, Chloroform-*d*) δ**: 158.30, 155.71, 147.76, 147.13, 147.04, 142.20, 137.02, 135.64, 134.52, 134.01, 133.95, 133.56, 132.46, 131.10, 129.99, 127.52, 127.42, 127.37, 127.29, 127.22, 127.13, 126.90, 126.57, 126.19, 125.69, 124.94, 124.37, 123.85, 123.56, 123.38, 122.02, 120.45, 116.69,

113.27, 112.48, 104.79, 103.25, 96.79, 94.78, 84.71, 76.40, 76.15, 75.90, 73.88, 73.23, 70.51, 64.41, 64.21, 53.31, 49.16, 44.13, 39.09, 38.44, 36.53, 36.49, 36.36, 33.61, 31.86, 31.76, 30.68, 30.38, 27.04, 24.88, 23.88, 23.52, 22.94, 22.21, 21.81, 21.72, 21.29, 20.09, 19.76, 18.84, 18.75, 15.18, 14.15, 14.01, 11.97, 11.10, 10.93. **HRMS(ESI) m/z**: calculated for $[C_{57}H_{71}NO_4 + H]^+$ 834.5641 , found 834.5633.

1z, 4-(2-ethoxynaphthalen-1-yl)-1-(5-(4-((((1*R*,2*S*,5*R*)-2-isopropyl-5-methylcyclohexyl)oxy) methyl)phenyl)pyridin-2-yl)but-3-yn-2-one / (*Z*)-4-(2-ethoxynaphthalen-1-yl)-1-(5-(4-((((1*R*,2*S*, 5*R*)-2-isopropyl-5-methylcyclohexyl)oxy) -methyl)phenyl)pyridin-2-yl)but-1-en-3-yn-2-ol

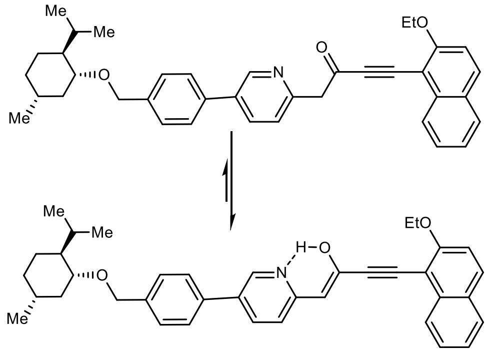

Yellow oil. **^{1}H NMR (500 MHz, Chloroform-*d*) δ**: 8.63 (d, *J* = 2.2 Hz, 1H), 8.40 – 8.38 (m, 1H), 7.90 (dd, *J* = 8.4, 2.3 Hz, 1H), 7.87 (d, *J* = 9.2 Hz, 1H), 7.82 (dd, *J* = 9.1, 7.8 Hz, 2H), 7.61 – 7.59 (m, 3H), 7.52 – 7.51 (m, 3H), 7.45 (ddd, *J* = 8.0, 6.7, 1.2 Hz, 1H), 7.28 (d, *J* = 9.1 Hz, 1H), 7.16 (d, *J* = 8.3 Hz, 1H), 6.11 (s, 1H, *enol*), 4.77 (d, *J* = 11.6 Hz, 1H), 4.50 (d, *J* = 11.6 Hz, 1H), 4.36 (q, *J* = 7.0 Hz, 2H), 3.27 (td, *J* = 10.6, 4.0 Hz, 2H), 2.65 (s, 1H), 2.39 (dddt, *J* = 13.9, 6.8, 4.9, 2.5 Hz, 2H), 2.27 (dt, *J* = 12.0, 2.0 Hz, 2H), 1.71 (ddd, *J* = 13.5, 10.8, 3.1 Hz, 4H), 1.60 (t, *J* = 7.0 Hz, 3H), 1.41 – 1.37 (m, 1H), 1.02 – 0.97 (m, 10H), 0.81 (dd, *J* = 7.0, 4.1 Hz, 6H). (*enol: keto* = 3.8 :1) **^{13}C NMR (126 MHz, Chloroform-*d*) δ**: 158.24, 156.10, 155.61, 151.80, 147.65, 147.10, 146.44, 142.16, 138.18, 137.88, 135.98, 135.60, 135.29, 134.50, 133.95, 133.64, 133.52, 132.54, 132.42, 131.23, 129.94, 127.57, 127.51, 127.47, 127.44, 127.23, 127.19, 127.07, 126.53, 126.02, 125.88, 125.55, 124.36, 123.84, 123.58, 123.48, 123.35, 122.16, 120.38, 113.26, 112.46, 104.78, 103.19, 94.67, 84.58, 78.01, 77.96, 69.03, 68.98, 64.41, 53.28, 47.34, 39.33, 33.57, 30.59, 24.60, 24.58, 23.11, 22.28, 21.42, 20.06, 15.16, 15.13, 14.11, 13.97. **HRMS(ESI) m/z**: calculated for $[C_{38}H_{41}NO_3 + H]^+$ 560.3165 , found 560.3154.

General procedure F: Initial studies for the synthesis of 2a

To a vial was added catalyst (10 mol%), ligand (15 mol%), solvent (1.0 mL) and stirring bar. The vial was wrapped with Teflon tape and fitted with corresponding cap. The vial was stirred at room temperature for 30 min. Then the substrate **1a** (0.1 mmol, 1.0 eq) was added. The vial was stirred at 20 °C for a certain time. After the completion of the reaction detected by TLC, the mixture was diluted with DCM and washed with water for three times. The extracts were dried and concentrated in vacuo. The crude product was purified by column chromatograph over silica gel. The ee values were determined by chiral HPLC.

Optimization of reaction conditions

Supplementary Table 1. Optimization of the catalyst. [a]

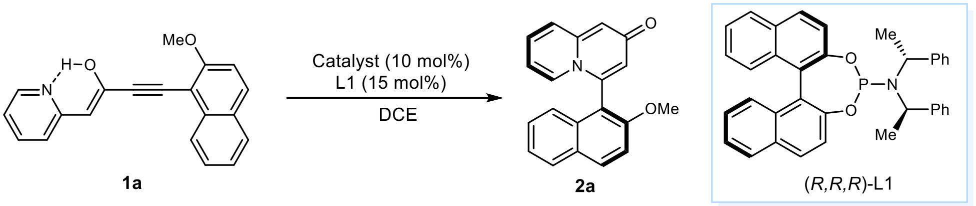

Entry	Catalyst	ee
1	Cu_2O	63%
2	$[CuOTf]_2C_6H_6$	33%
3	CuOAc	60%
4	CuF	64%
5	CuCl	73%
6	CuBr	65%
7	CuI	54%
8	$Cu(MeCN)_4PF_6$	61%
9	$CuBF_4$	36%
10	(*R*)-TRIP-Cu	5%
11	CuF_2	62%
12	$Cu(OAc)_2$	60%
13	$Cu(OTf)_2$	46%
14	$CuSO_4$	69%
15	AgOTf	29%
16	$Pd(PPh_3)_2Cl_2$	N.D.

[a]Reaction conditions: **1a** (0.1 mmol, 1.0 eq), catalyst (10 mol%), L1 (15 mol%), DCE (1.0 mL), 20 °C for 3 d. The ee values were determined by chiral HPLC.

Supplementary Table 2. Effect of the solvent for the reaction [a]

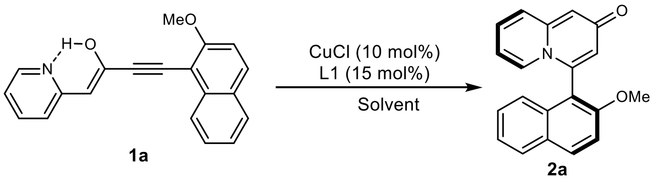

Entry	Solvent	ee
1	Toluene	76%
2	DCM	72%
3	DCE	73%
4	$CHCl_3$	70%
5	MeCN	56%
6	Et_2O	64%
7	THF	69%
8	MeOH	69%
9	Dioxane	66%
10	MTBE	60%
11	Benzene	73%
12	DMF	N.D.

[a] Reaction conditions: **1a** (0.1 mmol, 1.0 eq), CuCl (10 mol%), L1 (15 mol%), solvent (1.0 mL), 20 °C for 3 d. The ee values were determined by chiral HPLC.

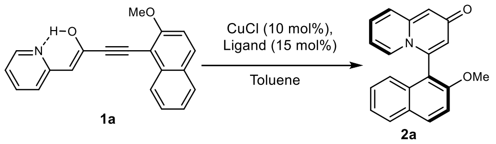

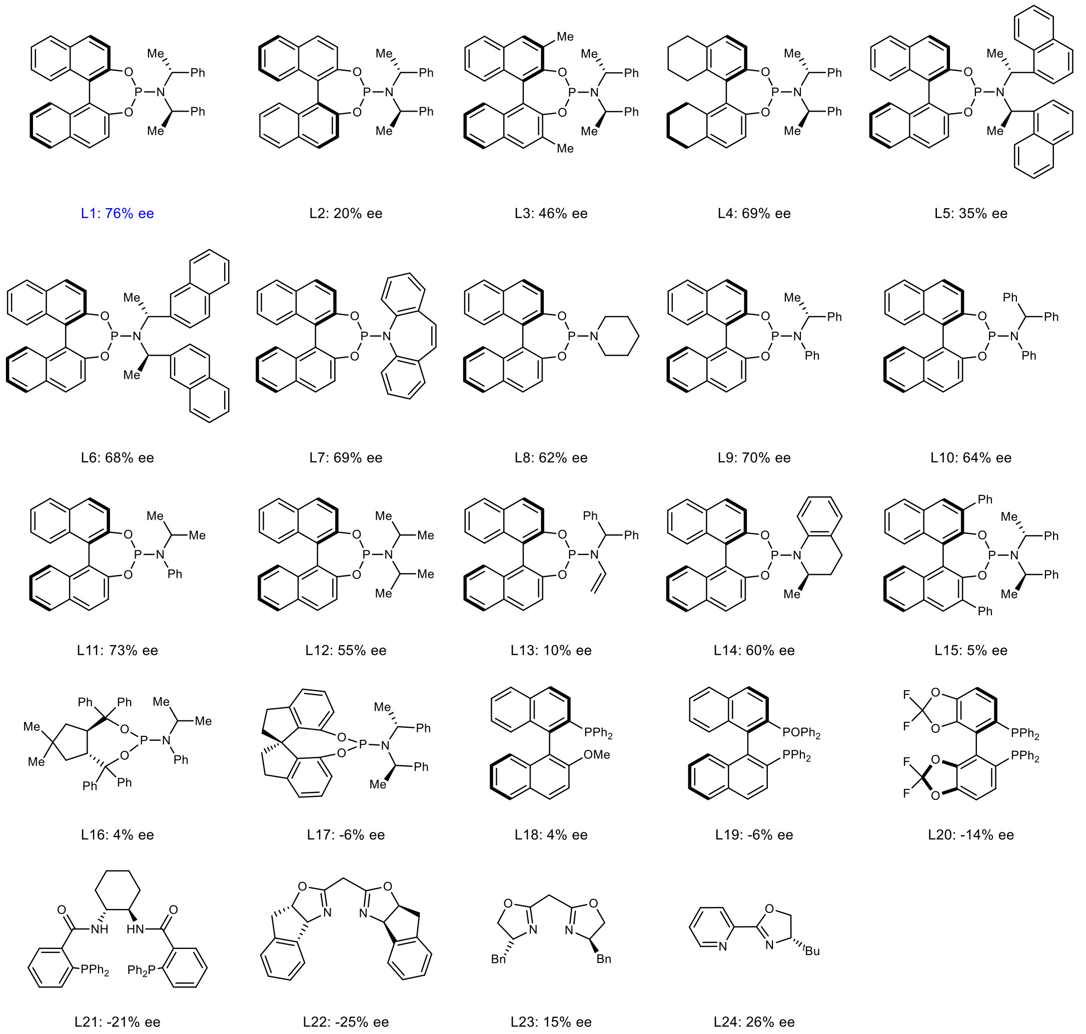

Supplementary Fig. 1. Effect of the Ligand for the reaction. Reaction conditions: **1a** (0.1 mmol, 1.0 eq), CuCl (10 mol%), Ligand (15 mol%), toluene (1.0 mL), 20 °C for 3 d. The ee values were determined by chiral HPLC.

General procedure G: Comparison of $(PhO)_2POOH$ and CPA for the reaction

To a vial was added of CuCl (1.0 mg, 10 mol%), L1 (8.5 mg, 15 mol%), toluene (2.0 mL) and stirring bar. The vial was wrapped with Teflon tape and fitted with corresponding cap. The vial was stirred at room temperature for 30 min. Then corresponding substrate (0.1 mmol, 1.0 eq) and phosphoric acid (0.1 mmol, 1.0 eq) were added into the mixture. The reaction was stirred at 20 °C for 36 h. Then, the mixture was diluted with EA and washed with water for three times. The

extracts were dried over anhydrous Na_2SO_4 and concentrated under the vacuum. The product was purified by column chromatograph over silica gel. The ee values were determined by chiral HPLC.

Supplementary Table 3. Comparison of $(PhO)_2POOH$ and CPA for the reaction [a]

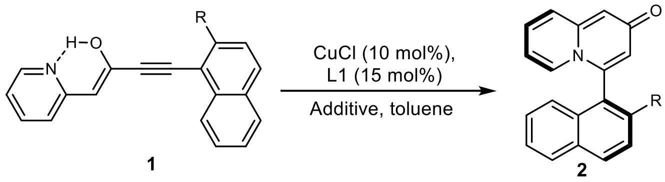

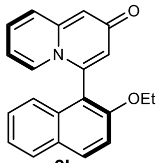

2b

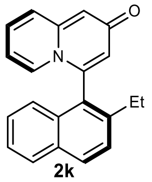

2k

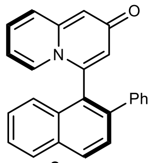

2n

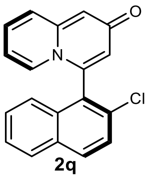

2q

	ee	yield	ee	yield	ee	yield	ee	yield
$(PhO)_2POOH$	80%	85%	86%	90%	94%	95%	63%	70%
(*rac*)-CPA	91%	89%	88%	97%	92%	91%	62%	72%
(*R*)-CPA	92%	88%	88%	94%	93%	93%	62%	73%

[a] Reaction conditions: **1** (0.1 mmol, 1.0 eq), CuCl (10 mol%), L1 (15 mol%), additive (0.1 mmol, 1.0 eq), toluene (2.0 mL), 20 °C for 3 d. The ee values were determined by chiral HPLC.

General procedure H: Synthesis of the enantioenriched products 2

To a vial was added of CuCl (1.0 mg, 10 mol%), L1 (8.5 mg, 15 mol%), toluene (2.0 mL) and stirring bar. The vial was wrapped with Teflon tape and fitted with corresponding cap. The vial was stirred at room temperature for 30 min. Then the corresponding substrate (0.1 mmol, 1.0 eq) and chiral phosphoric acid (34.8 mg, 0.1 mmol, 1.0 eq) were added. The reaction was stirred at 20 °C for 36 h. Then, the mixture was diluted with EA and washed with water for three times. The extracts were dried and concentrated in vacuo. The crude product was purified by column chromatograph over silica gel. The ee values were determined by chiral HPLC.

2a, 4-(2-methoxynaphthalen-1-yl)-2*H*-quinolizin-2-one

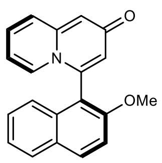

Following the general procedure **H**, **2a** was afforded as yellow solid (24.7 mg, 82 % yield), m.p. 91-92 °C. $[\alpha]_D^{25}$ +70.0 (*c* = 0.5, $CHCl_3$). **^{1}H NMR (500 MHz, Chloroform-*d*) δ**: 8.12 (d, *J* = 9.1 Hz, 1H), 7.93 (dd, *J* = 6.1, 3.4 Hz, 1H), 7.46 – 7.43 (m, 3H), 7.36 – 7.30 (m, 3H), 7.13 (dd, *J* = 9.0, 6.4 Hz, 1H), 6.84 (d, *J* = 4.9 Hz, 2H), 6.39 (t, *J* = 6.9 Hz, 1H), 3.90 (s, 3H). **^{13}C NMR (126 MHz, Chloroform-*d*) δ:** 175.95, 154.90, 145.16, 141.46, 133.01, 132.25, 130.31, 128.90, 128.52, 128.41, 126.26, 125.71, 124.67, 123.69, 123.12, 115.90, 113.67, 112.70, 110.80, 56.47. **HRMS(ESI) m/z**: calculated for $[C_{20}H_{15}NO_2 + H]^+$ 302.1181, found 302.1176. **HPLC data** (Chiralpak AD column, hexane : isopropanol = 70 : 30, 1.0 mL/min): tr = 19.2 min (major), tr = 31.1 min (minor), ee = 76%.

2b, 4-(2-ethoxynaphthalen-1-yl)-2*H*-quinolizin-2-one

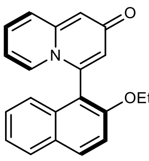

Following the general procedure **H**, **2b** was afforded as yellow solid (27.9 mg, 88% yield), m.p. 97-98 °C. $[\alpha]_D^{25}$ +110.0 (*c* = 0.5, $CHCl_3$). **^{1}H NMR (500 MHz, Chloroform-*d*) δ**: 8.08 (d, *J* = 9.1 Hz, 1H), 7.91 (dd, *J* = 6.5, 3.0 Hz, 1H), 7.45 – 7.40 (m, 1H), 7.43 (d, *J* = 4.0 Hz, 1H), 7.40 (s, 1H), 7.37 (dd, *J* = 8.8, 5.4 Hz, 2H), 7.34 – 7.30 (m, 1H), 7.13 (dd, *J* = 9.0, 6.4 Hz, 1H), 6.89 (s, 1H), 6.82 (s, 1H), 6.40 (t, *J* = 6.9 Hz, 1H), 4.19 (qd, *J* = 7.0, 2.1 Hz, 2H), 1.23 (t, *J* = 7.0 Hz, 3H). **^{13}C NMR (126 MHz, Chloroform-*d*) δ**: 174.85, 153.15, 144.02, 140.28, 131.44, 131.40, 128.43, 127.85, 127.48, 127.33, 127.00, 125.31, 123.46, 123.38, 122.37, 113.77, 112.85, 110.80, 110.38, 63.80, 13.76. **HRMS(ESI) m/z**: calculated for $[C_{21}H_{17}NO_2 + H]^+$ 316.1138, found 316.1128. **HPLC data** (Chiralpak OD column, hexane : isopropanol = 70 : 30, 1.0 mL/min): tr = 8.1 min (major), tr =15.6 min (minor), ee = 92%.

2c, 4-(6-bromo-2-ethoxynaphthalen-1-yl)-2*H*-quinolizin-2-one

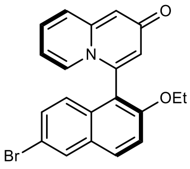

Following the general procedure **H**, **2c** was afforded as yellow solid (30.9 mg, 79 % yield), m.p. 52-53 °C. $[\alpha]_D^{25}$ -255.2 (*c* = 0.5, $CHCl_3$). **^{1}H NMR (500 MHz, Chloroform-*d*) δ**: 8.08 (d, *J* = 2.0

Hz, 1H), 8.00 (d, J = 9.1 Hz, 1H), 7.51 (dd, J = 9.0, 2.0 Hz, 1H), 7.44 (d, J = 9.3 Hz, 1H), 7.31 (s, 1H), 7.31 – 7.27 (m, 2H), 7.13 (dd, J = 9.1, 6.4 Hz, 1H), 6.82 (d, J = 15.8 Hz, 2H), 6.40 (t, J = 6.7 Hz, 1H), 4.20 (tt, J = 7.1, 3.5 Hz, 2H), 1.24 (t, J = 7.0 Hz, 3H). **^{13}C NMR (126 MHz, Chloroform-*d*) δ:** 175.62, 154.51, 145.19, 140.89, 132.57, 131.62, 131.36, 131.05, 130.33, 129.96, 129.55, 129.32, 128.74, 125.21, 124.63, 123.38, 118.29, 114.94, 112.29, 65.01, 14.74. **HRMS(ESI) m/z**: calculated for $[C_{21}H_{16}BrNO_2 + H]^+$ 394.0443, found 394.0442. **HPLC data** (Chiralpak OD column, hexane : isopropanol = 70 : 30, 1.0 mL/min): tr = 7.9 min (major), tr =11.7 min (minor), ee = 90%.

2d, 4-(7-bromo-2-ethoxynaphthalen-1-yl)-2*H*-quinolizin-2-one

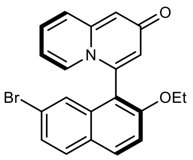

Following the general procedure **H**, **2d** was afforded as yellow solid (37.3 mg, 95 % yield), m.p. 66-67 °C. $[\alpha]_D^{25}$ -148.4 (c = 0.25, CHCl$_3$). **^{1}H NMR (500 MHz, Chloroform-*d*) δ**: 8.03 (d, J = 9.0 Hz, 1H), 7.75 (d, J = 8.6 Hz, 1H), 7.52 (s, 1H), 7.47 (d, J = 8.6 Hz, 1H), 7.40 (d, J = 9.0 Hz, 1H), 7.32 – 7.25 (m, 2H), 7.12 (s, 1H), 6.83 (s, 2H), 6.42 (t, J = 6.7 Hz, 1H), 4.16 (q, J = 7.2 Hz, 2H), 1.18 (t, J = 6.9 Hz, 3H). **^{13}C NMR (126 MHz, Chloroform-*d*) δ**: 174.32, 153.97, 144.17, 139.97, 132.68, 131.61, 131.37, 129.03, 128.24, 127.83, 127.00, 126.26, 126.02, 124.32, 123.72, 121.83, 112.99, 112.89, 111.47, 63.90, 13.68. **HRMS(ESI) m/z**: calculated for $[C_{21}H_{16}BrNO_2 + H]^+$ 394.0443, found 394.0440. **HPLC data** (Chiralpak OD column, hexane : isopropanol = 70 : 30, 1.0 mL/min): tr = 6.9 min (major), tr =10.7 min (minor), ee = 91%.

2e, 4-(2-ethoxy-7-phenylnaphthalen-1-yl)-2*H*-quinolizin-2-one

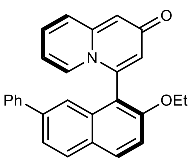

Following the general procedure **H**, **2e** was afforded as yellow solid (29.5 mg, 75 % yield), m.p. 66-67 °C. $[\alpha]_D^{25}$ -482.5 (c = 0.25, CHCl$_3$). **^{1}H NMR (500 MHz, Chloroform-*d*) δ**: 8.11 (d, J = 9.1 Hz, 1H), 7.99 (d, J = 8.5 Hz, 1H), 7.70 (dd, J = 8.5, 1.6 Hz, 1H), 7.56 (d, J = 7.7 Hz, 3H), 7.43 (q, J = 9.2, 8.3 Hz, 3H), 7.38 (t, J = 7.3 Hz, 2H), 7.34 – 7.29 (m, 1H), 7.12 (dd, J = 9.0, 6.4 Hz, 1H), 6.97 (s, 1H), 6.82 (s, 1H), 6.39 (t, J = 6.9 Hz, 1H), 4.23 – 4.19 (m, 2H), 1.24 (t, J = 7.0 Hz, 3H).

^{13}C NMR (126 MHz, Chloroform-*d*) δ: 175.88, 154.70, 145.29, 141.76, 141.08, 141.05, 140.56, 132.84, 132.37, 129.51, 129.04, 128.88, 128.79, 128.14, 127.77, 127.53, 124.65, 124.47, 124.45, 121.06, 115.04, 113.83,112.31, 64.92, 14.80. **HRMS(ESI) m/z**: calculated for $[C_{27}H_{21}NO_2 + H]^+$ 392.1651, found 392.1644. **HPLC data** (Chiralpak OD column, hexane : isopropanol = 70 : 30, 1.0 mL/min): tr = 8.4 min (major), tr =10.2 min (minor), ee = 84%.

2f, 4-(2-ethoxy-7-(phenylethynyl)naphthalen-1-yl)-2*H*-quinolizin-2-one

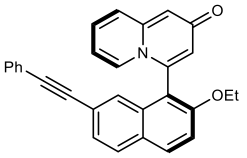

Following the general procedure **H**, **2f** was afforded as yellow solid (33.1 mg, 80 % yield), m.p. 70-71 °C. $[\alpha]_D^{25}$ -562.3 (*c* = 0.5, $CHCl_3$). **^{1}H NMR (500 MHz, Chloroform-*d*) δ**: 8.05 (d, *J* = 9.0 Hz, 1H), 7.87 (d, *J* = 8.4 Hz, 1H), 7.60 (s, 1H), 7.59 – 7.52 (m, 3H), 7.39 (d, *J* = 9.1 Hz, 1H), 7.35 – 7.31 (m, 4H), 7.29 (d, *J* = 9.4 Hz, 1H), 7.11 (t, *J* = 7.8 Hz, 1H), 6.87 (d, *J* = 7.3 Hz, 2H), 6.40 (t, *J* = 6.9 Hz, 1H), 4.17 (q, *J* = 7.0 Hz, 2H), 1.21 (t, *J* = 6.9 Hz, 3H). **^{13}C NMR (126 MHz, Chloroform-*d*) δ:** 175.63, 154.76, 145.23, 141.17, 132.42, 132.25, 131.89, 131.67, 129.43, 129.01, 128.86, 128.57, 128.54,128.44, 128.33, 128.18, 127.12, 126.50, 124.54, 122.94, 122.74, 114.38, 114.32, 112.36, 91.09, 89.40, 64.85, 14.71. **HRMS(ESI) m/z**: calculated for $[C_{29}H_{21}NO_2 + H]^+$ 416.1651, found 416.1648. **HPLC data** (Chiralpak AD column, hexane : isopropanol = 65:35, 1.0 mL/min): tr = 9.2 min (major), tr = 12.4 min (minor), ee = 95%.

2g, 4-(2-((4-methoxybenzyl)oxy)naphthalen-1-yl)-2*H*-quinolizin-2-one

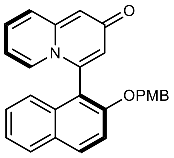

Following the general procedure **H**, **2g** was afforded as yellow solid (32.0 mg, 78 % yield), m.p. 63-64 °C. $[\alpha]_D^{25}$ -183.5 (*c* = 0.5, $CHCl_3$). **^{1}H NMR (500 MHz, Chloroform-*d*) δ**: 8.06 (d, *J* = 9.1 Hz, 1H), 7.91 (dd, *J* = 6.2, 3.3 Hz, 1H), 7.48 – 7.43 (m, 3H), 7.38 – 7.36 (m, 1H), 7.32 – 7.29 (m, 2H), 7.11 (d, *J* = 8.3 Hz, 3H), 6.86 (s, 2H), 6.82 (d, *J* = 8.1 Hz, 2H), 6.35 (t, *J* = 6.9 Hz, 1H), 5.16 (s, 2H), 3.80 (s, 3H). **^{13}C NMR (126 MHz, Chloroform-*d*) δ**: 175.80, 159.46, 153.98, 145.13, 141.35, 132.44, 132.44, 129.50, 129.15, 128.67, 128.56, 128.41, 128.17, 126.26, 124.78, 124.48, 123.48, 115.60, 114.80, 114.01, 112.05, 111.59, 111.49, 70.92, 55.26. **HRMS(ESI) m/z**:

calculated for $[C_{27}H_{21}NO_3 + H]^+$ 408.1600, found 408.1590. **HPLC data** (Chiralpak OD column, hexane : isopropanol = 70 : 30, 1.0 mL/min): tr = 13.4 min (major), tr =23.1 min (minor), ee = 91%.

2h, 4-(2-(dimethylamino)naphthalen-1-yl)-2*H*-quinolizin-2-one

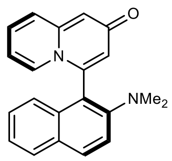

Following the general procedure **H**, **2h** was afforded as yellow solid (28.0 mg, 89 % yield), m.p. 204-205 °C. $[\alpha]_D^{25}$ +48.0 (c = 1.0, $CHCl_3$). **^{1}H NMR (500 MHz, Chloroform-*d*) δ**: 8.02 (d, J = 9.0 Hz, 1H), 7.88 (dd, J = 7.3, 2.0 Hz, 1H), 7.48 (d, J = 9.0 Hz, 1H), 7.42 (ddd, J = 8.0, 6.1, 1.5 Hz, 2H), 7.38 (d, J = 7.6 Hz, 1H), 7.33 – 7.31 (m, 2H), 7.15 (dd, J = 9.1, 6.5 Hz, 1H), 6.98 (s, 1H), 6.84 (s, 1H), 6.43 – 6.40 (m, 1H), 2.70 (s, 6H). **^{13}C NMR (126 MHz, Chloroform-*d*) δ**: 167.66, 150.92, 149.50, 145.37, 132.93, 132.50, 132.07, 131.07, 130.18, 130.00, 129.71, 128.80, 128.23, 127.03, 125.91, 125.04, 124.66, 122.29, 119.45, 116.76, 43.66. **HRMS(ESI) m/z**: calculated for $[C_{21}H_{18}N_2O + H]^+$ 315.1497, found 315.1488. **HPLC data** (Chiralpak OD column, hexane : isopropanol = 70 : 30, 1.0 mL/min): tr = 7.7 min (major), tr =10.9 min (minor), ee = 92%.

2i, 4-(2-(dibenzylamino)naphthalen-1-yl)-2*H*-quinolizin-2-one

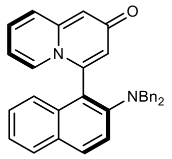

Following the general procedure **H**, **2i** was afforded as yellow solid (38.6 mg, 83 % yield), m.p. 75-76 °C. $[\alpha]_D^{25}$ -131.4 (c = 0.5, $CHCl_3$). **^{1}H NMR (500 MHz, Chloroform-*d*) δ**: 8.03 (d, J = 8.9 Hz, 1H), 7.93 (d, J = 8.1 Hz, 1H), 7.51 (dd, J = 8.0, 6.2 Hz, 2H), 7.44 (td, J = 7.5, 6.7, 1.3 Hz, 1H), 7.37 (d, J = 8.5 Hz, 1H), 7.32 (t, J = 4.6 Hz, 1H), 7.22 – 7.21(m, 6H), 7.06 (dd, J = 9.1, 6.4 Hz, 1H), 7.01 (d, J = 7.4 Hz, 1H), 6.96 (dd, J = 6.5, 2.9 Hz, 4H), 6.89 (d, J = 2.7 Hz, 1H), 6.82 (d, J = 2.7 Hz, 1H), 6.15 – 6.12 (m, 1H), 4.11 (s, 4H). **^{13}C NMR (126 MHz, Chloroform-*d*) δ**: 174.35, 147.66, 143.99, 141.83, 136.14, 131.39, 130.29, 129.89, 128.23, 127.66, 127.45, 127.30, 127.28, 126.83, 126.32, 125.85, 124.80, 123.42, 123.05, 121.91, 111.16, 110.58, 56.37. **HRMS(ESI) m/z**: calculated for $[C_{33}H_{26}N_2O + H]^+$ 467.2123, found 467.2116. **HPLC data** (Chiralpak AD column,

hexane : isopropanol = 70 : 30, 1.0 mL/min): tr = 10.5 min (major), tr =17.1 min (minor), ee = 98%.

2j, 4-(2-methylnaphthalen-1-yl)-2*H*-quinolizin-2-one

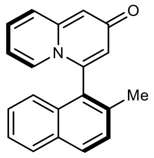

Following the general procedure **H**, **2j** was afforded as yellow solid (22.1 mg, 77 % yield), m.p. 45-46 °C. $[\alpha]_D^{25}$ -133.2 (c = 0.3, $CHCl_3$). **^{1}H NMR (500 MHz, Chloroform-*d*) δ**: 8.01 (d, J = 8.4 Hz, 1H), 7.95 (d, J = 8.2 Hz, 1H), 7.54 (dt, J = 7.7, 3.3 Hz, 2H), 7.45 (t, J = 7.8 Hz, 1H), 7.34 (d, J = 9.2 Hz, 1H), 7.29 (d, J = 8.4 Hz, 1H), 7.18 – 7.09 (m, 2H), 6.90 – 6.89 (m, 2H), 6.39 (t, J = 6.9 Hz, 1H), 2.29 (s, 3H). **^{13}C NMR (126 MHz, Chloroform-*d*) δ:** 175.75, 145.04, 143.39, 135.59, 132.16, 131.47， 130.47, 128.86, 128.64, 128.61, 128.43, 127.80, 127.76, 126.13, 125.67, 124.80, 124.04, 112.59, 111.81, 19.89. **HRMS(ESI) m/z**: calculated for $[C_{20}H_{15}NO + H]^+$ 286.1232, found 286.1227. **HPLC data** (Chiralpak IG column, hexane : isopropanol = 50: 50, 1.0 mL/min): tr = 21.3 min (major), tr = 15.1 min (minor), ee = 85%.

2k, 4-(2-ethylnaphthalen-1-yl)-2*H*-quinolizin-2-one

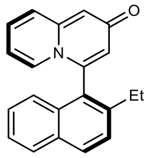

Following the general procedure **H**, **2k** was afforded as yellow solid (28.1 mg, 94 % yield), m.p. 66-67 °C. $[\alpha]_D^{25}$ +210.4 (c = 0.5, $CHCl_3$). **^{1}H NMR (500 MHz, Chloroform-*d*) δ**: 8.04 (d, J = 8.5 Hz, 1H), 7.95 (d, J = 8.2 Hz, 1H), 7.58 (d, J = 8.5 Hz, 1H), 7.53 (t, J = 7.5 Hz, 1H), 7.44 (t, J = 7.7 Hz, 1H), 7.27 (t, J = 9.7 Hz, 2H), 7.14 (d, J = 7.4 Hz, 1H), 7.08 (t, J = 7.9 Hz, 1H), 6.90 (s, 1H), 6.81 (s, 1H), 6.34 – 6.31 (m, 1H), 2.66 – 2.54 (m, 1H), 2.52 – 2.47 (m, 1H), 1.20 (t, J = 7.6 Hz, 3H). **^{13}C NMR (126 MHz, Chloroform-*d*) δ**: 174.68, 143.91, 142.03, 140.49, 139.05, 131.11, 130.32, 129.75, 128.13, 127.48, 127.37, 126.70, 126.10, 126.07, 125.16, 123.70, 123.22, 111.25, 110.93, 25.81, 14.37. **HRMS(ESI) m/z**: calculated for $[C_{21}H_{17}NO + H]^+$ 300.1388, found 300.1386. **HPLC data** (Chiralpak IG column, hexane : isopropanol = 50 : 50, 1.0 mL/min): tr = 16.3 min (major), tr = 24.9 min (minor), ee = 88%.

2l, 4-(2-butylnaphthalen-1-yl)-2*H*-quinolizin-2-one

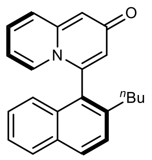

Following the general procedure **H**, **2l** was afforded as yellow solid (26.8 mg, 82 % yield), m.p. 34-35 °C. $[\alpha]_D^{25}$ -116.8 (c = 0.5, $CHCl_3$). **^{1}H NMR (500 MHz, Chloroform-*d*) δ**: 8.01 (d, J = 8.5 Hz, 1H), 7.92 (d, J = 8.1 Hz, 1H), 7.55 (d, J = 8.5 Hz, 1H), 7.50 (t, J = 7.5 Hz, 1H), 7.41 (t, J = 7.7 Hz, 1H), 7.28 (d, J = 9.0 Hz, 1H), 7.24 (d, J = 8.4 Hz, 1H), 7.11 (d, J = 7.4 Hz, 1H), 7.09 – 7.07 (m, 1H), 6.88 – 6.87 (m, 1H), 6.78 (s, 1H), 6.31 (t, J = 6.9 Hz, 1H), 2.64 – 2.58 (m, 1H), 2.45 – 2.39 (m, 1H), 1.61 – 1.49 (m, 2H), 1.29 – 1.23 (m, 2H), 0.82 (t, J = 7.3 Hz, 3H). **^{13}C NMR (126 MHz, Chloroform-*d*) δ**: 174.66, 143.89, 142.07, 139.29, 131.08, 131.00, 130.35, 129.56, 129.52, 128.15, 127.52, 127.36, 126.66, 126.57, 126.32, 125.14, 123.64, 123.20, 111.23, 32.36, 32.02, 21.58, 12.76. **HRMS(ESI) m/z**: calculated for $[C_{23}H_{21}NO + H]^+$ 328.1701, found 328.1698. **HPLC data** (Chiralpak OD column, hexane : isopropanol = 70 : 30, 1.0 mL/min): tr = 21.1 min (major), tr =36.9 min (minor), ee = 91%.

2m, 4-(2-isopropylnaphthalen-1-yl)-2*H*-quinolizin-2-one

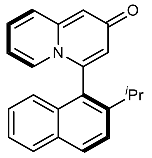

Following the general procedure **H**, **2m** was afforded as yellow solid (29.8 mg, 95 % yield), m.p. 120-121 °C. $[\alpha]_D^{25}$ -128.0 (c = 0.3, $CHCl_3$). **^{1}H NMR (500 MHz, Chloroform-*d*) δ**: 8.06 (dd, J = 16.9, 8.6 Hz, 1H), 7.94 (d, J = 8.1 Hz, 1H), 7.61 (dd, J = 10.2, 8.6 Hz, 1H), 7.53 (t, J = 7.5 Hz, 1H), 7.43 (t, J = 7.6 Hz, 1H), 7.31 (s, 1H), 7.24 (d, J = 8.6 Hz, 1H), 7.16 (dd, J = 14.7, 7.4 Hz, 1H), 7.10 (dd, J = 9.1, 6.5 Hz, 1H), 6.90 – 6.89 (m, 1H), 6.82 (s, 1H), 6.34 (t, J = 7.0 Hz, 1H), 2.79 (p, J = 6.8 Hz, 1H), 1.27 (d, J = 6.8 Hz, 3H), 1.20 (d, J = 6.8 Hz, 3H). **^{13}C NMR (126 MHz, Chloroform-*d*) δ**: 174.61, 144.81, 143.93, 142.17, 140.49, 131.16, 130.15, 130.07, 129.77, 128.20, 127.56, 127.32, 126.70, 125.24, 123.75, 123.47, 123.21, 122.93, 111.25, 30.35, 23.12, 22.36. **HRMS(ESI) m/z**: calculated for $[C_{22}H_{19}NO + H]^+$ 314.1545, found 314.1541. **HPLC data** (Chiralpak IA column, hexane : isopropanol = 70: 30, 1.0 mL/min): tr = 38.1 min (major), tr = 21.1 min (minor), ee = 93%.

2n, 4-(2-phenylnaphthalen-1-yl)-2*H*-quinolizin-2-one

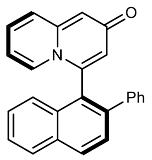

Following the general procedure **H**, **2n** was afforded as yellow solid (32.4 mg, 93 % yield), m.p. 51-52 °C. $[\alpha]_D^{25}$ +288.5 (*c* = 0.5, $CHCl_3$). **^{1}H NMR (500 MHz, Chloroform-*d*) δ**: 8.17 (d, *J* = 8.5 Hz, 1H), 8.04 (d, *J* = 8.2 Hz, 1H), 7.68 (d, *J* = 8.5 Hz, 1H), 7.62 (t, *J* = 7.5 Hz, 1H), 7.52 (t, *J* = 7.6 Hz, 1H), 7.41 (t, *J* = 8.1 Hz, 2H), 7.29 (d, *J* = 8.7 Hz, 1H), 7.24 – 7.23 (m, 3H), 7.16 (t, *J* = 7.7 Hz, 3H), 6.98 (s, 1H), 6.89 (s, 1H), 6.46 (d, *J* = 7.2 Hz, 1H). **^{13}C NMR (126 MHz, Chloroform-*d*) δ**: 172.97, 143.67, 142.36, 139.52, 138.56, 131.81, 130.16, 130.01, 128.46, 128.34, 128.05, 127.61, 127.39, 127.27, 127.16, 126.94, 126.00, 125.90, 125.49, 123.86, 123.56, 112.32, 110.52. **HRMS(ESI) m/z**: calculated for $[C_{25}H_{17}NO_2 + H]^+$ 348.1388, found 348.1386. **HPLC data** (Chiralpak OD column, hexane : isopropanol = 70 : 30, 1.0 mL/min): tr = 6.9 min (major), tr =10.3 min (minor), ee = 93%.

2o, 4-([1,2'-binaphthalen]-1'-yl)-2*H*-quinolizin-2-one

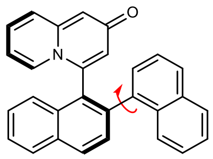

Following the general procedure **H**, **2o** was afforded as yellow solid (35.0 mg, 89 % yield), m.p. 80-81 °C. $[\alpha]_D^{25}$ -76.0 (*c* = 0.5, $CHCl_3$). PS: There are conformers existed around the red arrow. The rotation barrier is 18.9 kcal/mol by calculation at the M062X/6-311+G(d,p),SMD(Toluene)// M062X/6-31g(d) level. **^{1}H NMR (500 MHz, Chloroform-*d*) δ**: 8.19 (dd, *J* = 12.1, 8.4 Hz, 2H), 8.10 (d, *J* = 8.2 Hz, 2H), 7.85 (d, *J* = 8.1 Hz, 1H), 7.82 (d, *J* = 8.2 Hz, 1H), 7.77 (d, *J* = 8.3 Hz, 2H), 7.74 (t, *J* = 9.0 Hz, 1H), 7.70 – 7.64 (m, 4H), 7.60 (d, *J* = 8.5 Hz, 1H), 7.51 – 7.45 (m, 4H), 7.45 – 7.37 (m, 4H), 7.37 – 7.32 (m, 4H), 7.21 (t, *J* = 7.7 Hz, 2H), 7.16 (d, *J* = 9.2 Hz, 2H), 7.09 (dd, *J* = 9.2, 6.4 Hz, 2H), 7.06 – 6.95 (m, 1H), 6.91 (s, 1H), 6.56 (d, *J* = 2.7 Hz, 1H), 6.50 (dd, *J* = 7.5, 2.7 Hz, 2H), 6.47 (d, *J* = 6.3 Hz, 2H), 5.95 (s, 1H). **^{13}C NMR (126 MHz, Chloroform-*d*) δ**: 174.20, 173.76, 143.43, 143.32, 141.97, 141.12, 138.38, 137.81, 136.37, 134.91, 132.57, 132.53, 132.03, 131.91, 130.81, 130.73, 130.27, 130.20, 129.03, 129.01, 128.54, 128.30, 128.27, 127.93, 127.89, 127.70, 127.59, 127.57, 127.37, 127.33, 127.24, 127.18, 127.09, 127.04, 126.15, 126.10, 125.80, 125.32, 124.82, 124.66, 124.22, 124.10, 124.07, 123.98, 123.67, 123.41, 123.25, 123.12,

111.12, 110.70, 110.50, 110.45. **HRMS(ESI) m/z**: calculated for $[C_{29}H_{19}NO + H]^+$ 398.1545, found 398.1544. **HPLC data** (Chiralpak IA column, hexane : isopropanol = 80: 20, 1.0 mL/min): tr = 31.3 min (major), tr = 45.5 min (minor), ee = 99%.

2p, 4-(2-bromonaphthalen-1-yl)-2*H*-quinolizin-2-one

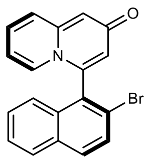

Following the general procedure **H**, **2p** was afforded as yellow solid (29.1 mg, 83 % yield), m.p. 62-63 °C. $[\alpha]_D^{25}$ -130.6 (c = 0.3, $CHCl_3$). **^{1}H NMR (500 MHz, Chloroform-*d*) δ**: 7.98 (dd, J = 8.5, 5.1 Hz, 2H), 7.82 (d, J = 8.8 Hz, 1H), 7.62 (t, J = 7.6 Hz, 1H), 7.51 (t, J = 7.7 Hz, 1H), 7.42 (d, J = 8.5 Hz, 1H), 7.31 (s, 1H), 7.14 – 7.09 (m, 2H), 6.87 (s, 1H), 6.82 (s, 1H), 6.40 (t, J = 6.9 Hz, 1H). **^{13}C NMR (126 MHz, Chloroform-*d*) δ**: 174.59, 143.87, 141.60, 131.66, 131.39, 130.96, 129.04, 128.86, 128.46, 127.69, 127.64, 127.62, 127.56, 126.32, 123.80, 123.57, 122.02, 111.58, 111.22. **HRMS(ESI) m/z**: calculated for $[C_{19}H_{12}BrNO + H]^+$ 350.0181, found 350.0171. **HPLC data** (Chiralpak AD column, hexane : isopropanol = 70 : 30, 1.0 mL/min): tr = 17.1 min (minor), tr = 22.0 min (major), ee = 66%.

2q, 4-(2-chloronaphthalen-1-yl)-2*H*-quinolizin-2-one

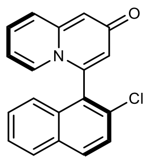

Following the general procedure **H**, **2q** was afforded as yellow solid (22.3 mg, 73 % yield), m.p. 59-60 °C. $[\alpha]_D^{25}$ -42.3 (c = 0.5, $CHCl_3$). **^{1}H NMR (500 MHz, Chloroform-*d*) δ**: 8.01 (dd, J = 10.6, 8.3 Hz, 2H), 7.85 – 7.54 (m, 2H), 7.48 (s, 1H), 7.33 (d, J = 7.8 Hz, 1H), 7.16 – 7.15 (m, 3H), 6.92 (s, 2H), 6.44 (s, 1H). **^{13}C NMR (126 MHz, Chloroform-*d*) δ:** 175.36, 145.03, 141.15, 139.23, 132.71, 132.30, 132.13, 128.81, 128.76, 128.67, 128.64, 127.57, 127.23, 127.08, 126.07, 124.86, 124.33, 112.92, 112.13. **HRMS(ESI) m/z**: calculated for $[C_{19}H_{12}ClNO + H]^+$ 306.0686, found 306.0682. **HPLC data** (Chiralpak AD column, hexane : isopropanol = 70 : 30, 1.0 mL/min): tr = 18.0 min (minor), tr = 23.0 min (major), ee = 63%.

2r, 9-bromo-4-(2-ethoxynaphthalen-1-yl)-2*H*-quinolizin-2-one

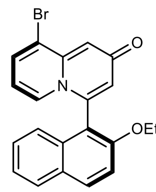

Following the general procedure **H**, **2r** was afforded as yellow solid (27.8 mg, 70 % yield), m.p. 58-59 °C. $[\alpha]_D^{25}$ -222.4 (c = 0.5, $CHCl_3$). **^{1}H NMR (500 MHz, Chloroform-*d*) δ**: 8.09 (d, J = 8.8 Hz, 1H), 7.91 (d, J = 6.8 Hz, 1H), 7.79 (t, J = 9.8 Hz, 1H), 7.53 (s, 1H), 7.44 – 7.41 (m, 3H), 7.30 (d, J = 9.9 Hz, 2H), 6.96 (s, 1H), 6.35 (s, 1H), 4.19 – 4.16 (m, 2H), 1.21 (s, 3H). **^{13}C NMR (126 MHz, Chloroform-*d*) δ:** 176.51, 154.21, 143.39, 142.76, 132.80, 132.60, 132.58, 132.32, 129.53, 128.92, 128.47, 128.28, 124.63, 123.20, 117.63, 114.65, 113.84, 111.06, 111.04, 64.93, 14.82. **HRMS(ESI) m/z**: calculated for $[C_{21}H_{16}BrNO_2 + H]^+$ 394.0443, found 394.0440. **HPLC data** (Chiralpak OD column, hexane : isopropanol = 70 : 30, 1.0 mL/min): tr = 9.5 min (major), tr =16.7 min (minor), ee = 84%.

2s, 8-bromo-4-(2-ethoxynaphthalen-1-yl)-2*H*-quinolizin-2-one

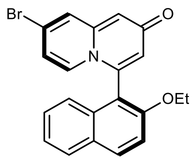

Following the general procedure **H**, **2s** was afforded as yellow solid (34.2 mg, 87 % yield), m.p. 65-66 °C. $[\alpha]_D^{25}$ -207.4 (c = 0.5, $CHCl_3$). **^{1}H NMR (500 MHz, Chloroform-*d*) δ**: 8.07 (d, J = 9.1 Hz, 1H), 7.90 – 7.88 (m, 1H), 7.43 (t, J = 3.7 Hz, 3H), 7.40 (d, J = 9.2 Hz, 1H), 7.36 – 7.35 (m, 1H), 7.16 (d, J = 7.7 Hz, 1H), 6.81 (s, 1H), 6.63 (s, 1H), 6.38 (dd, J = 7.8, 2.1 Hz, 1H), 4.20 – 3.17 (m, 2H), 1.25 (t, J = 6.9 Hz, 3H). **^{13}C NMR (126 MHz, Chloroform-*d*) δ:** 176.06, 154.26, 145.08, 141.70, 132.83, 132.36, 130.41, 128.94, 128.47, 128.30, 125.67, 124.65, 123.52, 123.21, 115.63, 114.25, 114.09, 113.83, 110.98, 64.95, 14.83. **HRMS(ESI) m/z**: calculated for $[C_{21}H_{16}BrNO_2 + H]^+$ 394.0443, found 394.0439. **HPLC data** (Chiralpak AD column, hexane : isopropanol = 70 : 30, 1.0 mL/min): tr = 33.6 min (major), tr =16.5 min (minor), ee = 88%.

2t, 8-chloro-4-(2-ethoxynaphthalen-1-yl)-2*H*-quinolizin-2-one

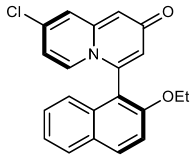

Following the general procedure **H**, **2t** was afforded as yellow solid (34.1 mg, 98 % yield), m.p. 47-48 °C. $[\alpha]_D^{25}$ -284.3 (c = 0.25, $CHCl_3$). **^{1}H NMR (500 MHz, Chloroform-*d*) δ**: 8.10 (d, J = 9.1 Hz, 1H), 7.93 – 7.91 (m, 1H), 7.47 – 7.45 (m, 2H), 7.42 (d, J = 9.1 Hz, 1H), 7.41 – 7.38 (m, 1H), 7.27 (d, J = 7.7 Hz, 2H), 6.83 (s, 1H), 6.69 (s, 1H), 6.30 (dd, J = 7.7, 2.4 Hz, 1H), 4.21 (qd, J = 7.0, 4.6 Hz, 2H), 1.27 (t, J = 6.9 Hz, 3H). **^{13}C NMR (126 MHz, Chloroform-*d*) δ**: 175.01, 153.23, 143.88, 140.53, 134.30, 131.75, 131.37, 129.75, 127.91, 127.41, 127.30, 125.34, 123.66, 122.26, 121.09, 113.35, 112.78, 112.25, 109.98, 63.94, 13.81. **HRMS(ESI) m/z**: calculated for $[C_{21}H_{16}ClNO_2 + H]^+$ 350.0948, found 350.0945. **HPLC data** (Chiralpak OD column, hexane : isopropanol = 70 : 30, 1.0 mL/min): tr = 9.2 min (major), tr =21.8 min (minor), ee = 95%.

2u, 7-bromo-4-(2-ethoxynaphthalen-1-yl)-2*H*-quinolizin-2-one

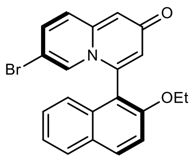

Following the general procedure **H**, **2u** was afforded as yellow solid (21.9 mg, 56 % yield), m.p. 73-74 °C. $[\alpha]_D^{25}$ -289.8 (c = 0.2, $CHCl_3$). **^{1}H NMR (500 MHz, Chloroform-*d*) δ**: 8.12 (d, J = 9.1 Hz, 1H), 7.94 (dd, J = 6.7, 2.7 Hz, 1H), 7.48 (q, J = 3.3 Hz, 2H), 7.45 – 7.42 (m, 2H), 7.39 – 7.27 (m, 1H), 7.20 (d, J = 9.6 Hz, 1H), 7.12 (dd, J = 9.6, 1.7 Hz, 1H), 6.84 (d, J = 2.7 Hz, 1H), 6.77 (d, J = 2.8 Hz, 1H), 4.28 – 4.20 (m, 2H), 1.28 (t, J = 7.0 Hz, 3H). **^{13}C NMR (126 MHz, Chloroform-d) δ**: 174.94, 153.14, 142.10, 140.18, 131.85, 131.45, 130.67, 127.99, 127.93, 127.42, 127.27, 125.82, 124.49, 123.67, 122.39, 113.21, 112.70, 111.44, 105.02, 63.85, 13.77. **HRMS(ESI) m/z**: calculated for $[C_{21}H_{16}BrNO_2 + H]^+$ 394.0443, found 394.0439. **HPLC data** (Chiralpak IG column, hexane : isopropanol = 45: 55, 1.0 mL/min): tr = 37.7 min (major), tr = 18.3 min (minor), ee = 94%.

2v, 4-(2-ethoxynaphthalen-1-yl)-7-methyl-2*H*-quinolizin-2-one

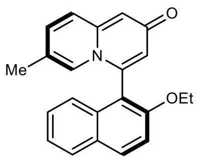

Following the general procedure **H**, **2v** was afforded as yellow solid (31.9 mg, 96 % yield), m.p. 47-48 °C. $[\alpha]_D^{25}$ -261.0 (c = 0.5, $CHCl_3$). **^{1}H NMR (500 MHz, Chloroform-*d*) δ**: 8.10 (d, J = 9.1 Hz, 1H), 7.93 (dd, J = 6.4, 3.3 Hz, 1H), 7.46 – 7.44 (m, 3H), 7.39 (d, J = 5.7 Hz, 1H), 7.27 (d, J =

9.2 Hz, 1H), 7.13 (s, 1H), 7.01 (d, *J* = 9.1 Hz, 1H), 6.86 – 6.86 (m, 1H), 6.81 (s, 1H), 4.22 – 4.19 (m, 2H), 2.01 (s, 3H), 1.24 (t, *J* = 7.0 Hz, 3H). **^{13}C NMR (126 MHz, Chloroform-*d*) δ**: 174.36, 153.18, 142.97, 140.04, 131.54, 131.39, 130.85, 127.98, 127.31, 127.03, 125.32, 124.99, 123.54, 123.24, 122.59, 120.65, 114.27, 113.05, 110.28, 63.94, 17.10, 13.79. **HRMS(ESI) m/z**: calculated for $[C_{22}H_{19}NO_2 + H]^+$ 330.1494, found 330.1492. **HPLC data** (Chiralpak OD column, hexane : isopropanol = 70 : 30, 1.0 mL/min): tr = 7.7min (major), tr =13.1 min (minor), ee = 94%.

2w, 6-(2-ethoxynaphthalen-1-yl)-8*H*-pyrido[1,2-*a*]pyrazin-8-one

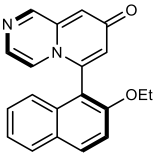

Following the general procedure **H**, **2w** was afforded as white solid (29.0 mg, 92 % yield), m.p. 181-182 °C $[\alpha]_D^{25}$ +43.2 (*c* = 0.5, $CHCl_3$). **^{1}H NMR (500 MHz, Chloroform-*d*) δ**: 8.74 (s, 1H), 8.09 (d, *J* = 9.1 Hz, 1H), 7.91 – 7.89 (m, 1H), 7.45 – 7.42 (m, 3H), 7.33 (dd, *J* = 11.6, 5.1 Hz, 2H), 7.03 (d, *J* = 4.8 Hz, 1H), 6.93 (d, *J* = 8.9 Hz, 2H), 4.19 (p, *J* = 6.7 Hz, 2H), 1.24 (t, *J* = 6.9 Hz, 3H). **^{13}C NMR (126 MHz, Chloroform-*d*) δ**: 175.78, 153.26, 151.31, 140.59, 132.02, 131.26, 127.84, 127.59, 127.49, 127.36, 126.41, 123.67, 122.06, 119.10, 112.92, 112.90, 112.58, 111.93, 63.87, 13.77. **HRMS(ESI) m/z**: calculated for $[C_{20}H_{16}N_2O_2 + H]^+$ 317.1290, found 317.1282. **HPLC data** (Chiralpak AD column, hexane : isopropanol = 70 : 30, 1.0 mL/min): tr = 14.4 min (minor), tr =15.7 min (major), ee = 85%.

2x, 4-(2-ethoxynaphthalen-1-yl)-7-(4-((((3a*S*,5a*R*,8a*R*,8b*S*)-2,2,7,7-tetramethyltetrahydro-3a*H*-bis([1,3]dioxolo)[4,5-b:4',5'-d]pyran-3a-yl)methoxy)methyl)phenyl)-2*H*-quinolizin-2-one

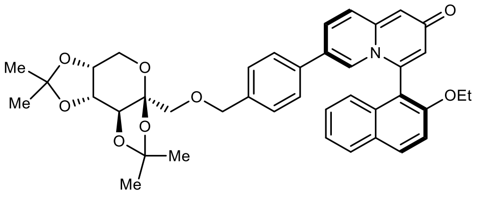

Following the general procedure **H**, **2x** was afforded as yellow solid (51.7 mg, 78 % yield), m.p. 55-56 °C. $[\alpha]_D^{25}$ +79.0 (*c* = 1.3, $CHCl_3$). **^{1}H NMR (500 MHz, Chloroform-*d*) δ**: 8.11 (d, *J* = 9.1 Hz, 1H), 7.93 (dd, *J* = 7.5, 2.6 Hz, 1H), 7.56 (s, 1H), 7.46 (d, *J* = 4.9 Hz, 6H), 7.32 – 7.31 (m, 3H), 7.15 (d, *J* = 7.9 Hz, 2H), 6.94 – 6.92 (m, 1H), 4.64 (t, *J* = 11.5 Hz, 1H), 4.60 – 4.57 (m, 2H), 4.42 (d, *J* = 2.6 Hz, 1H), 4.26 – 4.20 (m, 3H), 3.93 (dd, *J* = 13.0, 1.8 Hz, 1H), 3.75 (d, *J* = 13.0 Hz, 1H),

3.62 – 3.56 (m, 2H), 1.56 (s, 3H), 1.41 (s, 3H), 1.38 (s, 3H), 1.34 (s, 3H), 1.23 (t, *J* = 6.9 Hz, 3H). **^{13}C NMR (126 MHz, Chloroform-*d*) δ:** 154.36, 153.00, 144.36, 138.91, 134.55, 133.25, 132.34, 130.41, 129.02, 128.85, 128.62, 128.45, 128.33, 127.80, 126.48, 126.33, 125.81, 124.71, 123.18, 123.00, 120.55, 113.75, 108.84, 108.54, 102.61, 73.00, 71.73, 70.96, 70.16, 65.01, 61.01, 26.59, 25.80, 25.44, 24.04, 14.83. **HRMS(ESI) m/z**: calculated for $[C_{40}H_{41}NO_8 + H]^+$ 664.2910, found 664.2906.

2y, 4-(2-ethoxynaphthalen-1-yl)-7-(4-((((*R*)-2,5,7,8-tetramethyl-2-((4*S*, 8*R*)-4,8,12-trimethyltridec-yl)chroman-6-yl)oxy)methyl)phenyl)-2*H*-quinolizin-2-one

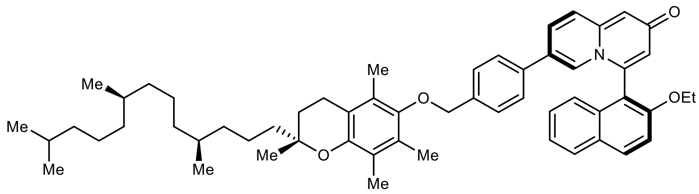

Following the general procedure **H**, **2y** was afforded as yellow solid (74.2 mg, 89 % yield), m.p. 35-36 °C. $[\alpha]_D^{25}$ -73 (*c* = 0.5, $CHCl_3$). **^{1}H NMR (500 MHz, Chloroform-*d*) δ**: 8.06 (d, *J* = 9.1 Hz, 1H), 7.89 (d, *J* = 7.4 Hz, 1H), 7.61 (s, 1H), 7.51 (s, 1H), 7.44 (d, *J* = 6.7 Hz, 4H), 7.39 (s, 3H), 7.18 (d, *J* = 7.9 Hz, 2H), 6.89 (s, 1H), 6.83 (s, 1H), 4.63 (s, 2H), 4.19 (q, *J* = 7.0 Hz, 2H), 2.58 (dd, *J* = 17.3, 10.0 Hz, 3H), 2.22 (d, *J* = 14.6 Hz, 3H), 2.16 (s, 3H), 2.12 (s, 3H), 2.09 (s, 3H), 1.79 (dq, *J* = 18.3, 6.5 Hz, 2H), 1.54 (dq, *J* = 13.4, 7.3, 6.3 Hz, 3H), 1.39 (d, *J* = 6.6 Hz, 2H), 1.31 – 1.18 (m, 12H), 1.09 – 1.05 (m, 3H), 0.93 – 0.79 (m, 15H). **^{13}C NMR (126 MHz, Chloroform-*d*) δ**: 174.89, 153.25, 147.01, 146.89, 143.01, 140.60, 137.28, 134.40, 131.66, 131.51, 128.04, 128.00, 127.49, 127.41, 127.27, 127.24, 127.20, 126.71, 126.03, 125.59, 125.23, 124.75, 123.80, 123.61, 122.55, 122.00, 116.64, 113.85, 112.82, 73.86, 73.24, 72.88, 63.99, 39.03, 38.37, 36.45, 36.41, 36.29, 31.80, 31.69, 30.28, 26.98, 23.80, 23.44, 22.87, 21.73, 21.63, 20.02, 18.76, 18.67, 13.86, 11.80, 10.93, 10.81. **HRMS(ESI) m/z**: calculated for $[C_{57}H_{71}NO_4 + H]^+$ 834.5641, found 834.5638.

2z, 4-(2-ethoxynaphthalen-1-yl)-7-(4-((((1*R*,2*S*,5*R*)-2-isopropyl-5-methyl cyclohexyl)oxy)methyl) phenyl)-2*H*-quinolizin-2-one

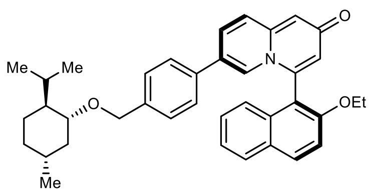

Following the general procedure **H**, **2z** was afforded as yellow solid (41.4 mg, 74 % yield), m.p. 55-56 °C. $[\alpha]_D^{25}$ -164.6 (c = 1.5, $CHCl_3$). **^{1}H NMR (500 MHz, Chloroform-*d*) δ**: 8.10 (d, J = 9.1 Hz, 1H), 7.93 (dd, J = 7.8, 2.1 Hz, 1H), 7.51 (d, J = 1.4 Hz, 1H), 7.48 (t, J = 2.7 Hz, 2H), 7.43 (d, J = 9.2 Hz, 1H), 7.40 (dd, J = 3.6, 1.2 Hz, 2H), 7.32 (d, J = 7.8 Hz, 3H), 7.16 – 7.15 (m, 2H), 6.91 (d, J = 2.7 Hz, 1H), 6.82 (d, J = 2.7 Hz, 1H), 4.64 (d, J = 11.6 Hz, 1H), 4.37 (d, J = 11.6 Hz, 1H), 4.22 (q, J = 7.0 Hz, 2H), 3.18 (td, J = 10.5, 4.1 Hz, 1H), 2.27 (td, J = 7.0, 2.8 Hz, 1H), 2.18 (ddt, J = 12.2, 3.8, 1.9 Hz, 1H), 1.67 (ddt, J = 18.5, 12.9, 3.4 Hz, 2H), 1.38 (dd, J = 6.6, 3.3 Hz, 2H), 1.30 (d, J = 4.2 Hz, 3H), 1.25 (t, J = 7.0 Hz, 3H), 0.96 (d, J = 6.6 Hz, 3H), 0.91 (d, J = 7.1 Hz, 3H), 0.73 (d, J = 6.9 Hz, 3H). **^{13}C NMR (126 MHz, Chloroform-*d*) δ**: 174.87, 153.21, 142.97, 140.54, 138.43, 134.01, 131.62, 131.48, 128.09, 127.97, 127.39, 127.38, 127.16, 125.61, 125.48, 125.09, 124.52, 123.69, 123.58, 122.55, 113.81, 112.78, 110.40, 78.11, 68.81, 63.94, 47.24, 39.26, 33.49, 30.53, 28.70, 24.54, 22.20, 21.34, 19.99, 15.07, 13.84. **HRMS(ESI) m/z**: calculated for $[C_{38}H_{41}NO_3 + H]^+$ 560.3165, found 560.3161.

Racemization experiments

Compounds **2g, 2i, 2k, 2n** or **2q** (0.1 mmol) was dissolved in chlorobenzene (2.0 mL) in a sealed tube, respectively. The tube was placed in a metal heating plate at 140 °C. At given interval of time, small samples were removed and subjected into HPLC to measure the enantiomeric excess.

The enantiomerisation barrier, corresponding to barrier to rotation for **2g, 2i, 2k, 2n** and **2q** atropisomers, was obtained by kinetic of racemization of an enantiomer.[3] The slope of the first-order kinetic line gives the racemization constant ($k_{racemization} = 2 \times k_{enantiomerisation}$). Eyring equation gives the enantiomerisation barrier from enantiomerisation constant ($k_{enantiomerisation}$), R = 8.31451 J K^{-1} mol^{-1}, $h = 6.62608\times10^{-34}$ J s and $k_B = 1.38066\times10^{-23}$ J K^{-1}.

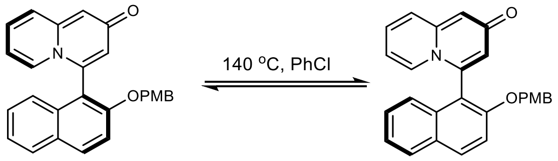

2g **ent-2g**

Time (s)	ee	-ln (ee)
0	87.5	-4.471638793
1800	86.22	-4.456902169
3600	86.24	-4.457134107

14400	80.86	-4.392719264
57600	75.1	-4.318820559
86400	69.66	-4.243626265
115200	69.98	-4.248209487
144000	68.34	-4.224495247
201600	63.96	-4.158257888
388800	42.26	-3.743841012
475200	33.44	-3.509752788
561600	28.56	-3.352007137
648000	21.66	-3.075467242
734400	16.04	-2.775085602
820800	15.8	-2.76000994
907200	10.56	-2.357073278
993600	7.06	-1.954445052

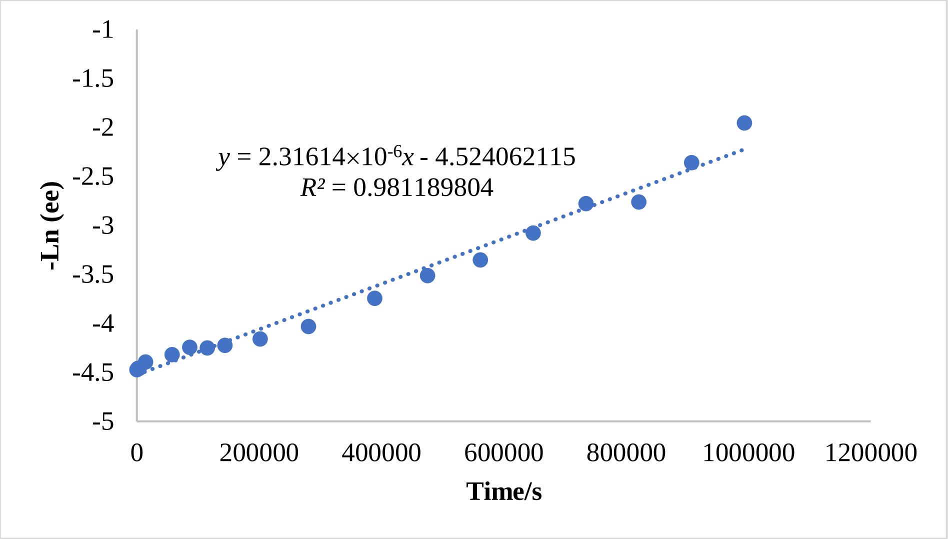

Supplementary Fig. 2. Racemization experiments of 2g.

$k_{\mathbf{2g}\ \text{racemization}} = 2.31614\times10^{-6}\ \text{s}^{-1}$, $k_{\mathbf{2g}\ \text{enantiomerisation}} = 1.15807\times10^{-6}\ \text{s}^{-1}$;

$T^{140}{}_{1/2} = 83.1$ h; $\Delta G^{\neq} = 35.08$ kcal mol^{-1}.

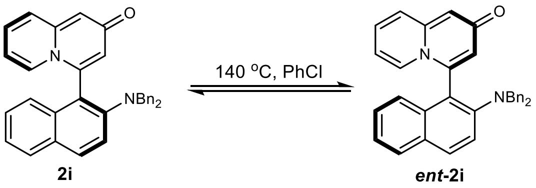

Time (s)	ee	-ln (ee)
0	96.76	-4.572233686
43200	96.06	-4.564972996
86400	96.0	-4.564348191
129600	95.3	-4.557029811

172800	94.58	-4.549446037
216000	93.18	-4.534533106
302400	92.76	-4.530015512
388800	91.28	-4.513931706
475200	89.9	-4.498697941
561600	89.08	-4.489534842
648000	87.32	-4.469579532
734400	87.16	-4.46774551

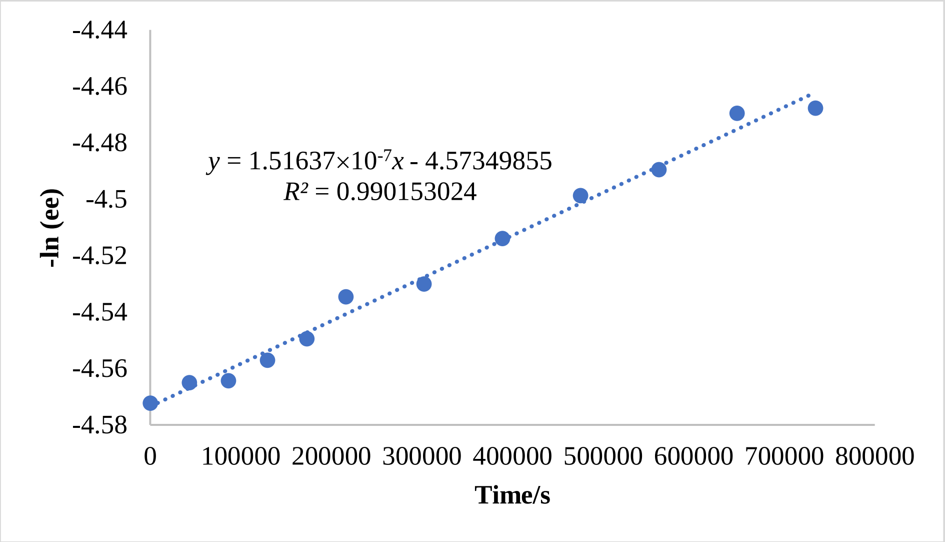

Supplementary Fig. 3. Racemization experiments of 2i.

$k_{\mathbf{2i}\text{ racemization}} = 1.51637\times10^{-7}\ \mathrm{s}^{-1}$, $k_{\mathbf{2i}\text{ enantiomerisation}} = 7.58185\times10^{-8}\ \mathrm{s}^{-1}$;

$T^{140}{}_{1/2} = 1269.7$ h; $\Delta G^{\neq} = 37.32$ kcal mol^{-1}.

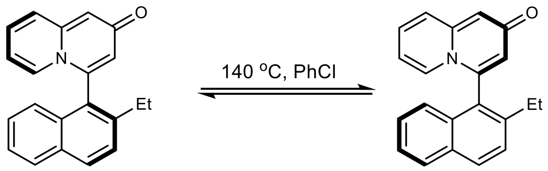

2k *ent*-**2k**

Time (s)	ee	-ln (ee)
0	85.0	-4.4426513
86400	84.92	-4.4417096
172800	84.6	-4.4379343
216000	84.56	-4.4374613
302400	84.32	-4.4346191
388800	84.28	-4.4341446
475200	84.14	-4.4324821

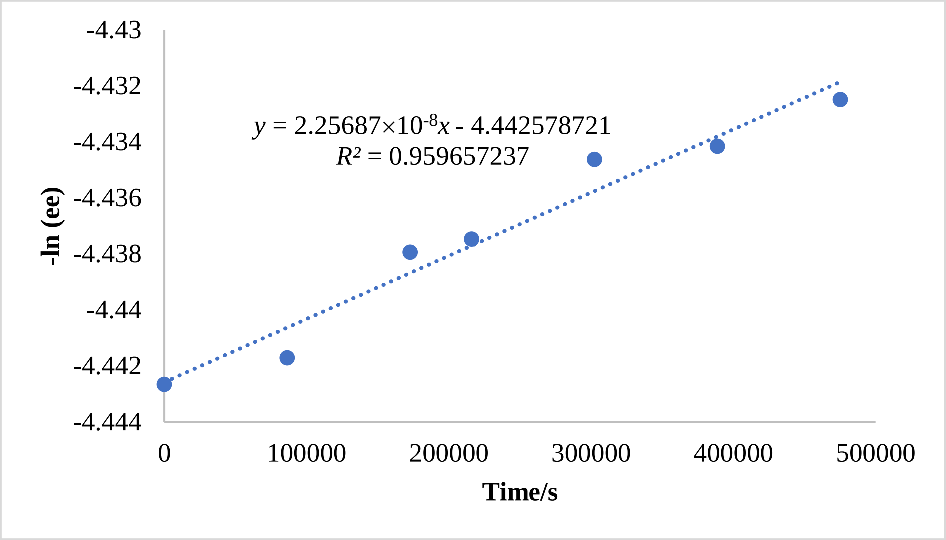

Supplementary Fig. 4. Racemization experiments of 2k.

$k_{\mathbf{2k}\ \text{racemization}} = 2.25687\times10^{-8}\ s^{-1}$, $k_{\mathbf{2k}\ \text{enantiomerisation}} = 1.28435\times10^{-8}\ s^{-1}$;

$T^{140}{}_{1/2} = 8531.3$ h; $\Delta G^{\neq} = 38.88$ kcal mol^{-1}.

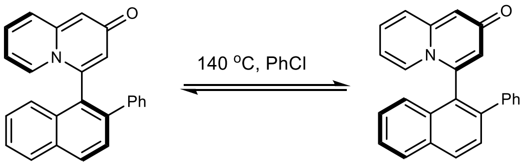

Time (s)	ee	-ln (ee)
0	92.0	-4.521788577
86400	90.36	-4.503801692
129600	87.54	-4.472095832
172800	86.18	-4.456438132
216000	87.02	-4.466137977
302400	84.46	-4.43627805
388800	83.19	-4.421127148
475200	82.46	-4.412313327
568800	81.62	-4.40207433
648000	79.9	-4.380775853

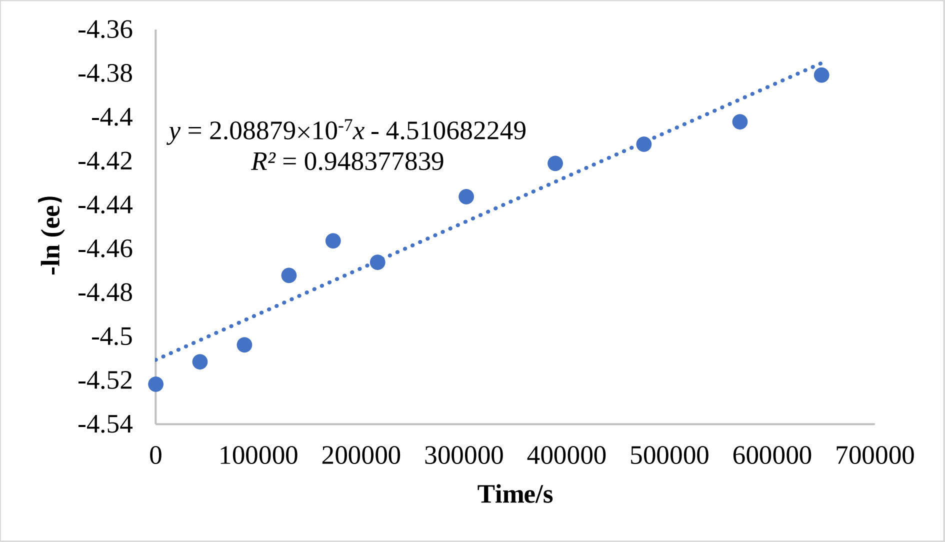

Supplementary Fig. 5. Racemization experiments of 2n.

$k_{\mathbf{2n}\ \text{racemization}} = 2.08879\times10^{-7}\ s^{-1}$, $k_{\mathbf{2n}\ \text{enantiomerisation}} = 1.04439\times10^{-7}\ s^{-1}$;

$T^{140}{}_{1/2} = 921.8$ h; $\Delta G^{\neq} = 37.05$ kcal mol^{-1}.

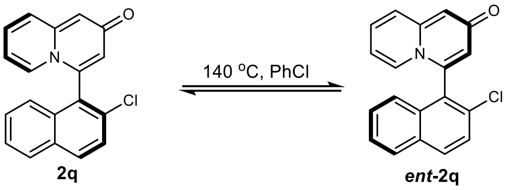

Time (s)	ee	-ln (ee)
0	62.84	-4.140591813
43200	62.56	-4.136126096
129600	62.08	-4.128423876
216000	61.56	-4.120012309
302400	61.36	-4.116758157
475200	60.93	-4.109725664
568800	60.63	-4.10478982

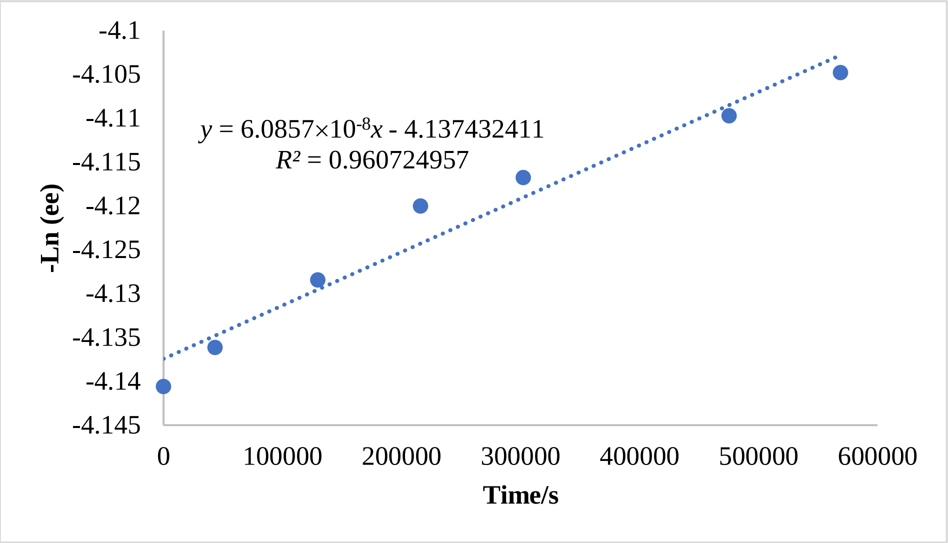

Supplementary Fig. 6. Racemization experiments of 2q.

$k_{\mathbf{2q}\ \text{racemization}} = 6.0857\times10^{-8}\ \text{s}^{-1}$, $k_{\mathbf{2q}\ \text{enantiomerisation}} = 3.0428\times10^{-8}\ \text{s}^{-1}$;

$T^{140}{}_{1/2} = 3163.8$ h; $\Delta G^{\neq} = 38.21$ kcal mol^{-1}.

Procedure for gram-scale reaction of 2b.

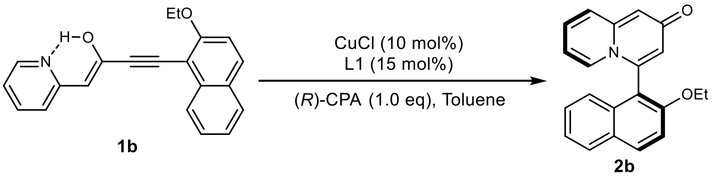

To a flame-dried round-bottom flask equipped with a stirring bar was added CuCl (40 mg, 10 mol%), **L1** (340 mg, 15 mol%) and toluene (50 mL). The reaction was stirred at room temperature for 30 min. Then the corresponding substrate **1b** (1260 mg, 4 mmol, 1.0 eq) and chiral phosphoric acid (1390 mg, 4 mmol, 1.0 eq) were added. The reaction was stirred at 20 °C for 36 h. Then, the mixture was diluted with EA and washed with 1 N NaOH for three times. The combined extracts were dried over anhydrous Na_2SO_4, filtered and concentrated in vacuo. The product was purified by column chromatograph over silica gel affording **2b** in 86% yield (1.09 g) with 92% ee.

Synthetic transformations.

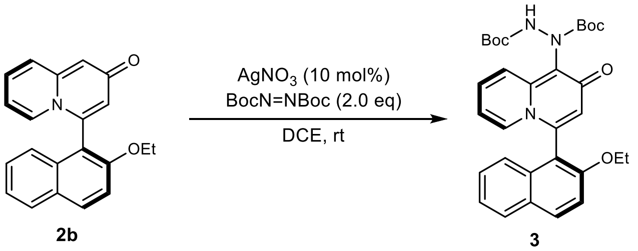

To a 2 dram scintillation vial equipped with a magnetic stirring bar was added substrate **2b** (31.5 mg, 1.0 eq), diazene compound (46 mg, 2.0 eq) and $AgNO_3$ (1.7 mg, 10 mol%). The vial was then charged with DCE (1.0 mL) and stirred at room temperature for a certain time. After the completion of the reaction (detected by TLC), saturated NH_4Cl aqueous was added and the reaction mixture was exacted with DCM (10 mL×3). The combined extracts were washed with brine, dried with Na_2SO_4, filtered and concentrated in vacuo. The residue was purified by column chromatograph over silica gel to afford **3**.

3, di-*tert*-butyl 1-(4-(2-ethoxynaphthalen-1-yl)-2-oxo-2*H*-quinolizin-1-yl) -hydrazine-1,2-dicarboxylate

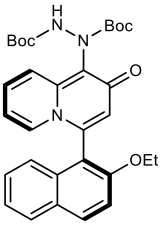

26.2 mg, 48% yield, white solid, m.p. 111-112 °C. $[\alpha]_D^{25}$ -160.3 (c = 0.4, $CHCl_3$). **^{1}H NMR (500 MHz, Chloroform-*d*) δ**: 8.42 – 8.26 (m, 1H), 8.09 (d, J = 9.2 Hz, 1H), 7.96 – 7.90 (m, 1H), 7.42 (td, J = 18.2, 14.6, 11.2 Hz, 5H), 7.31 (d, J = 8.5 Hz, 1H), 6.96 (s, 1H), 6.45 (t, J = 7.1 Hz, 1H), 4.19 (ddd, J = 16.6, 10.5, 4.9 Hz, 2H), 1.62 – 1.40 (m, 18H), 1.30 – 1.28 (m, 3H). **^{13}C NMR (126 MHz, Chloroform-*d*) δ**: 170.33, 154.86, 153.22, 142.46, 140.09, 138.23, 131.53, 128.29, 127.88, 127.30, 127.18, 125.60, 123.60, 122.57, 120.82, 113.77, 112.79, 111.44, 79.59, 63.85, 28.67, 27.35, 27.32, 27.16, 26.96, 21.66, 13.78. **HRMS(ESI) m/z**: calculated for $[C_{31}H_{35}N_3O_6 + H]^+$ 546.2604, found 546.2594. **HPLC data** (Chiralpak AD column, hexane : isopropanol = 85: 15, 1.0 mL/min):, tr = 10.2 min (major), tr = 19.6 min (minor), ee = 88%.

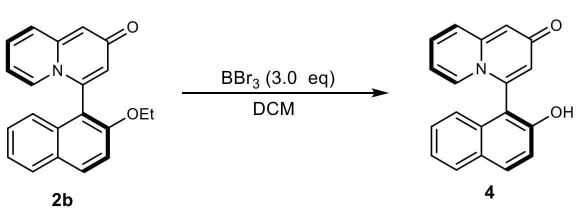

2b **4**

To a 2 dram scintillation vial equipped with a magnetic stirring bar was added substrate **2b** (315 mg, 1.0 mmol, 1.0 eq). The vial was then charged with DCM (10 mL) and stirred at -78 °C for a certain time. Then BBr_3 (750 mg, 3 mmol, 3.0 eq) was added slowly. The vial was warmed up to room temperature and stirred for a certain time. After the completion of the reaction detected

by TLC, saturated NH_4Cl aqueous was added and the reaction mixture was exacted with DCM (10 mL×3). The combined extracts were washed with brine, dried with Na_2SO_4, filtered and concentrated in vacuo. The residue was purified by column chromatograph over silica gel to afford **4**.

4, 4-(2-hydroxynaphthalen-1-yl)-2*H*-quinolizin-2-one

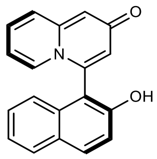

273 mg, 95% yield, white solid, m.p. 76-77 °C. $[\alpha]_D^{25}$ -152.5 (c = 0.3, MeOH). **^{1}H NMR (500 MHz, DMSO-d_6) δ**: 8.07 (d, J = 9.0 Hz, 1H), 7.95 (dd, J = 7.0, 2.2 Hz, 1H), 7.72 (d, J = 9.0 Hz, 1H), 7.61 (d, J = 7.4 Hz, 1H), 7.46 (dd, J = 9.0, 6.6 Hz, 1H), 7.42 – 7.37 (m, 3H), 7.22 – 7.20 (m, 1H), 6.97 (d, J = 2.8 Hz, 1H), 6.82 (d, J = 2.8 Hz, 1H), 6.78 (t, J = 7.0 Hz, 1H), 4.40 (s, 1H). **^{13}C NMR (126 MHz, DMSO-d_6) δ:** 174.60, 154.19, 145.54, 142.08, 132.84, 132.48, 129.91, 128.91, 128.30, 128.22, 125.79, 124.55, 123.87, 122.85, 118.82, 113.20, 111.55, 110.71, 59.92, 49.07. **HRMS(ESI) m/z**: calculated for $[C_{40}H_{30}N_2O_4 + H]^+$ 288.1025, found 288.1021. **HPLC data** (Chiralpak IA column, hexane : isopropanol = 65: 35, 1.0 mL/min): tr = 25.2 min (major), tr = 33.0 min (minor), ee = 92%.

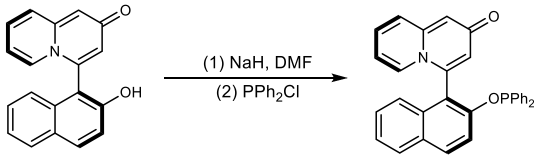

4 **5**

To a 2 dram scintillation vial equipped with a magnetic stirring bar was added substrate **4** (28.8 mg, 0.1 mmol, 1.0 eq), DMF (1.0 mL). The vial was cooled to 0 °C and stirred at the same temperature for a 10 min. Then NaH (60% dispersion in mineral oil) (8.0 mg, 0.2 mmol, 2.0 eq) was added and the mixture was stirred at this temperature for 30 min. Subsequently, $ClPPh_2$ (44 mg, 0.2 mmol, 2.0 eq) was added and the reaction was warmed up to room temperature. After the completion of the reaction detected by TLC, saturated NH_4Cl aqueous was added and the reaction mixture was exacted with EA (10 mL×3). The combined extracts were washed with brine, dried with Na_2SO_4, filtered and concentrated in vacuo. The residue was purified by column chromatograph over silica gel to afford **5**.

5, 4-(2-((diphenylphosphanyl)oxy)naphthalen-1-yl)-2*H*-quinolizin-2-one

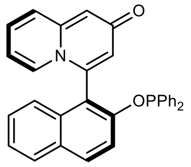

28.5 mg, 61% yield, white solid, m.p. 72-73 °C. $[\alpha]_D^{25}$ -84.0 (c = 0.9, $CHCl_3$). **^{1}H NMR (500 MHz, Chloroform-*d*) δ**: 7.98 (d, J = 9.1 Hz, 1H), 7.91 – 7.89 (m, 1H), 7.85 (d, J = 9.1 Hz, 1H), 7.72 – 7.68 (m, 2H), 7.50 (dtd, J = 8.1, 4.3, 1.5 Hz, 2H), 7.49 – 7.37 (m, 7H), 7.26 – 7.20 (m, 4H), 7.00 (dd, J = 9.1, 6.5 Hz, 1H), 6.81 (d, J = 2.7 Hz, 1H), 6.75 (d, J = 2.7 Hz, 1H), 6.28 – 6.25 (m, 1H). **^{13}C NMR (126 MHz, Chloroform-*d*) δ**: 174.24, 146.28, 146.22, 144.06, 138.97, 131.97, 131.57, 130.93, 130.64, 130.55, 129.89, 129.81, 128.53, 127.93, 127.83, 127.58, 127.56, 127.48, 127.43, 125.61, 125.21, 123.26, 123.04, 119.22, 117.70, 111.33, 110.69. **^{31}P NMR (202 MHz, Chloroform-*d*) δ**: 32.15. **HRMS(ESI) m/z**: calculated for $[C_{31}H_{22}NO_2P + H]^+$ 472.1466, found 472.1461. **HPLC data** (Chiralpak IA column, hexane : isopropanol = 70: 30, 1.0 mL/min): tr = 22.6 min (major), tr = 17.6 min (minor), ee = 90%.

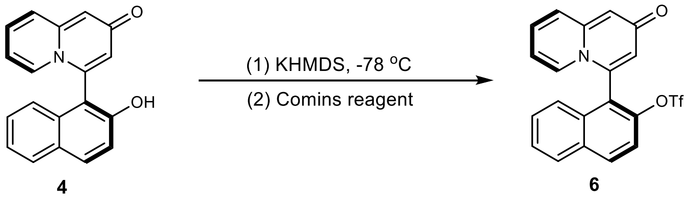

To a flame-dried round-bottom flask equipped with a magnetic stirring bar was added substrate **4** (288 mg, 1.0 mmol, 1.0 eq). The vial was then charged with THF (10 mL) and stirred at -78 °C for a certain time. Then KHMDS (1.0 M in THF, 2.0 mL, 2.0 eq) was added. The reaction was stirred at the same temperature for 1.0 h. Then Comins reagent (780 mg, 2.0 eq) was added in portions and the reaction was stirred at the same temperature for same time. After the completion of the reaction detected by TLC, saturated NH_4Cl aqueous was added and the reaction mixture was exacted with EA (10 mL×3). The combined extracts were washed with brine, dried with Na_2SO_4, filtered and concentrated in vacuo. The residue was purified by column chromatograph over silica gel to afford **6**.

6, 1-(2-oxo-2*H*-quinolizin-4-yl)naphthalen-2-yl trifluoromethanesulfonate

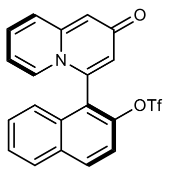

348.6 mg, 83% yield, white solid, m.p. 94-95 °C. $[\alpha]_D^{25}$ -52.8 (c = 0.5, $CHCl_3$). **^{1}H NMR (500 MHz, Chloroform-*d*) δ**: 8.27 (d, J = 9.2 Hz, 1H), 8.09 (d, J = 8.2 Hz, 1H), 7.69 (t, J = 7.6 Hz, 1H), 7.64 – 7.58 (m, 2H), 7.48 (d, J = 8.9 Hz, 1H), 7.38 – 7.31 (m, 3H), 7.10 (s, 1H), 7.02 (s, 1H), 6.65 (t, J = 6.9 Hz, 1H). **^{13}C NMR (126 MHz, Chloroform-*d*) δ**: 172.57, 144.56, 143.79, 136.95, 133.07, 131.56, 130.42, 129.36, 128.78, 128.43, 128.10, 127.38, 125.08, 124.02, 123.54, 120.96, 118.62, 115.88, 113.48, 110.79. **^{19}F NMR (470 MHz, Chloroform-*d*) δ**: -73.80. **HRMS(ESI) m/z**: calculated for $[C_{20}H_{12}F_3NO_4S + H]^+$ 420.0517, found 420.0507. **HPLC data** (Chiralpak OD column, hexane : isopropanol = 70: 30, 1.0 mL/min): tr = 7.0 min (major), tr = 10.7 min (minor), ee = 91%.

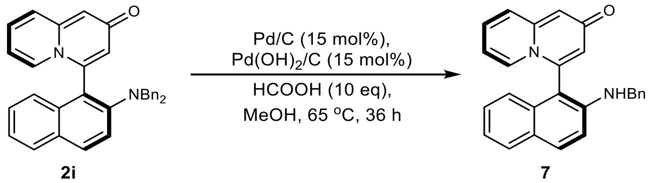

To a 2 dram scintillation vial equipped with a magnetic stirring bar was added substrate **2i** (46.6 mg, 0.1 mmol, 1.0 eq), HCOOH (46 mg, 1 mmol, 10 eq), Pd/C (0.015 mmol, 15 mol%), PdOH/C (0.015 mmol, 15 mol%) and MeOH (1.0 mL). The vial was stirred at 65 °C for a certain time. After the completion of the reaction detected by TLC, saturated NH_4Cl aqueous was added and the reaction mixture was exacted with DCM (10 mL×3). The combined extracts were washed with brine, dried with Na_2SO_4, filtered and concentrated in vacuo. The residue was purified by column chromatograph over silica gel to afford **7**.

7, 4-(2-(benzylamino)naphthalen-1-yl)-2*H*-quinolizin-2-one

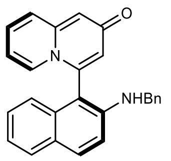

30.4 mg, 81% yield, white solid, m.p. 217-218 °C. $[\alpha]_D^{25}$ -43.0 (c = 0.2, $CHCl_3$). **^{1}H NMR (500 MHz, Chloroform-*d*) δ**: 7.86 (d, J = 9.0 Hz, 1H), 7.78 (d, J = 8.0 Hz, 1H), 7.37 – 7.34 (m, 2H), 7.29 – 7.27 (m, 6H), 7.21 (q, J = 4.4 Hz, 1H), 7.13 (dd, J = 8.8, 4.8 Hz, 2H), 7.02 (d, J = 8.4 Hz,

1H), 6.97 (d, J = 2.8 Hz, 1H), 6.72 (d, J = 2.7 Hz, 1H), 6.38 (t, J = 6.9 Hz, 1H), 5.14 (s, 1H), 4.54 (dd, J = 6.0, 3.8 Hz, 2H). **^{13}C NMR (126 MHz, Chloroform-*d*) δ**: 175.06, 144.66, 143.14, 140.72, 137.96, 131.55, 130.88, 128.25, 127.90, 127.65, 127.37, 126.96, 126.19, 126.07, 125.90, 125.71, 123.65, 121.63, 121.04, 113.16, 111.31, 110.89, 106.99, 46.30. **HRMS(ESI) m/z**: calculated for $[C_{26}H_{20}N_2O + H]^+$ 377.1654, found 377.1651. **HPLC data** (Chiralpak IG column, hexane : isopropanol = 50: 50, 1.0 mL/min): tr = 28.0 min (major), tr = 13.6 min (minor), ee = 97%.

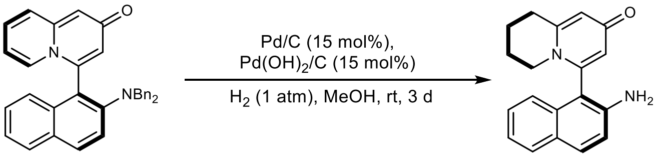

2i **8**

To a 2 dram scintillation vial equipped with a magnetic stirring bar was added substrate **2i** (46 mg, 0.1 mmol, 1.0 eq), Pd/C (0.015 mmol, 15 mol%), $Pd(OH)_2$/C (0.015 mmol, 15 mol%) and MeOH (1.0 mL). The vial was purged with N_2 for 3 times and then charged with a H_2 balloon. The mixture was stirred at room temperature for a certain time. After the completion of the reaction detected by TLC, saturated NH_4Cl aqueous was added and the reaction mixture was exacted with DCM (10 mL×3). The combined extracts were washed with brine, dried with Na_2SO_4, filtered and concentrated in vacuo. The residue was purified by column chromatograph over silica gel to afford **8**.[4]

8, 4-(2-aminonaphthalen-1-yl)-6,7,8,9-tetrahydro-2*H*-quinolizin-2-one

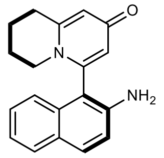

26 mg, 89% yield, white solid, m.p. 295-296 °C. $[\alpha]_D^{25}$ -19.6 (c = 0.5, $CHCl_3$). **^{1}H NMR (500 MHz, Chloroform-*d*) δ**: 7.91 (d, J = 9.0 Hz, 1H), 7.80 (dd, J = 8.2, 1.3 Hz, 1H), 7.41 (ddd, J = 8.4, 6.8, 1.3 Hz, 1H), 7.29 (ddd, J = 8.0, 6.8, 1.2 Hz, 1H), 7.17 (dd, J = 8.9, 2.2 Hz, 2H), 6.51 – 6.49 (m, 2H), δ 3.53 (ddd, J = 12.6, 7.2, 5.2 Hz, 1H), 3.45 (ddd, J = 13.6, 7.0, 5.3 Hz, 1H), 2.97 (s, 2H), 2.93 (t, J = 6.8 Hz, 2H), 1.89 (dt, J = 11.9, 6.7 Hz, 2H), 1.79 (td, J = 7.0, 5.3 Hz, 2H). **^{13}C NMR (126 MHz, Chloroform-*d*) δ**: 178.01, 150.73, 146.66, 142.36, 138.13, 131.60, 130.05, 127.71, 126.24, 125.77, 121.46, 120.10, 116.08, 112.98, 109.69, 46.47, 44.79, 27.79, 21.59. **HRMS(ESI) m/z**: calculated for $[C_{19}H_{18}N_2O + H]^+$ 291.1497, found 291.1489. **HPLC data**

(Chiralpak AD column, hexane : isopropanol = 70: 30, 1.0 mL/min): tr = 12.1 min (major), tr = 14.7 min (minor), ee = 99%.

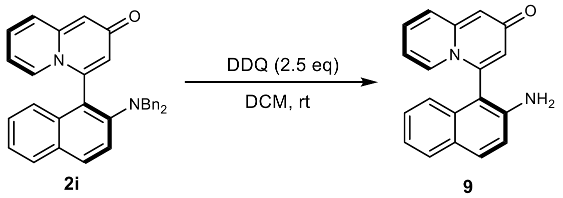

To a flame-dried round-bottom flask equipped with a magnetic stirring bar was added substrate **2i** (466 mg, 1.0 mmol, 1.0 eq), DDQ (270 mg, 2.5 mmol, 2.5 eq). The vial was then charged with DCM (10 mL) and stirred at room temperature for a certain time. After the completion of the reaction detected by TLC, saturated NH_4Cl aqueous was added and the reaction mixture was exacted with DCM (10 mL×3). The combined extracts were washed with brine, dried with Na_2SO_4, filtered and concentrated in vacuo. The residue was purified by column chromatograph over silica gel to afford **9**.

9, 4-(2-aminonaphthalen-1-yl)-2*H*-quinolizin-2-one

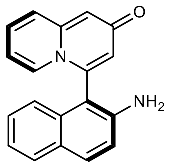

60.1 mg, 21% yield, white solid, m.p. 209-210 °C. $[\alpha]_D^{25}$ -25.9 (c = 0.5, $CHCl_3$). **^{1}H NMR (500 MHz, Chloroform-*d*) δ:** 7.81 (d, J = 8.8 Hz, 1H), 7.77 (d, J = 7.8 Hz, 1H), 7.40 (d, J = 7.2 Hz, 1H), 7.32 – 7.28 (m, 3H), 7.13 (t, J = 7.2 Hz, 2H), 7.01 – 6.98 (m, 2H), 6.75 (s, 1H), 6.40 (t, J = 6.9 Hz, 1H), 4.45 (s, 2H). **^{13}C NMR (126 MHz, Chloroform-*d*) δ**: 174.35, 144.68, 142.67, 141.11, 131.49, 130.82, 128.42, 128.33, 127.45, 126.92, 126.41, 125.34, 123.68, 121.82, 121.01, 117.54, 111.98, 110.49, 106.31. **HRMS(ESI) m/z**: calculated for $[C_{19}H_{14}N_2O + H]^+$ 287.1184, found 287.1179. **HPLC data** (Chiralpak IG column, hexane : isopropanol = 50: 50, 1.0 mL/min): tr = 17.0 min (major), tr = 11.1 min (minor), ee = 98%.

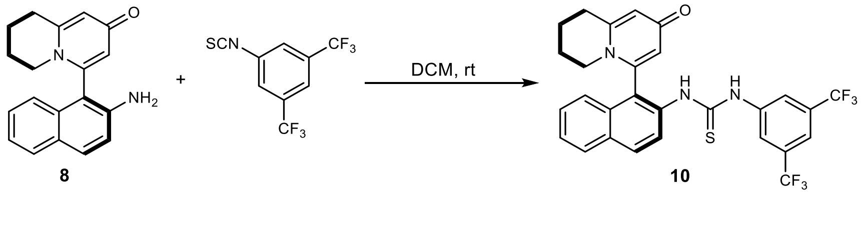

To a 2 dram scintillation vial equipped with a magnetic stirring bar was added substrate **8** (29.1 mg, 0.1 mmol, 1.0 eq), 3, 5-bis(trifluoromethyl)phenyl isothiocyanate (67.8 mg, 0.25 mmol, 2.5 eq). The vial was then charged with DCM (1.0 mL) and stirred at room temperature for a certain time. After the completion of the reaction detected by TLC, saturated NH_4Cl aqueous was added and the reaction mixture was exacted with DCM (10 mL×3). The combined extracts were washed with brine, dried with Na_2SO_4, filtered and concentrated in vacuo. The residue was purified by column chromatograph over silica gel to afford **10**.

10, 1-(3,5-bis(trifluoromethyl)phenyl)-3-(1-(2-oxo-6,7,8,9-tetrahydro-2*H* -quinolizin-4-yl) naphthalen-2-yl)thiourea

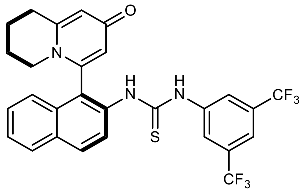

37.5 mg, 67 % yield, white solid, m.p. 138-139 °C. $[\alpha]_D^{25}$ +205.0 (*c* = 0.5, $CHCl_3$). **^{1}H NMR (500 MHz, Chloroform-*d*) δ**: 11.71 (s, 1H), 10.52 (s, 1H), 8.49 (s, 2H), 8.11 (d, *J* = 8.7 Hz, 1H), 8.01 (d, *J* = 8.9 Hz, 1H), 7.94 (d, *J* = 8.1 Hz, 1H), 7.63 (d, *J* = 14.4 Hz, 1H), 7.55 (t, *J* = 7.5 Hz, 1H), 7.49 (d, *J* = 8.2 Hz, 1H), 7.36 (d, *J* = 8.5 Hz, 1H), 6.49 (s, 1H), 6.38 (s, 1H), 3.95 (t, *J* = 10.1 Hz, 1H), 3.46 (dd, *J* = 13.5, 6.6 Hz, 1H), 2.93 (s, 2H), 1.89 – 1.75 (m, 4H). **^{13}C NMR (126 MHz, Chloroform-*d*) δ**: 179.86, 176.23, 151.94, 147.44, 140.73, 134.90, 130.96, 130.73, 130.70, 130.43, 128.07 (q, *J* = 128.07), 126.50, 125.64, 124.30, 123.47, 122.96, 121.30, 120.74, 119.89, 115.99, 115.54, 46.58, 27.91, 21.28, 17.12. **^{19}F NMR (470 MHz, Chloroform-*d*) δ**: -62.82. **HRMS(ESI) m/z**: calculated for $[C_{28}H_{21}F_6N_3OS+ Na]^+$ 584.1207, found 584.1199. **HPLC data** (Chiralpak OD column, hexane : isopropanol = 70: 30, 1.0 mL/min): tr = 4.1 min (major), tr = 6.5 min (minor), ee = 99%.

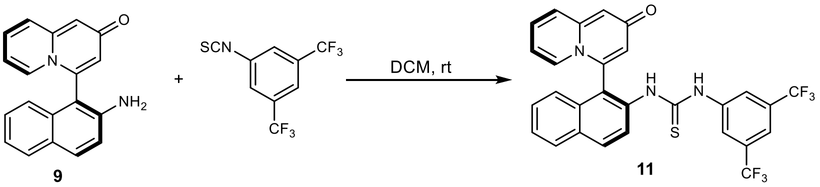

To a 2 dram scintillation vial equipped with a magnetic stirring bar was added substrate **9** (28.7 mg, 0.1 mmol, 1.0 eq), 3, 5-bis(trifluoromethyl)phenyl isothiocyanate (67.8 mg, 0.25 mmol,

2.5 eq). The vial was then charged with DCM (1.0 mL) and stirred at room temperature for a certain time. After the completion of the reaction detected by TLC, saturated NH_4Cl aqueous was added and the reaction mixture was exacted with DCM (10 mL×3). The combined extracts were washed with brine, dried with Na_2SO_4, filtered and concentrated in vacuo. The residue was purified by column chromatograph over silica gel to afford **11**.

11, 1-(3,5-bis(trifluoromethyl)phenyl)-3-(1-(2-oxo-2*H*-quinolizin-4-yl) naphthalen-2-yl)thiourea

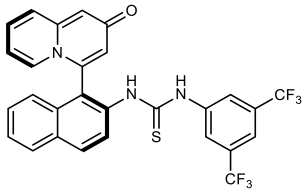

25.4 mg, 46% yield, white solid, m.p. 120-121 °C. $[\alpha]_D^{25}$ +57.0 (c = 0.1, $CHCl_3$). **^{1}H NMR (500 MHz, Chloroform-*d*) δ**: 11.52 (s, 1H), 10.59 (s, 1H), 8.36 (s, 2H), 8.21 (d, J = 8.9 Hz, 1H), 8.11 (d, J = 8.9 Hz, 1H), 7.99 (d, J = 8.2 Hz, 1H), 7.84 (d, J = 7.4 Hz, 1H), 7.63 (s, 1H), 7.57 (t, J = 7.6 Hz, 1H), 7.44 (t, J = 7.7 Hz, 1H), 7.34 (d, J = 4.4 Hz, 2H), 7.28 (d, J = 8.5 Hz, 1H), 6.98 (s, 1H), 6.65 (q, J = 3.9 Hz, 1H), 6.57 (s, 1H). **^{13}C NMR (126 MHz, Chloroform-*d*) δ:** 181.43, 173.86, 145.86, 142.10, 141.42, 141.36, 137.14, 132.08, 131.83 (q, J = 130.44), 131.12, 130.82, 130.47, 128.69, 127.84, 127.79, 126.72, 126.17, 124.42, 124.22, 123.71, 123.18, 122.26, 117.27, 114.41, 110.73. **^{19}F NMR (470 MHz, Chloroform-*d*) δ:** -62.83. **HRMS(ESI) m/z**: calculated for $[C_{28}H_{17}F_6N_3OS + H]^+$ 558.1075, found 558.1069. **HPLC data** (Chiralpak OD column, hexane : isopropanol = 70: 30, 1.0 mL/min): tr = 4.5 min (major), tr = 8.2 min (minor), ee = 97%.

Application of 10 for enantioselective Michael addition.

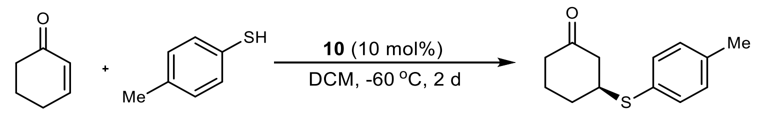

To a 2 dram scintillation vial equipped with a magnetic stirring bar was added cyclohex-2-en-1-one (9 mg, 0.1 mmol, 1.0 eq), catalyst **10** (5.8 mg, 0.01 mmol, 10 mol%) and DCM (1.0 mL). The vial was cooled to -60 °C. After the reaction was stirred at this temperature for 10 min, 4-methylbenzenethiol (24.8 mg, 0.2 mmol, 2.0 eq) was added and the mixture was stirred at the same temperature for 2 days. Then the saturated NH_4Cl aqueous was added to quench the reaction. The reaction mixture was warmed up to room temperature and exacted with DCM (10 mL×3). The combined extracts were washed with brine, dried with Na_2SO_4, filtered and

concentrated in vacuo. The residue was purified by column chromatograph over silica gel to afford the desired product.[5] 16.5 mg, 76% yield. **HPLC data** (Daicel Chiralpak AD column, hexane : isopropanol = 90: 10, 1.0 mL/min): tr = 6.3 min (major), tr = 7.1 min (minor), ee = 39%.

Supplementary Discussion

^{1}H NMR and ^{31}P NMR spectrum of 1b and CPA

Considering the co-existence of the *enol* and *keto* equivalent of **1b**, we investigated the interactions of (*R*)-CPA and **1b** by ^{1}H-NMR and ^{31}P-NMR. As can be seen from Supplementary Fig. 7, the original ratio of *enol/keto* was calculated as 2.5:1 by ^{1}H-NMR spectroscopy, and the H_a and H_b signals were recorded as δ 4.31 and δ 5.99. In the case of addition of (*R*)-CPA (Supplementary Fig. 8), the ratio of *enol/keto* was decreased to 1.4/1. Moreover, the mixture of (*R*)-CPA (1.0 equiv) and **1b** (1.0 equiv) exhibited, two signals (δ 4.50 and δ 5.92) for H_a and H_b respectively, at a significantly different chemical shift compared to **1b** in the absence of the CPA additive. The interactions were further confirmed by ^{31}P-NMR studies in DMSO-*d6* (Supplementary Fig. 9 and Fig. 10). The chemical shift of (*R*)-CPA is changed from 2.68 to 3.47 in the presence of **1b**.

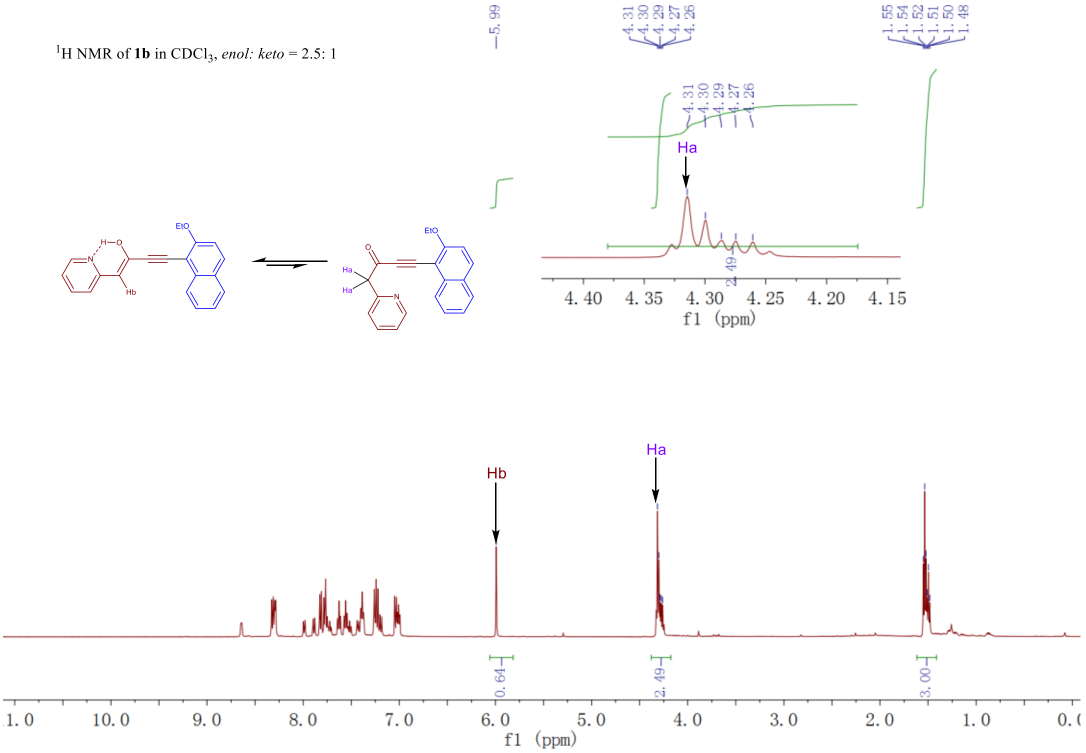

Supplementary Fig. 7. ^{1}H NMR spectrum of 1b.

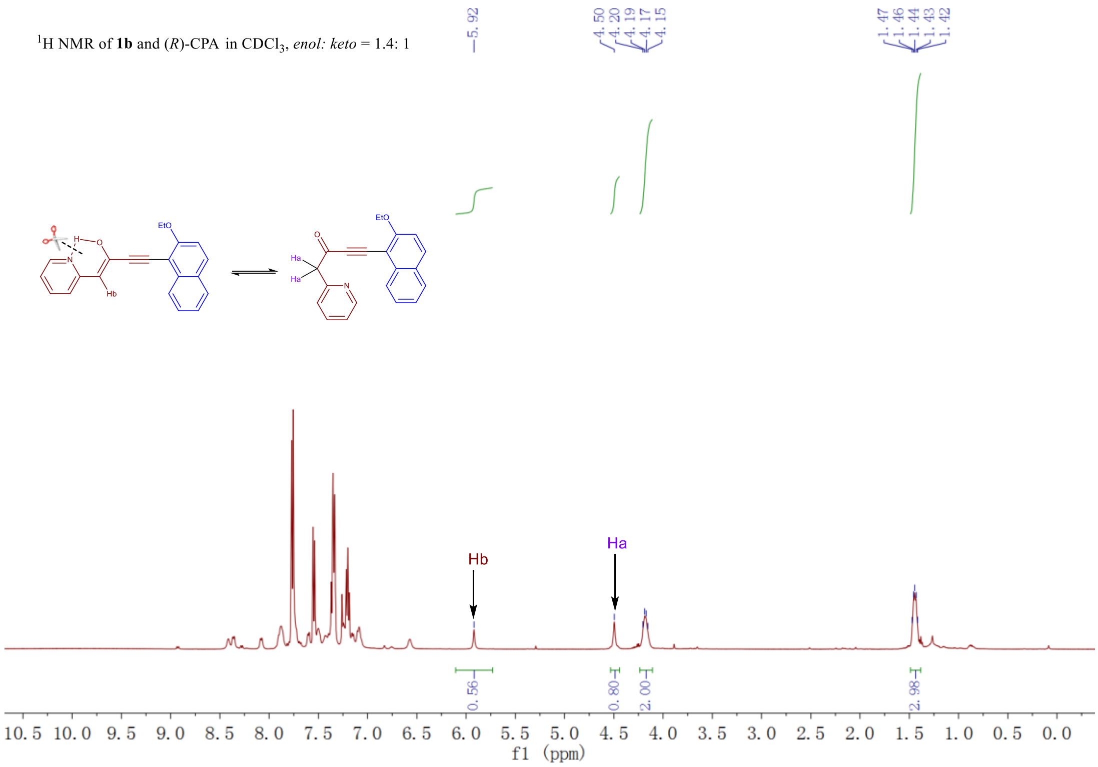

Supplementary Fig. 8. ^{1}H NMR spectrum of 1b and (*R*)-CPA.

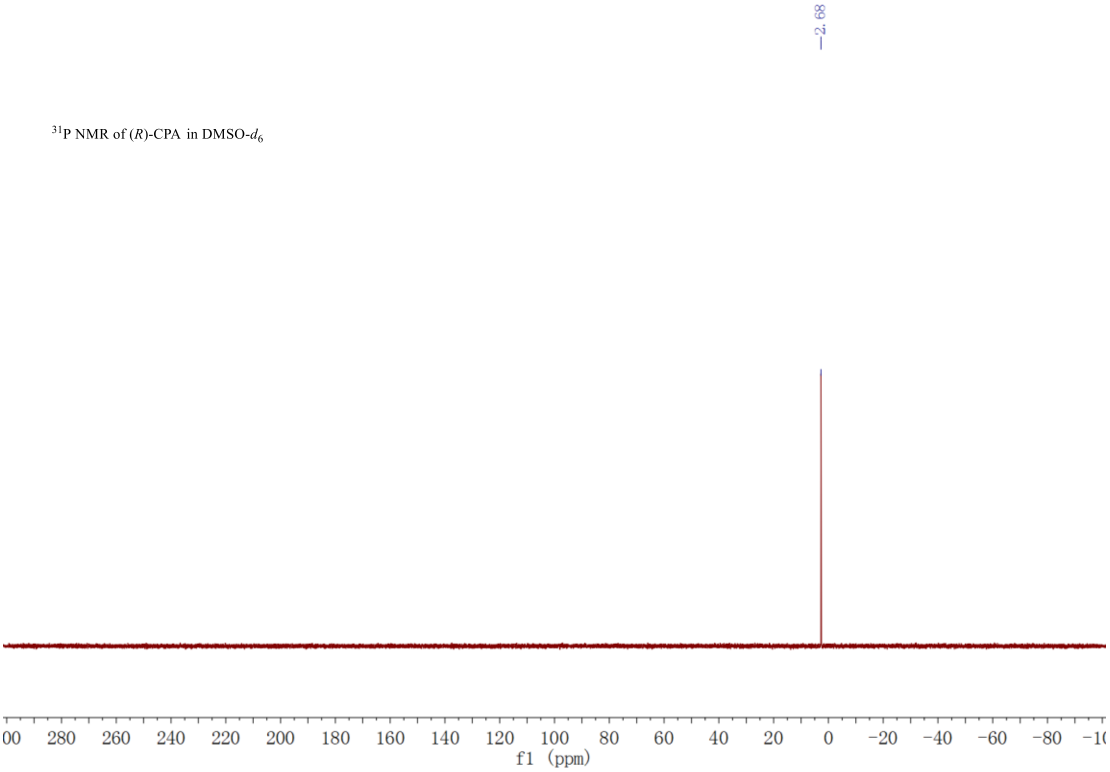

Supplementary Fig. 9. ^{31}P NMR spectrum of (*R*)-CPA.

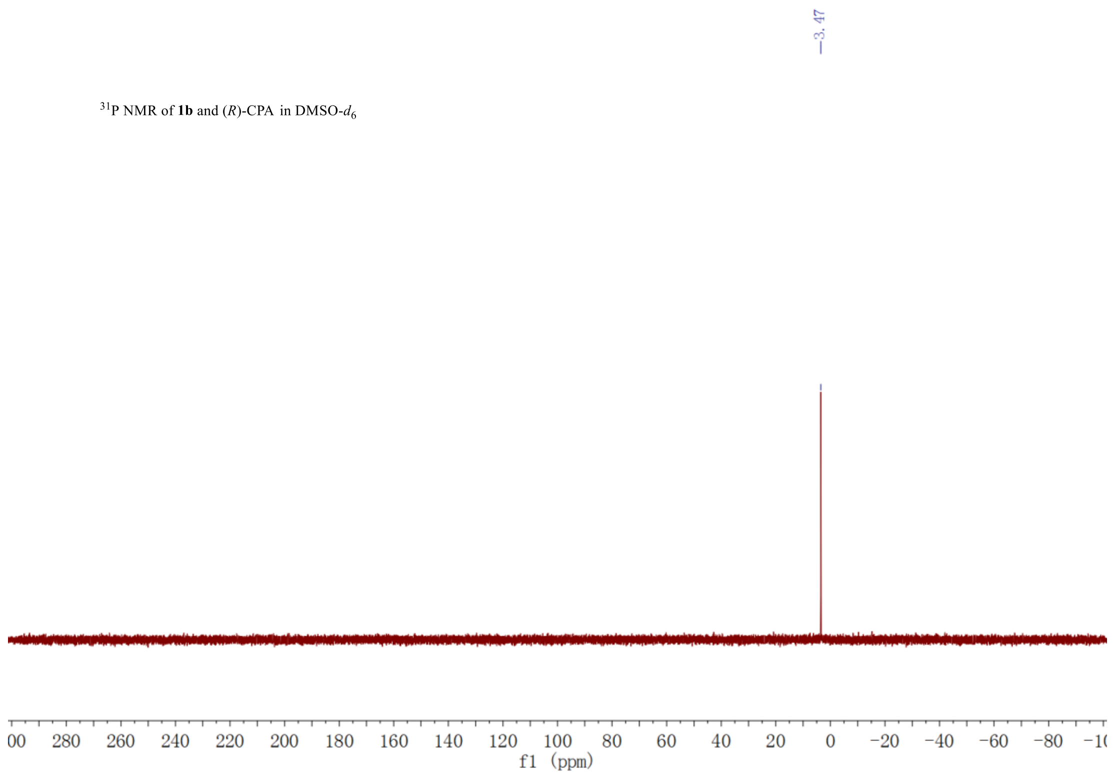

Supplementary Fig. 10. ^{31}P NMR spectrum of 1b and (*R*)-CPA.

Dynamic ^{1}H NMR spectrum of 1k and CPA

In order to study the ratio of *keto* and *enol* during the reaction, substrate **1k** was taken into consideration in $CDCl_3$ since the distinguished characteristic peaks between *keto* and ligand. The ratio of *keto* and *enol* during the reaction is unchanged from the dynamic ^{1}H NMR of the reaction. With the copper catalyst in the reaction system, the proton signals of CH_2 in *keto* moiety were recorded from δ 4.42 to δ 4.28 which indicated the direction of the carbonyl and copper catalyst. Those observations further strengthened our mechanism illustrated in Fig.6 of manuscript.

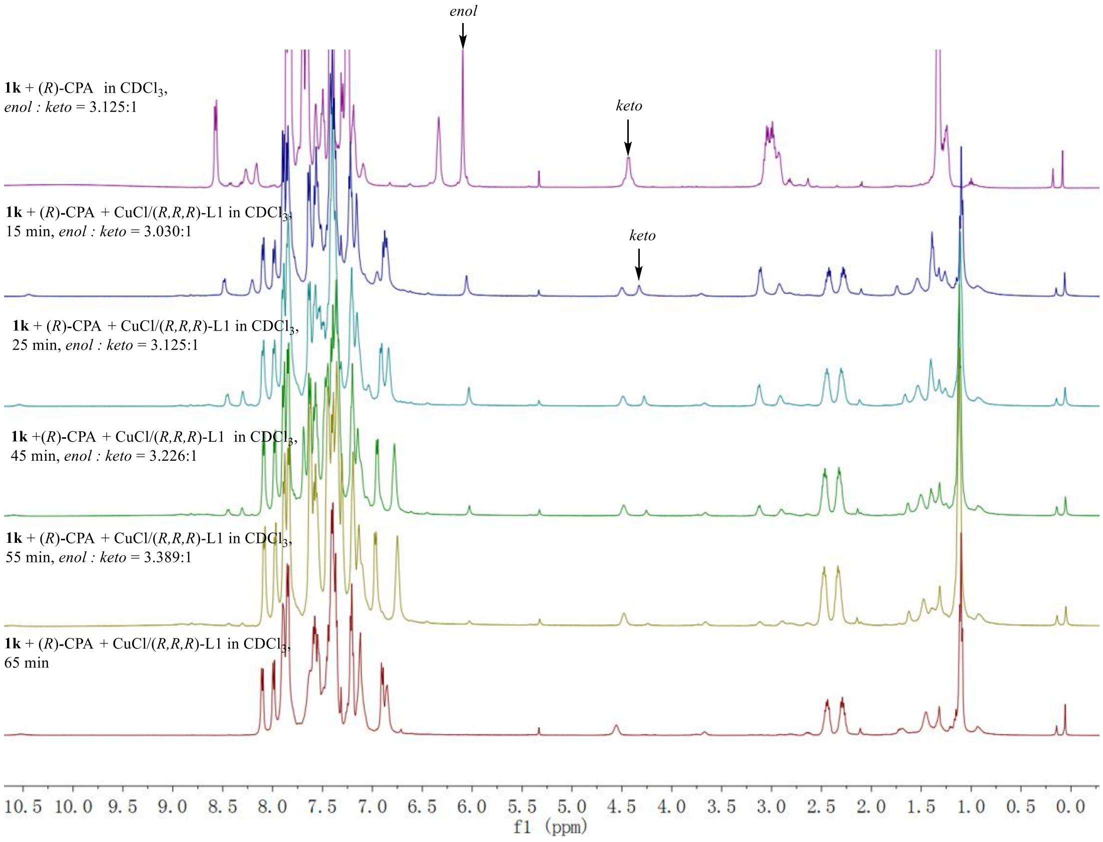

Supplementary Fig. 11. Dynamic ^{1}H NMR spectrum of 1k and (*R*)-CPA.

Linear effect of ligand and product

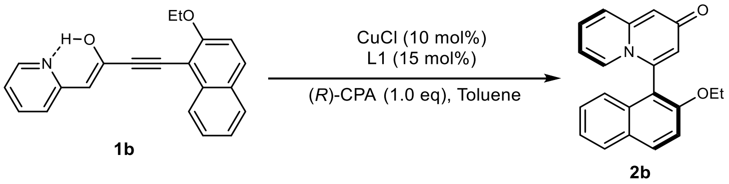

To a vial was added CuCl (1.0 mg, 10 mol%), L1 (8.5 mg, 15 mol%), toluene (2.0 mL) and stirring bar. The vial was wrapped with Teflon tape and fitted with corresponding cap. The vial was stirred at room temperature for 30 min. Then the corresponding substrate **1b** (31.5 mg, 0.1 mmol. 1.0 eq) and chiral phosphoric acid (34.8 mg, 0.1 mmol, 1.0 eq) were added. The reaction was stirred at 20 °C for 36 h. Then, the mixture was diluted with EA and washed with water for three times. The extracts were dried and concentrated in vacuo. The residue was purified by column chromatograph over silica gel. The ee values were determined by chiral HPLC.

ee of L1	0	24.0	37.0	54.7	77.0	>99
ee of Product **2b**	0	15.3	30.9	49.48	68.9	92.0

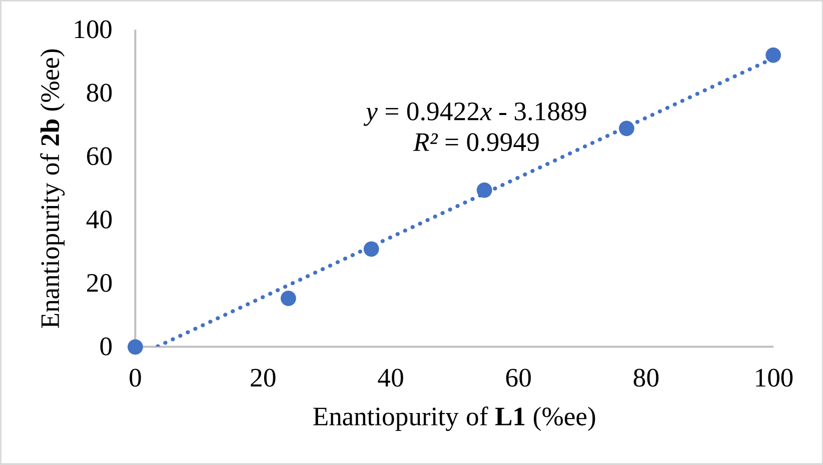

Supplementary Fig. 12. Linear effect of L1 and 2b.

The linear free energy relationship analysis [a]

Supplementary Table 4. Literature values for the substituents used in this reaction [6-10]

Entry	R	er	Charton value	B1	B5	L	σ	$\Delta\Delta G^{\neq}(er)$ (kcal/mol)
1	OMe	88.06:11.94	0.36	-	-	-	-0.23	1.16
2	OEt	96.0:4.0	0.48	-	-	-	-0.24	1.85
3	Ph	96.52:3.48	0.57	1.71	3.11	6.28	-0.01	1.93
4	Me	92.42:7.58	0.52	1.52	2.04	2.87	-0.17	1.46
5	Et	94.02:5.98	0.56	1.74	3.31	4.42	-0.15	1.60
6	^{n}Bu	95.45:4.55	0.68	1.73	3.39	5.35	-0.16	1.77
7	^{i}Pr	96.66:3.34	0.76	2.05	3.35	4.36	-0.12	1.96
8	Cl	18.32:81.68	0.55	1.73	1.73	3.47	0.24	0.87
9	Br	16.95:83.05	0.65	1.95	1.95	3.85	0.23	0.93
mean			0.57	1.38	2.09	3.39	-0.07	1.50
std.			0.1172	0.79	1.35	2.16	0.18	0.42

[a] $\Delta\Delta G^{\neq} = RTln(er)$, R = 0.001986 kcal K^{-1} mol^{-1}, T = 293.15 K.

For the purposes of comparison, the selected values were normalized according to this equation:

$$X_N = (X - X_{mean})/S_X$$

Where X_N is the normalized value, X is the value selected from the literature, X_{mean} is the mean for the range of X, and S_X is the standard derivation for the range in X. Supplementary Table 5 was obtained according to this equation.

Supplementary Table 5. Normalized values for the substituents used in this reaction[a]

Entry	R	er	Charton value	B1	B5	L	σ	*ΔΔG*$^{\neq}$*(er)* (kcal/mol)
1	OMe	88.06:11.94	-1.79	-	-	-	-0.88	1.16
2	OEt	96.0:4.0	-0.77	-	-	-	-0.94	1.85
3	Ph	96.52:3.48	0	0.41	0.75	1.33	0.31	1.93
4	Me	92.42:7.58	-0.43	0.17	-0.04	-0.24	-0.56	1.46
5	Et	94.02:5.98	-0.08	0.45	0.89	0.47	-0.45	1.60
6	^{n}Bu	95.45:4.55	0.94	0.44	0.96	0.89	-0.50	1.77
7	^{i}Pr	96.66:3.34	1.62	0.84	0.92	0.44	-0.28	1.96
8	Cl	18.32:81.68	-0.17	0.43	-0.27	0.03	1.66	0.87
9	Br	16.95:83.05	0.68	0.71	-0.11	0.21	1.63	0.93

[a]$\Delta\Delta G^{\neq} = RTln(er)$, R = 0.001986 kcal K^{-1} mol^{-1}, T = 293.15 K.

The steric factor and electronic effect were supposed to affect the enantioselectivity simultaneously. We choose Hammett parameter to describe the electronic effect. In addition, Charton values and Sterimol parameter was used to describe the steric factor. However, after a stepwise regression analysis, we can't develop an appropriate model to elucidate stereoselectivity trends. And this mainly attribute to the fact that Hammett parameter was used to describe the electronic effect when the substituent was located at *para*-or *meta*-position, it can't elucidate the electronic effect when the substituent was located at *ortho*-position.

Supplementary Table 6. Single Crystal of X-ray Analysis of 2h·H_2O.

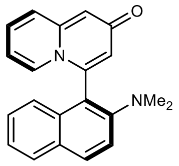

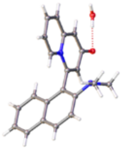

Identification code	**2h·H_2O**
Empirical formula	$C_{21}H_{20}N_2O_2$
Formula weight	332.39
Temperature/K	296.15
Crystal system	orthorhombic
Space group	$P2_12_12_1$
a/Å	8.7709(9)
b/Å	13.1001(14)
c/Å	15.5884(17)
α/°	90
β/°	90
γ/°	90
Volume/Å^3	1791.1(3)
Z	4
ρ_{calc}g/cm^3	1.233
μ/mm^{-1}	0.638
F(000)	704.0
Crystal size/mm^3	0.12 × 0.11 × 0.08
Radiation	CuKα (λ = 1.54178)
2Θ range for data collection/°	8.816 to 137.78
Index ranges	$-10 \leq h \leq 10$, $-15 \leq k \leq 15$, $-17 \leq l \leq 18$
Reflections collected	13749
Independent reflections	3298 [R_{int} = 0.0396, R_{sigma} = 0.0325]
Data/restraints/parameters	3298/0/231
Goodness-of-fit on F^2	1.097
Final R indexes [I>=2σ (I)]	R_1 = 0.0315, wR_2 = 0.0812
Final R indexes [all data]	R_1 = 0.0328, wR_2 = 0.0813
Largest diff. peak/hole / e Å^{-3}	0.11/-0.19
Flack parameter	0.04(8)

Spectral data

1a, 4-(2-methoxynaphthalen-1-yl)-1-(pyridin-2-yl)but-3-yn-2-one / (*Z*)-4-(2-methoxynaphthalen-1-yl)-1-(pyridin-2-yl)but-1-en-3-yn-2-ol

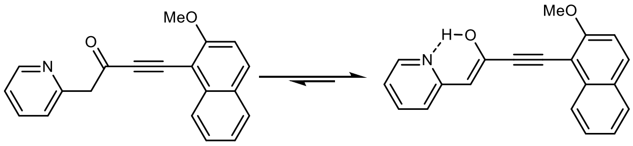

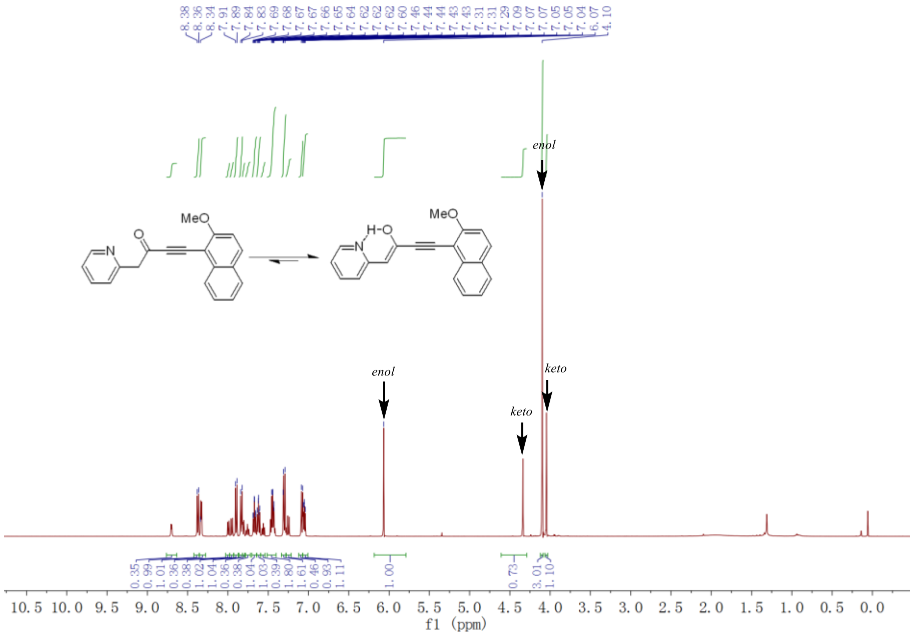

Supplementary Fig. 13. ^{1}H NMR spectrum of 1a.

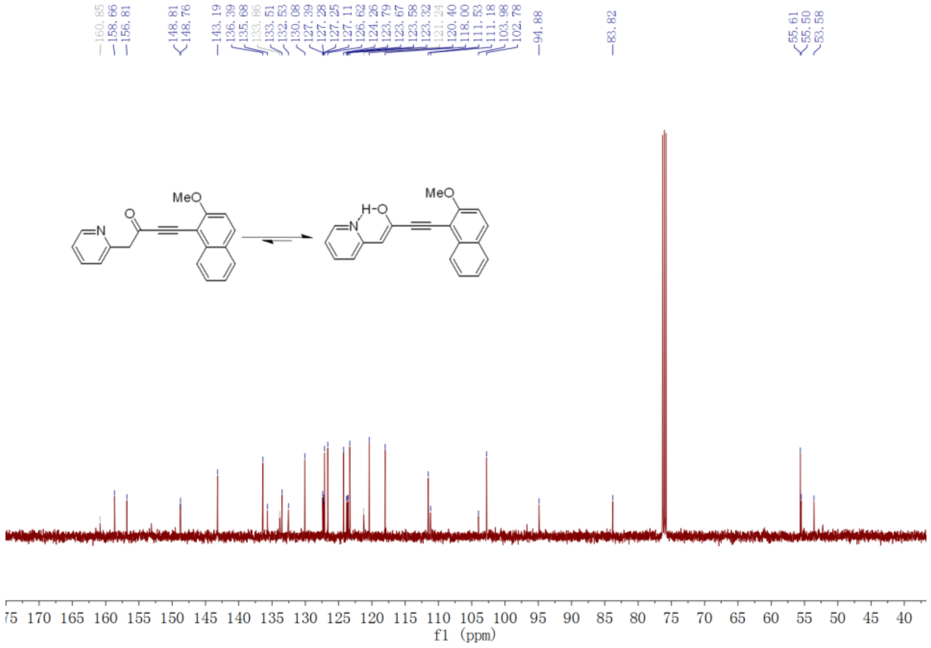

Supplementary Fig. 14. ^{13}C NMR spectrum of 1a.

1b, 4-(2-ethoxynaphthalen-1-yl)-1-(pyridin-2-yl)but-3-yn-2-one / (*Z*)-4-(2-ethoxynaphthalen-1-yl) -1-(pyridin-2-yl)but-1-en-3-yn-2-ol

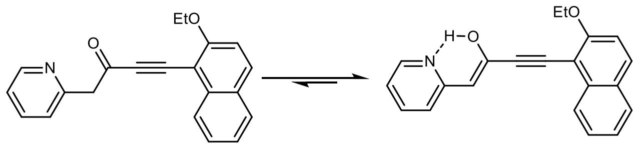

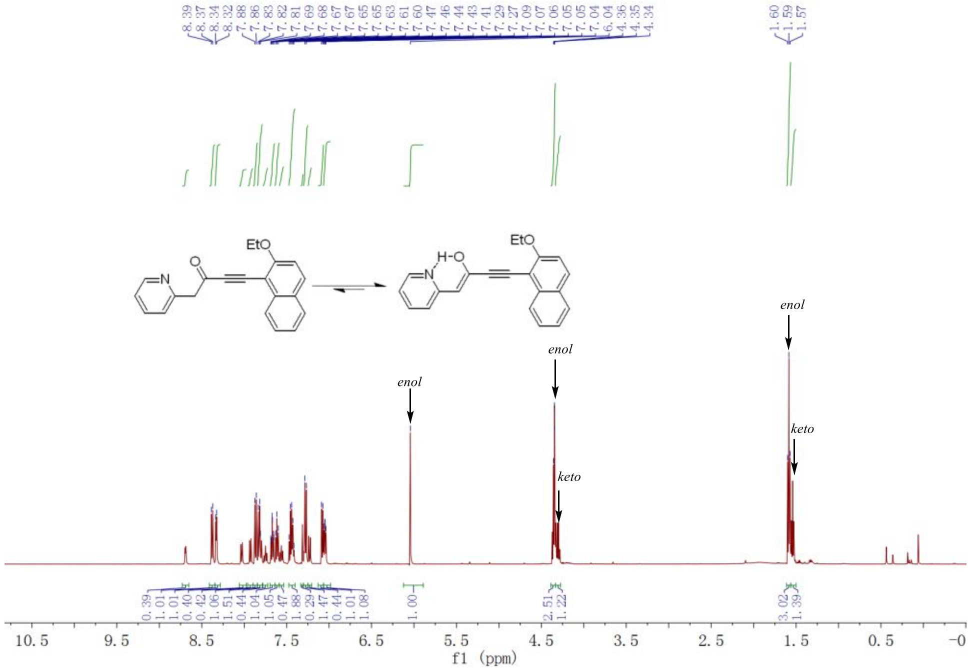

Supplementary Fig. 15. ^{1}H NMR spectrum of 1b.

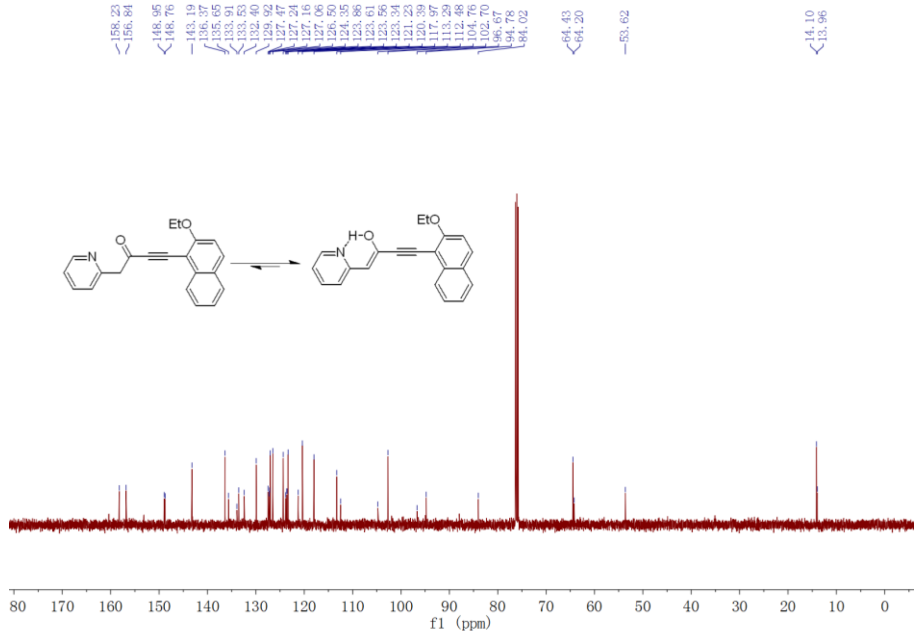

Supplementary Fig. 16. ^{13}C NMR spectrum of 1b.

1c, 4-(6-bromo-2-ethoxynaphthalen-1-yl)-1-(pyridin-2-yl)but-3-yn-2-one / (*Z*)-4-(6-bromo-2-ethoxynaphthalen-1-yl)-1-(pyridin-2-yl)but-1-en-3 -yn-2-ol.

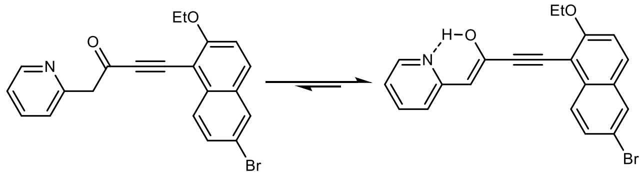

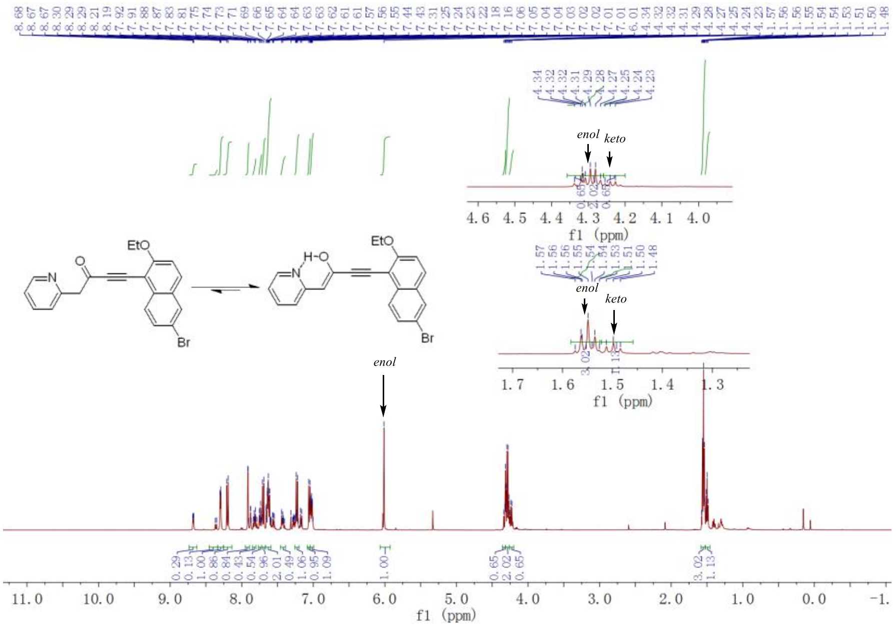

Supplementary Fig. 17. ^{1}H NMR spectrum of 1c.

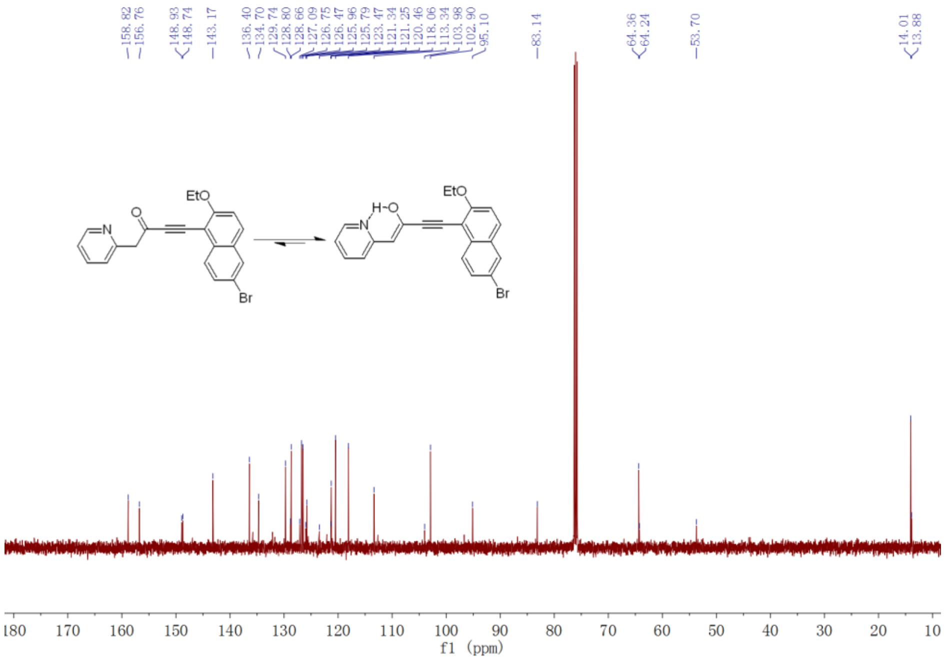

Supplementary Fig. 18. ^{13}C NMR spectrum of 1c.

1d, 4-(7-bromo-2-ethoxynaphthalen-1-yl)-1-(pyridin-2-yl)but-3-yn-2-one / (*Z*)-4-(7-bromo-2-ethoxynaphthalen-1-yl)-1-(pyridin-2-yl)but-1-en-3-yn-2-ol

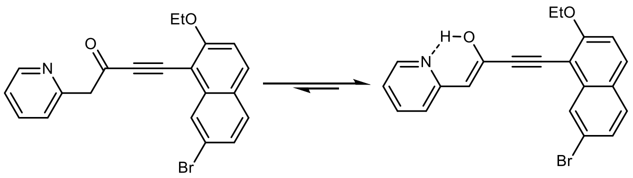

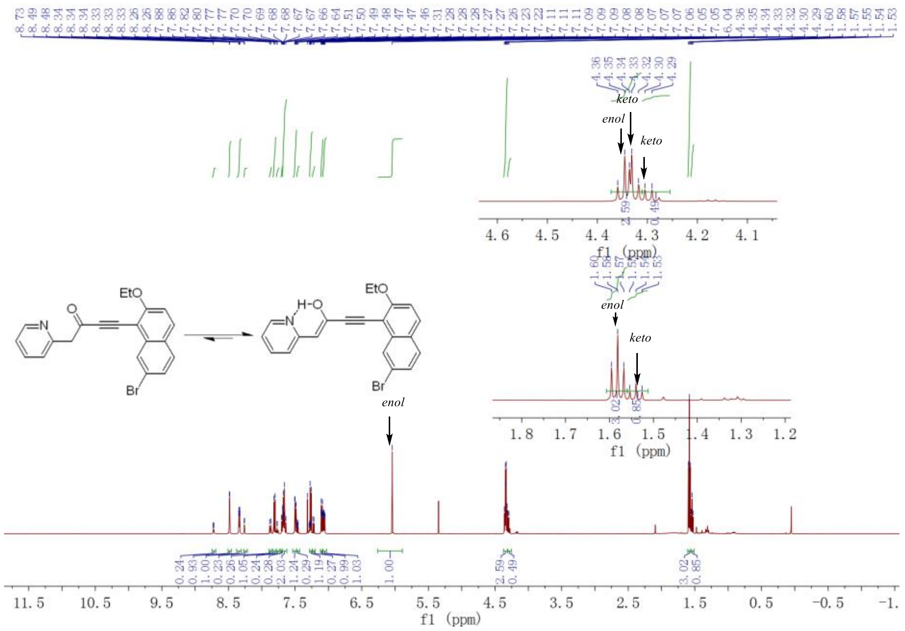

Supplementary Fig. 19. ^{1}H NMR spectrum of 1d.

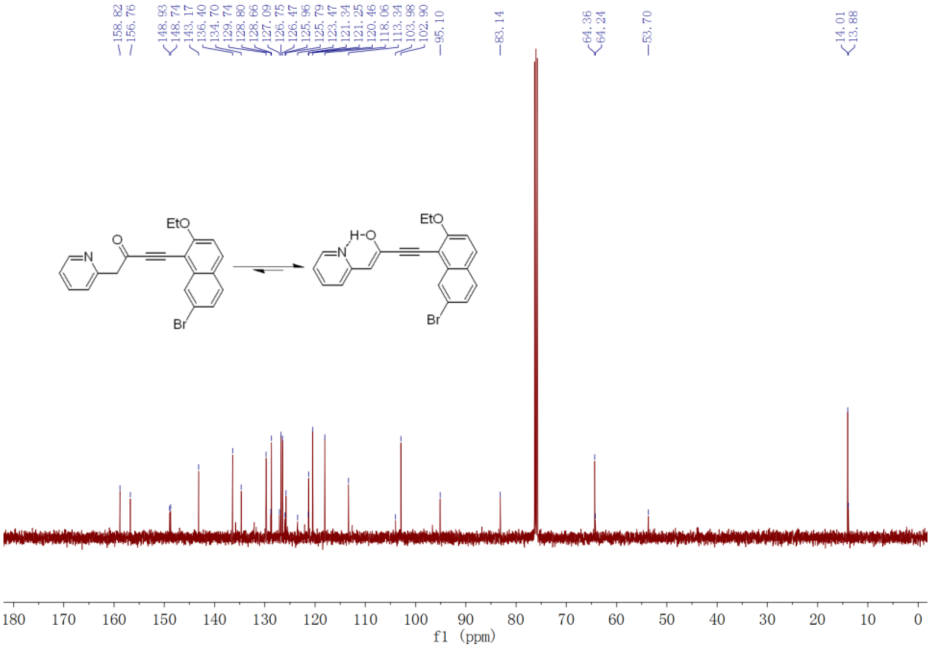

Supplementary Fig. 20. ^{13}C NMR spectrum of 1d.

1e, 4-(2-ethoxy-7-phenylnaphthalen-1-yl)-1-(pyridin-2-yl)but-3-yn-2-one / (*Z*)-4-(2-ethoxy-7-phenylnaphthalen-1-yl)-1-(pyridin-2-yl)but-1-en-3-yn-2-ol

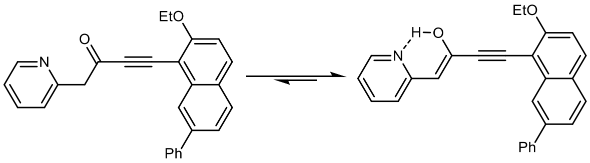

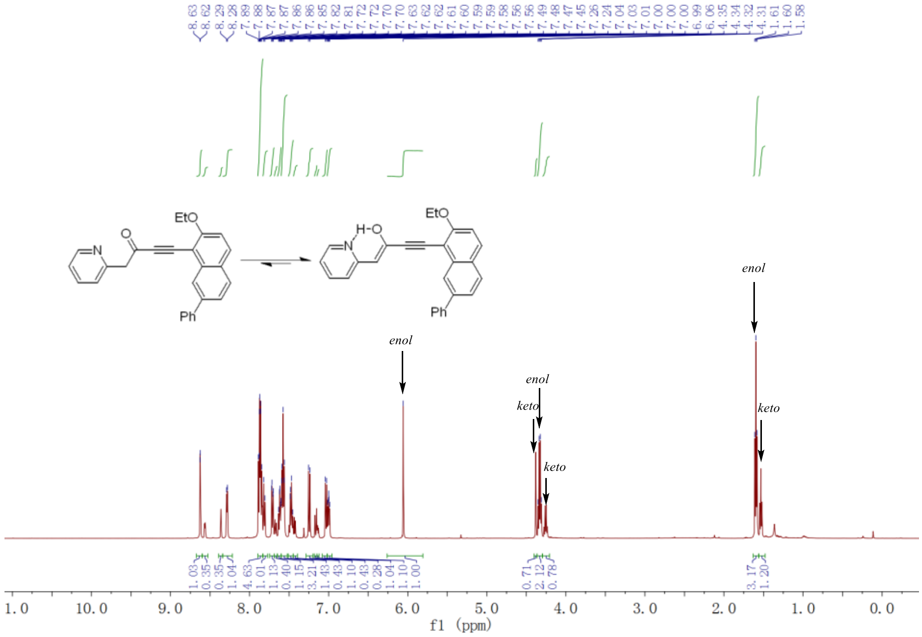

Supplementary Fig. 21. ^{1}H NMR spectrum of 1e.

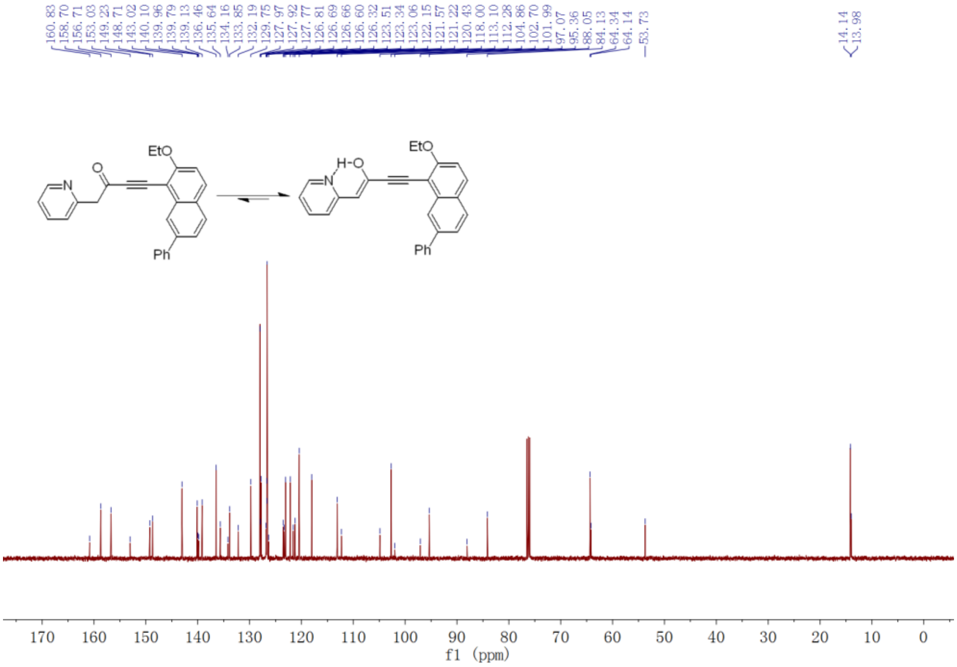

Supplementary Fig. 22. ^{13}C NMR spectrum of 1e.

1f, 4-(2-ethoxy-7-(phenylethynyl)naphthalen-1-yl)-1-(pyridin-2-yl)but-3-yn-2-one / (*Z*)-4-(2-ethoxy-7-(phenylethynyl)naphthalen-1-yl)-1-(pyridin-2-yl)but-1-en-3-yn-2-ol

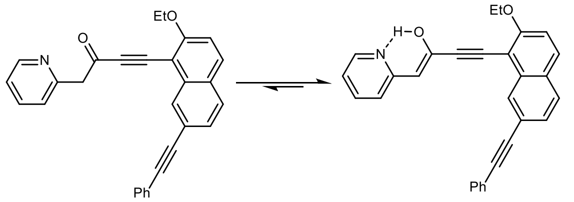

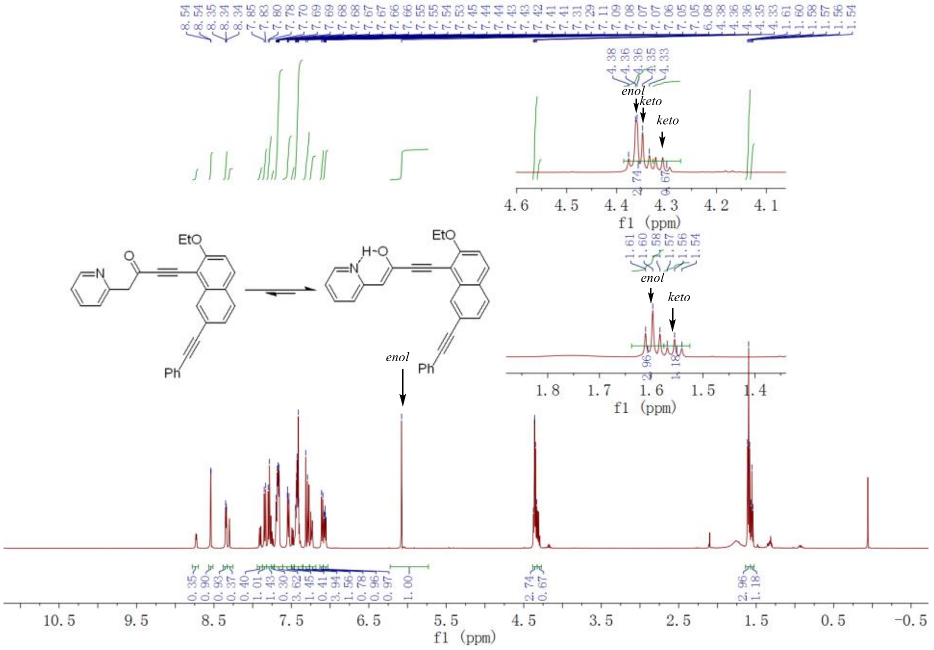

Supplementary Fig. 23. ^{1}H NMR spectrum of 1f.

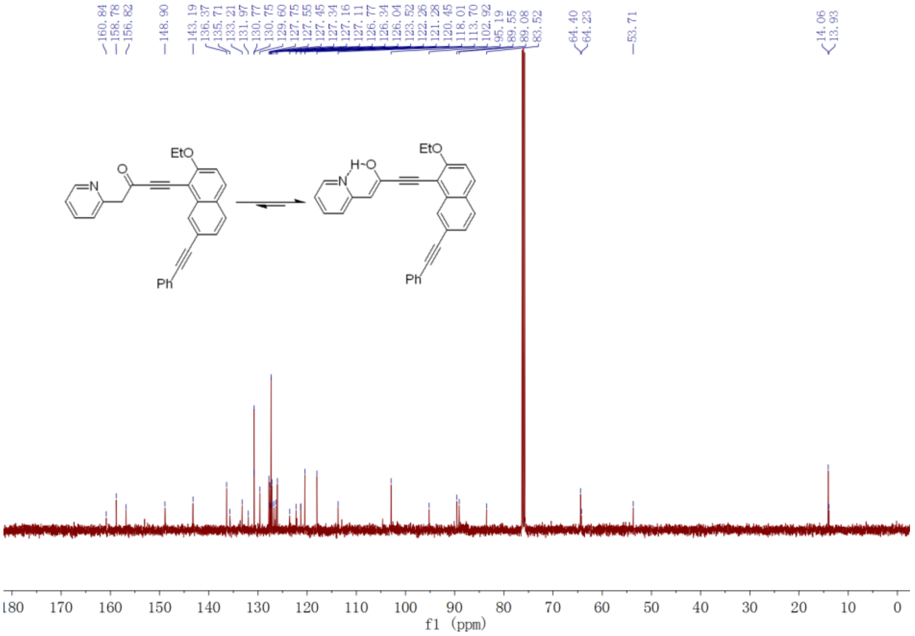

Supplementary Fig. 24. ^{13}C NMR spectrum of 1f.

1g, 4-(2-((4-methoxybenzyl)oxy)naphthalen-1-yl)-1-(pyridin-2-yl)but-3-yn-2-one / (*Z*)-4-(2-((4-methoxybenzyl)oxy)naphthalen-1-yl)-1-(pyridin-2-yl)but -1-en-3-yn-2-ol.

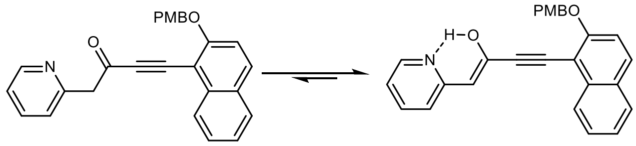

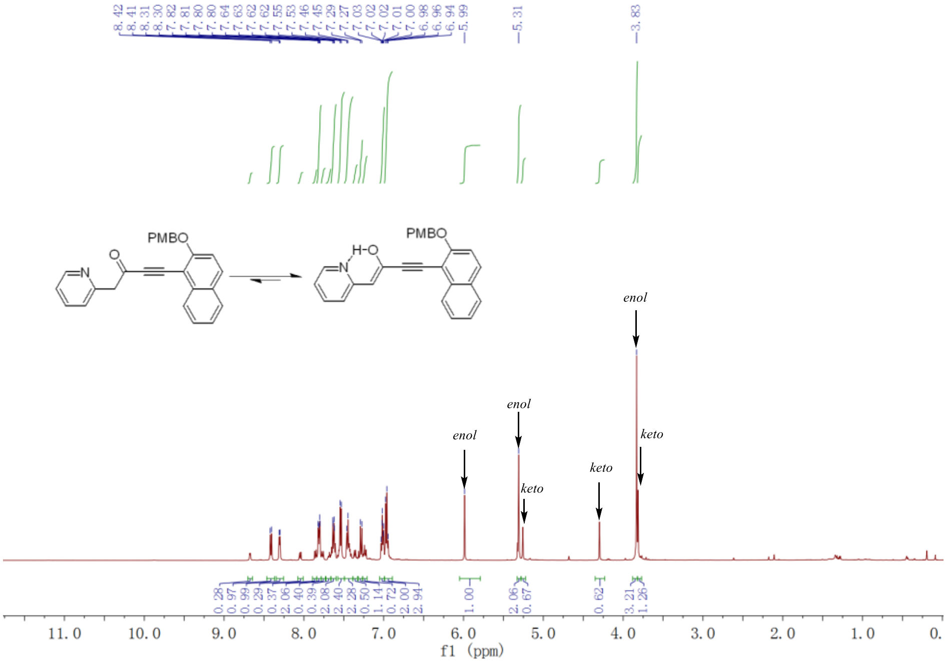

Supplementary Fig. 25. ^{1}H NMR spectrum of 1g.

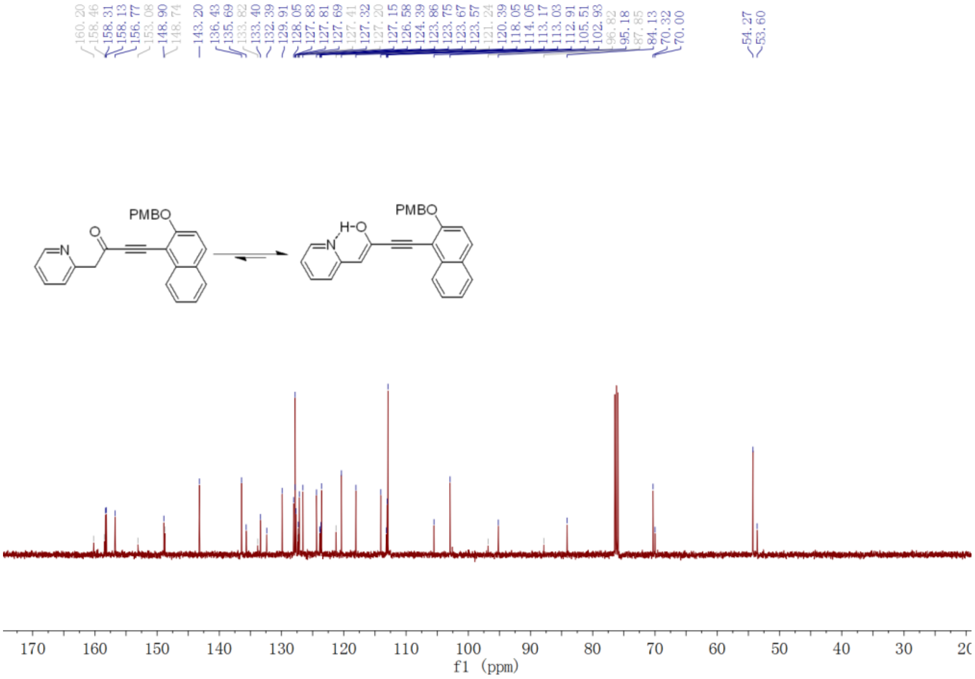

Supplementary Fig. 26. ^{13}C NMR spectrum of 1g.

1h, 4-(2-(dimethylamino)naphthalen-1-yl)-1-(pyridin-2-yl)but-3-yn-2-one / (*Z*)-4-(2-(dimethyl amino)naphthalen-1-yl)-1-(pyridin-2-yl)but-1-en-3-yn-2-ol

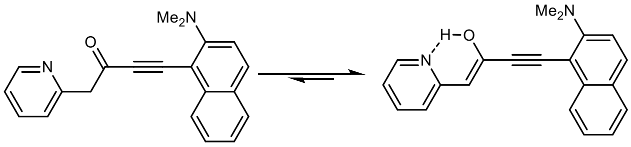

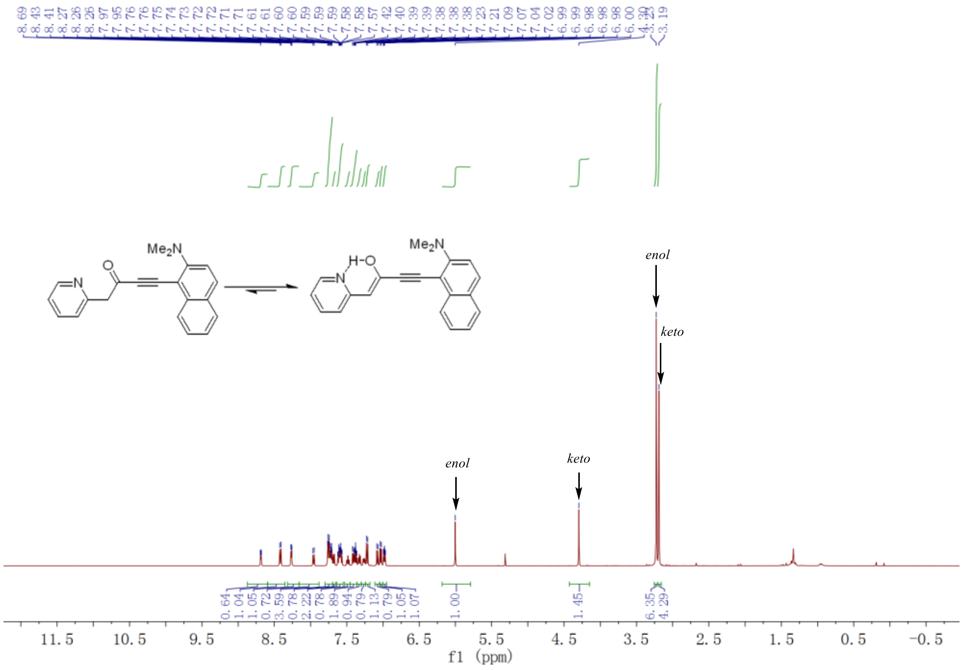

Supplementary Fig. 27. ^{1}H NMR spectrum of 1h.

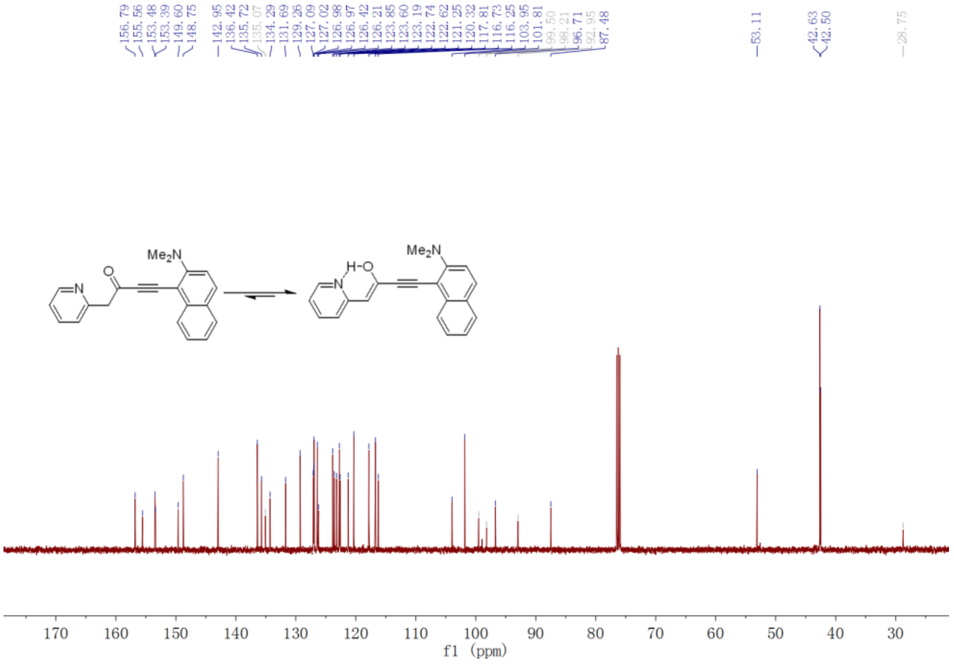

Supplementary Fig. 28. ^{13}C NMR spectrum of 1h.

1i, 4-(2-(dibenzylamino)naphthalen-1-yl)-1-(pyridin-2-yl)but-3-yn-2-one / (*Z*)-4-(2-(dibenzyl amino)naphthalen-1-yl)-1-(pyridin-2-yl)but-1-en-3-yn-2-ol

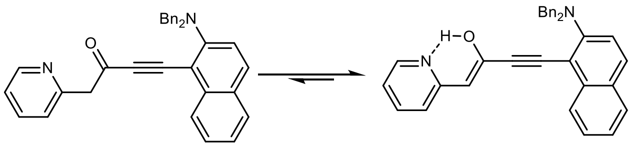

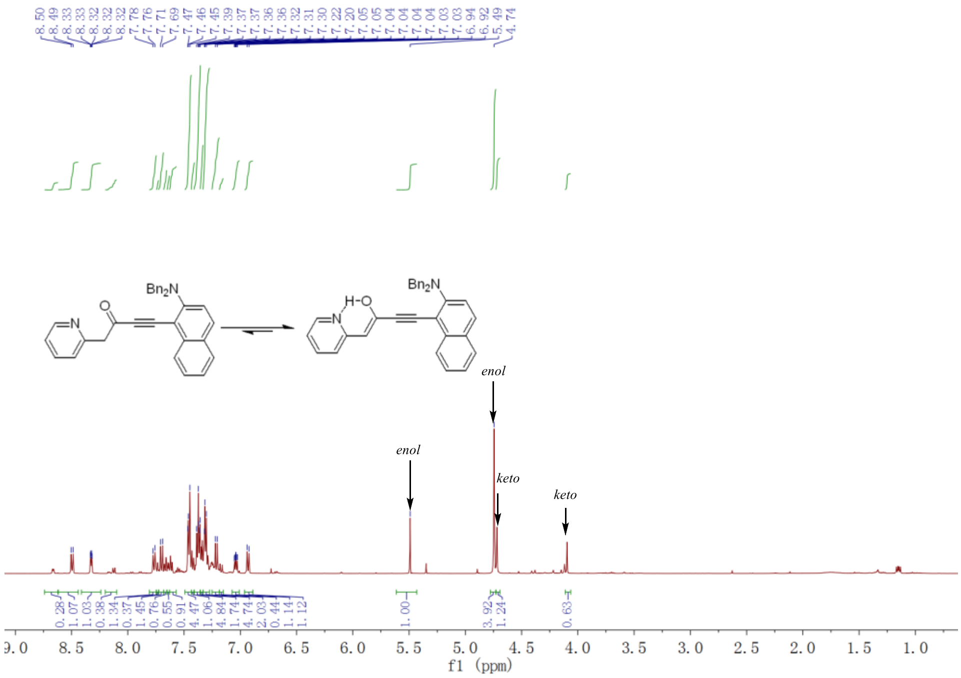

Supplementary Fig. 29. ^{1}**H NMR spectrum of 1i.**

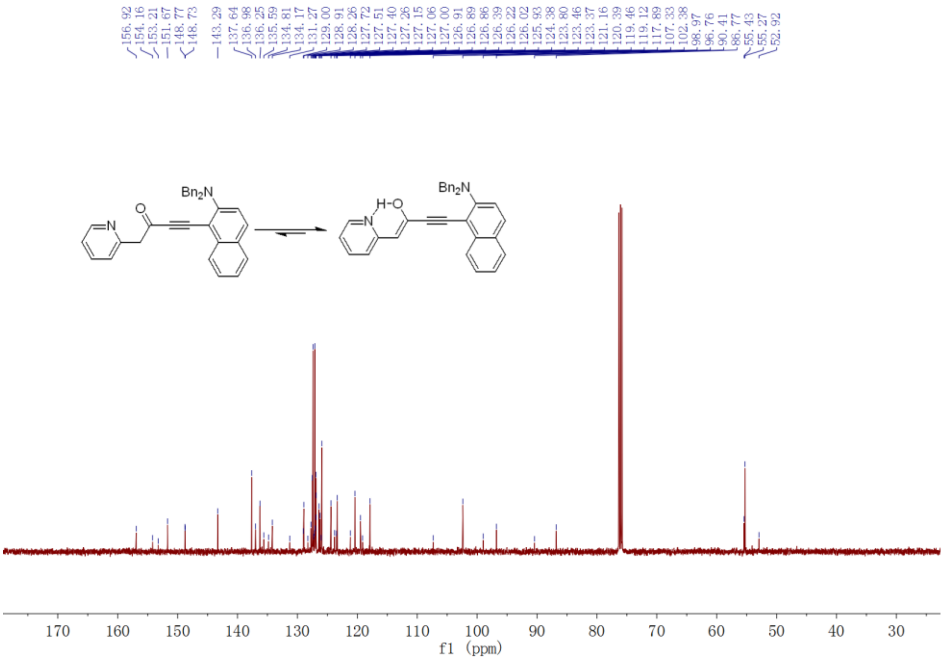

Supplementary Fig. 30. ^{13}**C NMR spectrum of 1i.**

1j, 4-(2-methylnaphthalen-1-yl)-1-(pyridin-2-yl)but-3-yn-2-one / (Z)-4-(2-methylnaphthalen-1-yl)-1-(pyridin-2-yl)but-1-en-3-yn-2-ol

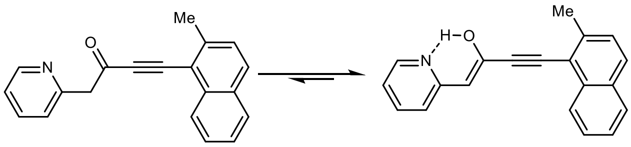

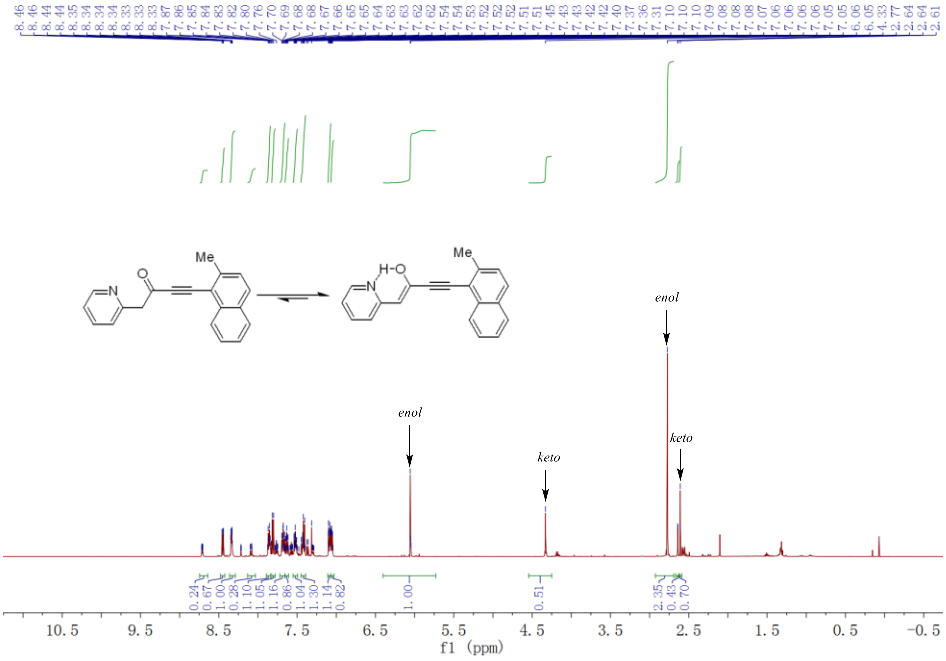

Supplementary Fig. 31. ^{1}H NMR spectrum of 1j.

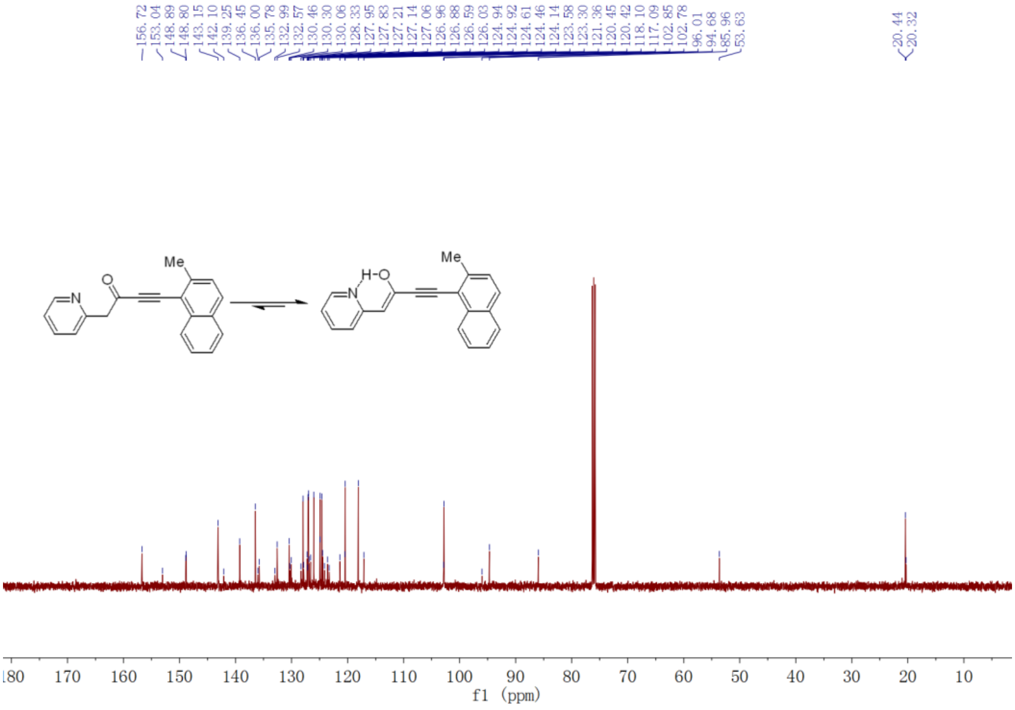

Supplementary Fig. 32. ^{13}C NMR spectrum of 1j.

1k, 4-(2-ethylnaphthalen-1-yl)-1-(pyridin-2-yl)but-3-yn-2-one / (*Z*)-4-(2-ethylnaphthalen-1-yl)-1-(pyridin-2-yl)but-1-en-3-yn-2-ol

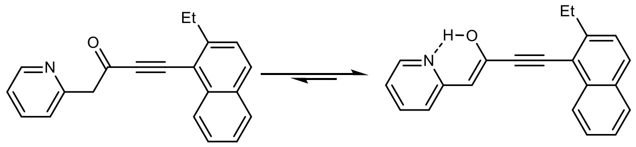

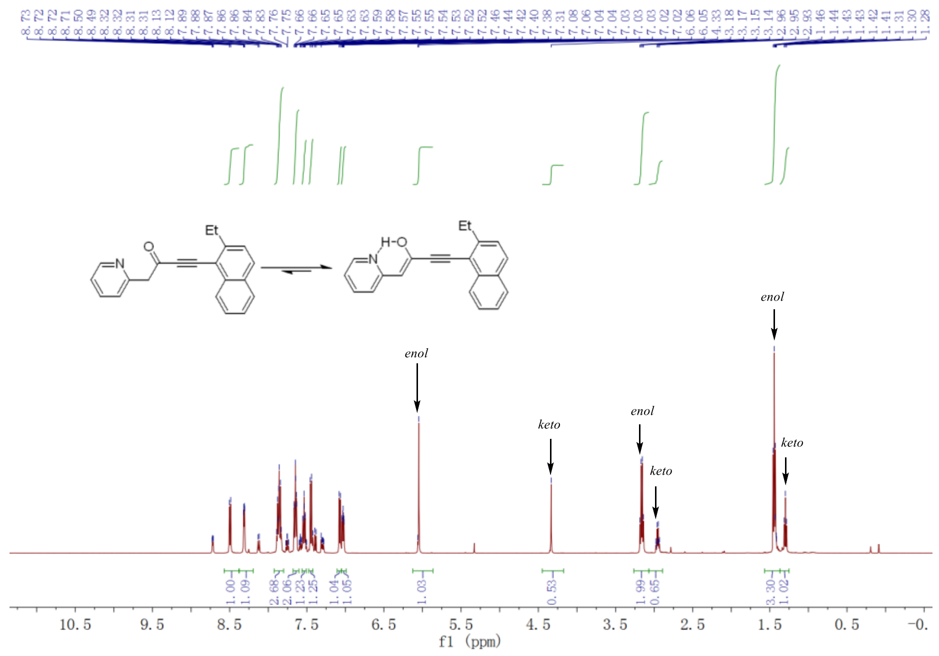

Supplementary Fig. 33. ^{1}H NMR spectrum of 1k.

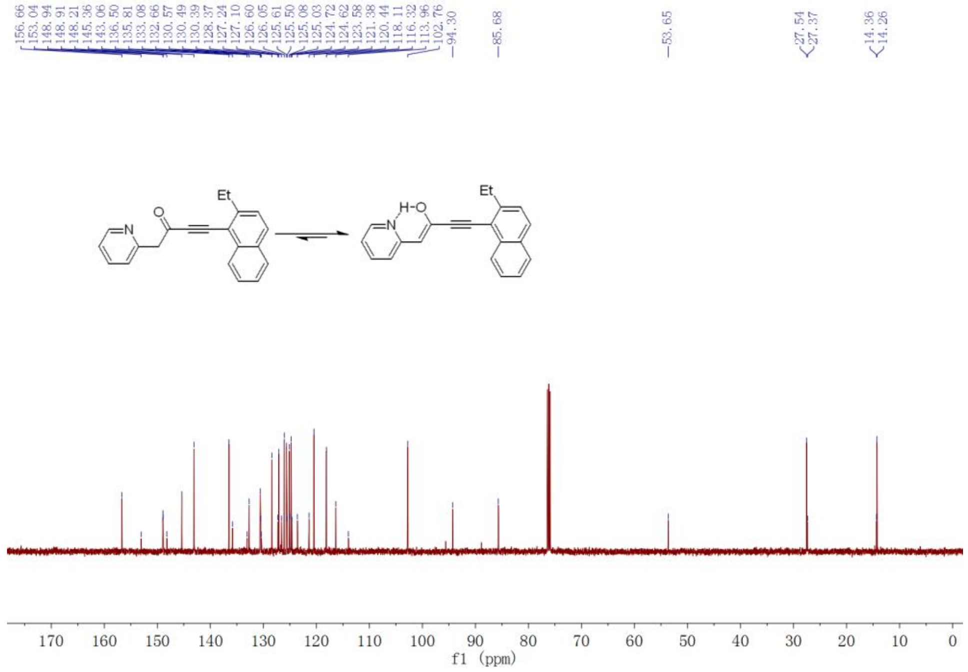

Supplementary Fig. 34. ^{13}C NMR spectrum of 1k.

1l, 4-(2-butylnaphthalen-1-yl)-1-(pyridin-2-yl)but-3-yn-2-one / (*Z*)-4-(2-butylnaphthalen-1-yl)-1-(pyridin-2-yl)but-1-en-3-yn-2-ol

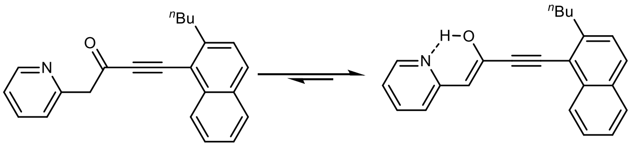

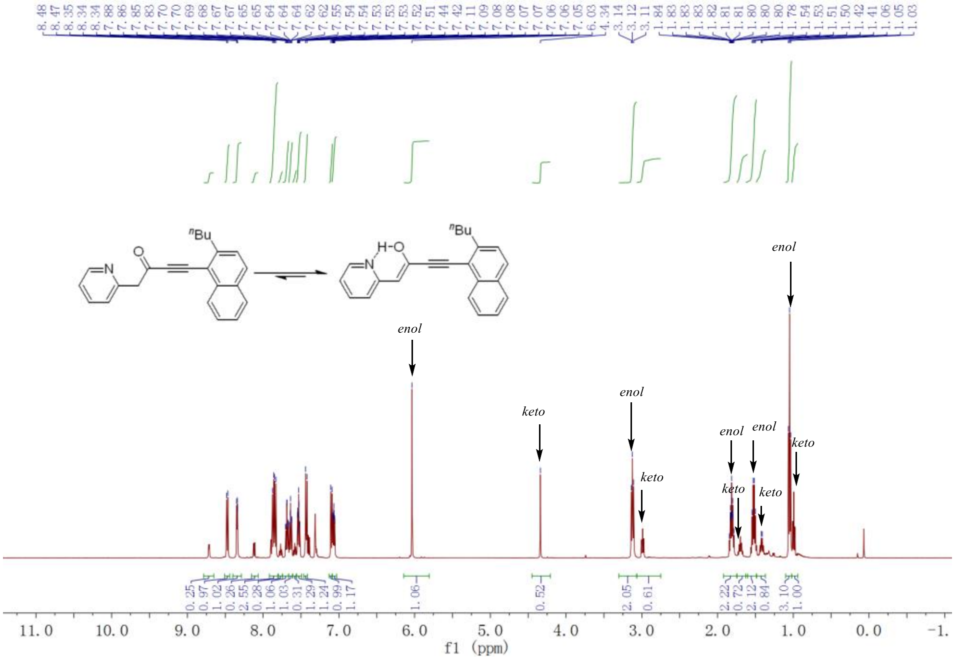

Supplementary Fig. 35. ^{1}H NMR spectrum of 1l.

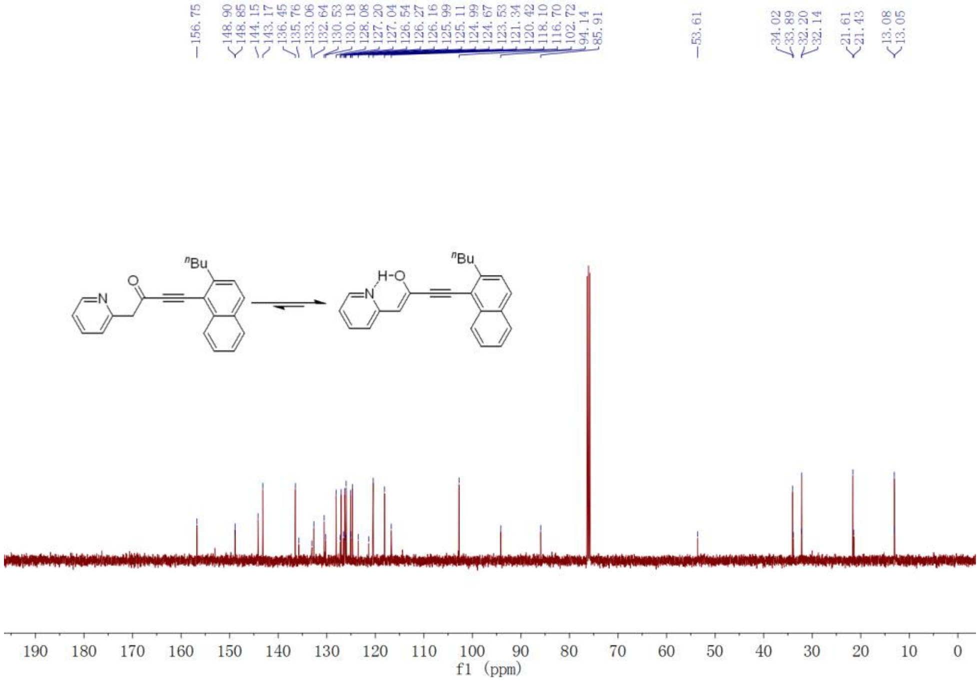

Supplementary Fig. 36. ^{13}C NMR spectrum of 1l.

1m, 4-(2-isopropylnaphthalen-1-yl)-1-(pyridin-2-yl)but-3-yn-2-one / (*Z*)-4-(2-isopropylnaphthalen-1-yl)-1-(pyridin-2-yl)but-1-en-3-yn-2-ol

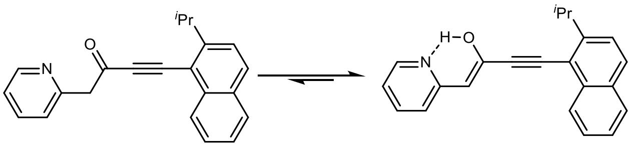

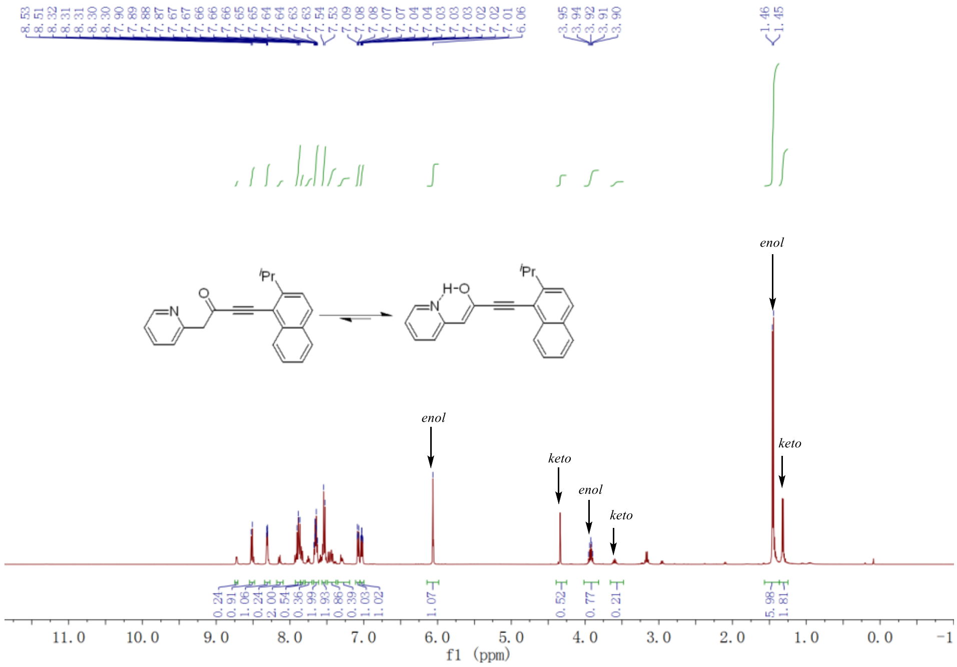

Supplementary Fig. 37. ^{1}H NMR spectrum of 1m.

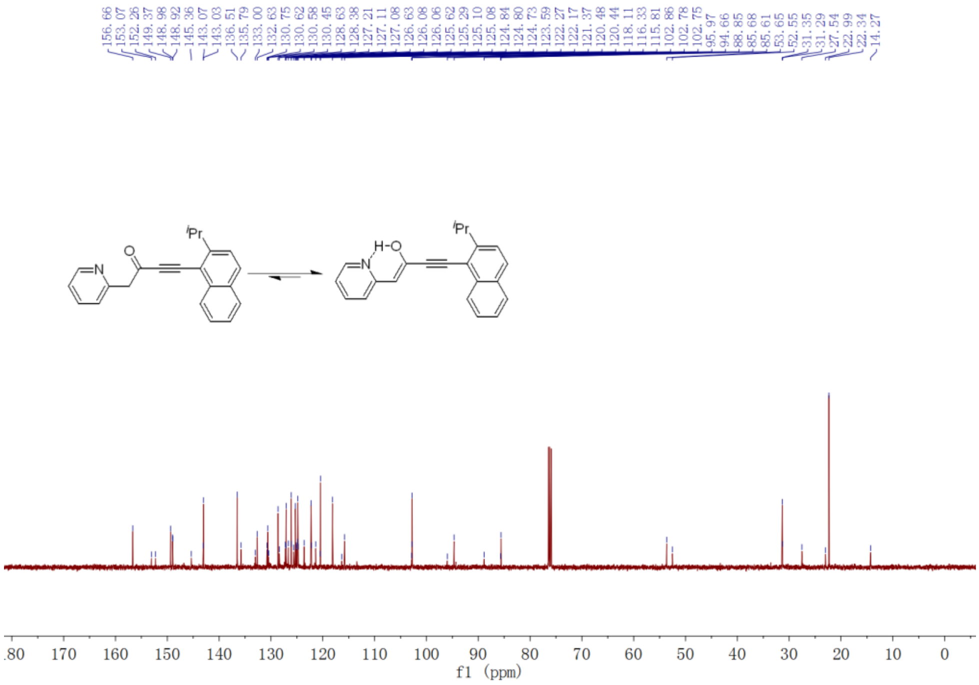

Supplementary Fig. 38. ^{13}C NMR spectrum of 1m.

1n, 4-(2-phenylnaphthalen-1-yl)-1-(pyridin-2-yl)but-3-yn-2-one / (*Z*)-4-(2-phenylnaphthalen-1-yl)-1-(pyridin-2-yl)but-1-en-3-yn-2-ol

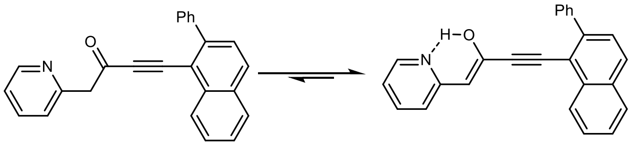

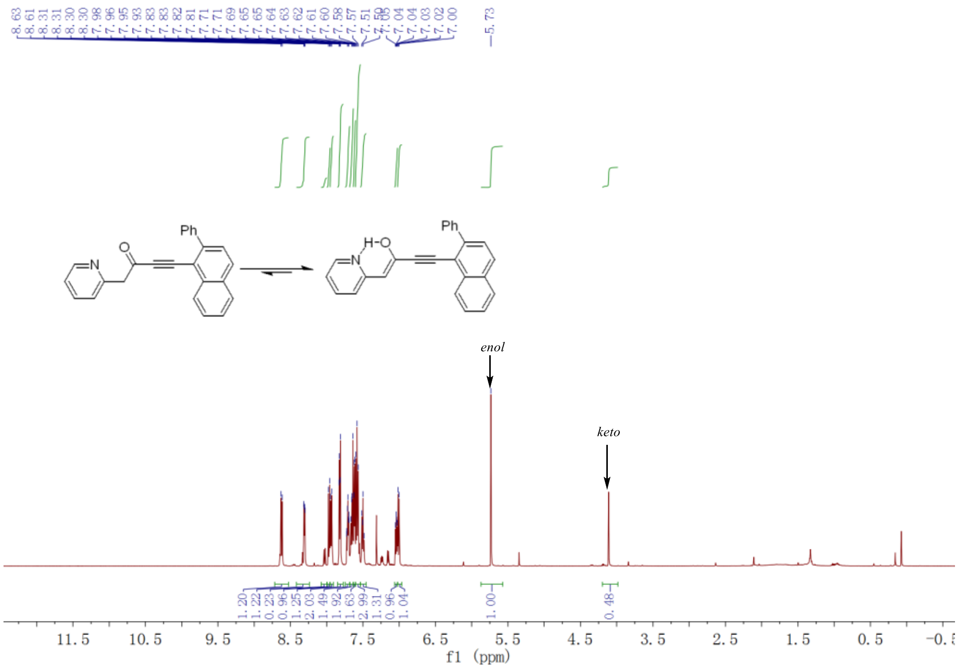

Supplementary Fig. 39. ^{1}H NMR spectrum of 1n.

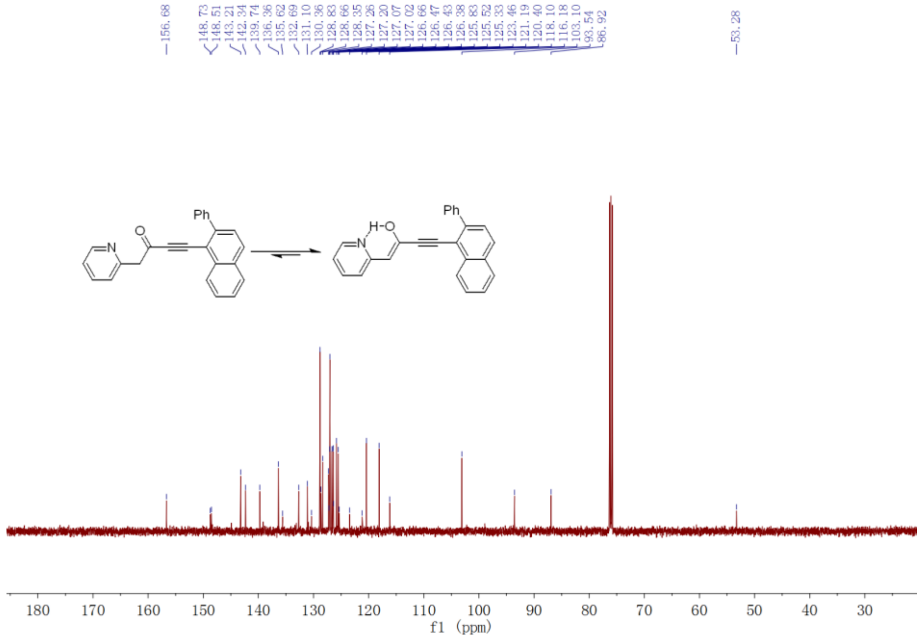

Supplementary Fig. 40. ^{13}C NMR spectrum of 1n.

1o, 4-([1,2'-binaphthalen]-1'-yl)-1-(pyridin-2-yl)but-3-yn-2-one / (Z)-4-([1,2'-binaphthalen]-1'-yl)-1-(pyridin-2-yl)but-1-en-3-yn-2-ol

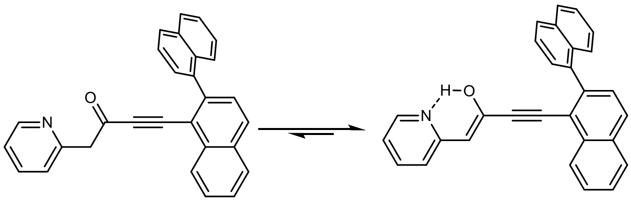

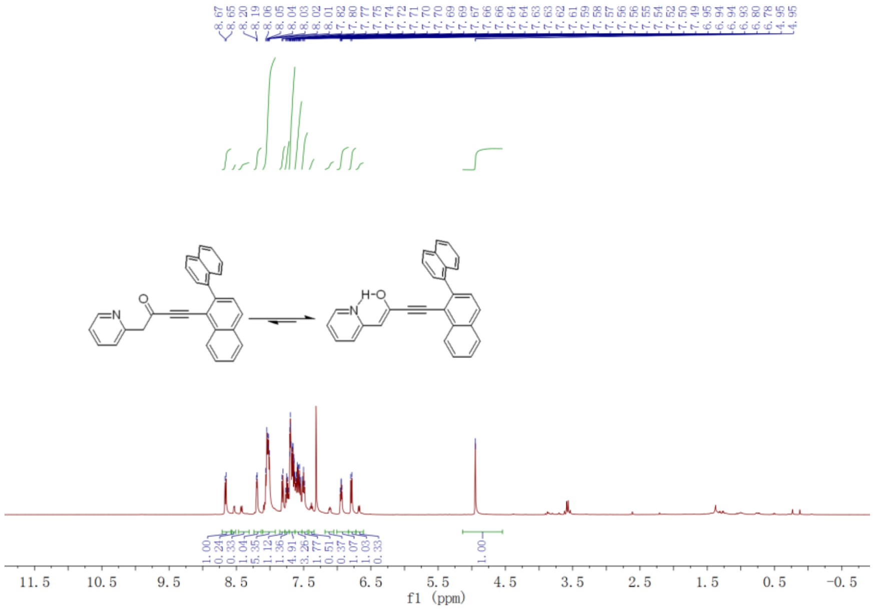

Supplementary Fig. 41. ^{1}H NMR spectrum of 1o.

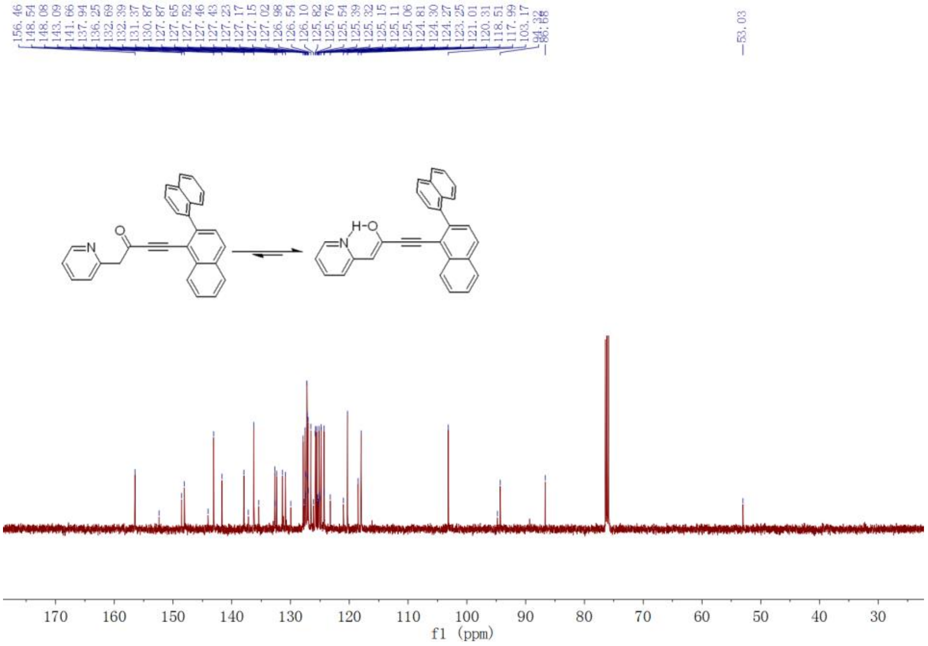

Supplementary Fig. 42. ^{13}C NMR spectrum of 1o.

1p, 4-(2-bromonaphthalen-1-yl)-1-(pyridin-2-yl)but-3-yn-2-one / (Z)-4-(2-bromonaphthalen-1-yl)-1-(pyridin-2-yl)but-1-en-3-yn-2-ol

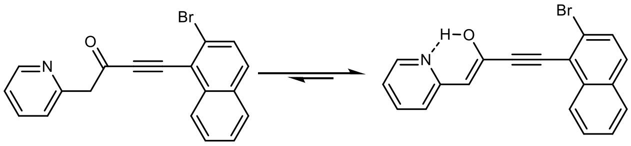

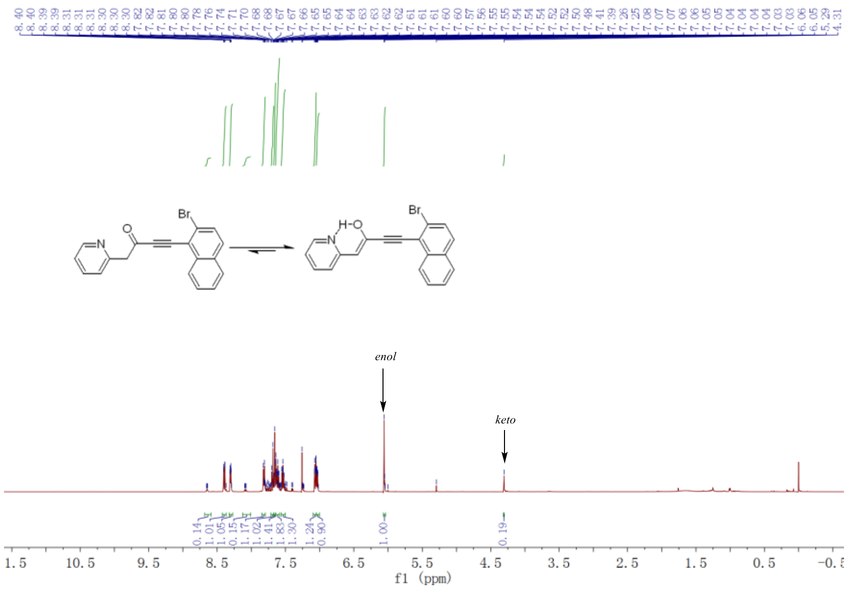

Supplementary Fig. 43. ^{1}H NMR spectrum of 1p.

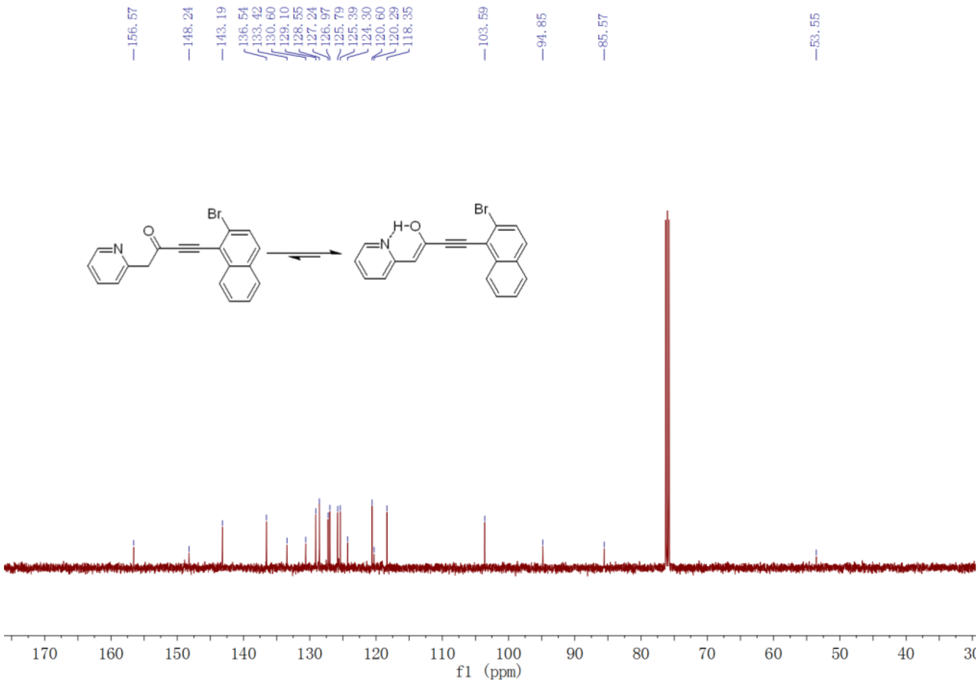

Supplementary Fig. 44. ^{13}C NMR spectrum of 1p.

1q, 4-(2-chloronaphthalen-1-yl)-1-(pyridin-2-yl)but-3-yn-2-one / (*Z*)-4-(2-chloronaphthalen-1-yl)-1-(pyridin-2-yl)but-1-en-3-yn-2-ol

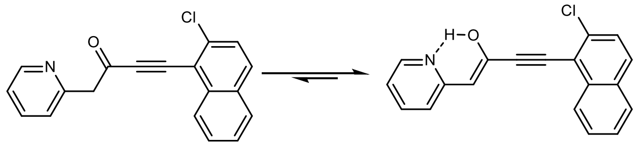

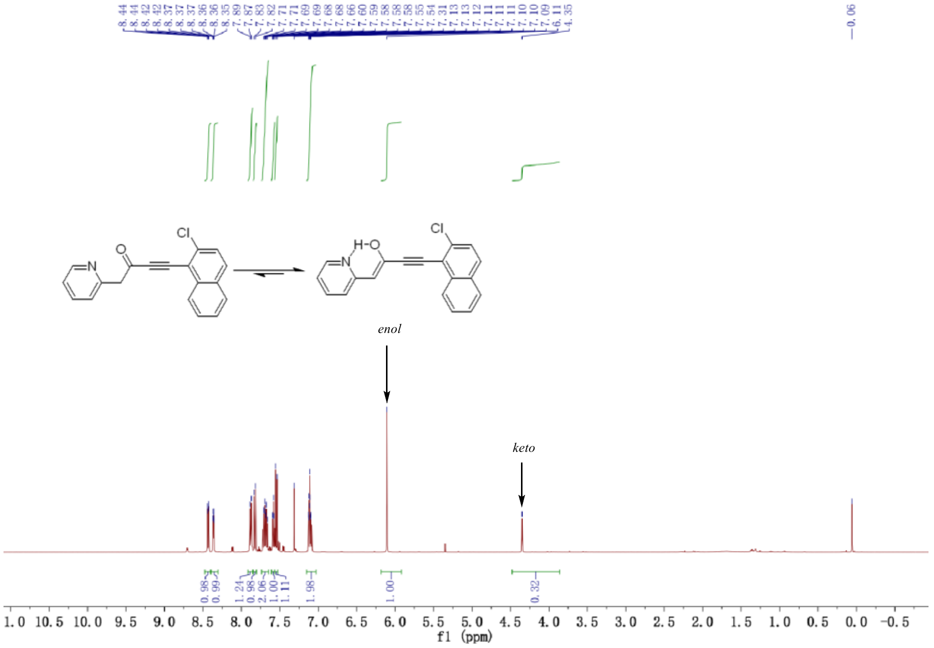

Supplementary Fig. 45. ^{1}H NMR spectrum of 1q.

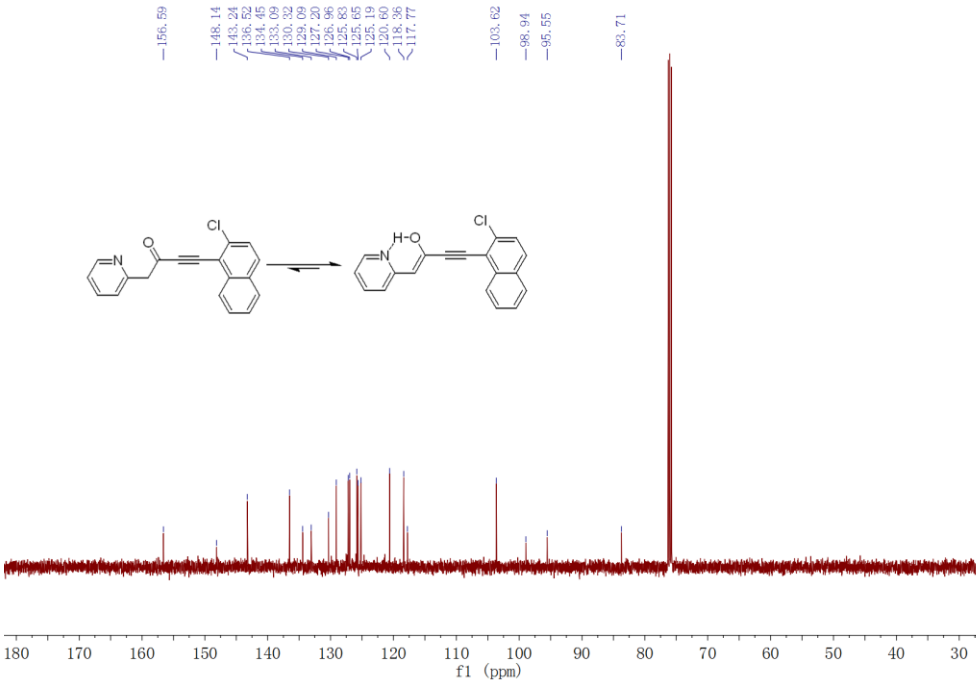

Supplementary Fig. 46. ^{13}C NMR spectrum of 1q.

1r, 1-(3-bromopyridin-2-yl)-4-(2-ethoxynaphthalen-1-yl)but-3-yn-2-one / (*Z*)-1-(3-bromopyridin-2-yl)-4-(2-ethoxynaphthalen-1-yl)but-1-en-3-yn-2-ol

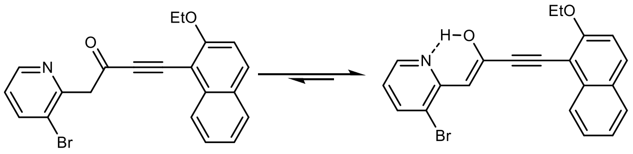

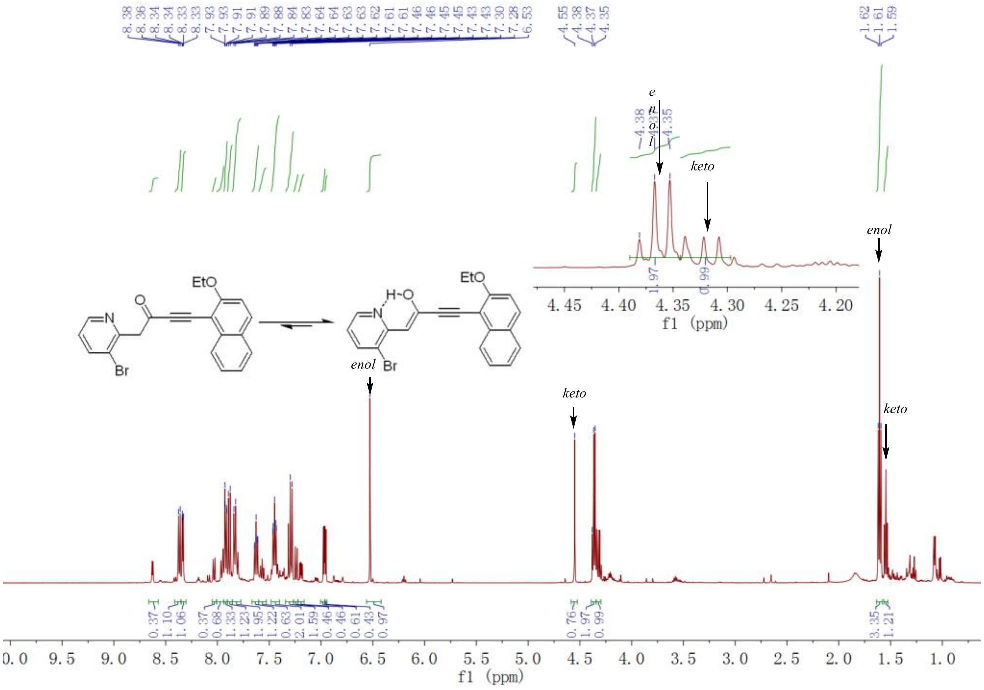

Supplementary Fig. 47. ^{1}H NMR spectrum of 1r.

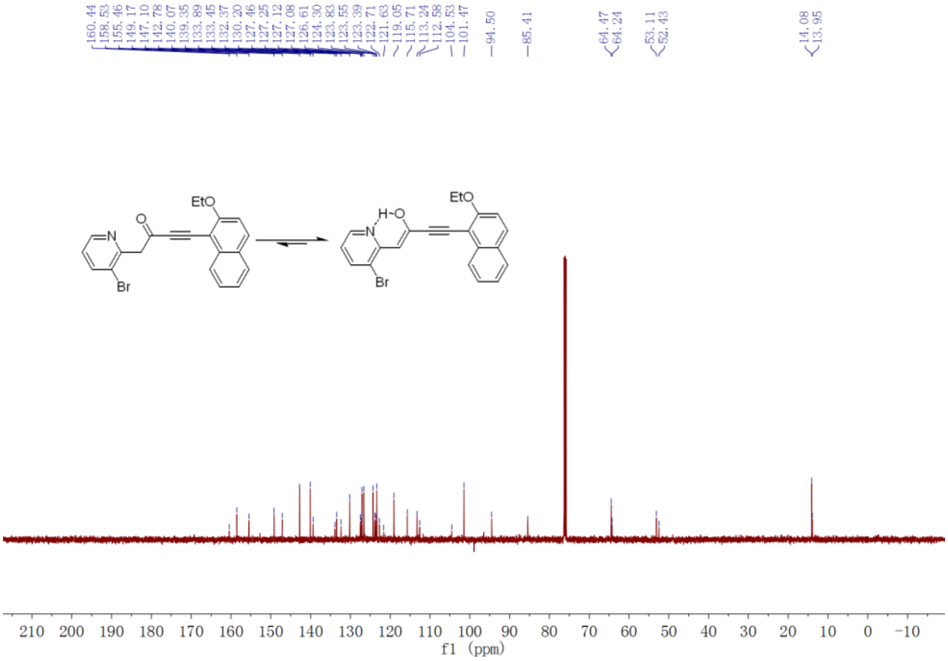

Supplementary Fig. 48. ^{13}C NMR spectrum of 1r.

1s, 1-(4-bromopyridin-2-yl)-4-(2-ethoxynaphthalen-1-yl)but-3-yn-2-one / (*Z*)-1-(4-bromopyridin -2-yl)-4-(2-ethoxynaphthalen-1-yl)but-1-en-3- yn-2-ol

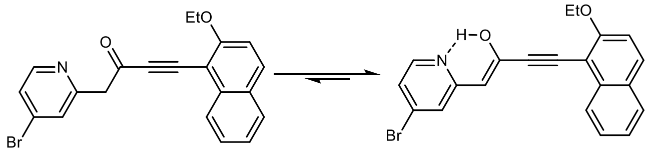

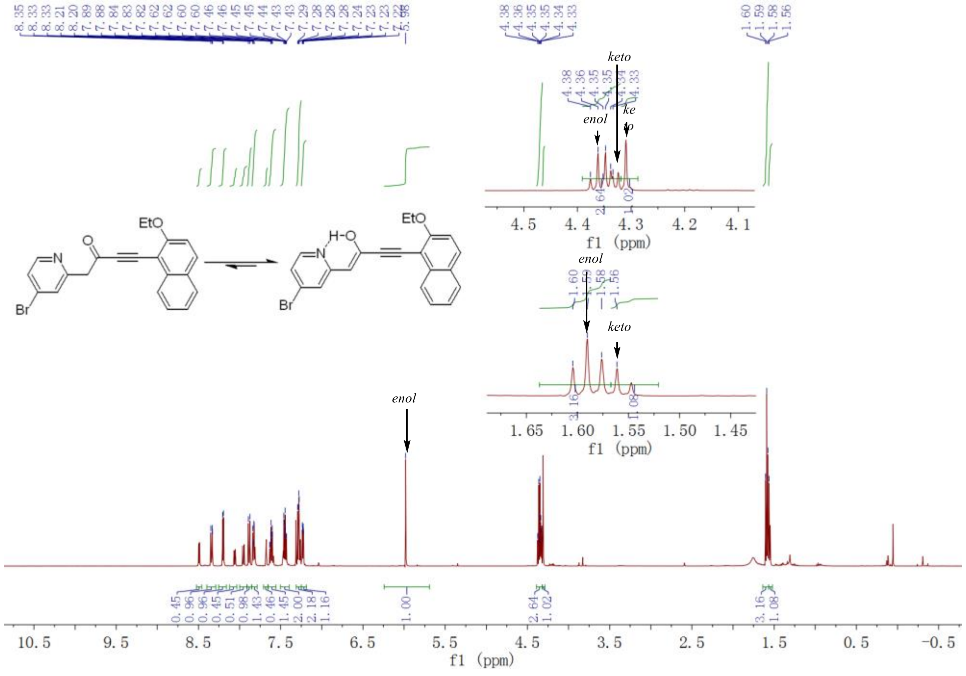

Supplementary Fig. 49. ^{1}H NMR spectrum of 1s.

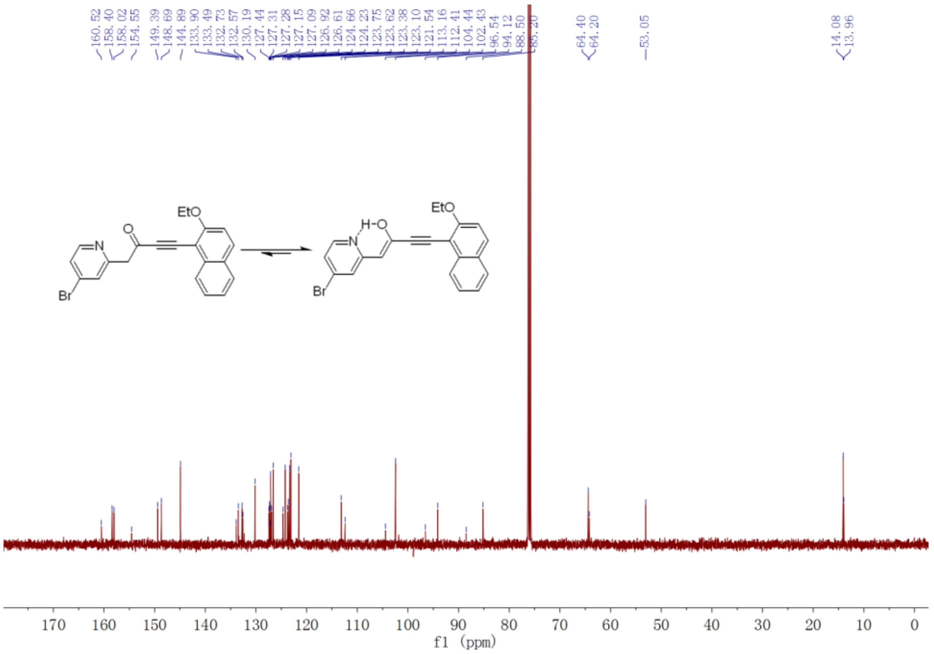

Supplementary Fig. 50. ^{13}C NMR spectrum of 1s.

1t, 1-(4-chloropyridin-2-yl)-4-(2-ethoxynaphthalen-1-yl)but-3-yn-2-one / (*Z*)-1-(4-chloropyridin-2-yl)-4-(2-ethoxynaphthalen-1-yl)but-1-en-3-yn-2-ol

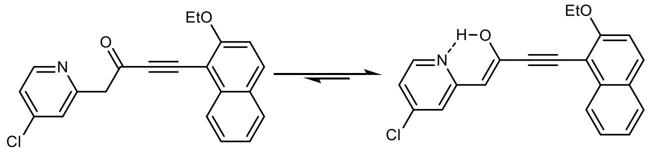

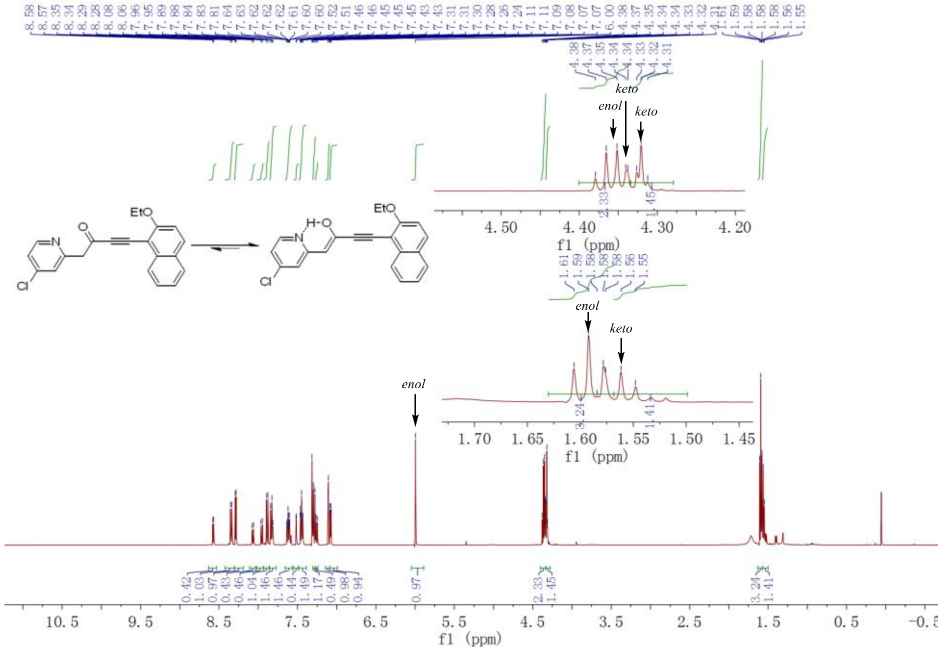

Supplementary Fig. 51. ^{1}H NMR spectrum of 1t.

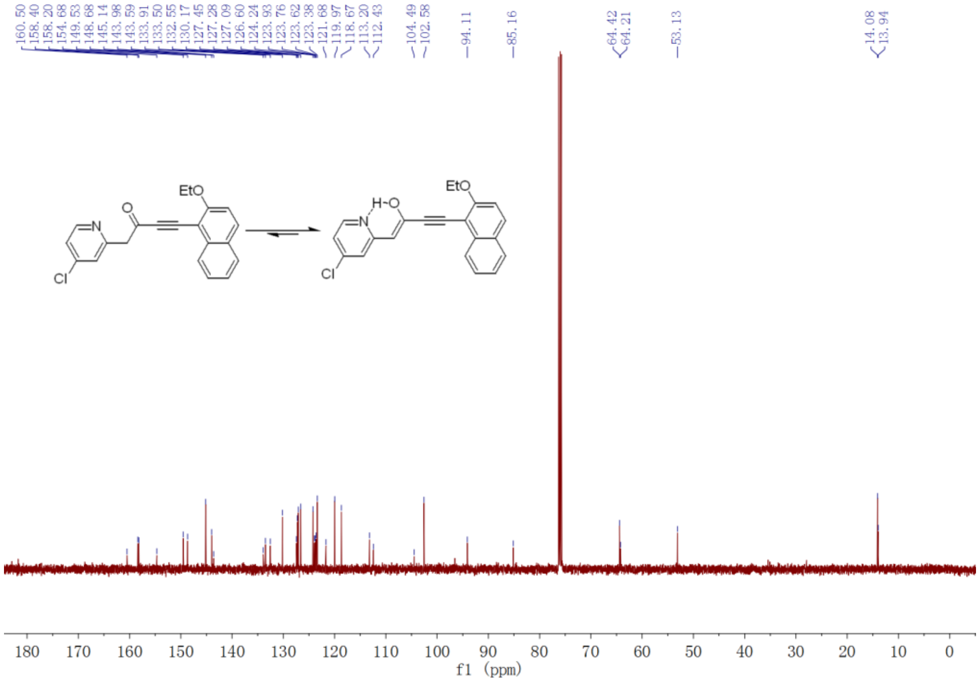

Supplementary Fig. 52. ^{13}C NMR spectrum of 1t.

1u, 1-(5-bromopyridin-2-yl)-4-(2-ethoxynaphthalen-1-yl)but-3-yn-2-one / (*Z*)-1-(5-bromopyridin-2-yl)-4-(2-ethoxynaphthalen-1-yl)but-1-en-3- yn-2-ol

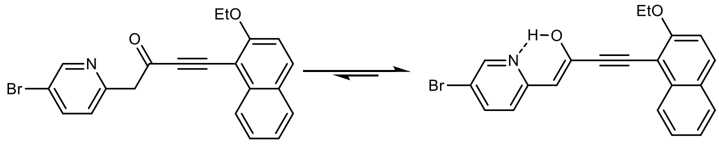

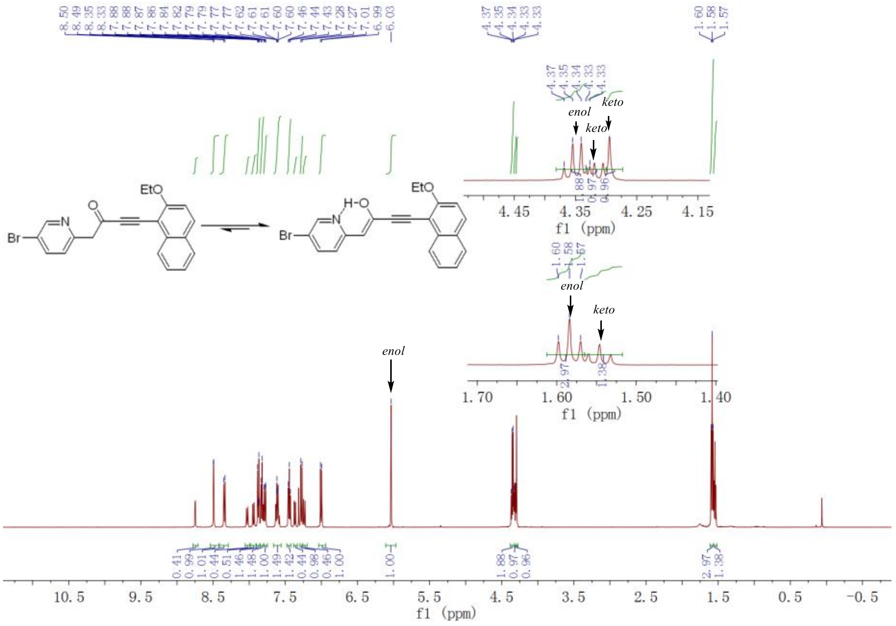

Supplementary Fig. 53. ^{1}H NMR spectrum of 1u.

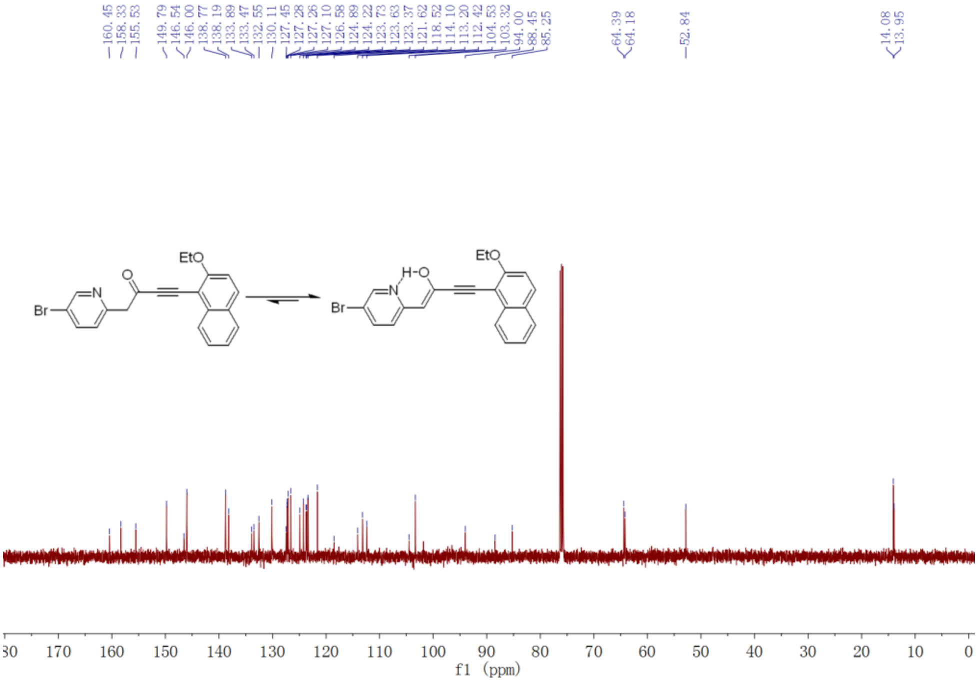

Supplementary Fig. 54. ^{13}C NMR spectrum of 1u.

1v, 4-(2-ethoxynaphthalen-1-yl)-1-(5-methylpyridin-2-yl)but-3-yn-2-one / (*Z*)-4-(2-ethoxynaphthalen-1-yl)-1-(5-methylpyridin-2-yl)but-1-en-3-yn-2-ol

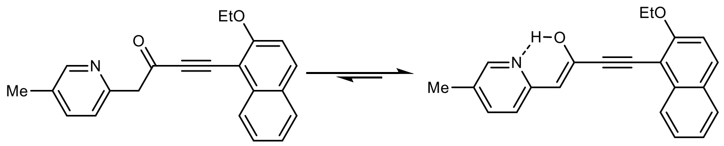

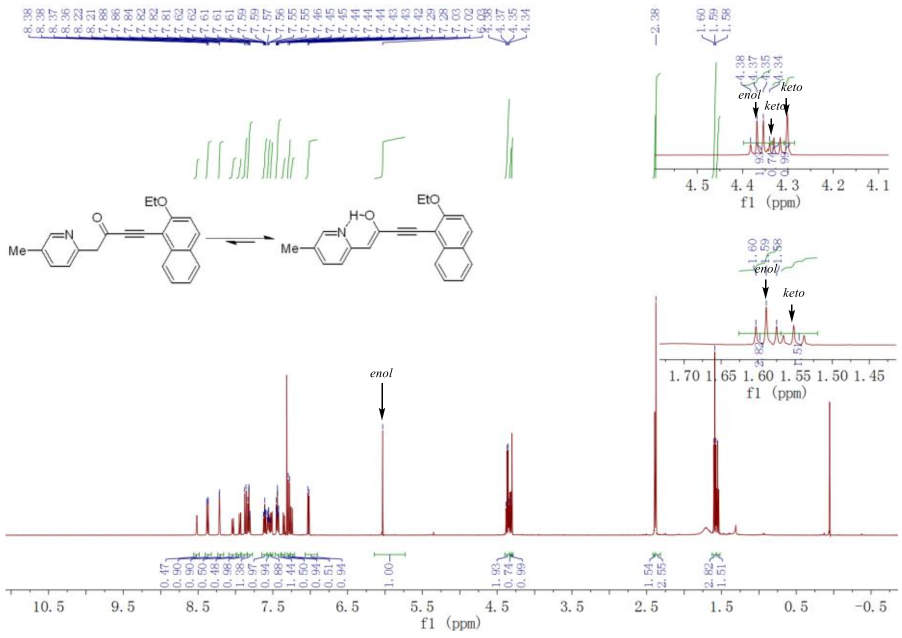

Supplementary Fig. 55. ^{1}H NMR spectrum of 1v.

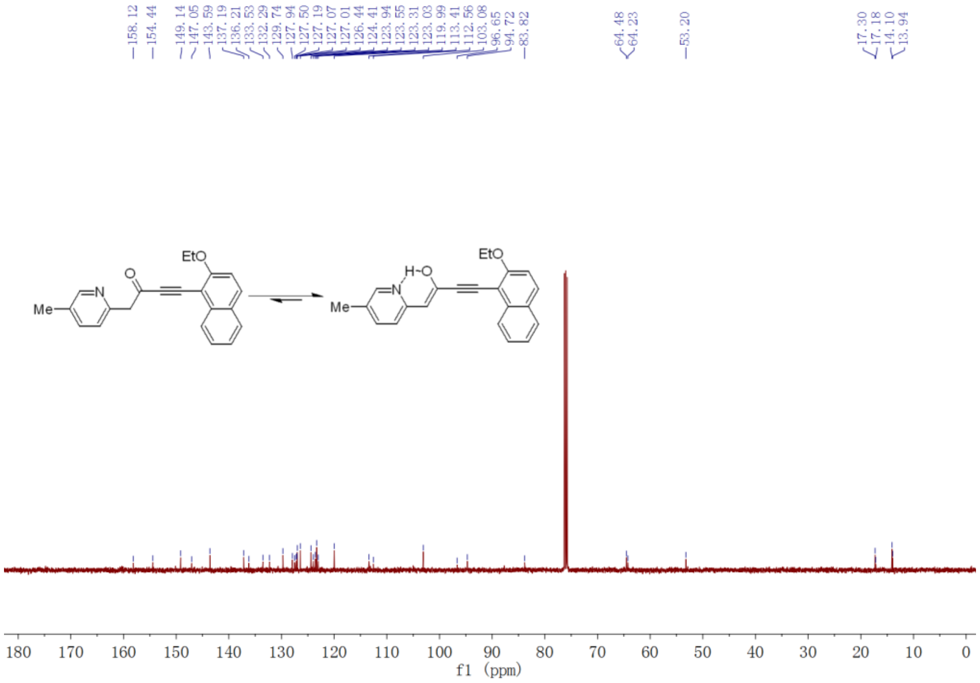

Supplementary Fig. 56. ^{13}C NMR spectrum of 1v.

1w, 4-(2-ethoxynaphthalen-1-yl)-1-(pyrazin-2-yl)but-3-yn-2-one / (*Z*)-4-(2-ethoxynaphthalen-1-yl)-1-(pyrazin-2-yl)but-1-en-3-yn-2-ol

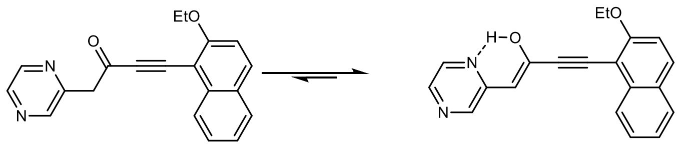

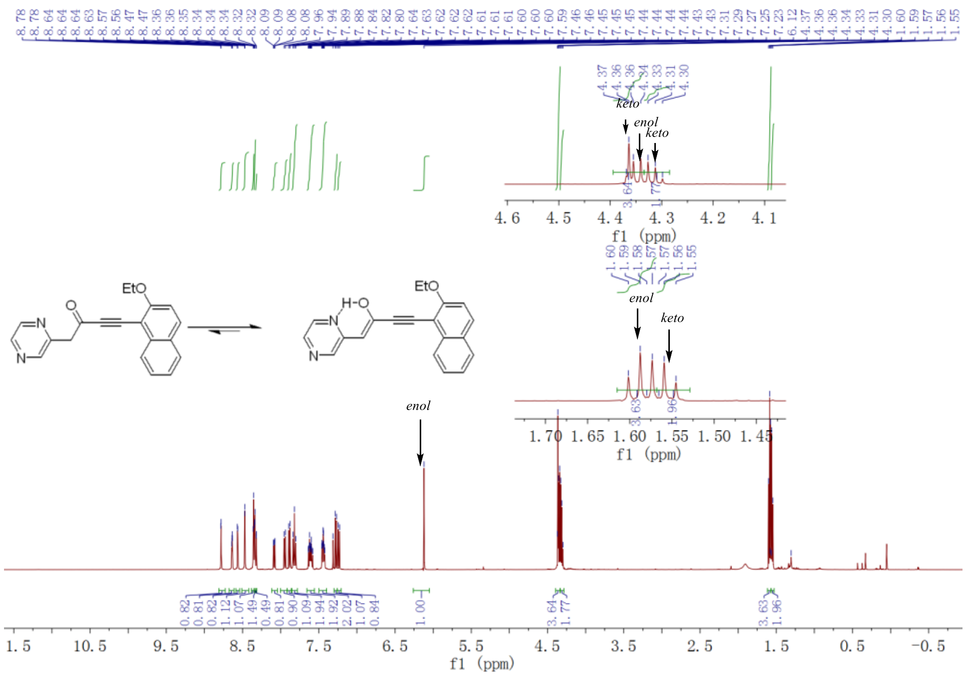

Supplementary Fig. 57. ^{1}H NMR spectrum of 1w.

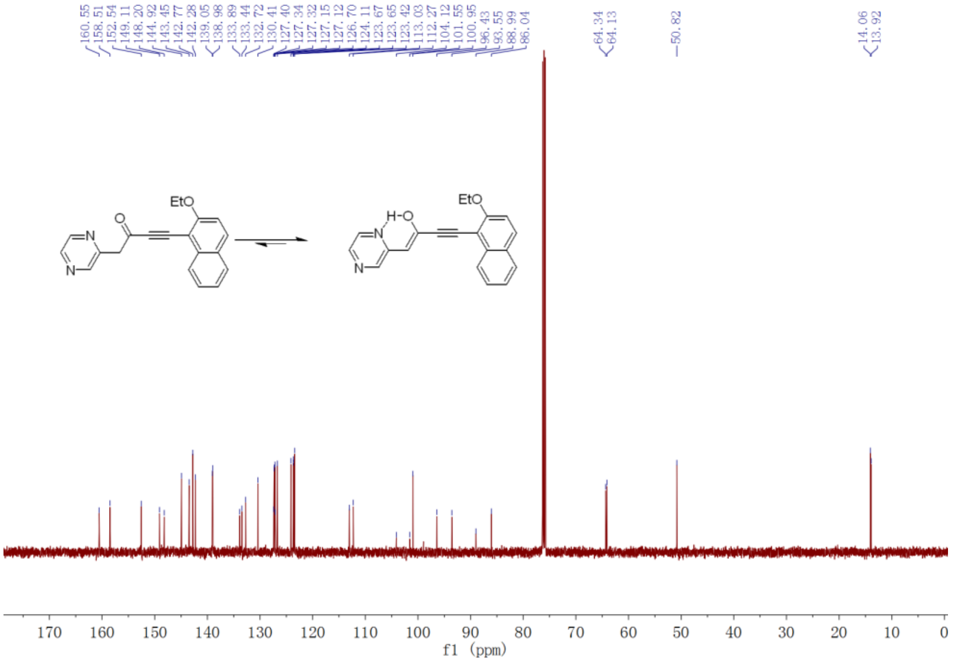

Supplementary Fig. 58. ^{13}C NMR spectrum of 1w.

1x, 4-(2-ethoxynaphthalen-1-yl)-1-(5-(4-((((3a*S*,5a*R*,8a*R*,8b*S*)-2,2,7,7-tetramethyltetrahydro-3a*H*-bis([1,3]dioxolo)[4,5-b:4',5'-d]pyran-3a-yl)methoxy)methyl)phenyl)pyridin-2-yl)but-3-yn-2-one / (*Z*)-4-(2-ethoxynaphthalen-1-yl)-1-(5-(4-((((3a*S*,5a*R*,8a*R*,8b*S*)-2,2,7,7-tetramethyltetrahydro-3a*H*-bis([1,3]dioxolo)[4,5-b:4',5'-d]pyran-3a-yl)methoxy)methyl)phenyl)pyridin-2-yl)but-1-en-3-yn-2-ol

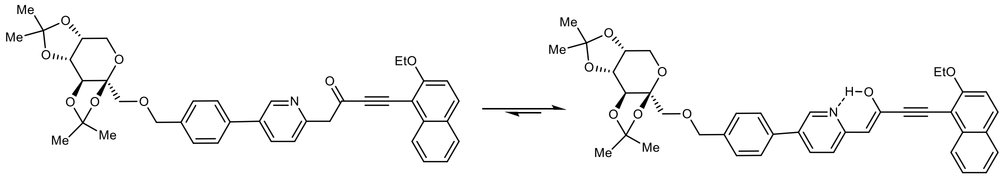

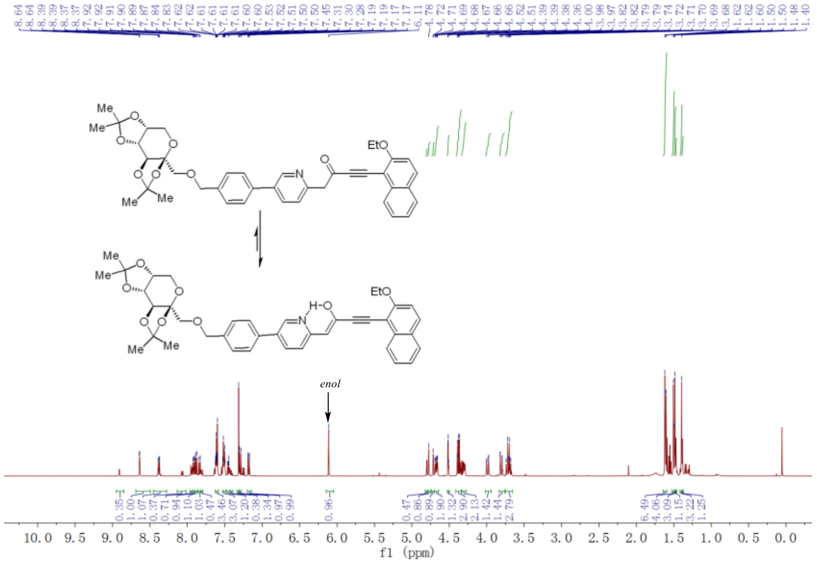

Supplementary Fig. 59. ^{1}H NMR spectrum of 1x.

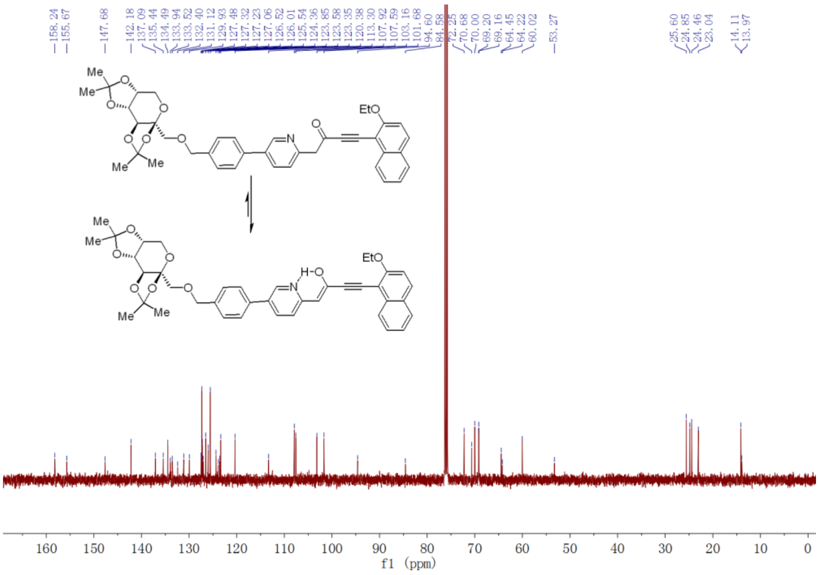

Supplementary Fig. 60. ^{13}C NMR spectrum of 1x.

1y, 4-(2-ethoxynaphthalen-1-yl)-1-(5-(4-((((*R*)-2,5,7,8-tetramethyl-2-((4*S*,8*R*)-4,8,12-trimethyltridecyl)chroman-6-yl)oxy)methyl)phenyl)pyridin-2-yl)but-3-yn-2-one / (*Z*)-4-(2-ethoxynaphthalen-1-yl)-1-(5-(4-((((*R*)-2,5,7,8-tetramethyl-2-((4*S*,8*R*)-4,8,12-trimethyltridecyl)chroman-6-yl)oxy)methyl)phenyl)pyridin-2-yl)but-1-en-3-yn-2-ol

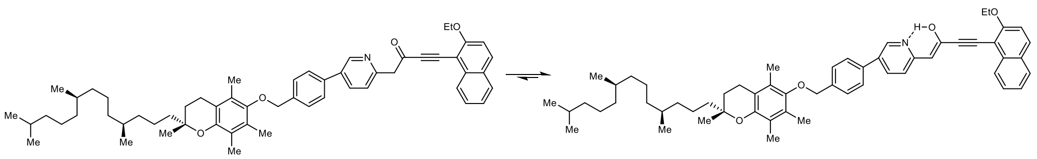

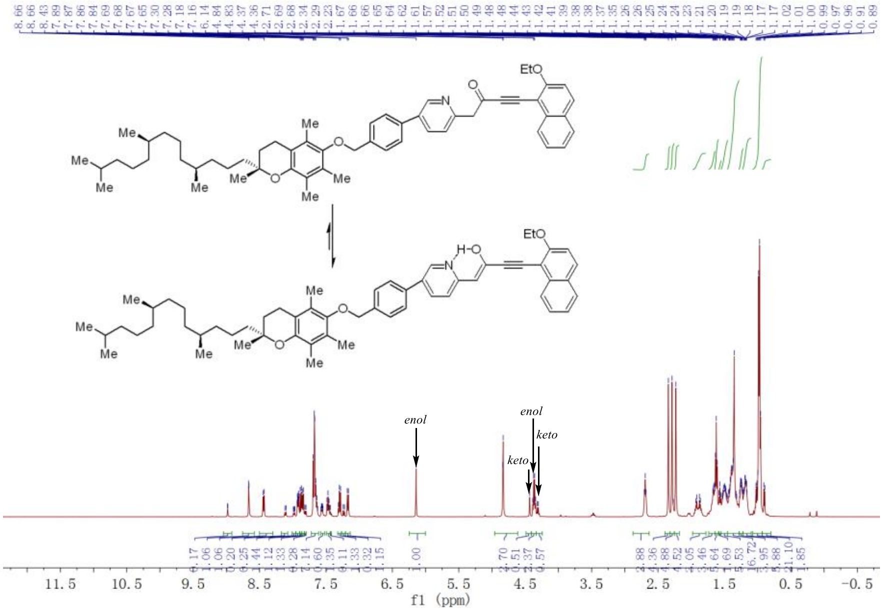

Supplementary Fig. 61. ^{1}H NMR spectrum of 1y.

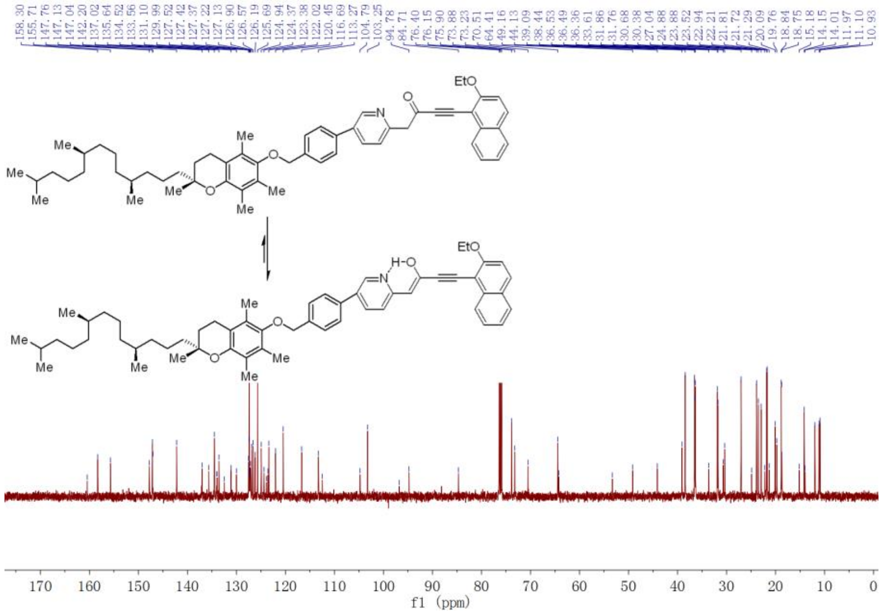

Supplementary Fig. 62. ^{13}C NMR spectrum of 1y.

1z, 4-(2-ethoxynaphthalen-1-yl)-1-(5-(4-((((1*R*,2*S*,5*R*)-2-isopropyl-5-methylcyclohexyl)oxy) methyl)phenyl)pyridin-2-yl)but-3-yn-2-one / (*Z*)-4-(2-ethoxynaphthalen-1-yl)-1-(5-(4-((((1*R*,2*S*, 5*R*)-2-isopropyl-5-methylcyclohexyl)oxy) -methyl)phenyl)pyridin-2-yl)but-1-en-3-yn-2-ol

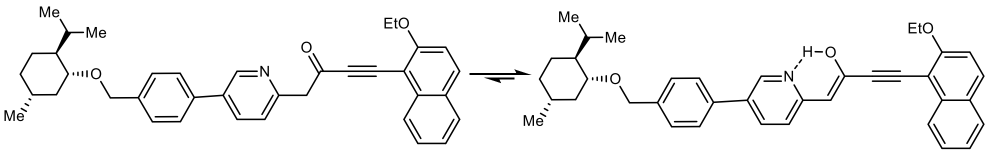

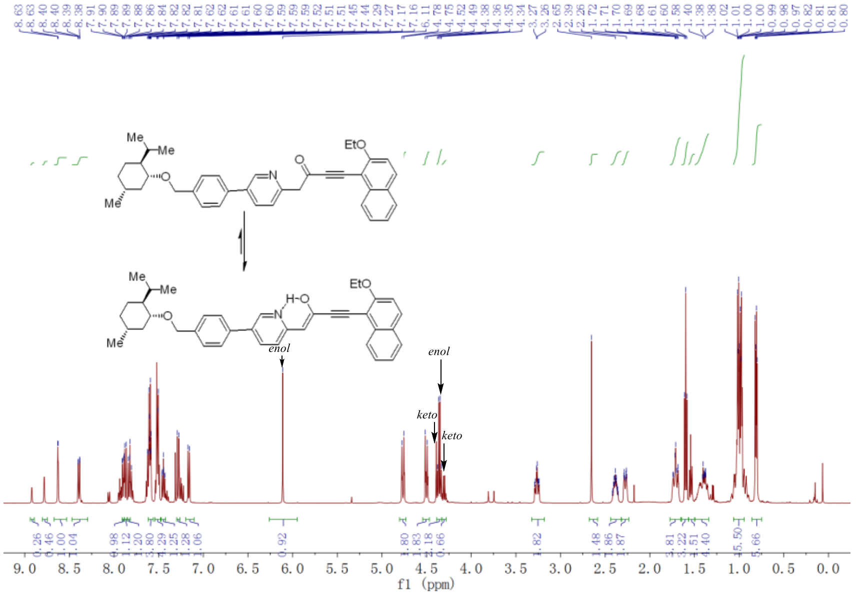

Supplementary Fig. 63. ^{1}H NMR spectrum of 1z.

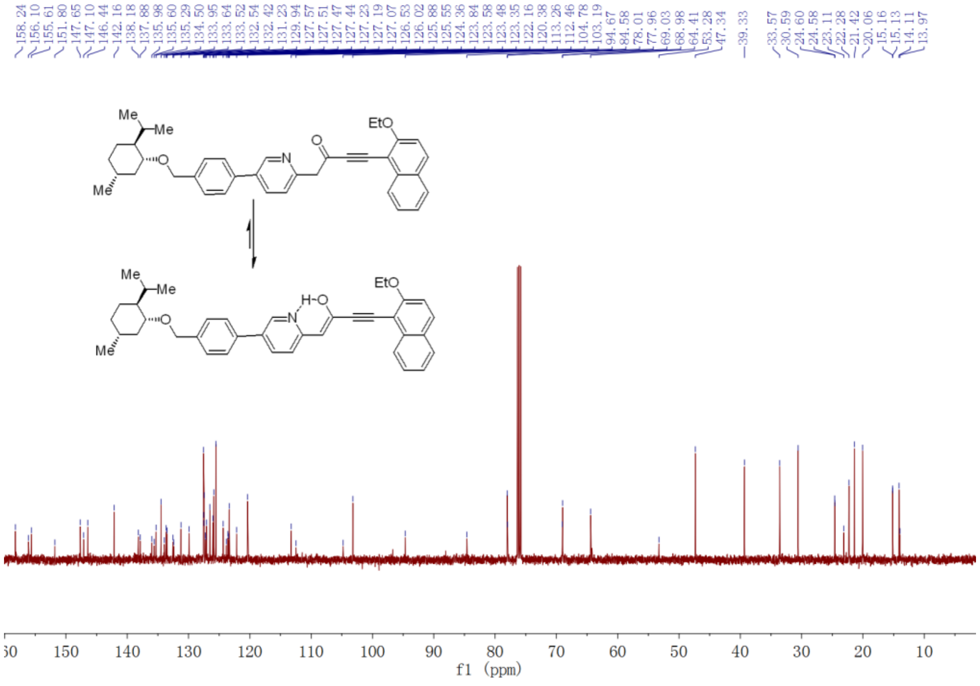

Supplementary Fig. 64. ^{13}C NMR spectrum of 1z.

2a, 4-(2-methoxynaphthalen-1-yl)-2*H*-quinolizin-2-one

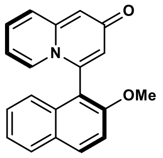

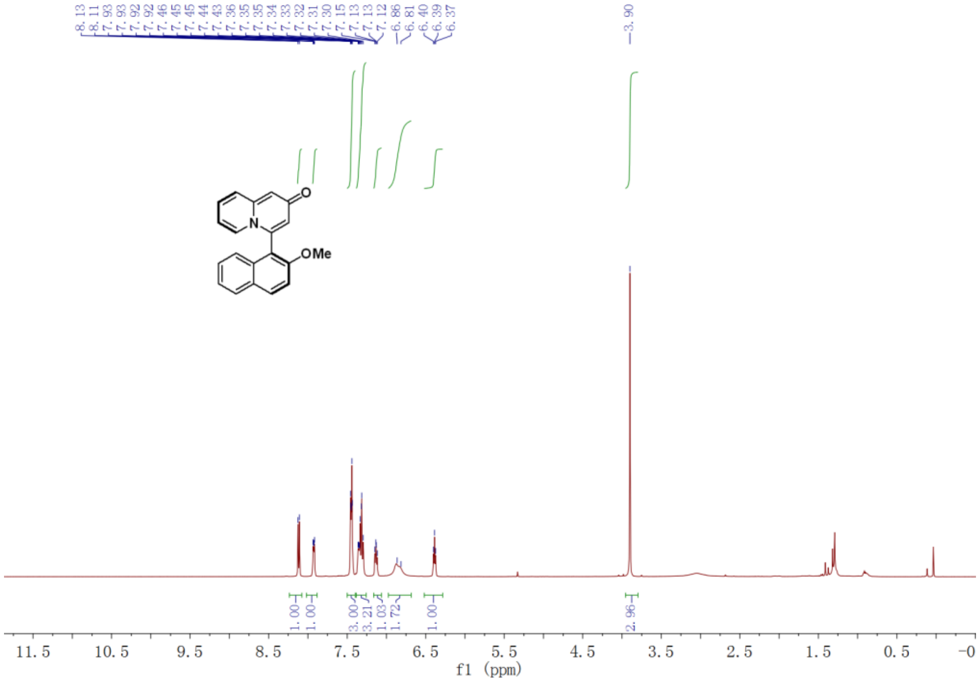

Supplementary Fig. 65. ^{1}H NMR spectrum of 2a.

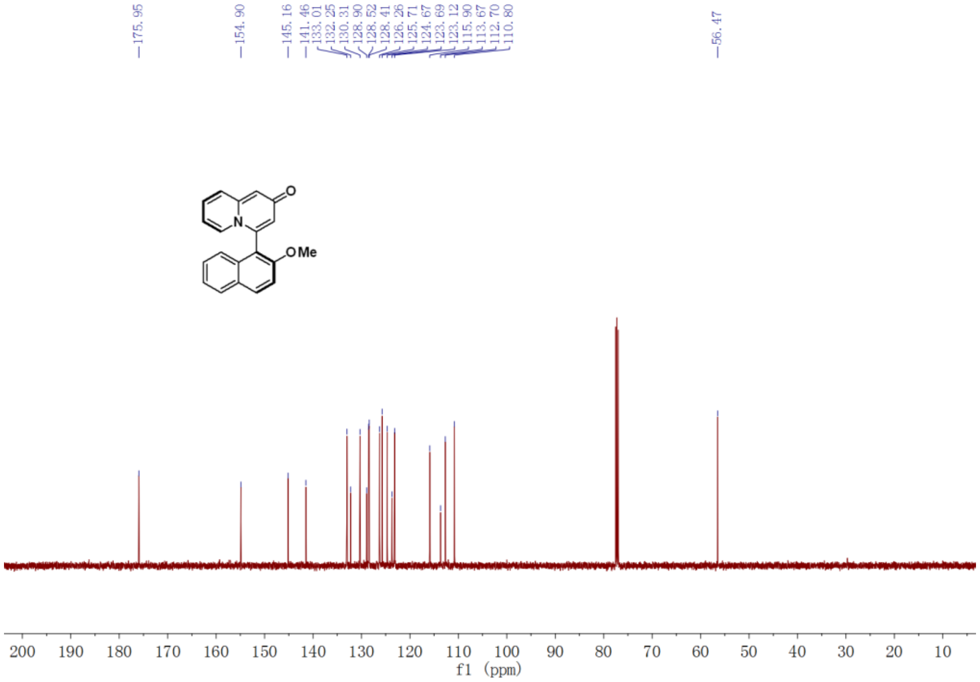

Supplementary Fig. 66. ^{13}C NMR spectrum of 2a.

2b, 4-(2-ethoxynaphthalen-1-yl)-2*H*-quinolizin-2-one

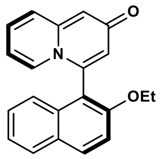

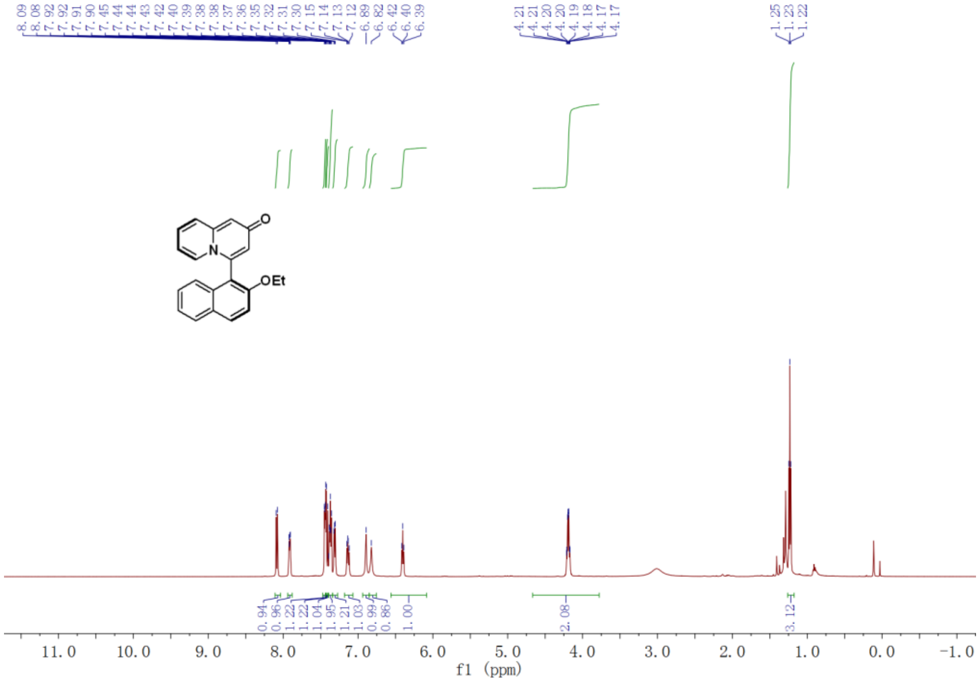

Supplementary Fig. 67. ^{1}H NMR spectrum of 2b.

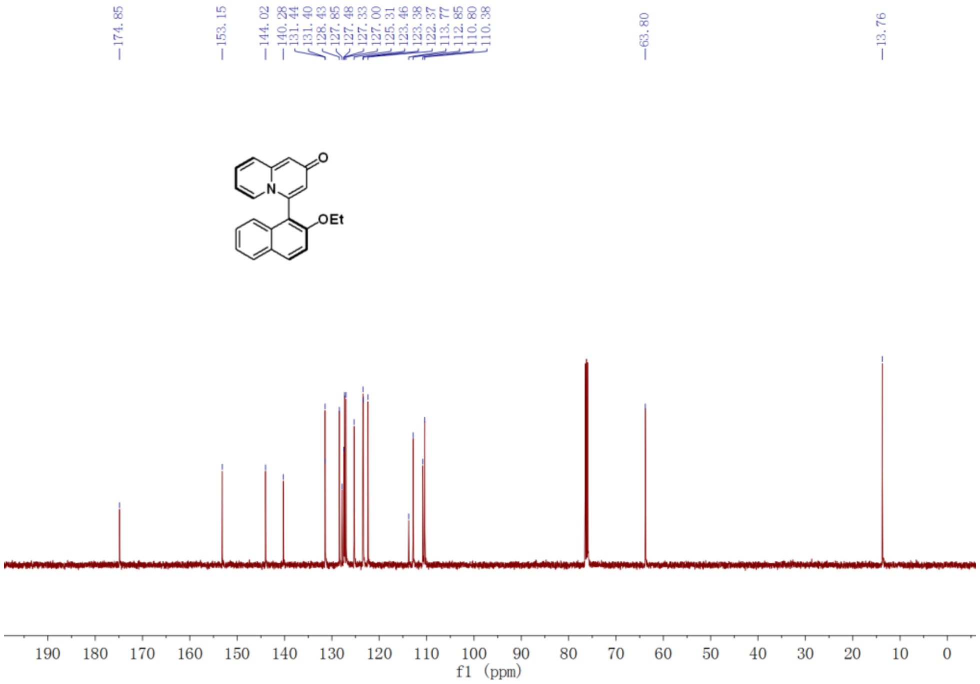

Supplementary Fig. 68. ^{13}C NMR spectrum of 2b.

2c, 4-(6-bromo-2-ethoxynaphthalen-1-yl)-2*H*-quinolizin-2-one

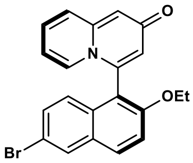

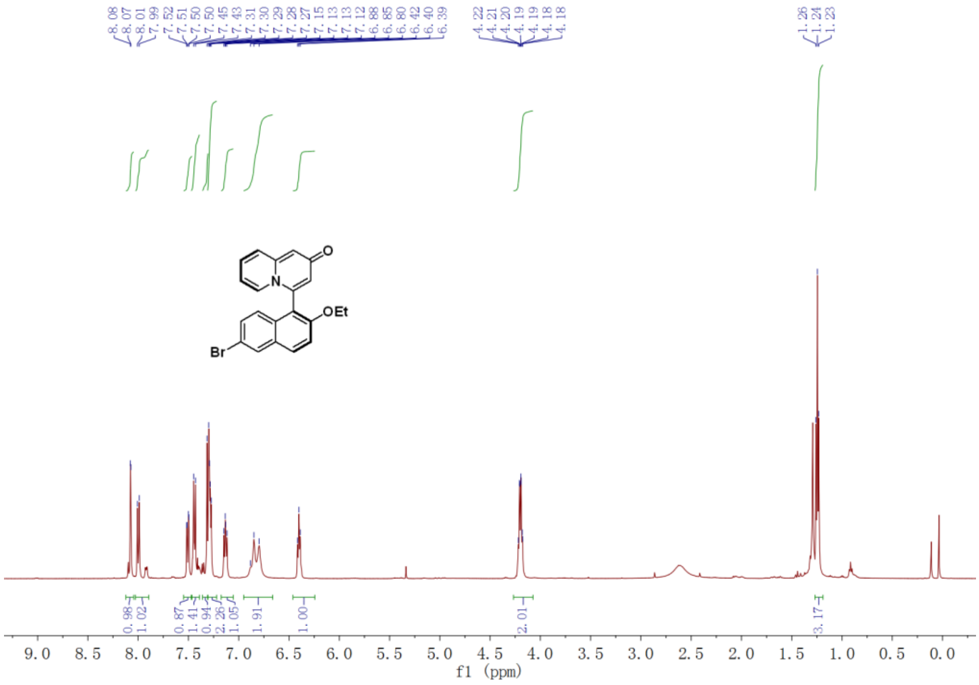

Supplementary Fig. 69. ^{1}H NMR spectrum of 2c.

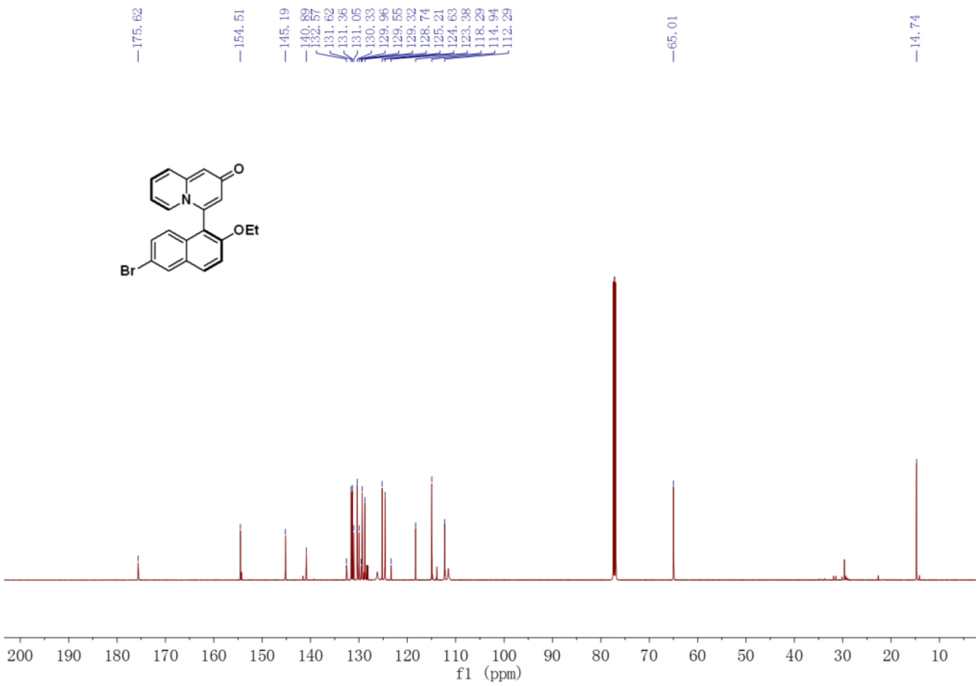

Supplementary Fig. 70. ^{13}C NMR spectrum of 2c.

2d, 4-(7-bromo-2-ethoxynaphthalen-1-yl)-2*H*-quinolizin-2-one

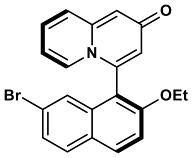

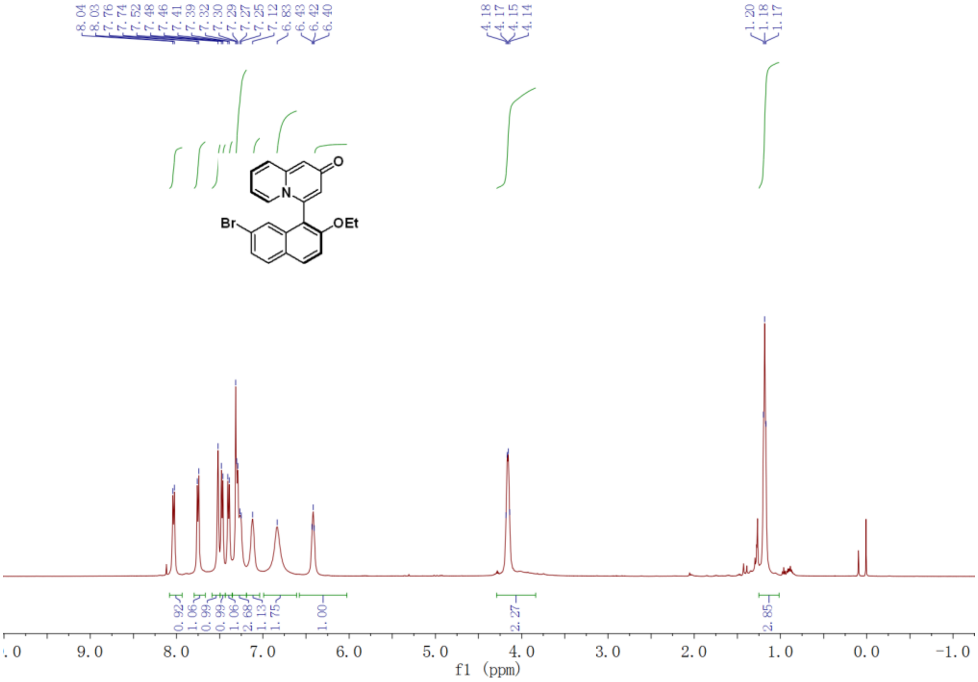

Supplementary Fig. 71. ^{1}H NMR spectrum of 2d.

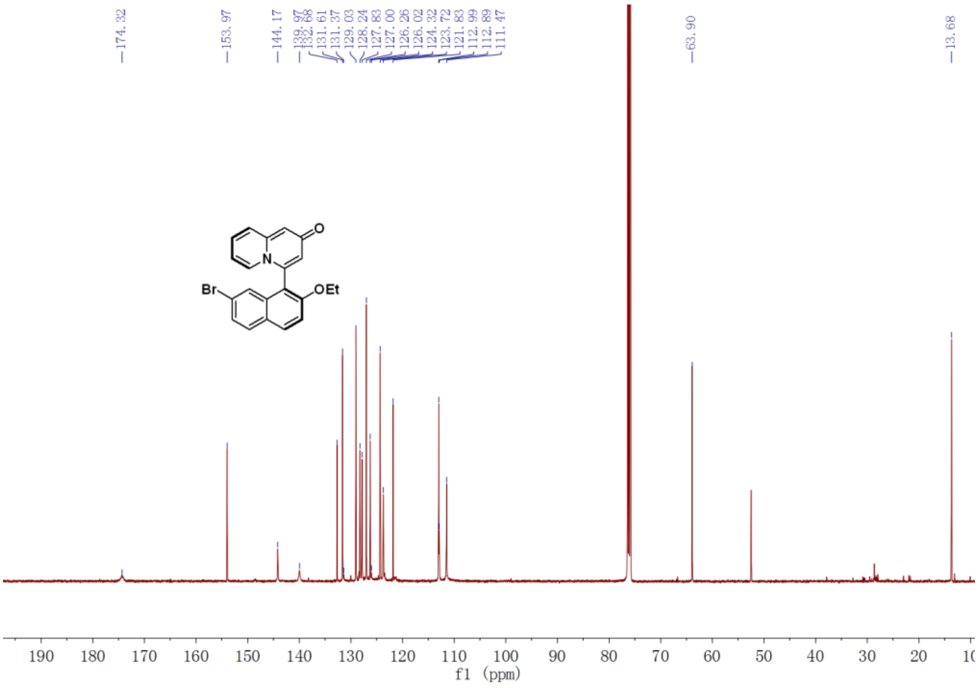

Supplementary Fig. 72. ^{13}C NMR spectrum of 2d.

2e, 4-(2-ethoxy-7-phenylnaphthalen-1-yl)-2*H*-quinolizin-2-one

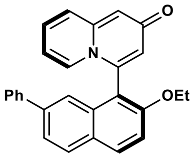

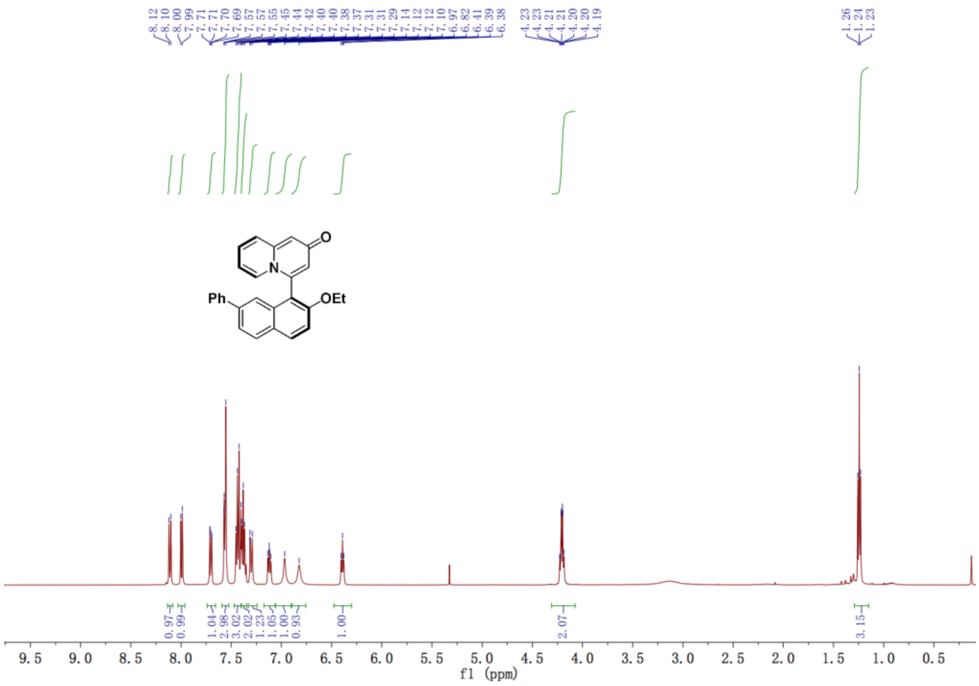

Supplementary Fig. 73. ¹H NMR spectrum of 2e.

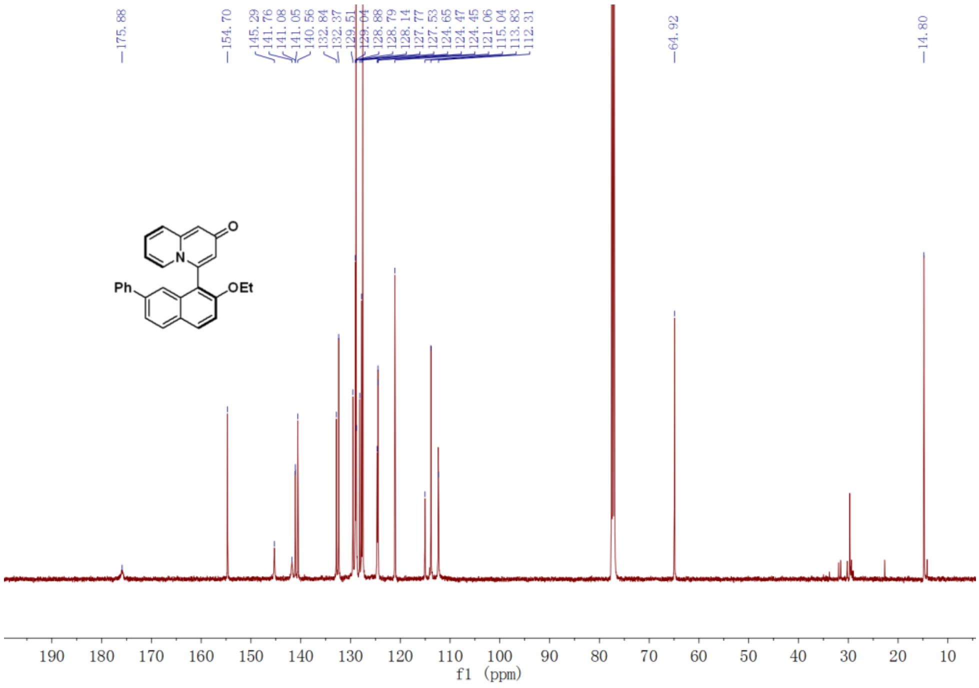

Supplementary Fig. 74. ^{13}C NMR spectrum of 2e.

2f, 4-(2-ethoxy-7-(phenylethynyl)naphthalen-1-yl)-2*H*-quinolizin-2-one

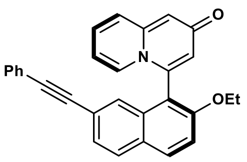

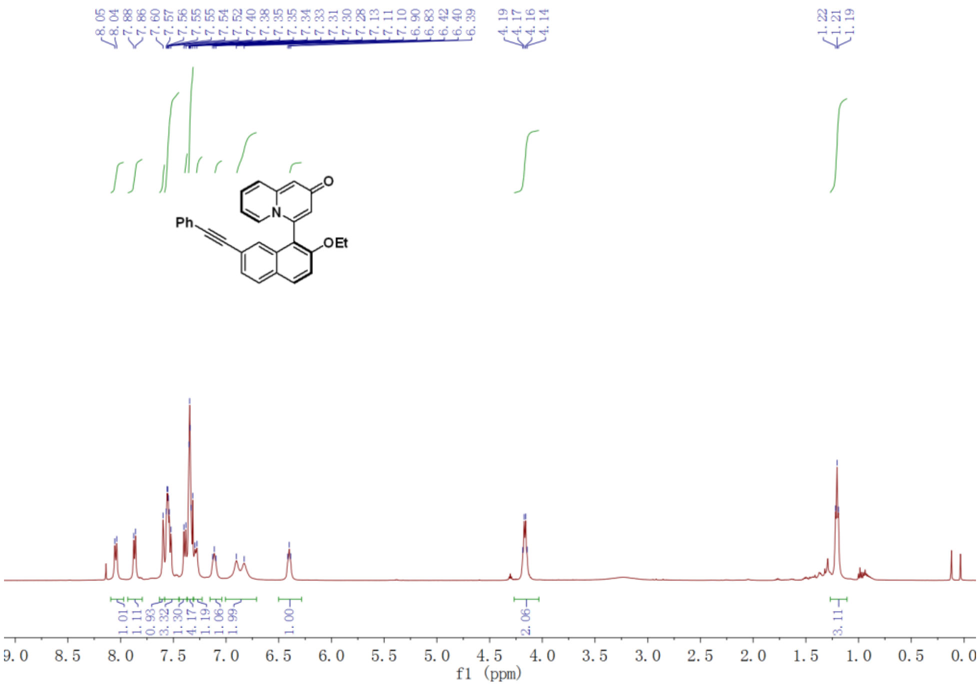

Supplementary Fig. 75. ^{1}H NMR spectrum of 2f.

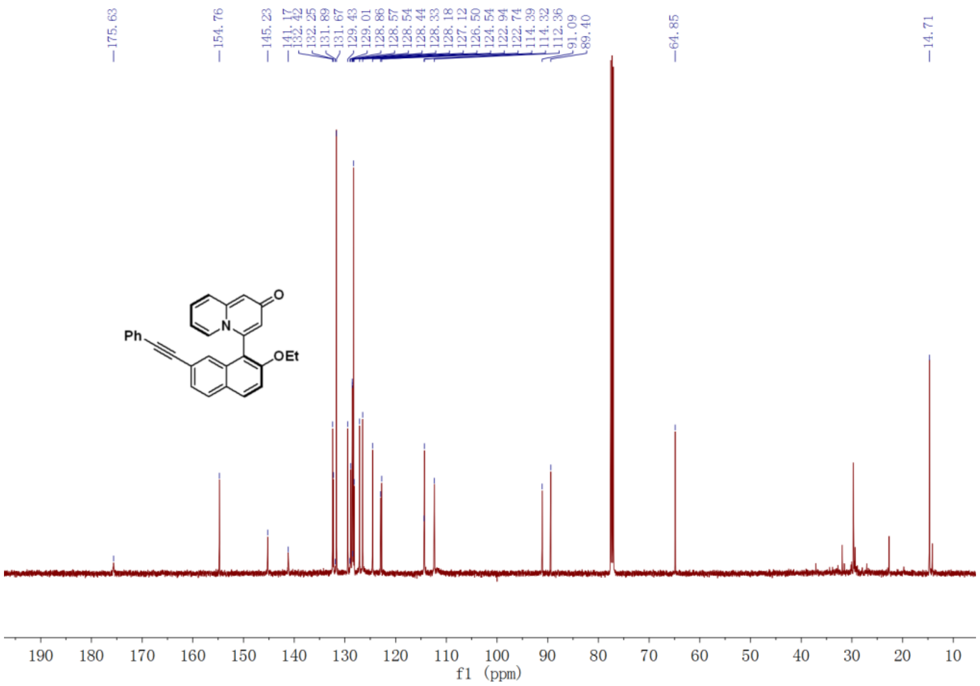

Supplementary Fig. 76. ^{13}C NMR spectrum of 2f.

2g, 4-(2-((4-methoxybenzyl)oxy)naphthalen-1-yl)-2*H*-quinolizin-2-one

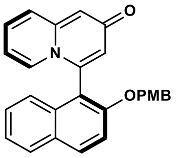

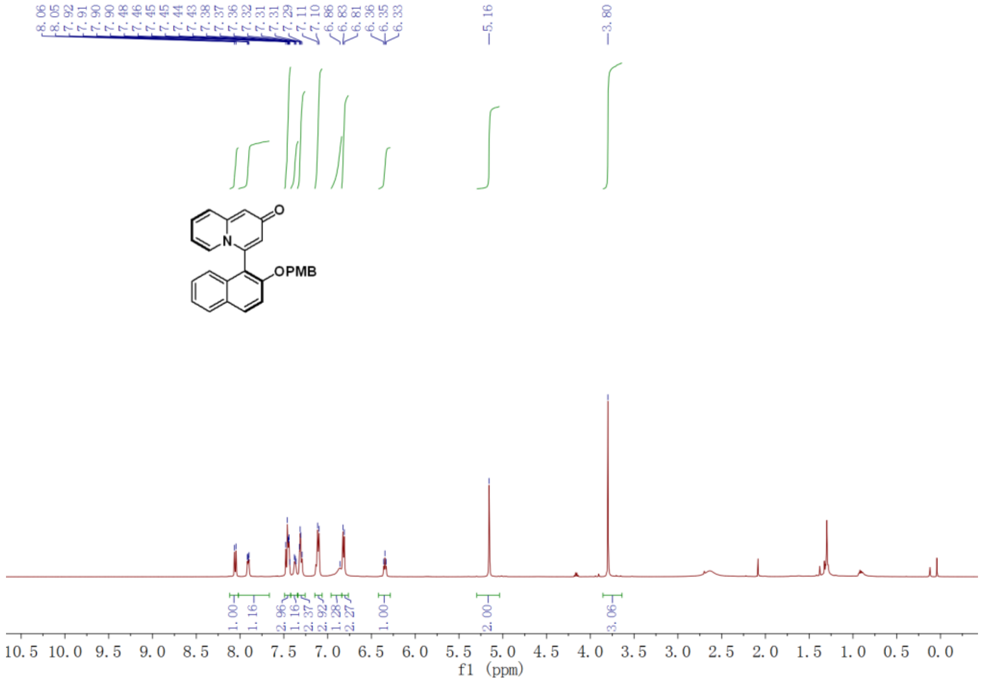

Supplementary Fig. 77. ^{1}H NMR spectrum of 2g.

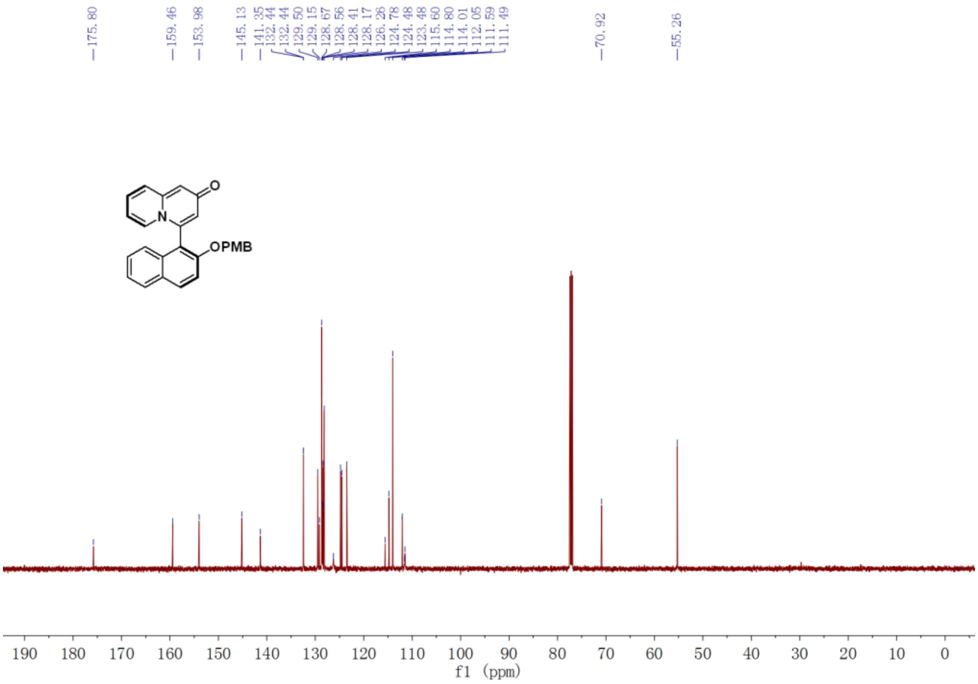

Supplementary Fig. 78. ^{13}C NMR spectrum of 2g.

2h, 4-(2-(dimethylamino)naphthalen-1-yl)-2*H*-quinolizin-2-one

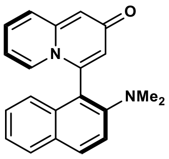

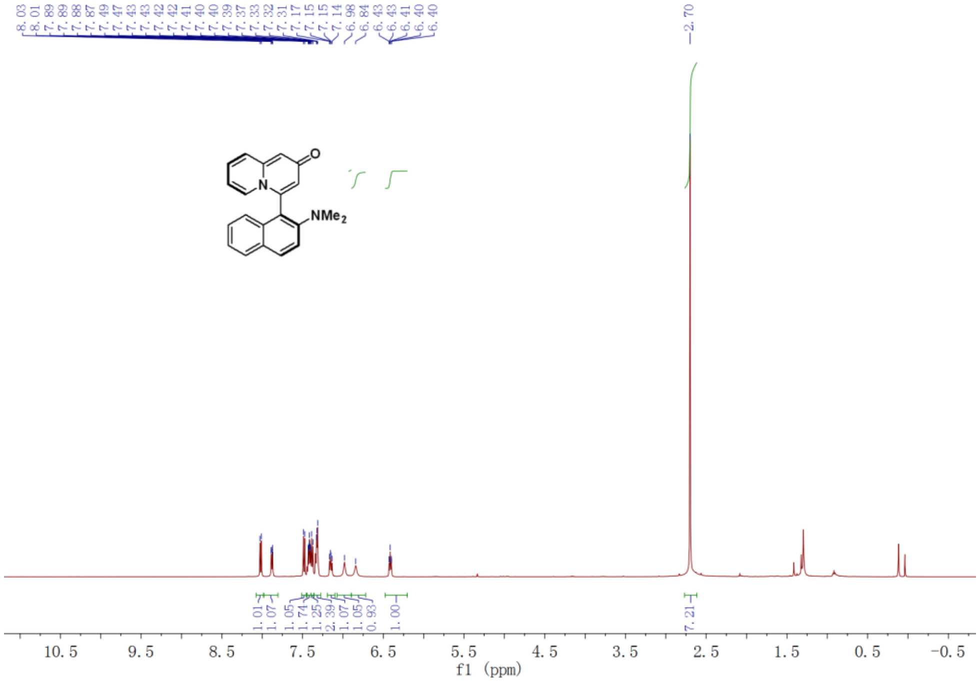

Supplementary Fig. 79. ^{1}H NMR spectrum of 2h.

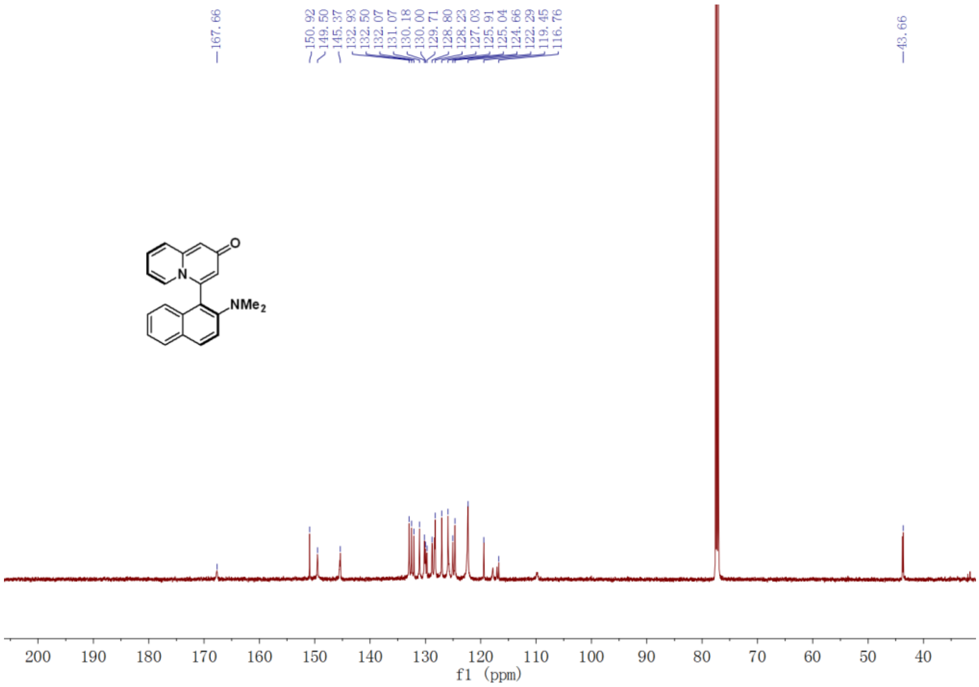

Supplementary Fig. 80. ^{13}C NMR spectrum of 2h.

2i, 4-(2-(dibenzylamino)naphthalen-1-yl)-2*H*-quinolizin-2-one

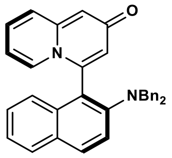

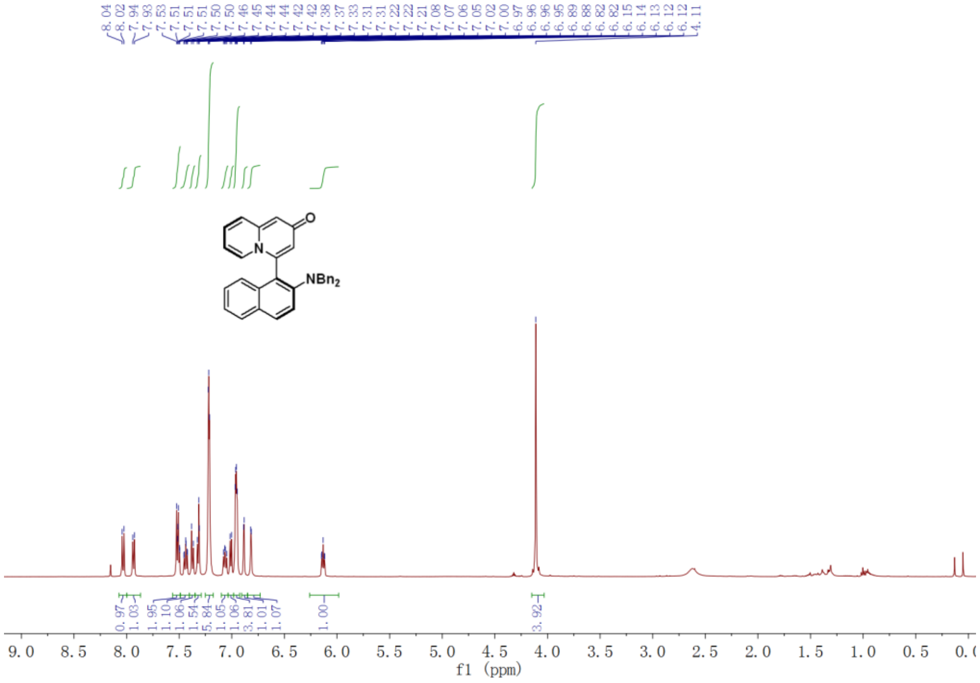

Supplementary Fig. 81. ^{1}H NMR spectrum of 2i.

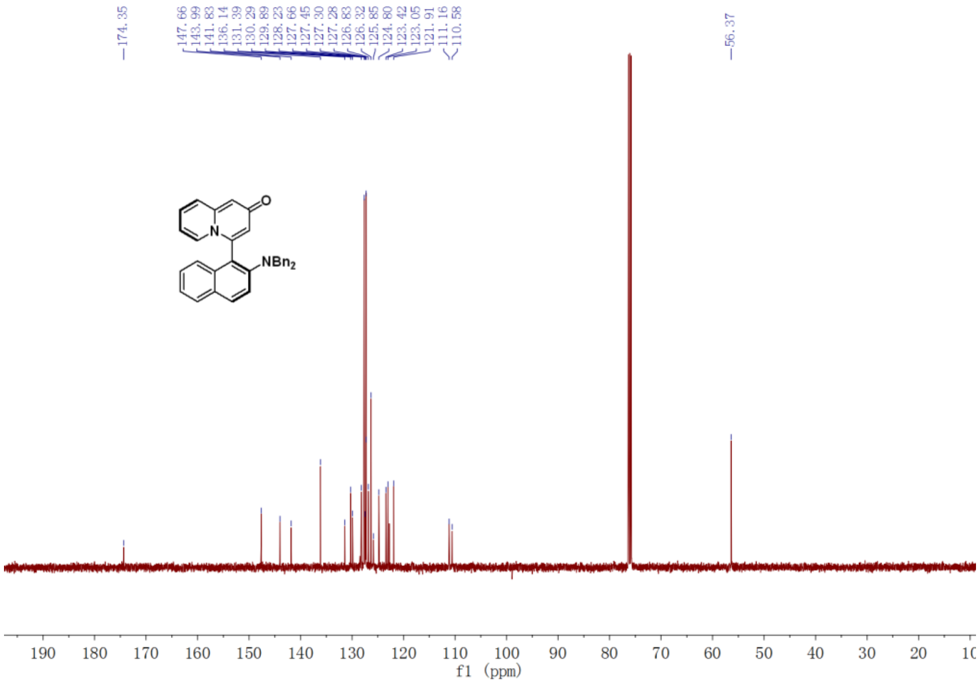

Supplementary Fig. 82. ^{13}C NMR spectrum of 2i.

2j, 4-(2-methylnaphthalen-1-yl)-2*H*-quinolizin-2-one

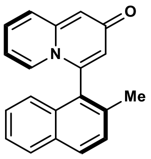

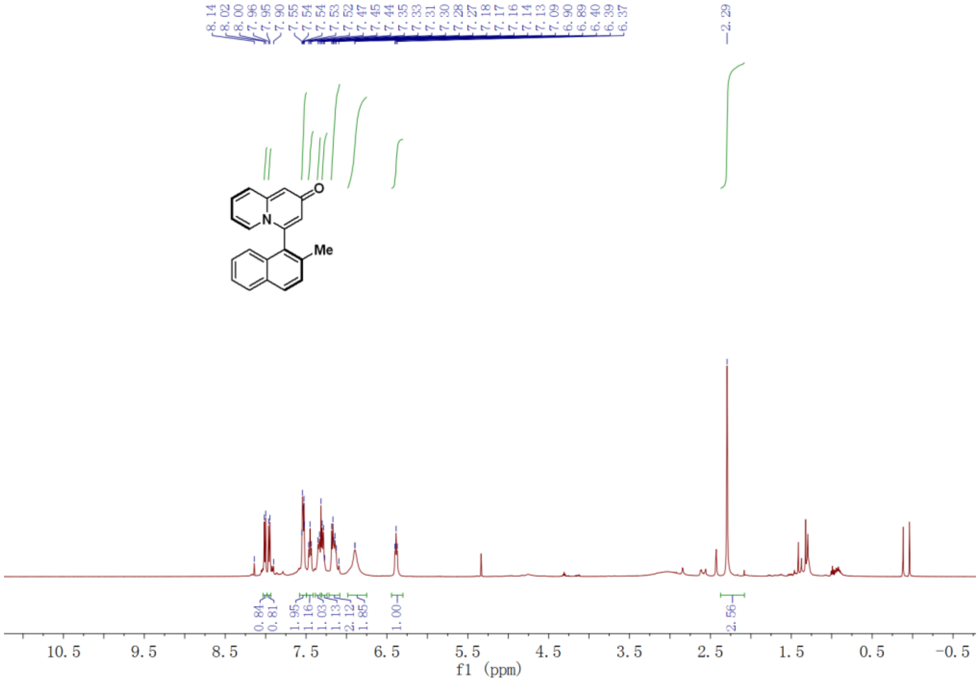

Supplementary Fig. 83. ^{1}H NMR spectrum of 2j.

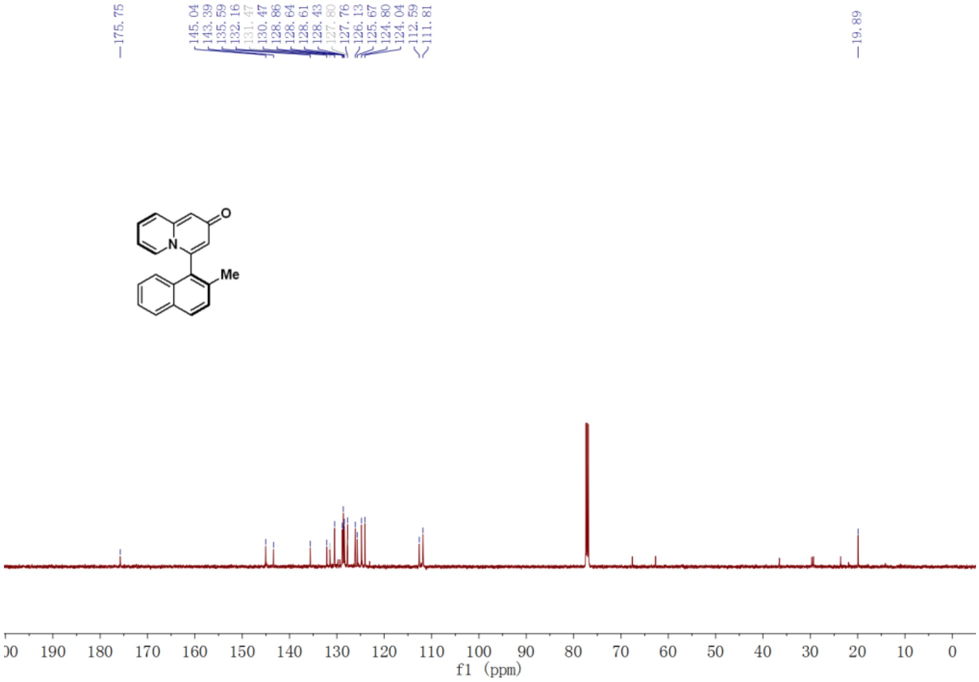

Supplementary Fig. 84. ^{13}C NMR spectrum of 2j.

2k, 4-(2-ethylnaphthalen-1-yl)-2*H*-quinolizin-2-one

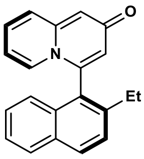

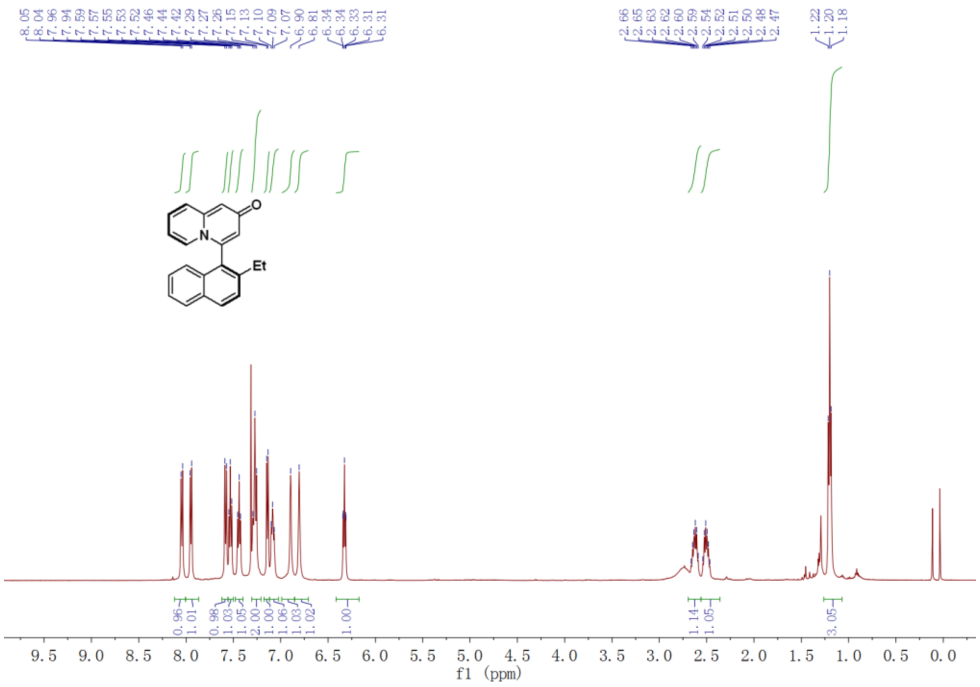

Supplementary Fig. 85. ^{1}H NMR spectrum of 2k.

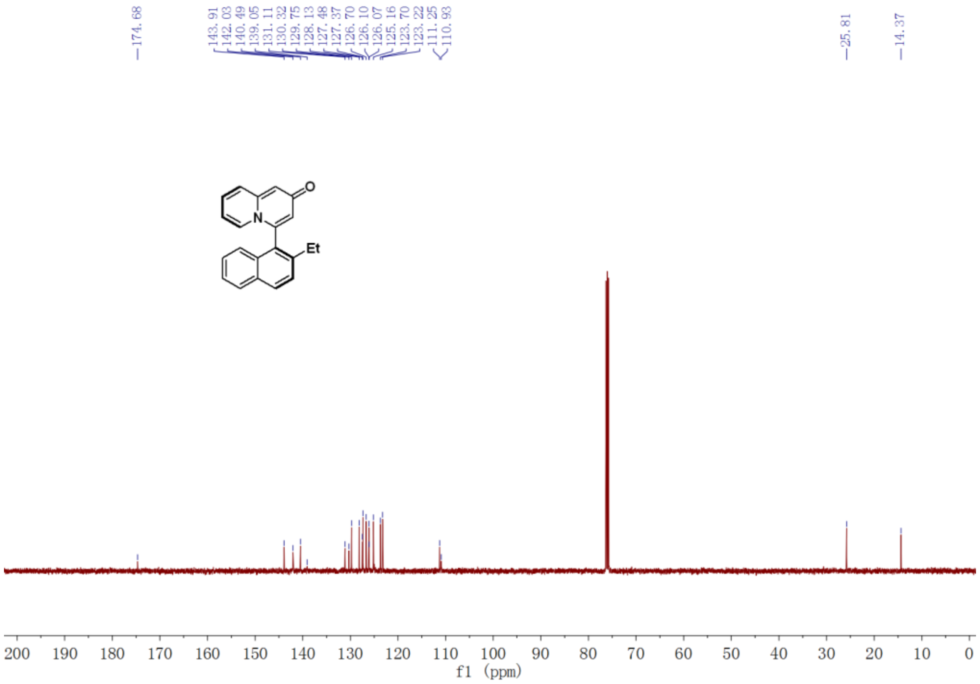

Supplementary Fig. 86. ^{13}C NMR spectrum of 2k.

2l, 4-(2-butylnaphthalen-1-yl)-2*H*-quinolizin-2-one

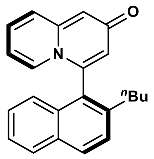

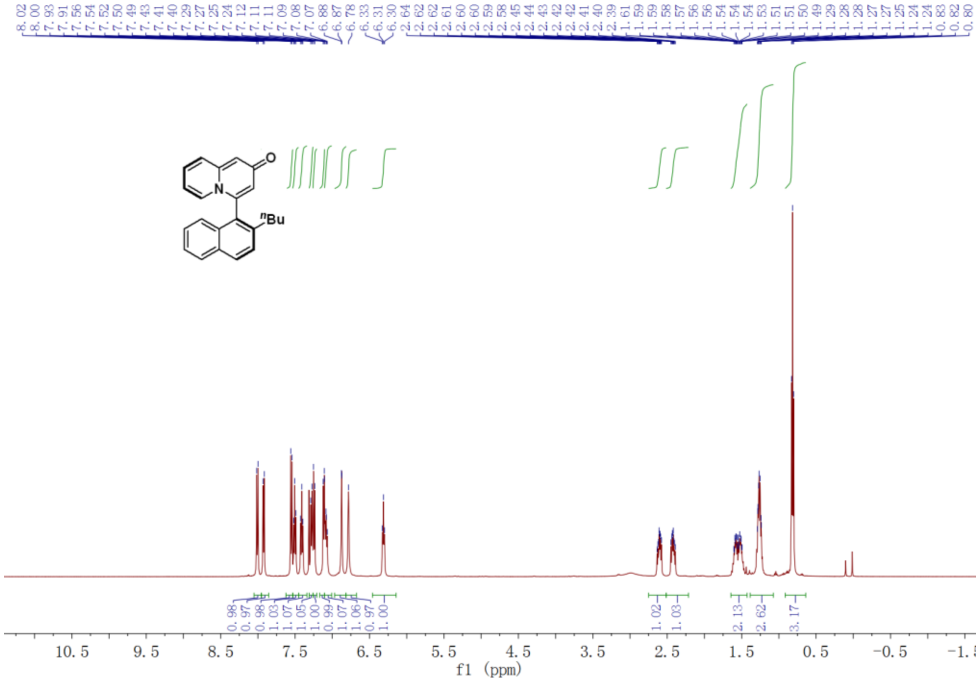

Supplementary Fig. 87. ^{1}H NMR spectrum of 2l.

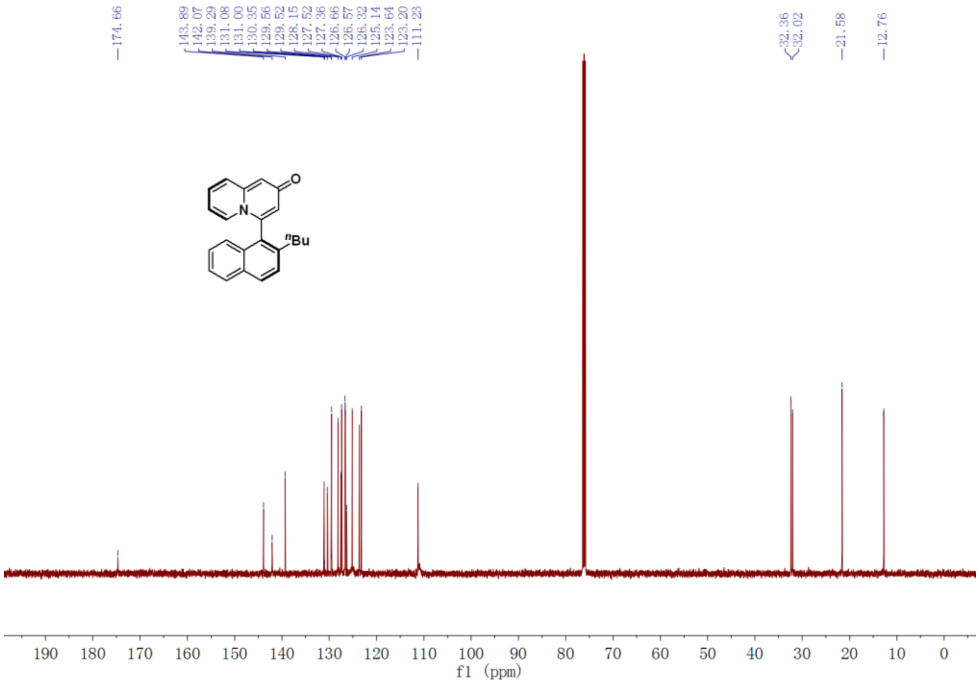

Supplementary Fig. 88. ^{13}C NMR spectrum of 2l.

2m, 4-(2-isopropylnaphthalen-1-yl)-2*H*-quinolizin-2-one

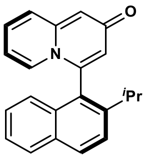

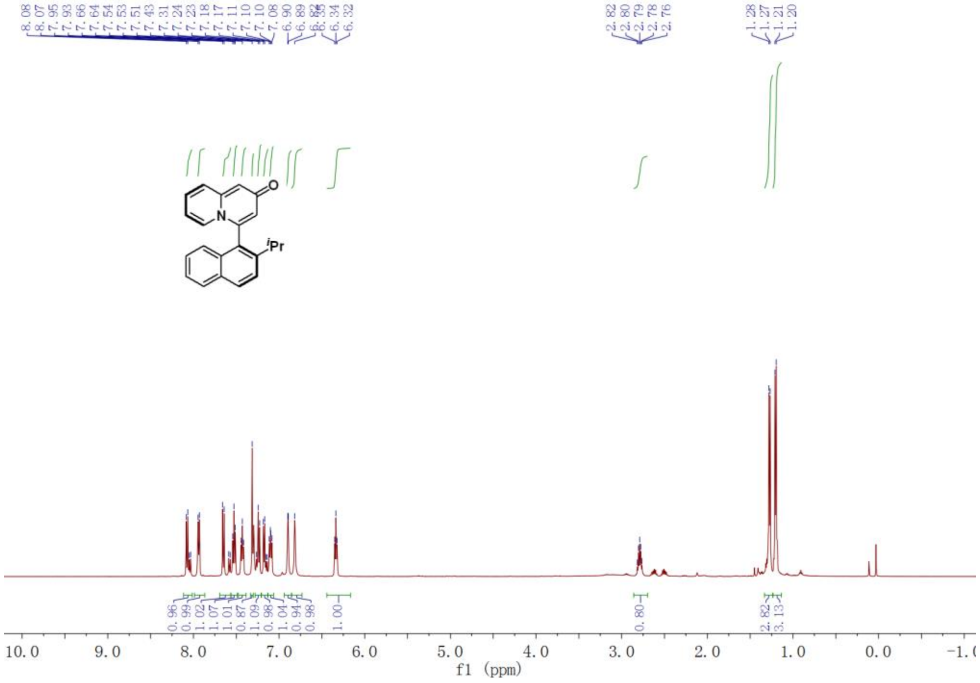

Supplementary Fig. 89. ^{1}H NMR spectrum of 2m.

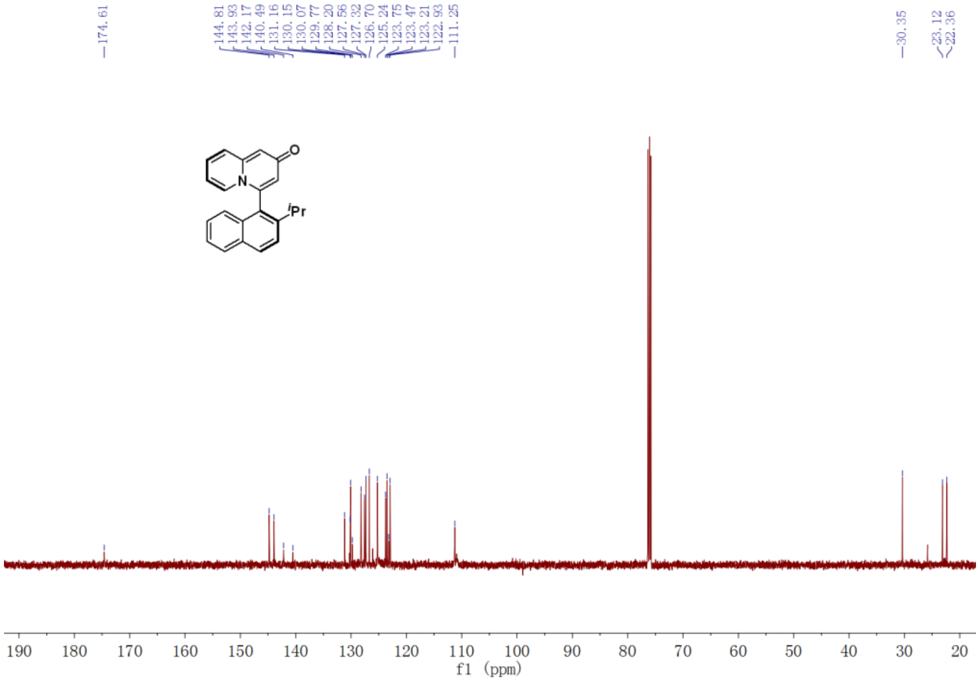

Supplementary Fig. 90. ^{13}C NMR spectrum of 2m.

2n, 4-(2-phenylnaphthalen-1-yl)-2*H*-quinolizin-2-one

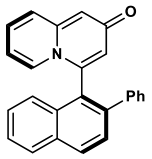

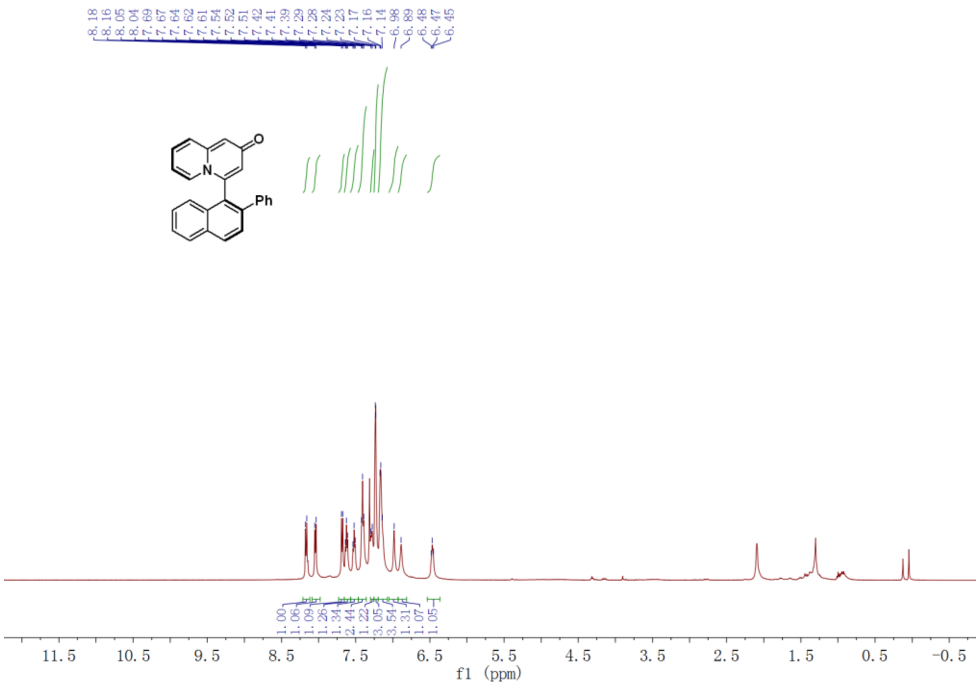

Supplementary Fig. 91. ^{1}H NMR spectrum of 2n.

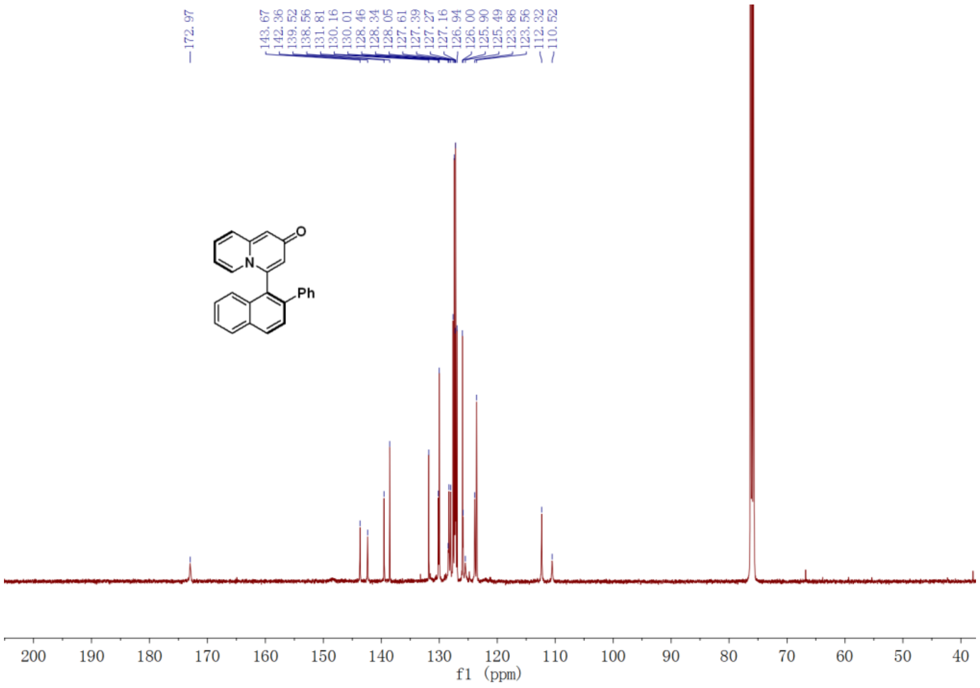

Supplementary Fig. 92. ^{13}C NMR spectrum of 2n.

2o, 4-([1,2'-binaphthalen]-1'-yl)-2*H*-quinolizin-2-one

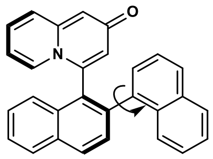

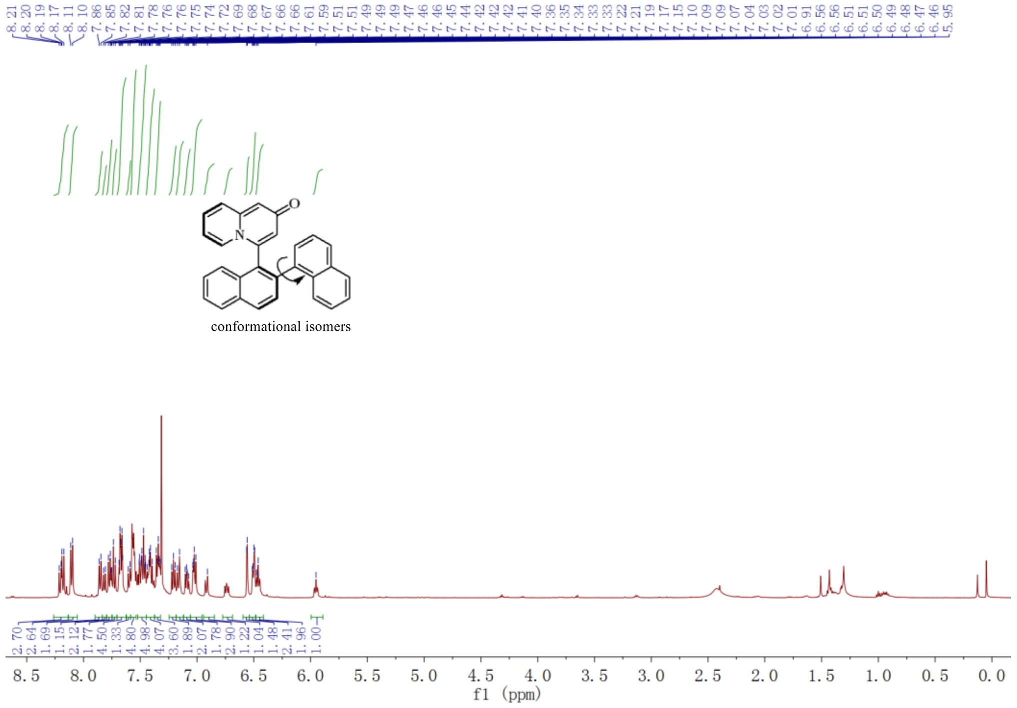

Supplementary Fig. 93. ^{1}H NMR spectrum of 2o.

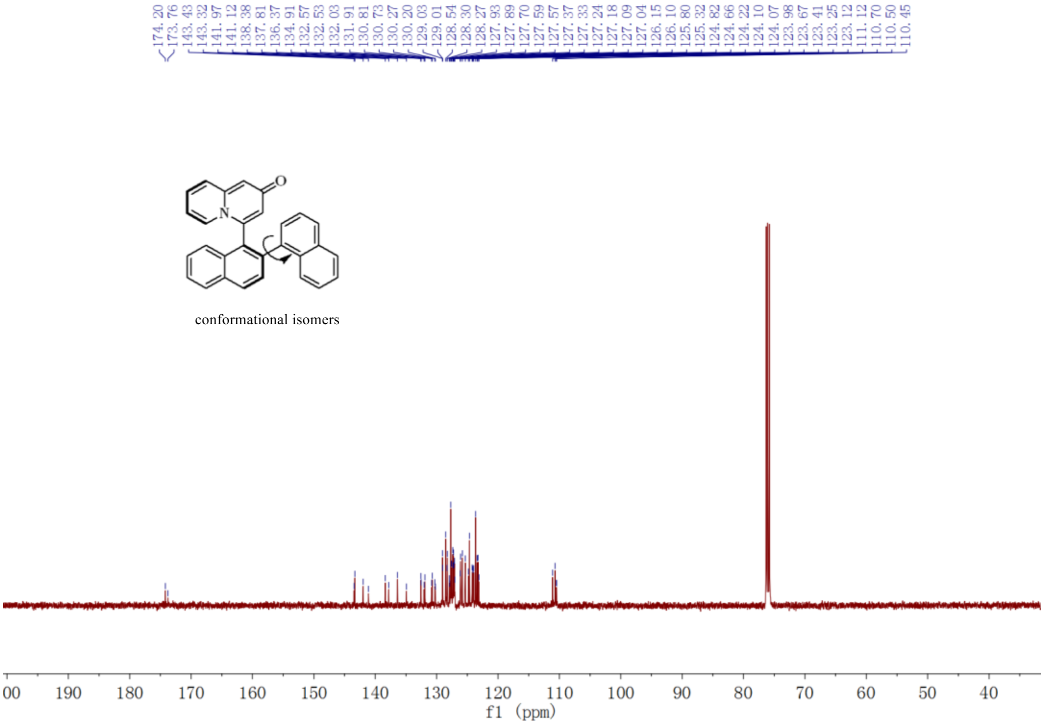

Supplementary Fig. 94. ^{13}C NMR spectrum of 2o.

2p, 4-(2-bromonaphthalen-1-yl)-2*H*-quinolizin-2-one

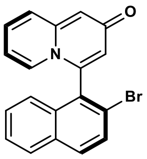

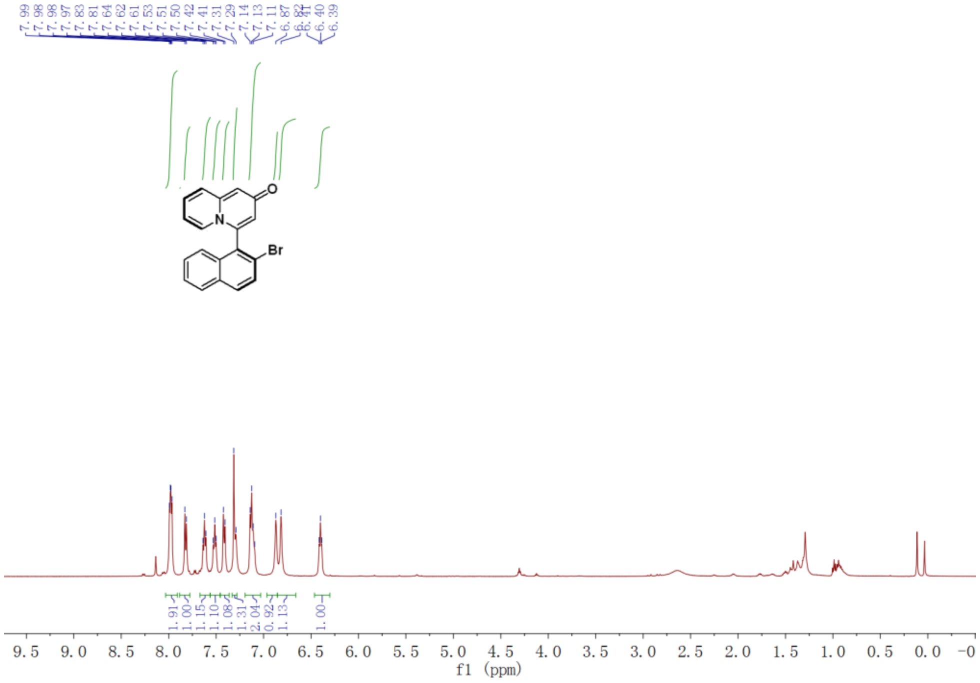

Supplementary Fig. 95. ^{1}H NMR spectrum of 2p.

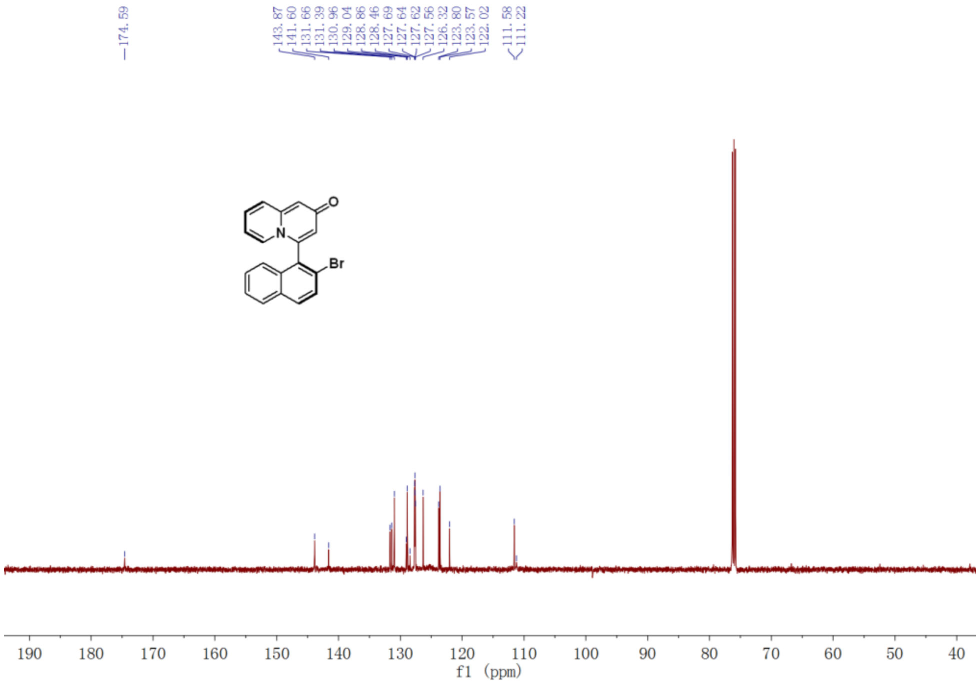

Supplementary Fig. 96. ^{13}C NMR spectrum of 2p.

2q, 4-(2-chloronaphthalen-1-yl)-2*H*-quinolizin-2-one

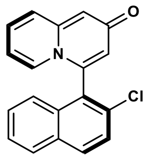

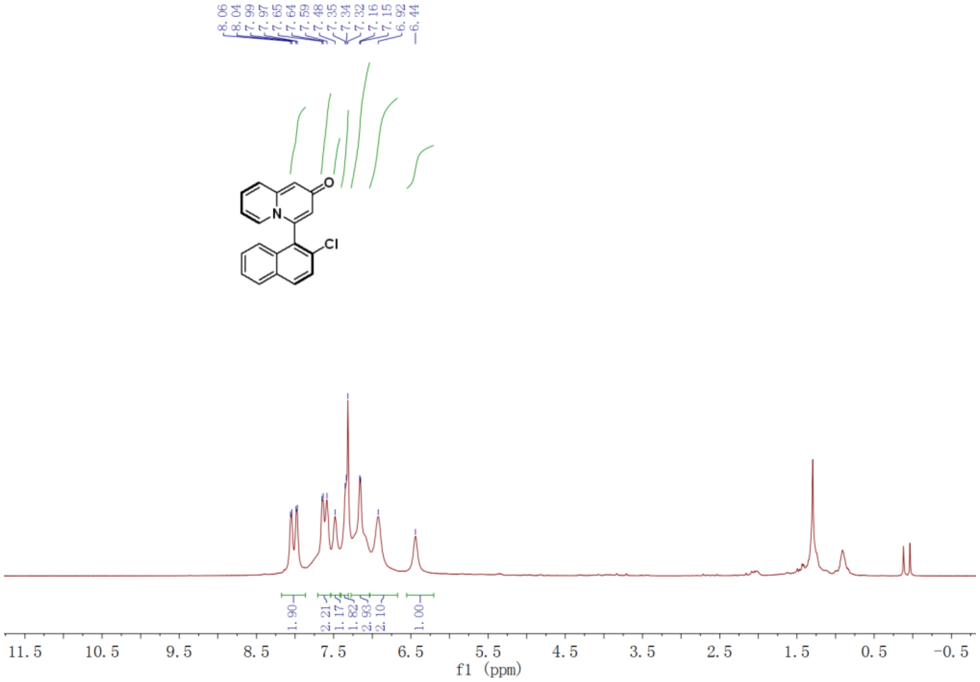

Supplementary Fig. 97. ^{1}H NMR spectrum of 2q.

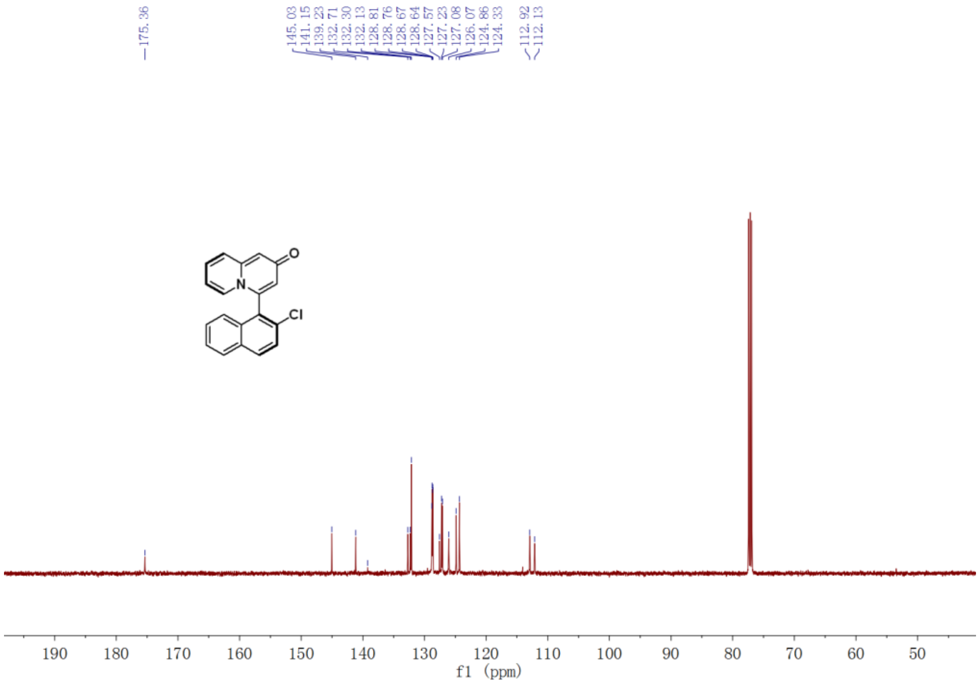

Supplementary Fig. 98. ^{13}C NMR spectrum of 2q.

2r, 9-bromo-4-(2-ethoxynaphthalen-1-yl)-2*H*-quinolizin-2-one

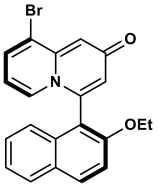

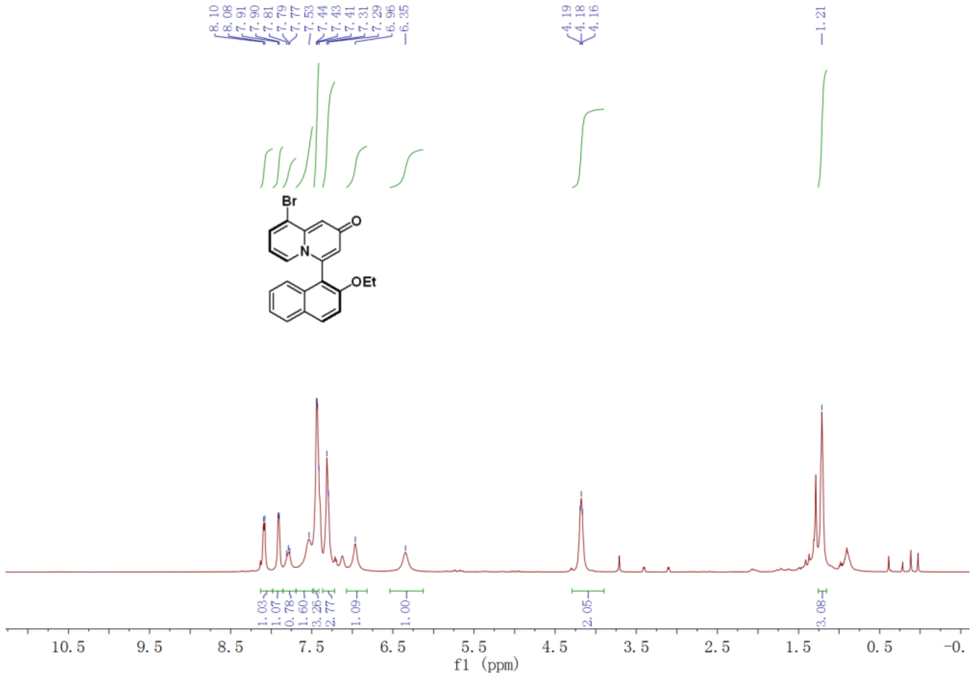

Supplementary Fig. 99. ^{1}H NMR spectrum of 2r.

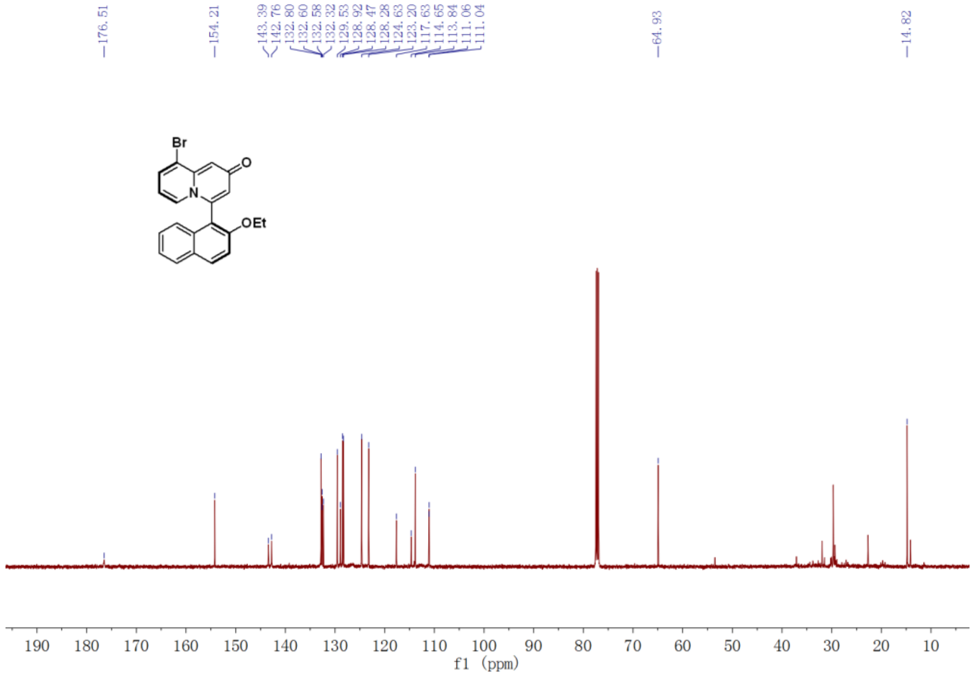

Supplementary Fig. 100. ^{13}C NMR spectrum of 2r.

2s, 8-bromo-4-(2-ethoxynaphthalen-1-yl)-2*H*-quinolizin-2-one

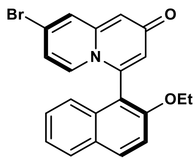

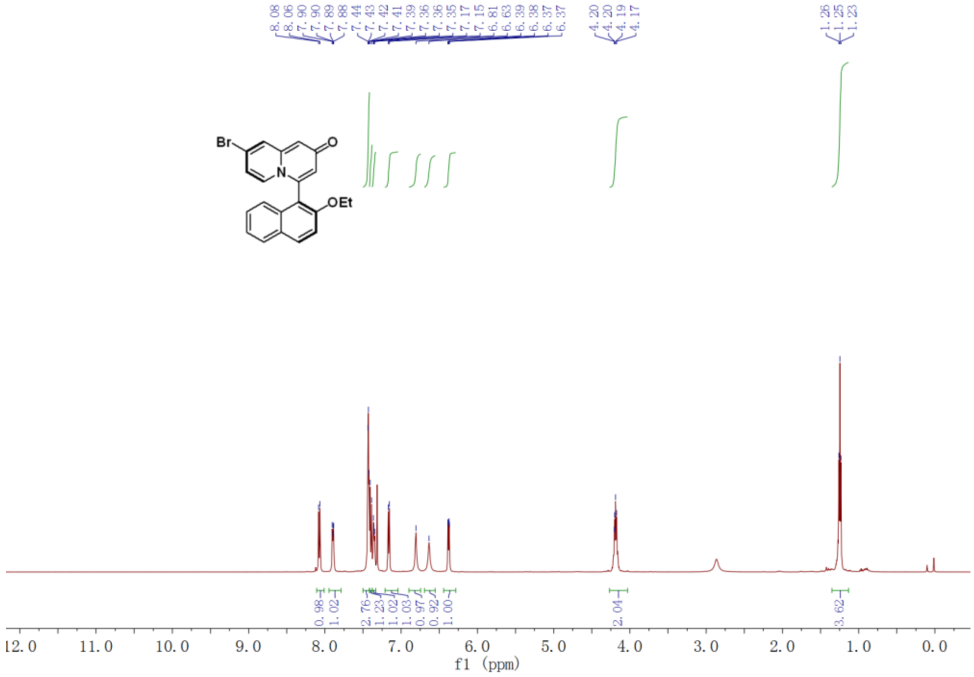

Supplementary Fig. 101. ¹H NMR spectrum of 2s.

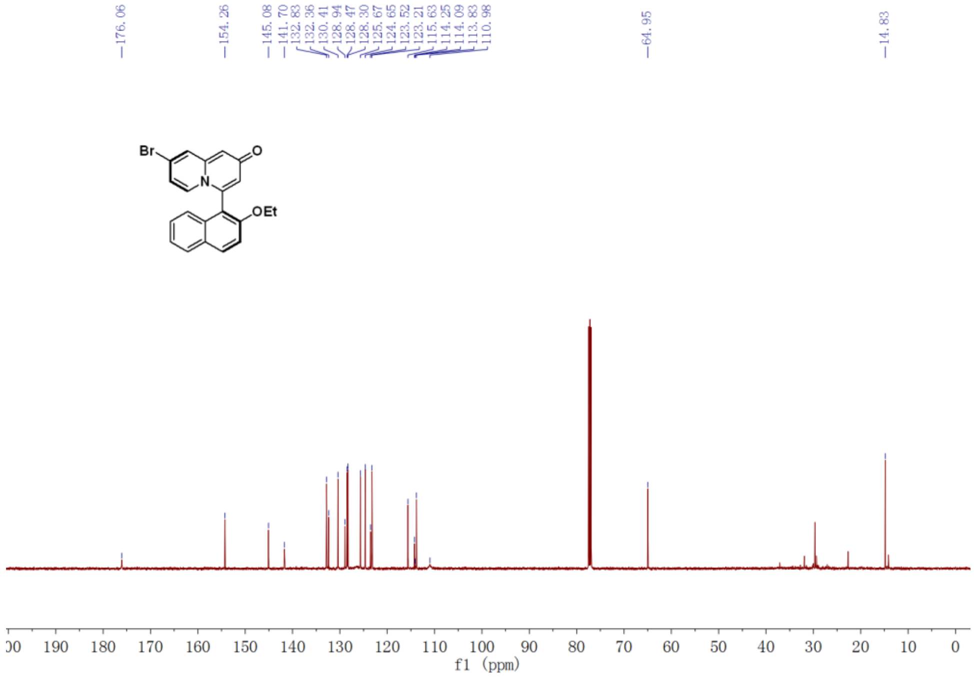

Supplementary Fig. 102. ¹³C NMR spectrum of 2s.

2t, 8-chloro-4-(2-ethoxynaphthalen-1-yl)-2*H*-quinolizin-2-one

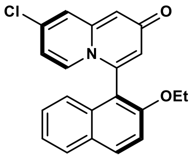

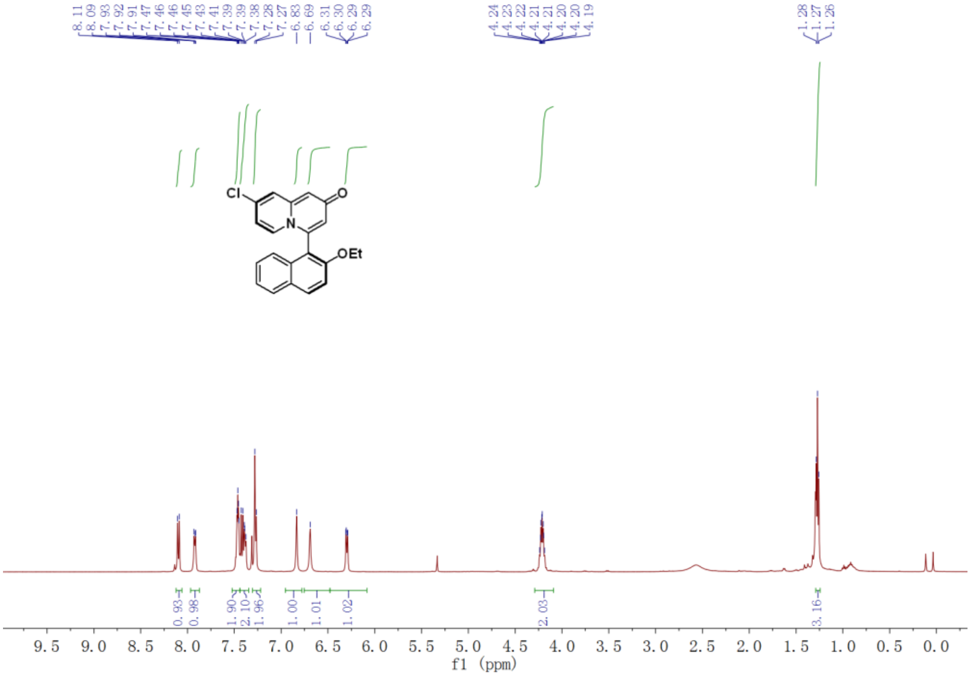

Supplementary Fig. 103. ^{1}H NMR spectrum of 2t.

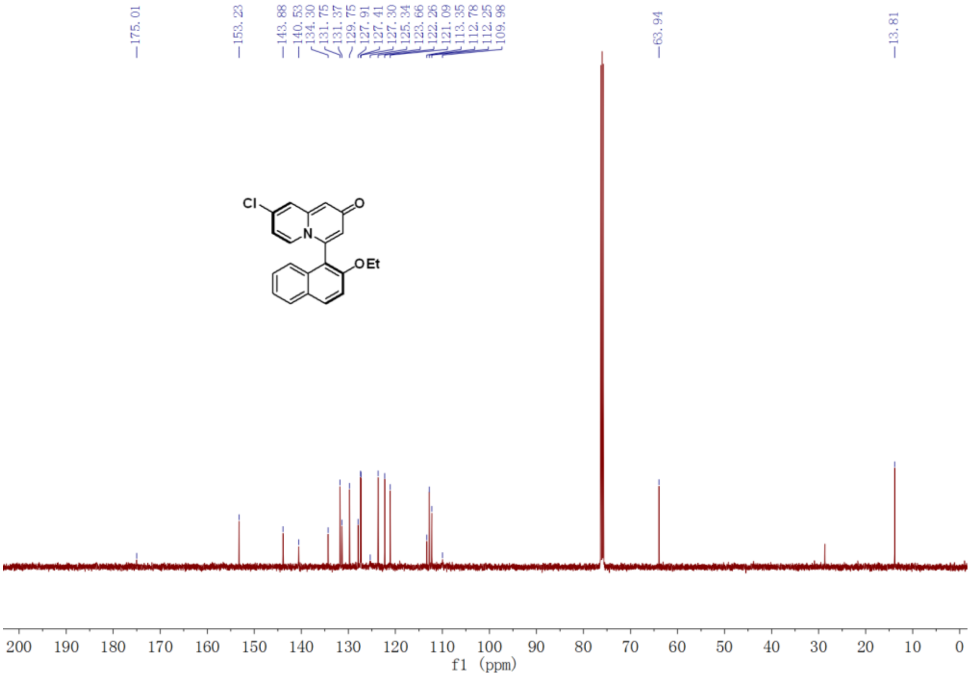

Supplementary Fig. 104. ^{13}C NMR spectrum of 2t.

2u, 7-bromo-4-(2-ethoxynaphthalen-1-yl)-2*H*-quinolizin-2-one

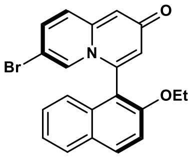

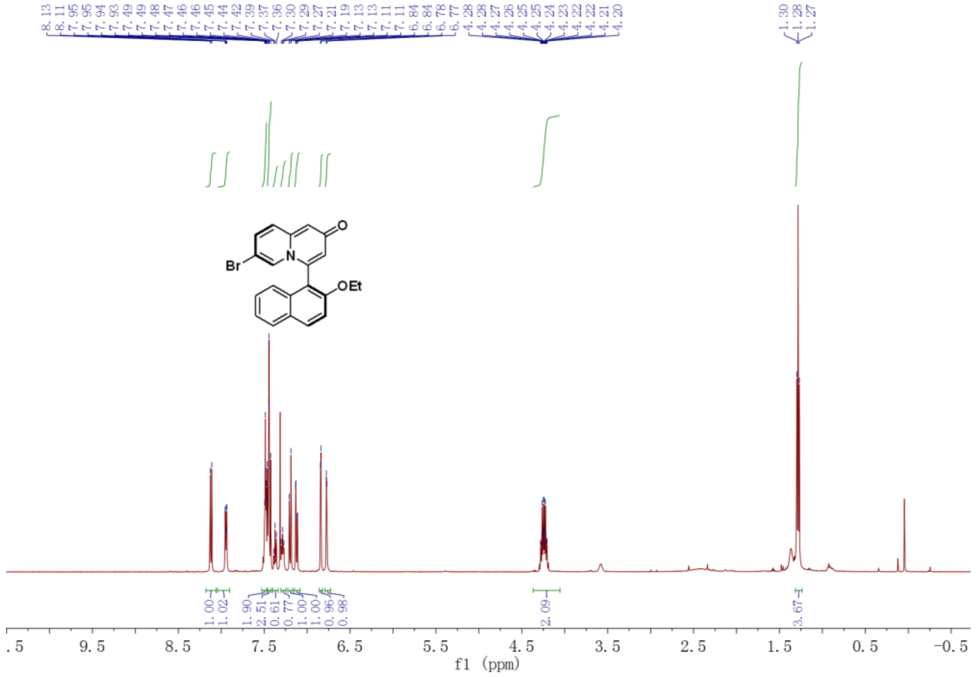

Supplementary Fig. 105. ^{1}H NMR spectrum of 2u.

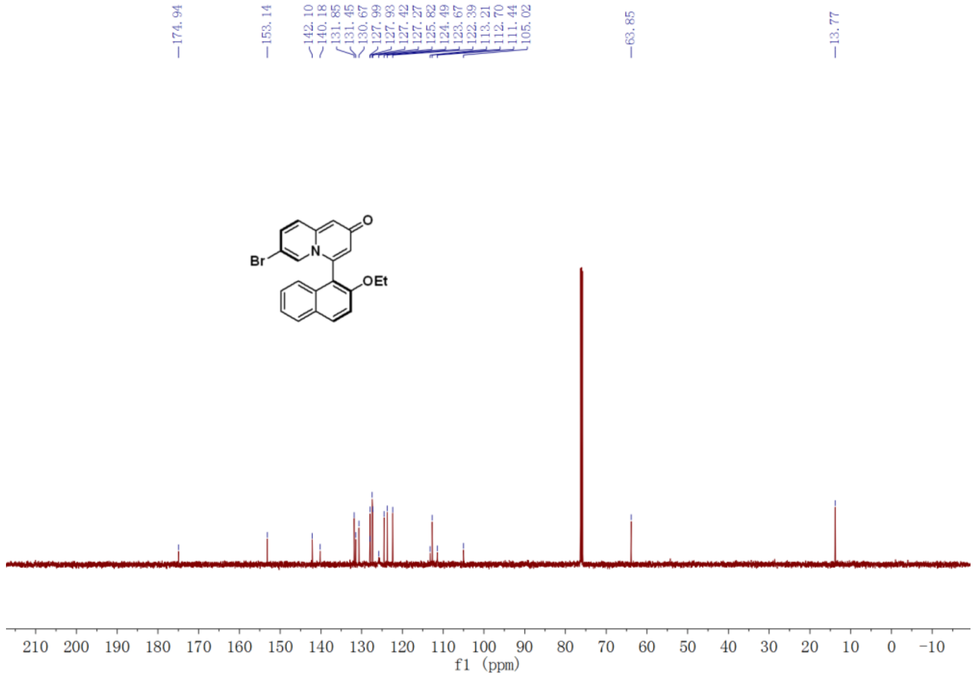

Supplementary Fig. 106. ^{13}C NMR spectrum of 2u.

2v, 4-(2-ethoxynaphthalen-1-yl)-7-methyl-2*H*-quinolizin-2-one

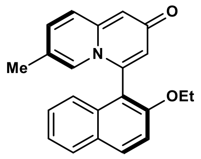

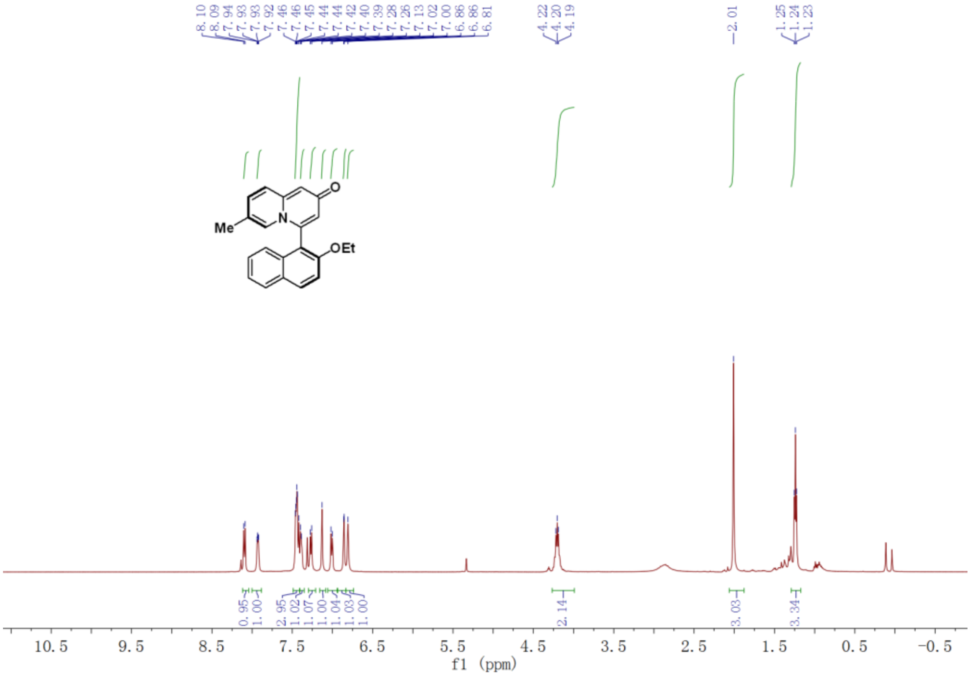

Supplementary Fig. 107. ^{1}H NMR spectrum of 2v.

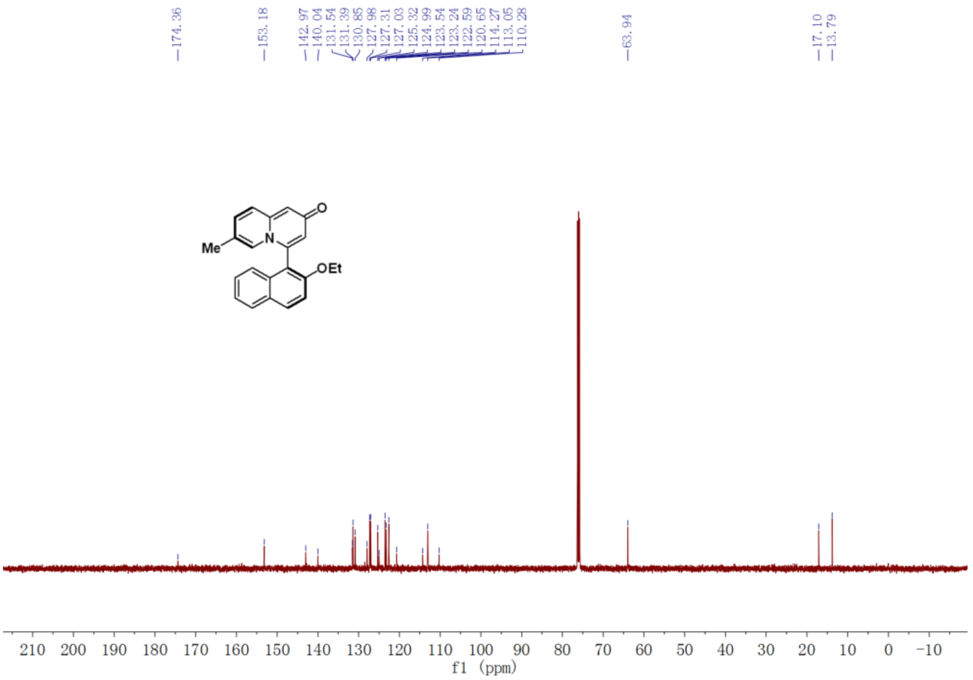

Supplementary Fig. 108. ^{13}C NMR spectrum of 2v.

2w, 6-(2-ethoxynaphthalen-1-yl)-8*H*-pyrido[1,2-*a*]pyrazin-8-one

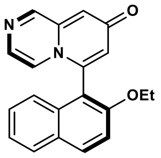

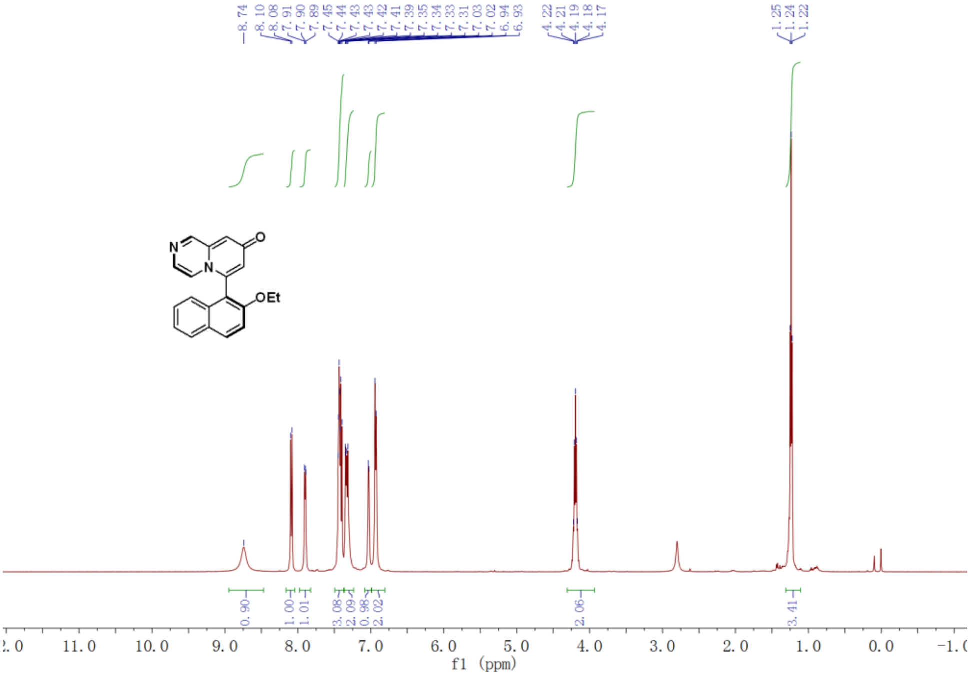

Supplementary Fig. 109. ^{1}H NMR spectrum of 2w.

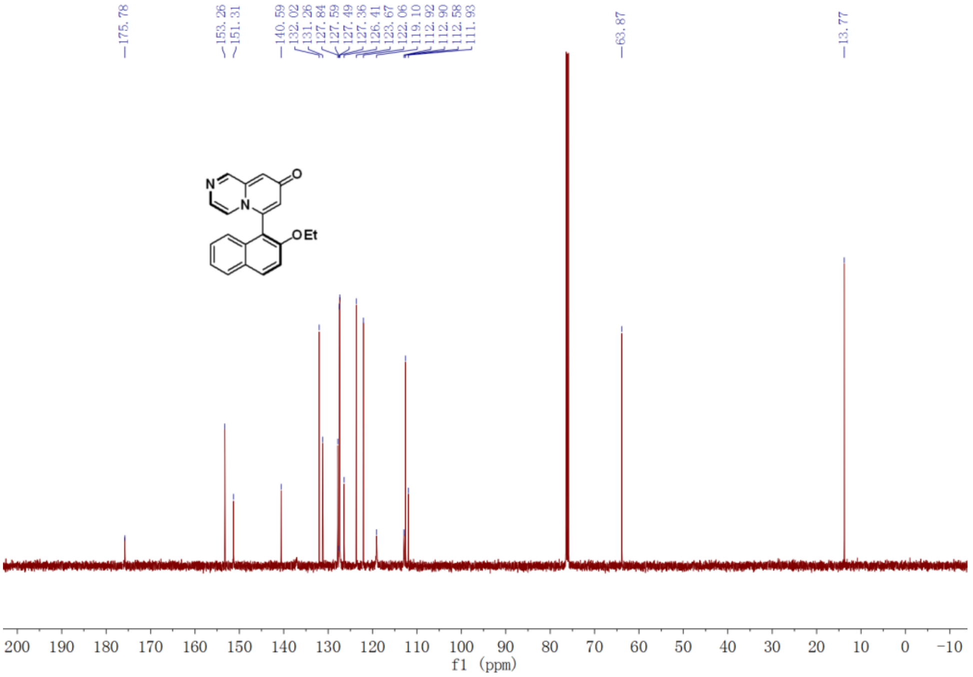

Supplementary Fig. 110. ^{13}C NMR spectrum of 2w.

2x, 4-(2-ethoxynaphthalen-1-yl)-7-(4-((((3a*S*,5a*R*,8a*R*,8b*S*)-2,2,7,7-Tetramethyltetrahydro-3a*H*-bis([1,3]dioxolo)[4,5-b:4',5'-d]pyran-3a-yl)methoxy)methyl)phenyl)-2*H*-quinolizin-2-one

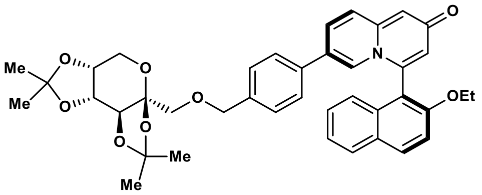

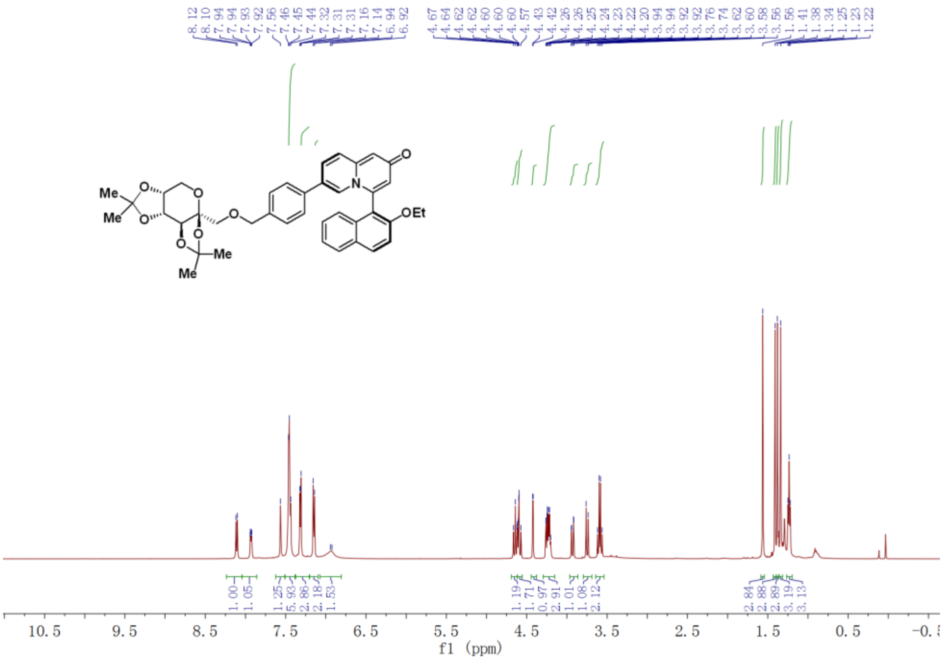

Supplementary Fig. 111. ^{1}H NMR spectrum of 2x.

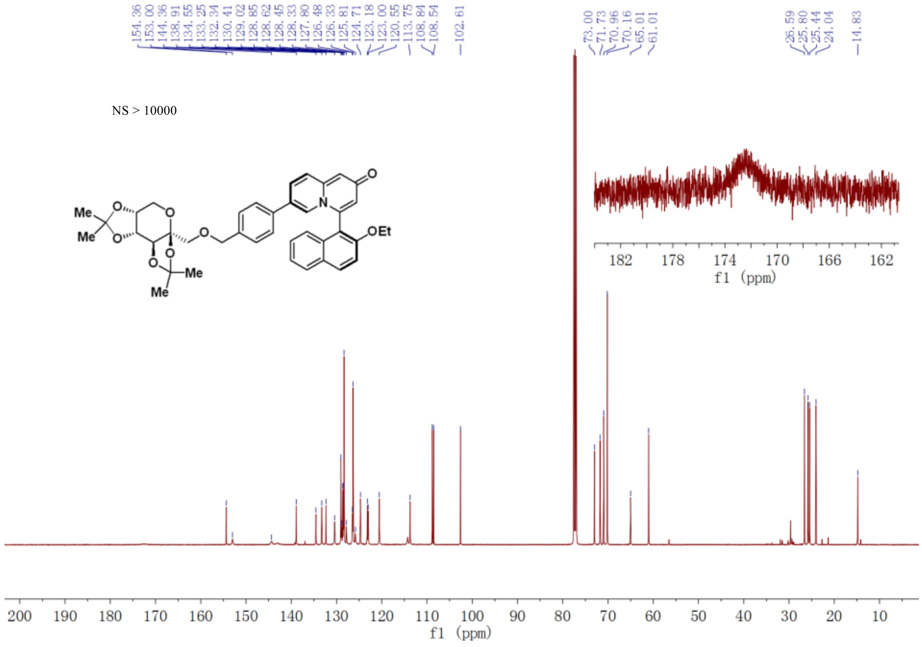

Supplementary Fig. 112. ^{13}C NMR spectrum of 2x.

2y, 4-(2-ethoxynaphthalen-1-yl)-7-(4-((((*R*)-2,5,7,8-tetramethyl-2-((4*S*, 8*R*)-4,8,12-trimethyltridec-yl)chroman-6-yl)oxy)methyl)phenyl)-2*H*-quinolizin-2-one

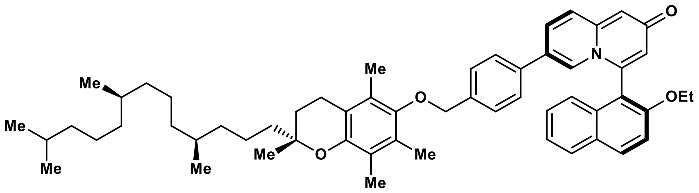

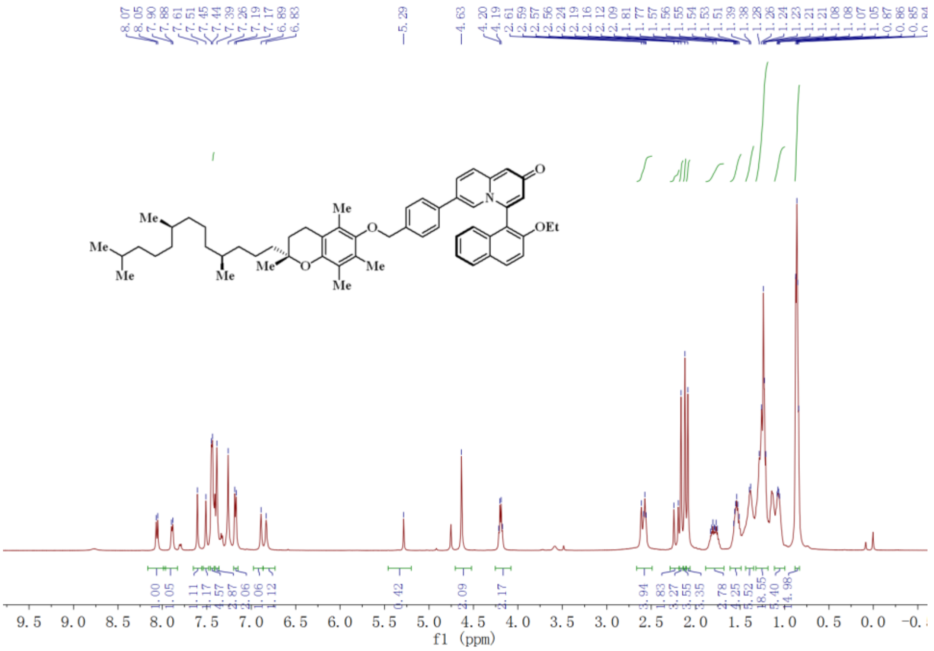

Supplementary Fig. 113. ^{1}H NMR spectrum of 2y.

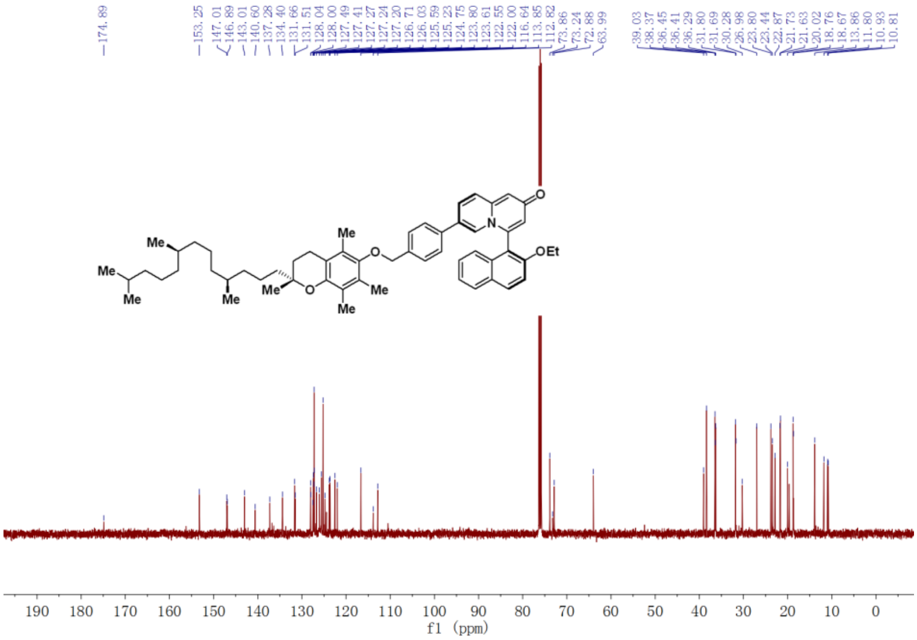

Supplementary Fig. 114. ^{13}C NMR spectrum of 2y.

2z, 4-(2-ethoxynaphthalen-1-yl)-7-(4-((((1*R*,2*S*,5*R*)-2-isopropyl-5-methyl -cyclohexyl)oxy)methyl) -phenyl)-2*H*-quinolizin-2-one

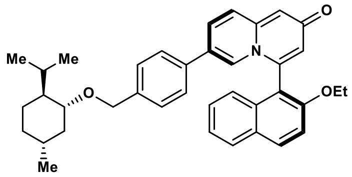

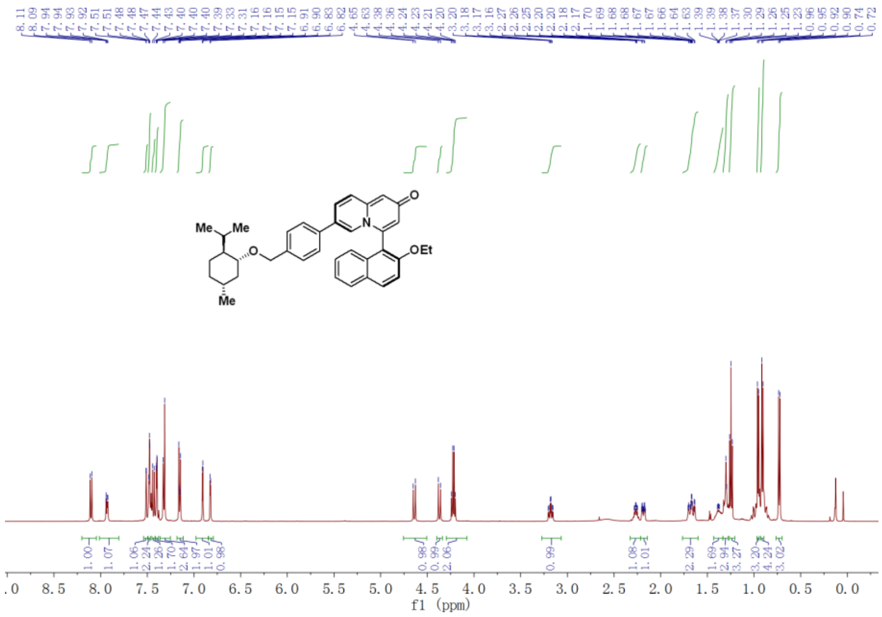

Supplementary Fig. 115. ^{1}H NMR spectrum of 2z.

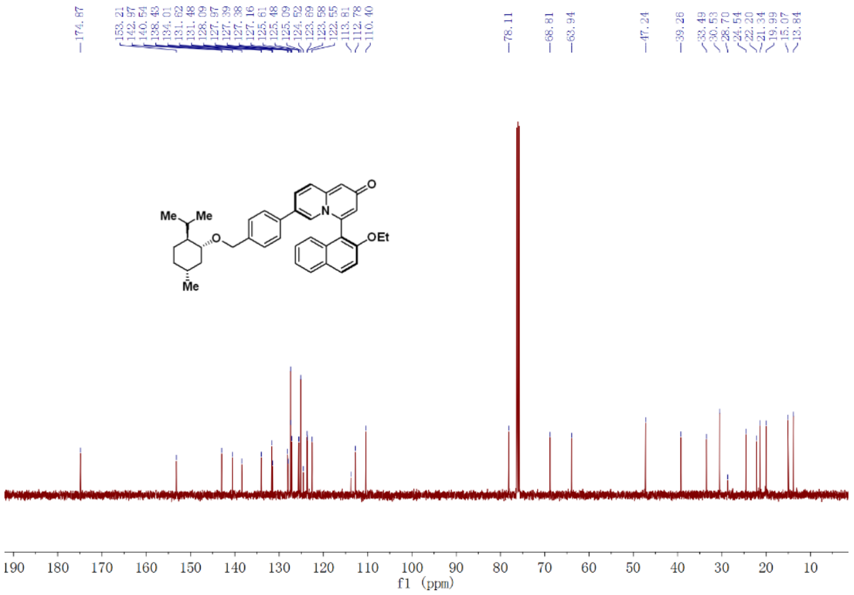

Supplementary Fig. 116. ^{13}C NMR spectrum of 2z.

3, di-*tert*-butyl 1-(4-(2-ethoxynaphthalen-1-yl)-2-oxo-2*H*-quinolizin-1-yl) -hydrazine-1,2 -dicarboxylate

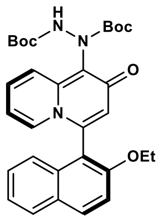

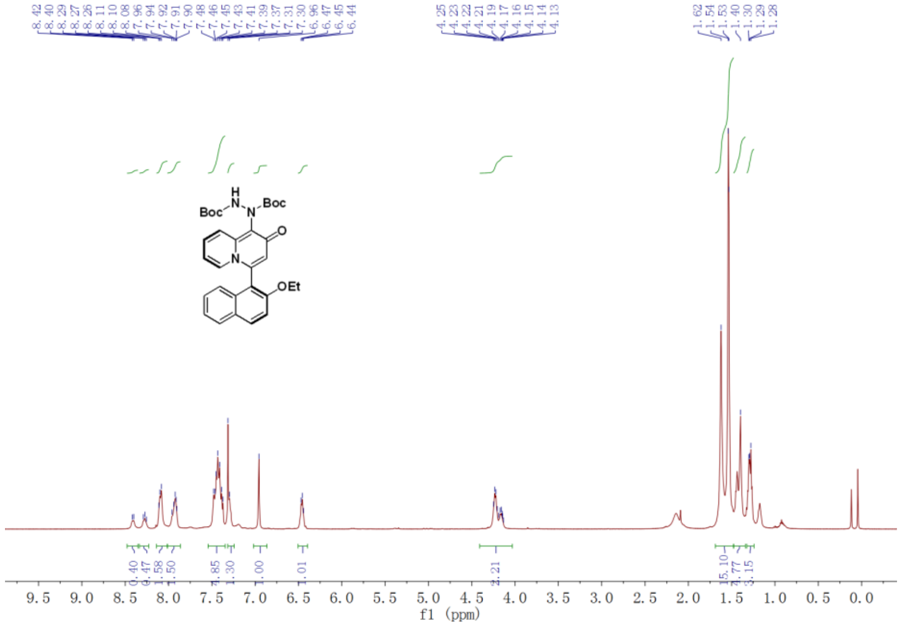

Supplementary Fig. 117. ^{1}H NMR spectrum of 3.

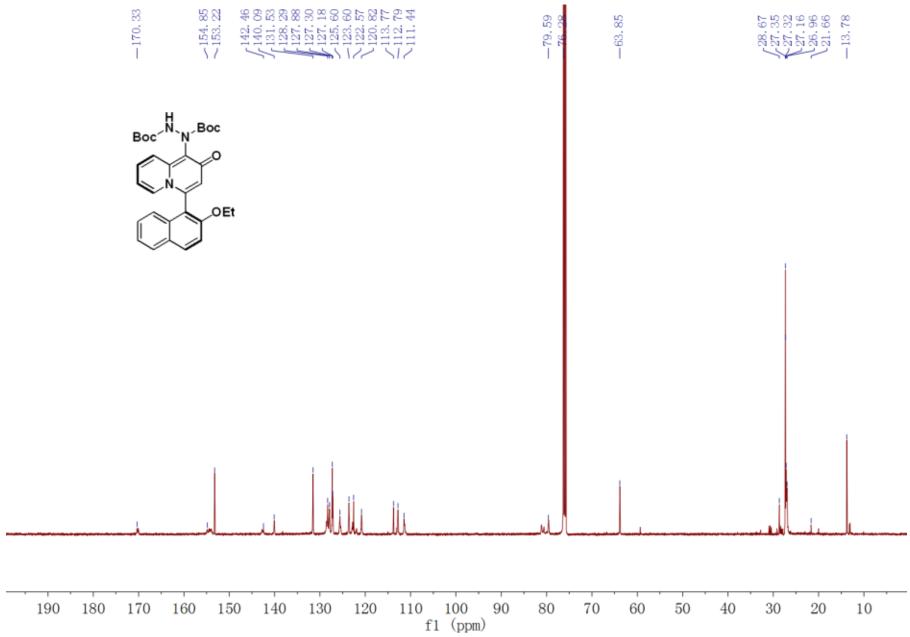

Supplementary Fig. 118. ^{13}C NMR spectrum of 3.

4, 4-(2-hydroxynaphthalen-1-yl)-2*H*-quinolizin-2-one

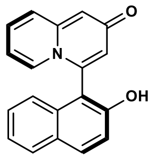

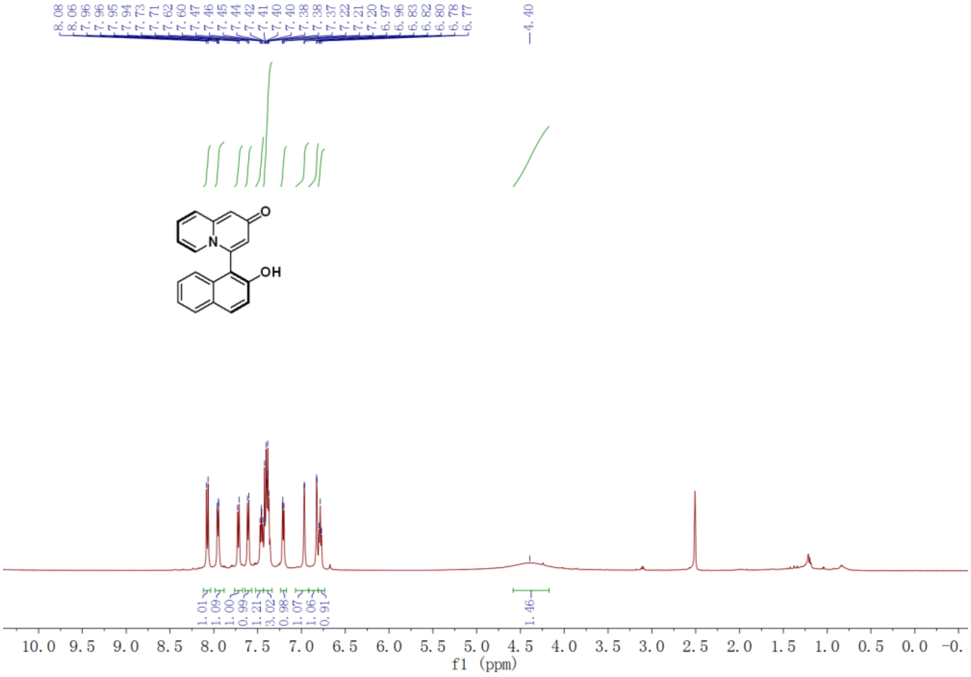

Supplementary Fig. 119. ^{1}H NMR spectrum of 4.

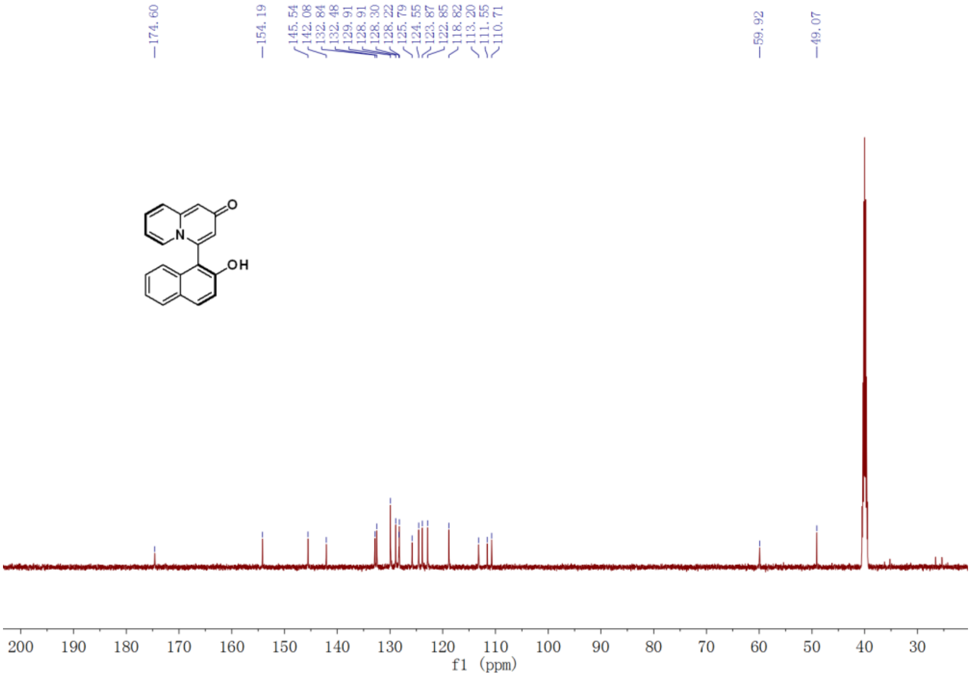

Supplementary Fig. 120. ^{13}C NMR spectrum of 4.

5, 4-(2-((diphenylphosphanyl)oxy)naphthalen-1-yl)-2*H*-quinolizin-2-one

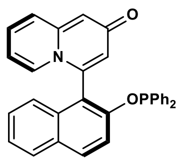

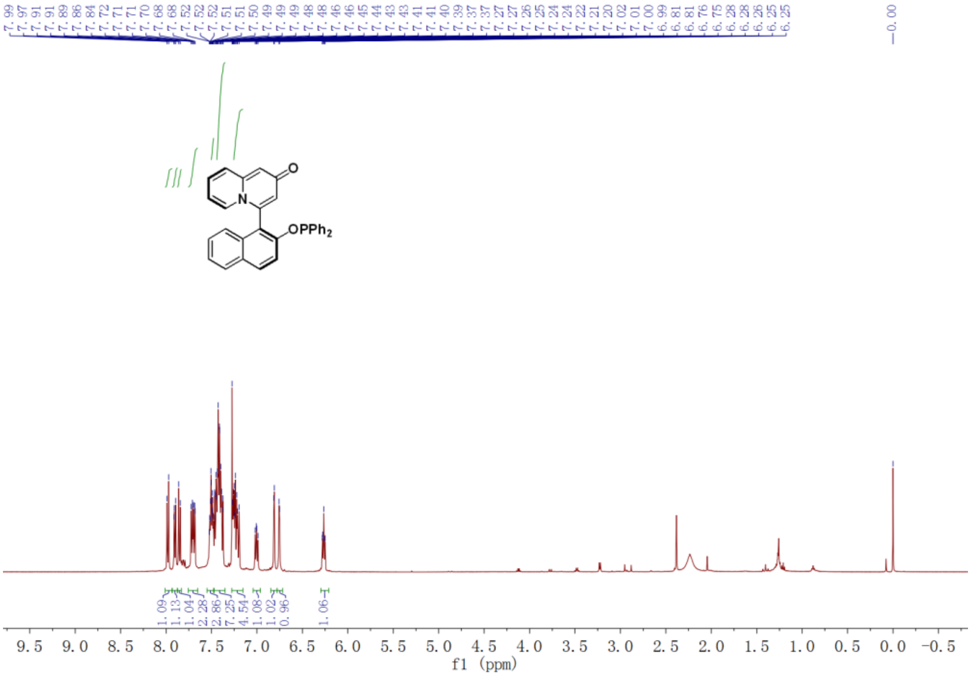

Supplementary Fig. 121. ^{1}H NMR spectrum of 5.

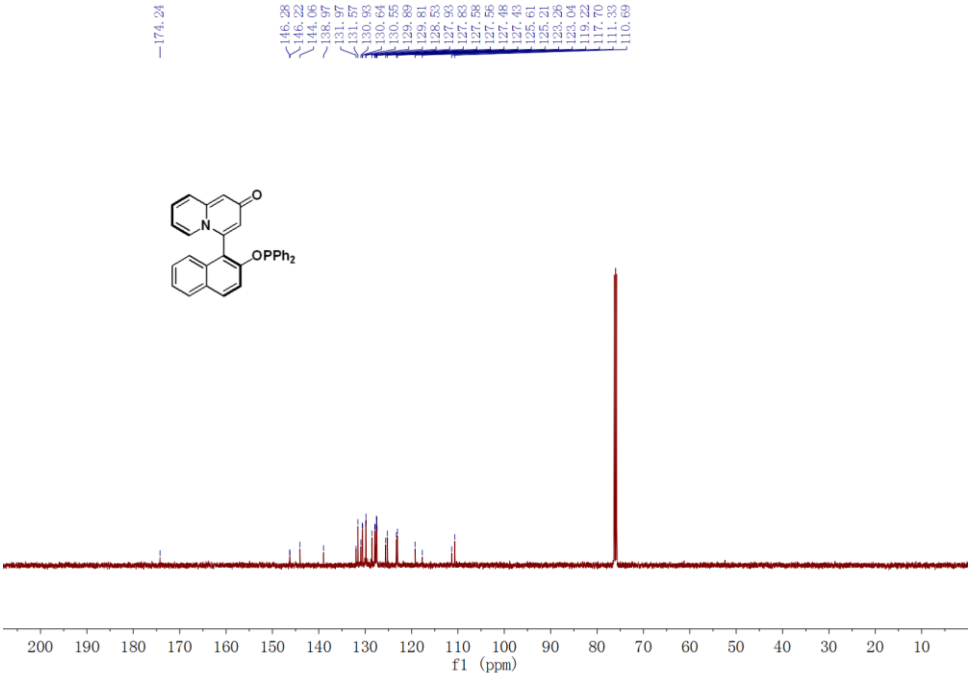

Supplementary Fig. 122. ^{13}C NMR spectrum of 5.

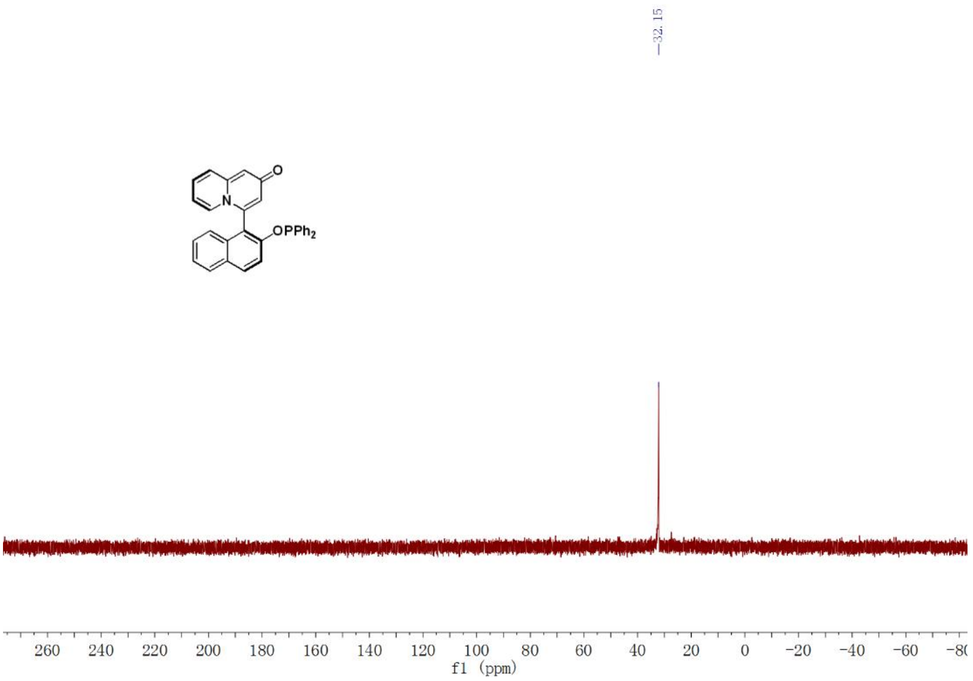

Supplementary Fig. 123. ^{31}P NMR spectrum of 5.

6, 1-(2-oxo-2*H*-quinolizin-4-yl)naphthalen-2-yl trifluoromethanesulfonate

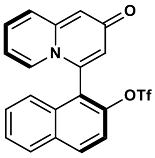

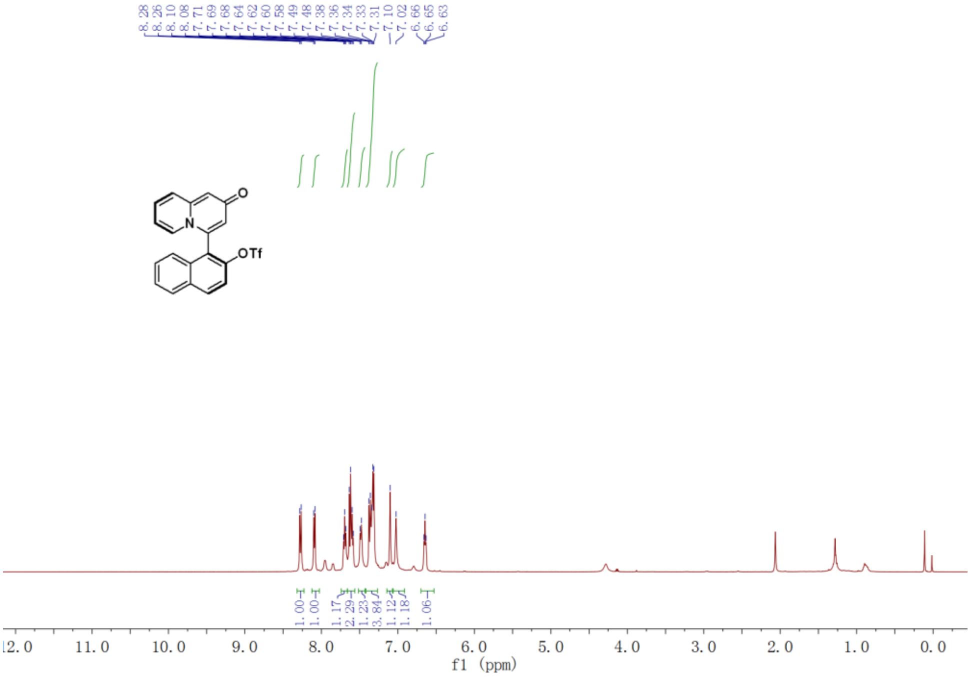

Supplementary Fig. 124. ^{1}H NMR spectrum of 6.

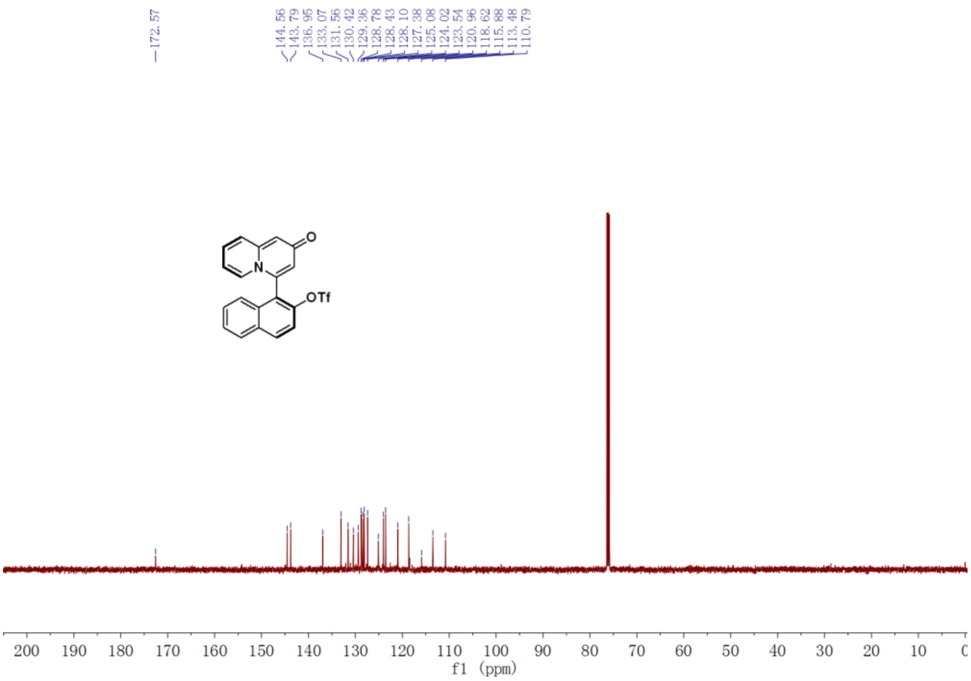

Supplementary Fig. 125. ^{13}**C NMR spectrum of 6.**

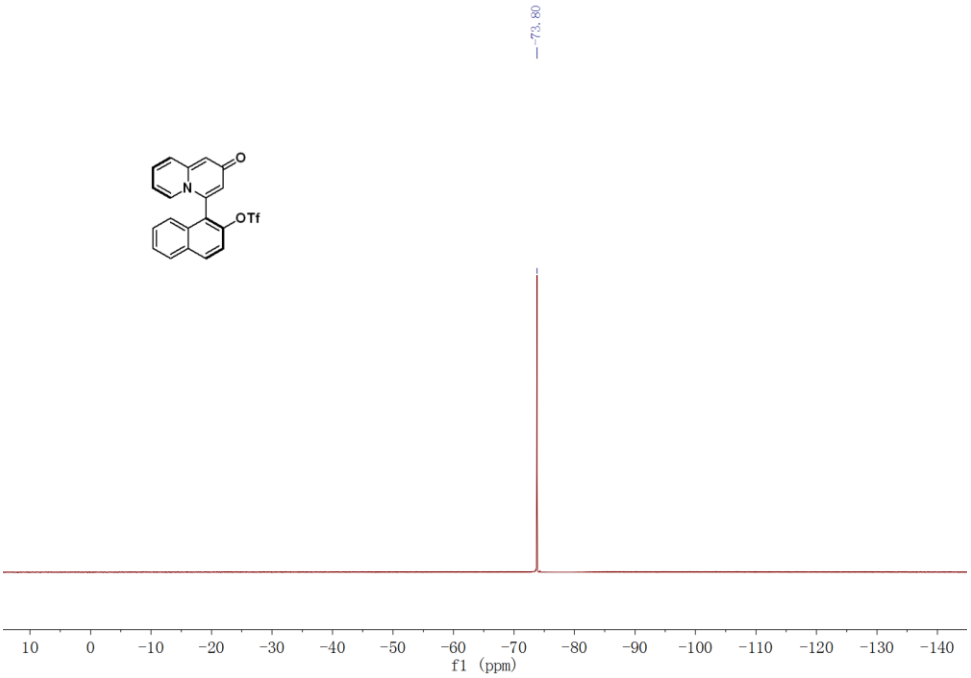

Supplementary Fig. 126. ^{19}**F NMR spectrum of 6.**

7, 4-(2-(benzylamino)naphthalen-1-yl)-2*H*-quinolizin-2-one

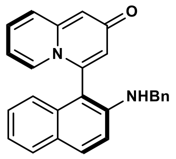

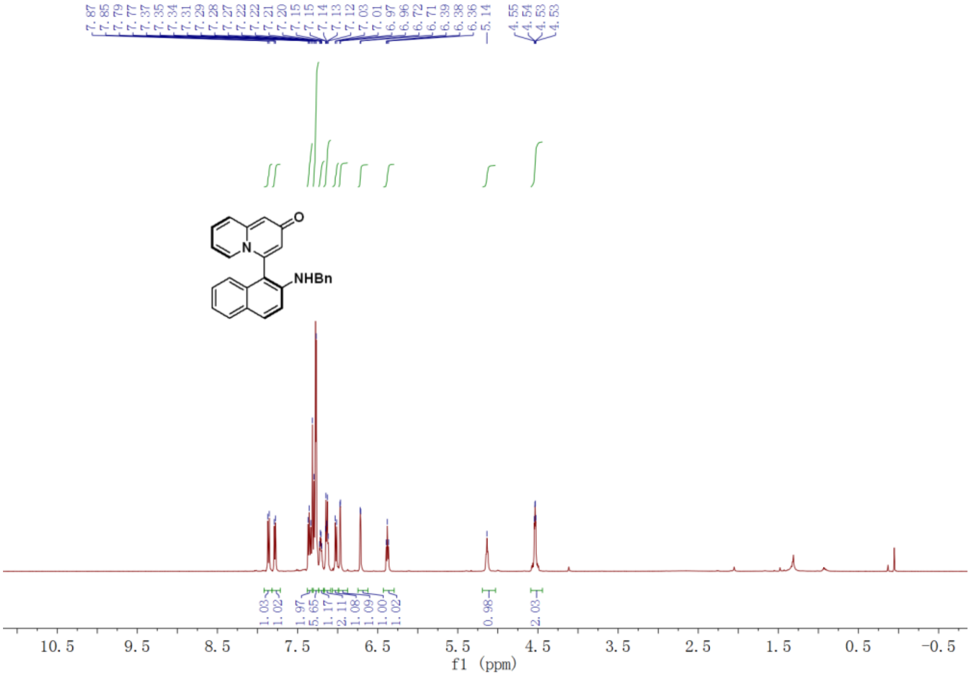

Supplementary Fig. 127. ^{1}H NMR spectrum of 7.

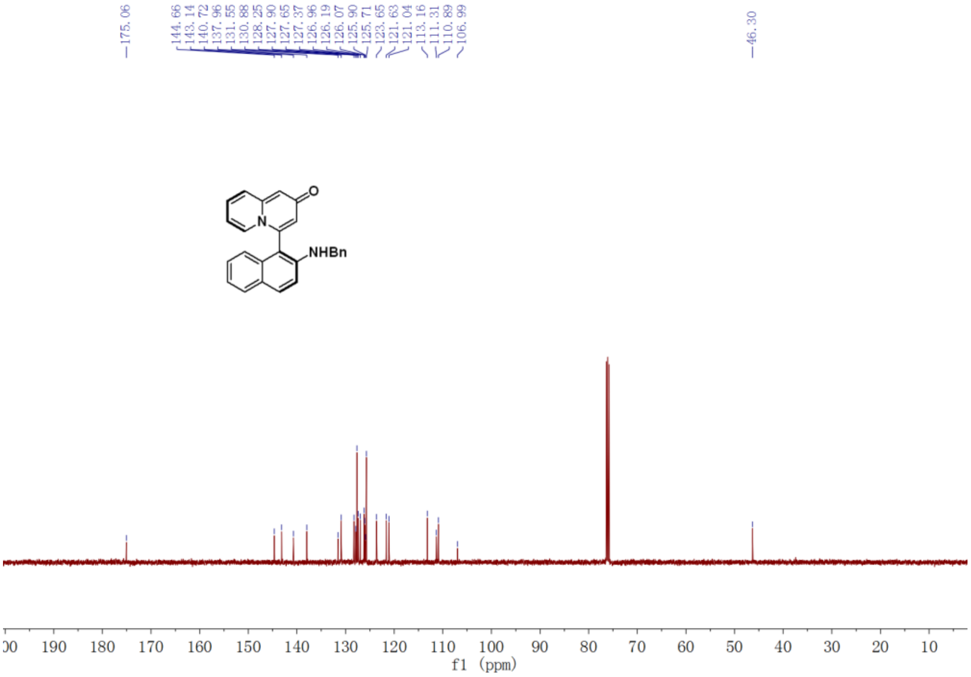

Supplementary Fig. 128. ^{13}C NMR spectrum of 7.

8, 4-(2-aminonaphthalen-1-yl)-6,7,8,9-tetrahydro-2*H*-quinolizin-2-one

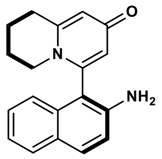

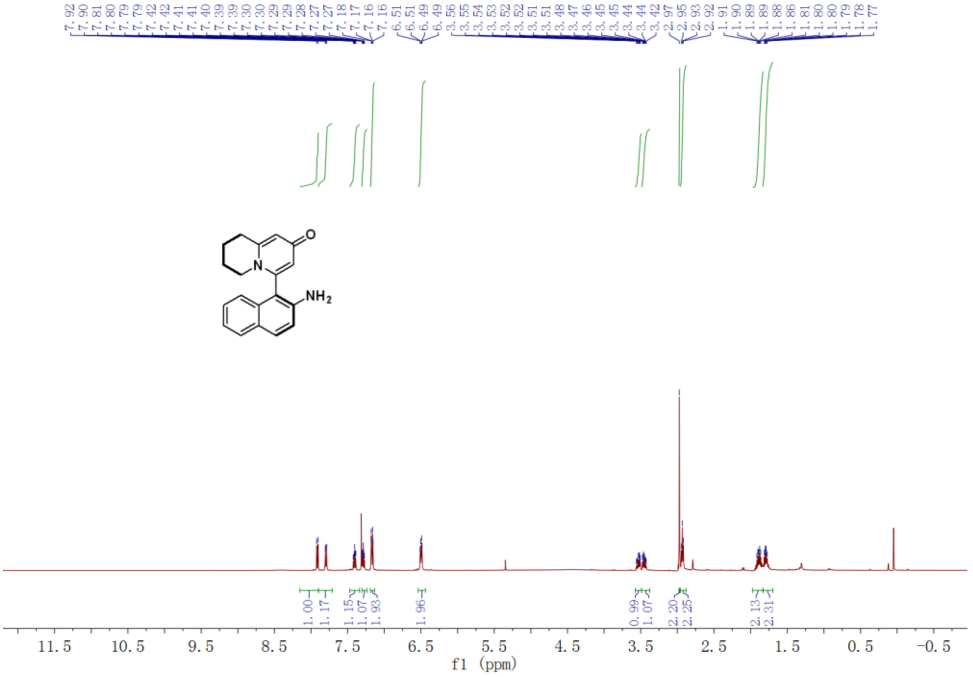

Supplementary Fig. 129. ^{1}H NMR spectrum of 8.

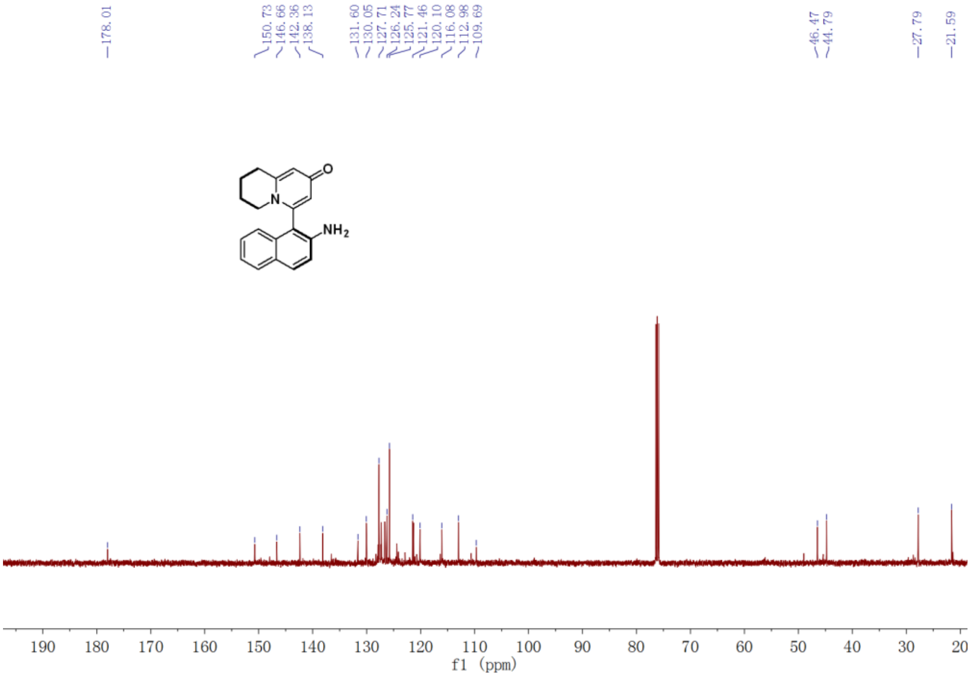

Supplementary Fig. 130. ^{13}C NMR spectrum of 8.

9, 4-(2-aminonaphthalen-1-yl)-2*H*-quinolizin-2-one

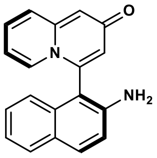

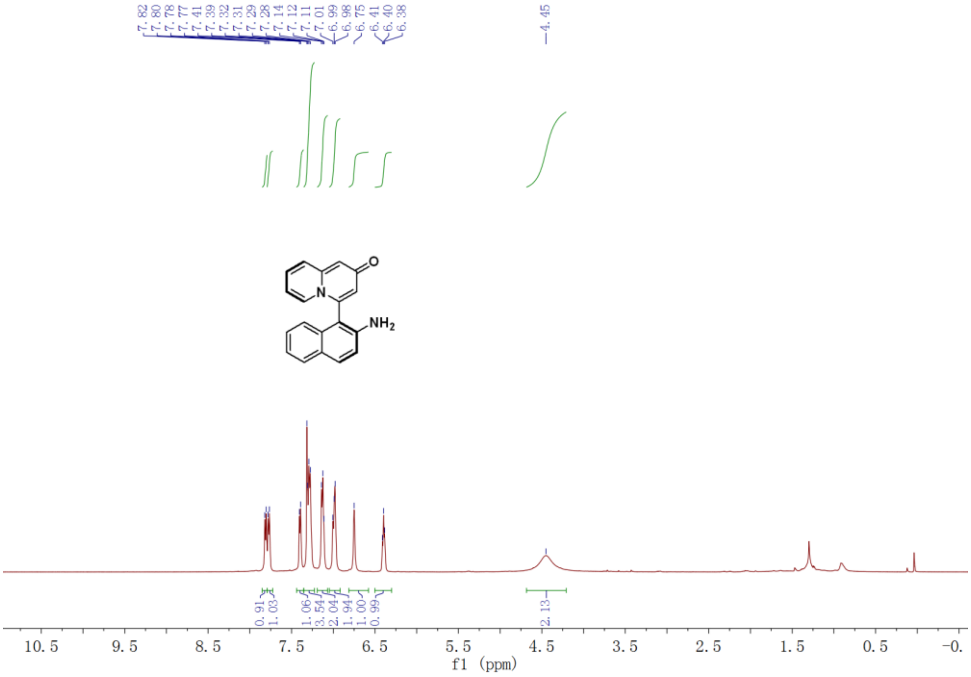

Supplementary Fig. 131. ^{1}H NMR spectrum of 9.

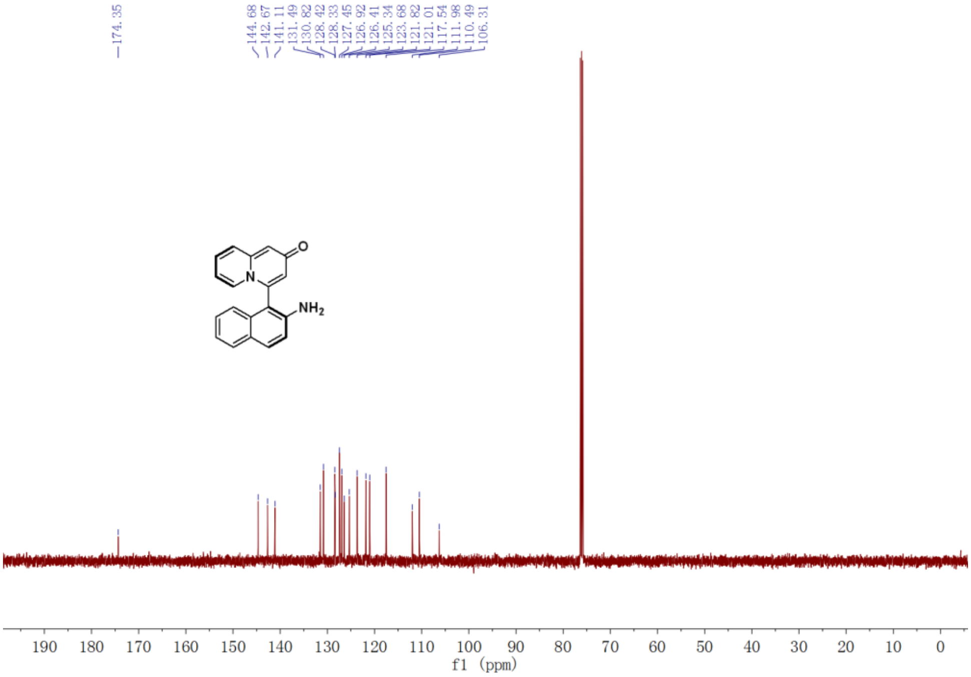

Supplementary Fig. 132. ^{13}C NMR spectrum of 9.

10, 1-(3,5-bis(trifluoromethyl)phenyl)-3-(1-(2-oxo-6,7,8,9-tetrahydro-2*H*-quinolizin-4-yl)naphthalen-2-yl)thiourea

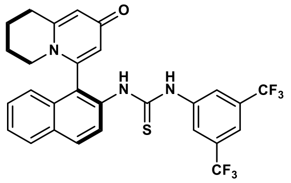

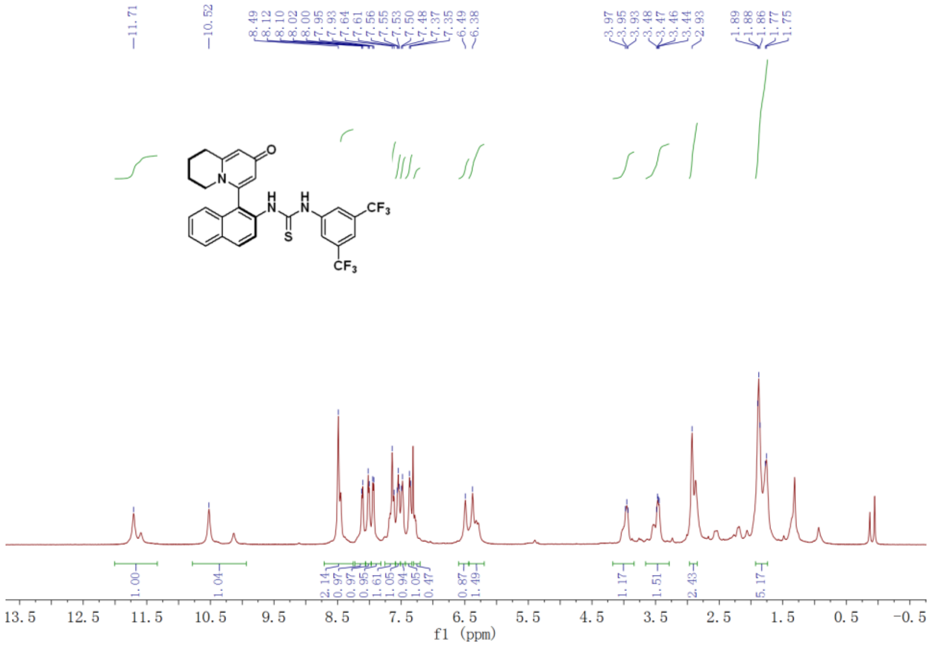

Supplementary Fig. 133. ^{1}H NMR spectrum of 10.

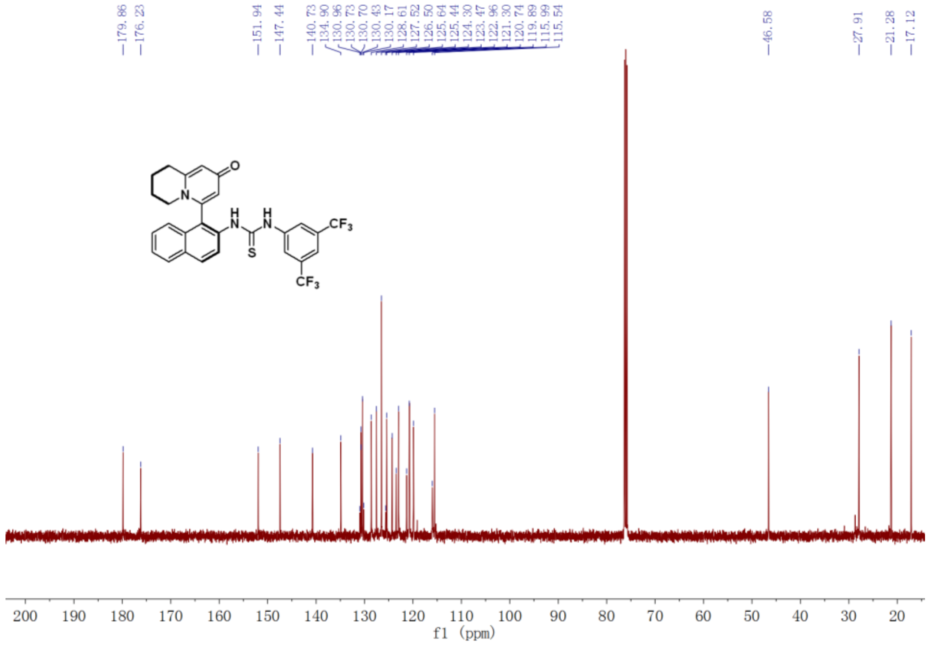

Supplementary Fig. 134. ^{13}C NMR spectrum of 10.

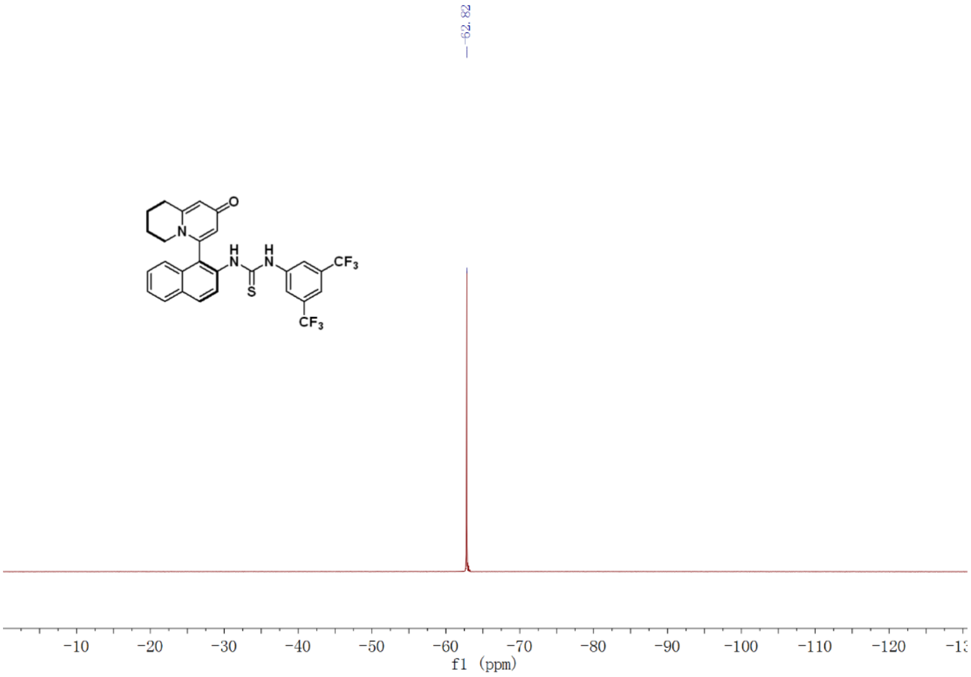

Supplementary Fig. 135. ^{19}F NMR spectrum of 10.

11, 1-(3,5-bis(trifluoromethyl)phenyl)-3-(1-(2-oxo-2**H**-quinolizin-4-yl) naphthalen-2-yl)thiourea

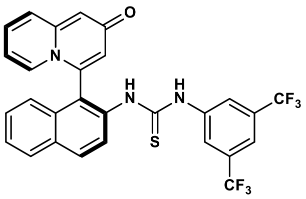

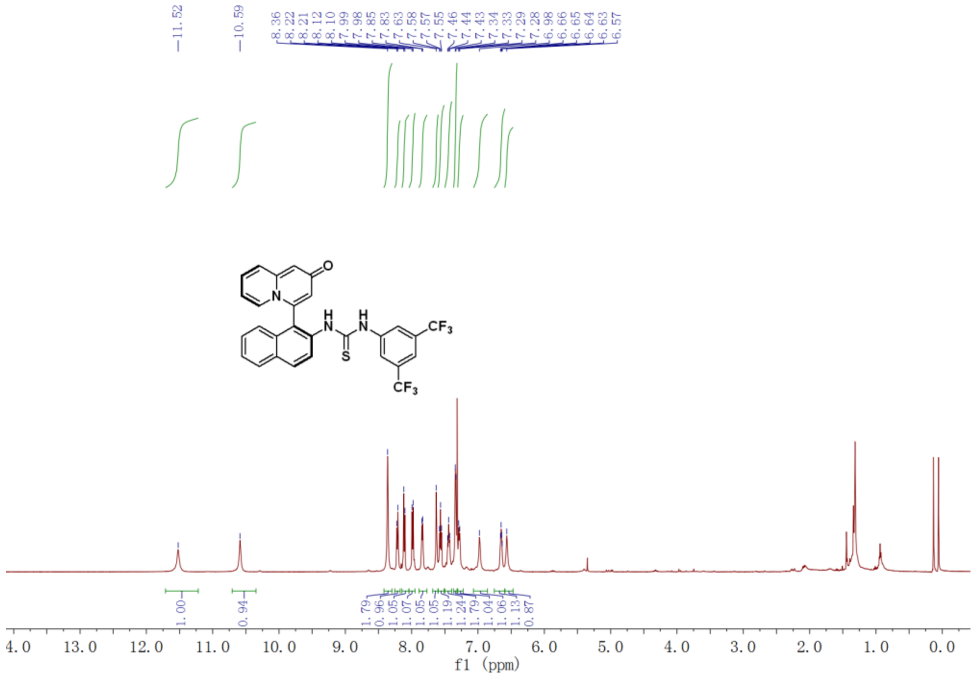

Supplementary Fig. 136. ^{1}H NMR spectrum of 11.

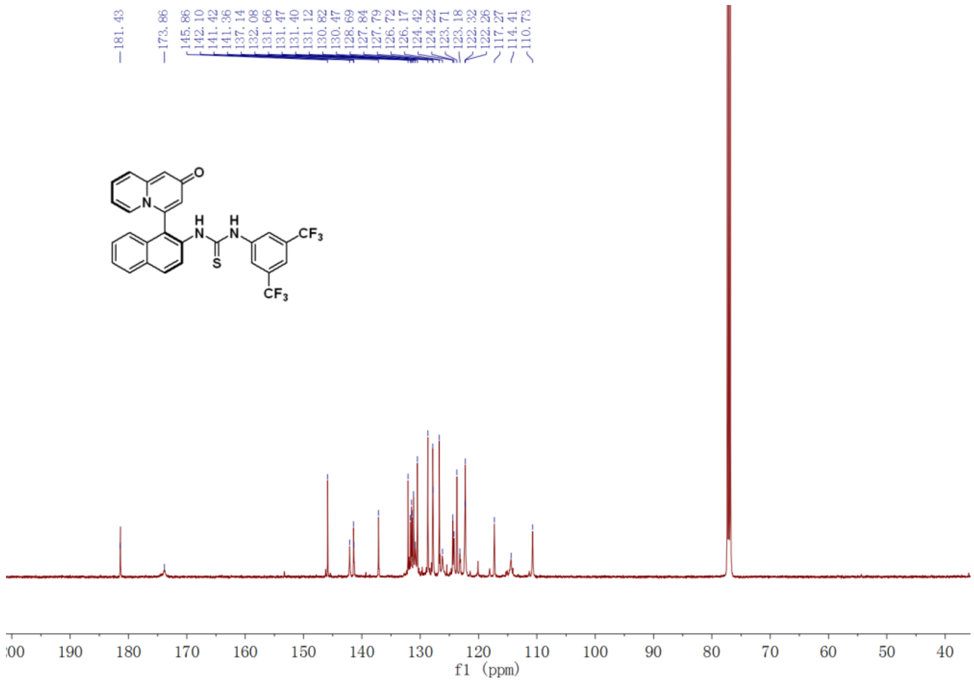

Supplementary Fig. 137. ^{13}C NMR spectrum of 11.

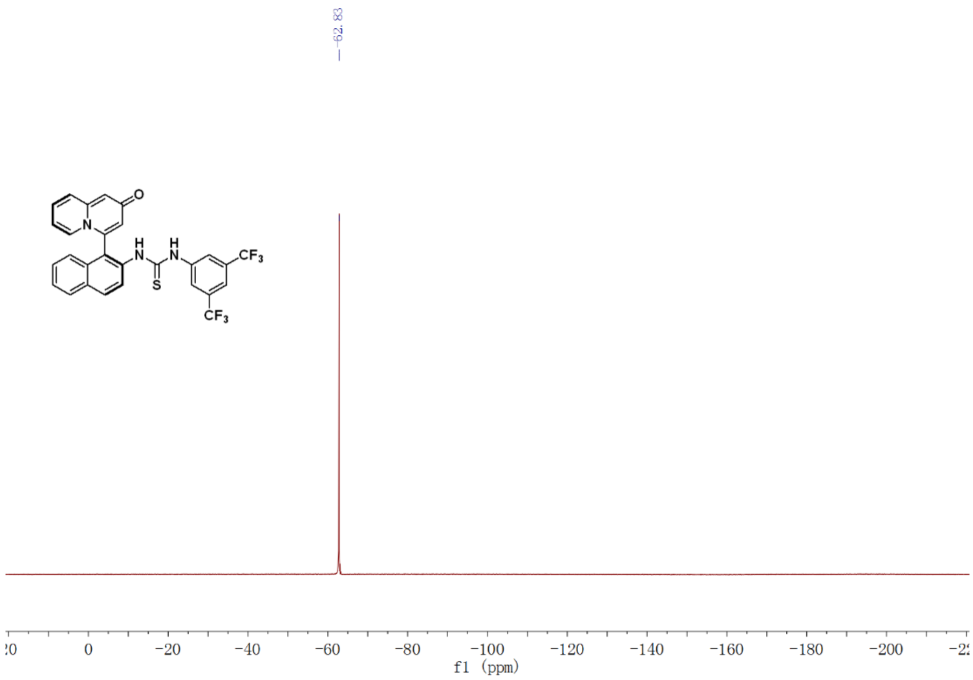

Supplementary Fig. 138. ^{19}F NMR spectrum of 11.

HPLC data

2a, 4-(2-methoxynaphthalen-1-yl)-2*H*-quinolizin-2-one

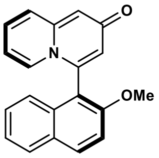

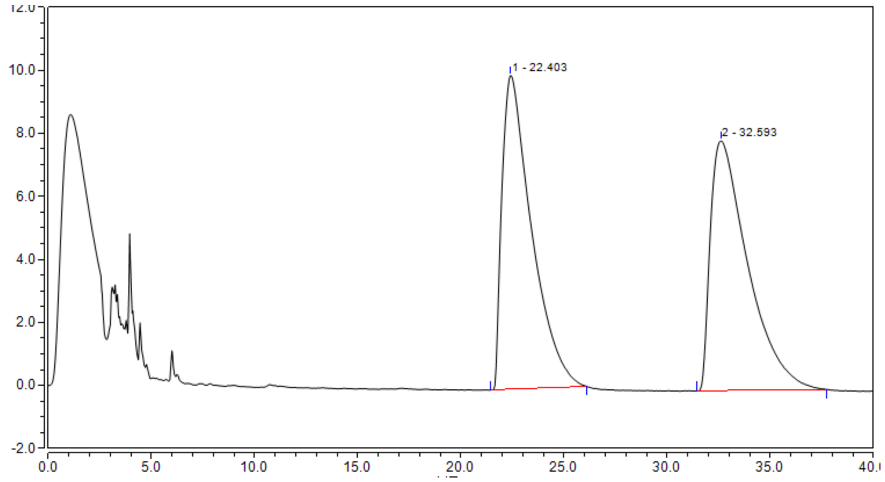

Ret. Time (min)	Area (mAU*min)	Height (mAU)	Area %	Height %
22.403	16.561	9.963	49.89	55.69
32.593	16.635	7.926	50.11	44.31
	**33.196**	**17.889**	**100.00**	**100.00**

Supplementary Fig. 139. HPLC spectrum of racemic 2a.

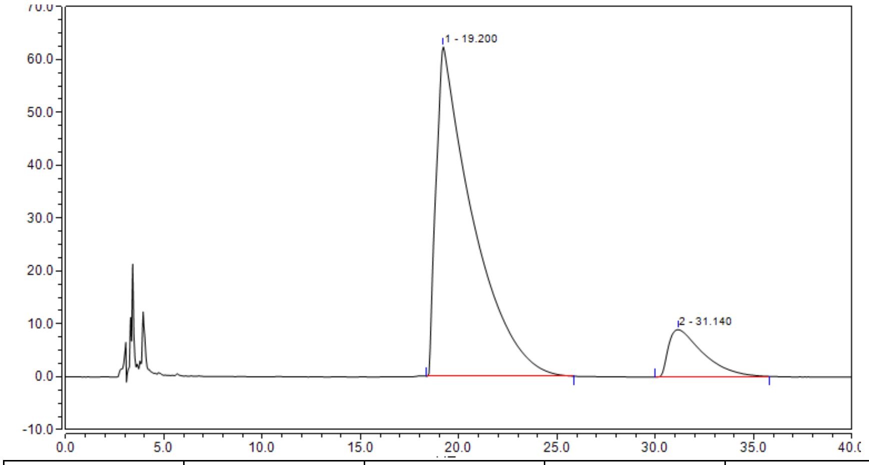

Ret. Time (min)	Area (mAU*min)	Height (mAU)	Area %	Height %
19.200	133.998	62.326	88.06	87.46
31.140	18.175	8.938	11.94	12.54
	**152.173**	**71.264**	**100.00**	**100.00**

Supplementary Fig. 140. HPLC spectrum of chiral 2a.

2b, 4-(2-ethoxynaphthalen-1-yl)-2*H*-quinolizin-2-one

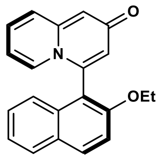

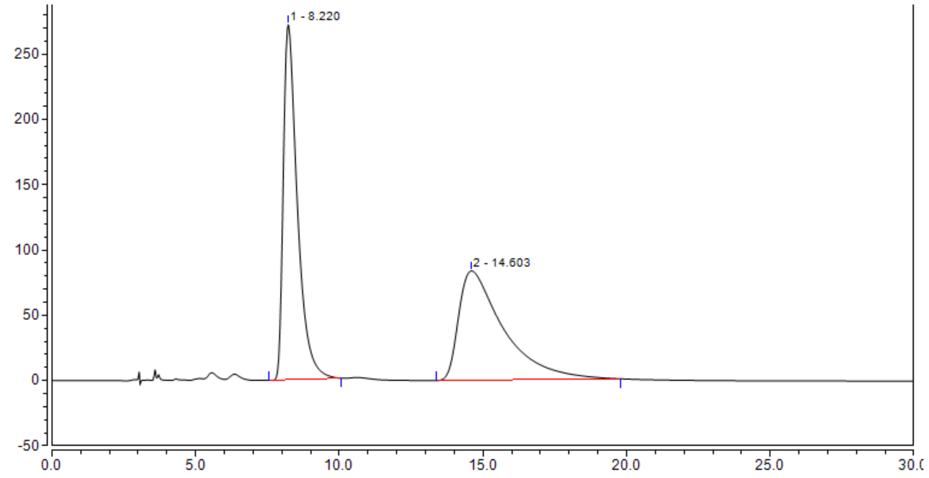

Ret. Time (min)	Area (mAU*min)	Height (mAU)	Area %	Height %
8.220	154.581	271.850	50.43	76.44
14.603	151.962	83.785	49.57	23.56
	**306.544**	**355.635**	**100.00**	**100.00**

Supplementary Fig. 141. HPLC spectrum of racemic 2b.

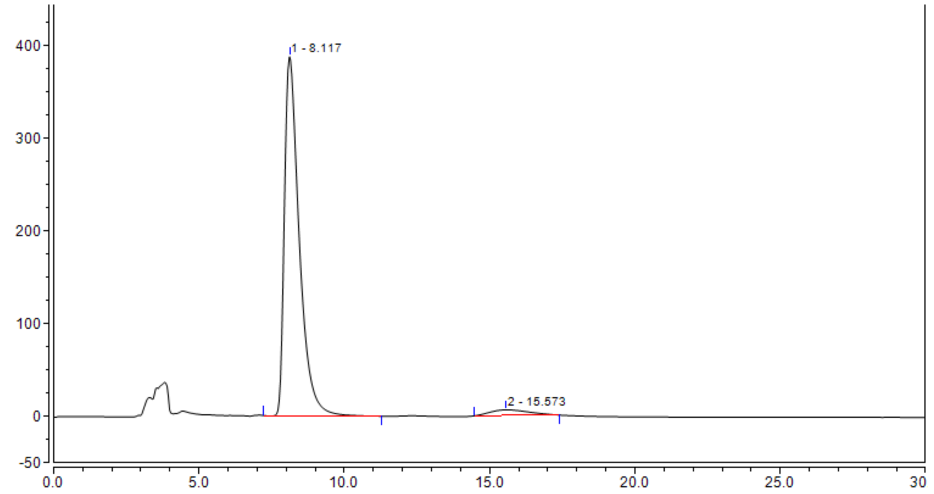

Ret. Time (min)	Area (mAU*min)	Height (mAU)	Area %	Height %
8.117	228.832	387.316	96.00	98.44
15.573	9.541	6.122	4.00	1.56
	**238.373**	**393.439**	**100.00**	**100.00**

Supplementary Fig. 142. HPLC spectrum of chiral 2b.

2c, 4-(6-bromo-2-ethoxynaphthalen-1-yl)-2*H*-quinolizin-2-one

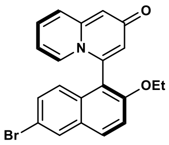

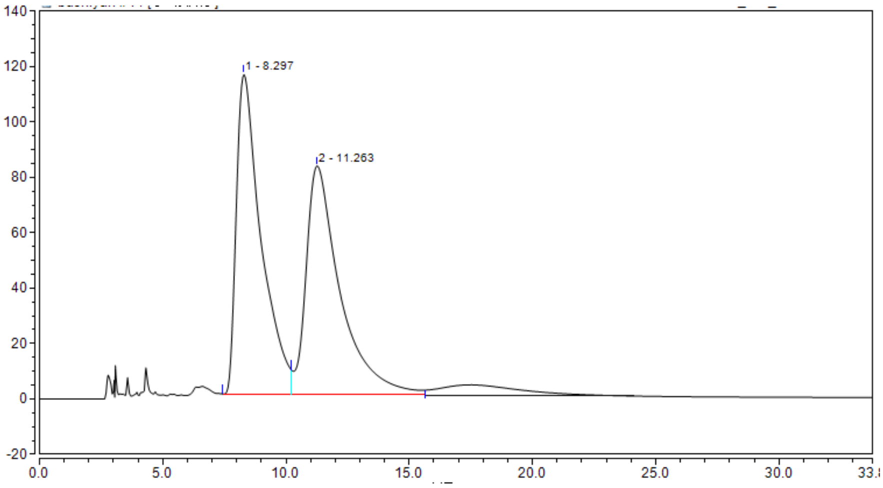

Ret. Time (min)	Area (mAU*min)	Height (mAU)	Area %	Height %
8.297	133.578	115.404	50.29	58.30
11.263	132.035	82.533	49.71	41.70
	**265.614**	**197.937**	**100.00**	**100.00**

Supplementary Fig. 143. HPLC spectrum of racemic 2c.

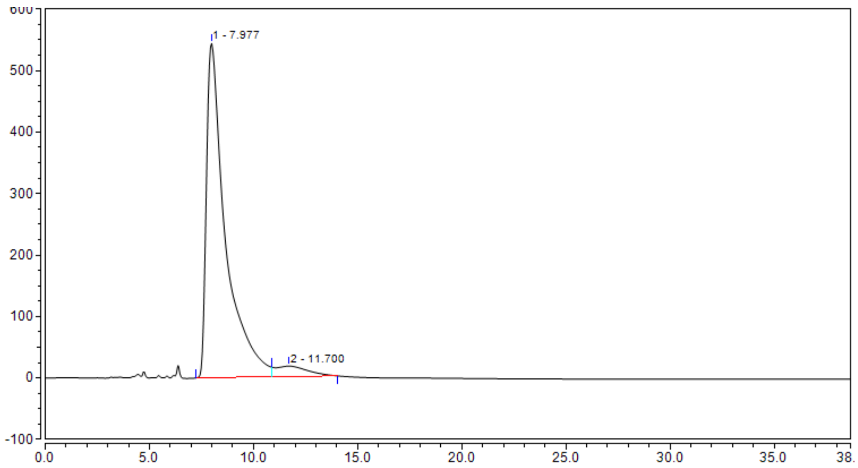

Ret. Time (min)	Area (mAU*min)	Height (mAU)	Area %	Height %
7.977	574.678	544.487	94.87	96.94
11.700	31.051	17.165	5.13	3.06
	**605.729**	**561.652**	**100.00**	**100.00**

Supplementary Fig. 144. HPLC spectrum of chiral 2c.

2d, 4-(7-bromo-2-ethoxynaphthalen-1-yl)-2*H*-quinolizin-2-one

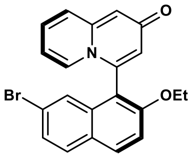

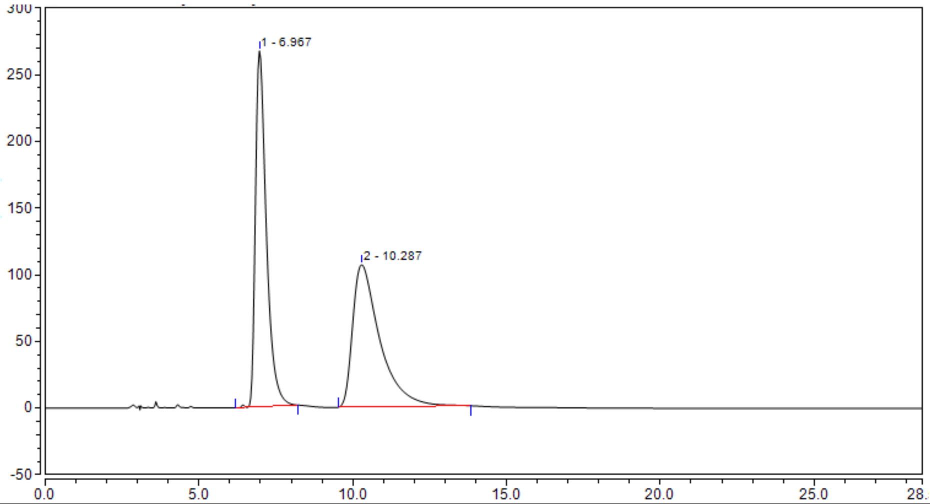

Ret. Time (min)	Area (mAU*min)	Height (mAU)	Area %	Height %
6.967	109.682	266.955	50.11	71.45
10.287	109.185	106.685	49.89	28.55
	**218.866**	**373.640**	**100.00**	**100.00**

Supplementary Fig. 145. HPLC spectrum of racemic 2d.

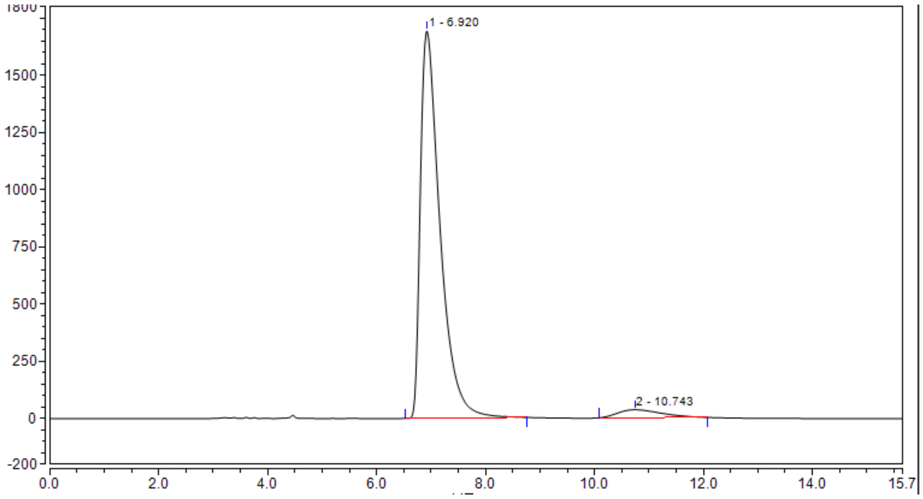

Ret. Time (min)	Area (mAU*min)	Height (mAU)	Area %	Height %
6.920	705.916	1691.753	95.61	97.98
10.743	32.420	34.793	4.39	2.02
	**738.336**	**1726.546**	**100.00**	**100.00**

Supplementary Fig. 146. HPLC spectrum of chiral 2d.

2e, 4-(2-ethoxy-7-phenylnaphthalen-1-yl)-2*H*-quinolizin-2-one

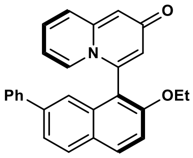

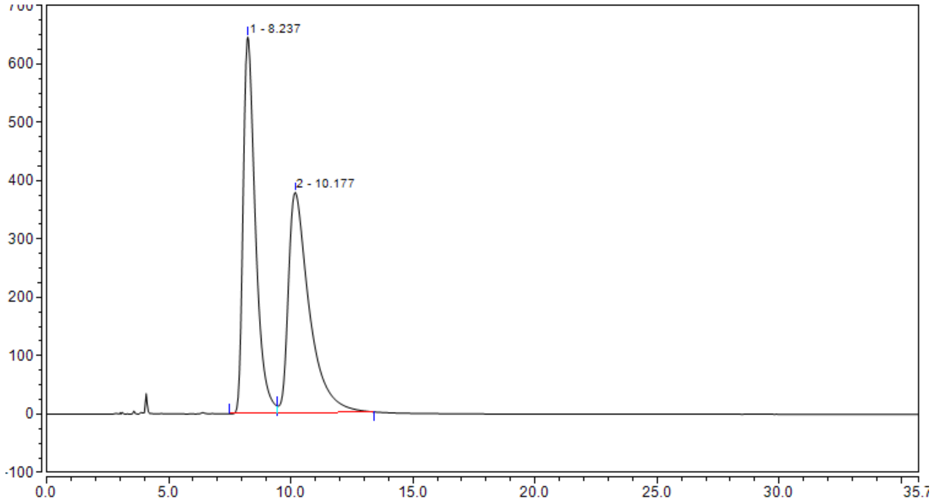

Ret. Time (min)	Area (mAU*min)	Height (mAU)	Area %	Height %
8.237	376.680	645.359	50.00	63.04
10.177	376.615	378.429	50.00	36.96
	**753.295**	**1023.788**	**100.00**	**100.00**

Supplementary Fig. 147. HPLC spectrum of racemic 2e.

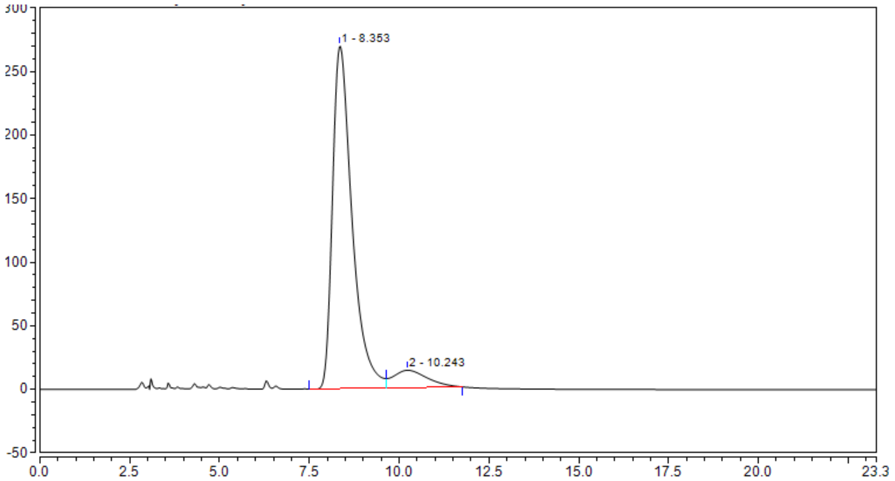

Ret. Time (min)	Area (mAU*min)	Height (mAU)	Area %	Height %
8.353	170.112	269.770	91.79	95.15
10.243	15.218	13.749	8.21	4.85
	**185.330**	**283.519**	**100.00**	**100.00**

Supplementary Fig. 148. HPLC spectrum of chiral 2e.

2f, 4-(2-ethoxy-7-(phenylethynyl)naphthalen-1-yl)-2*H*-quinolizin-2-one

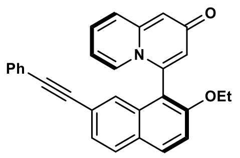

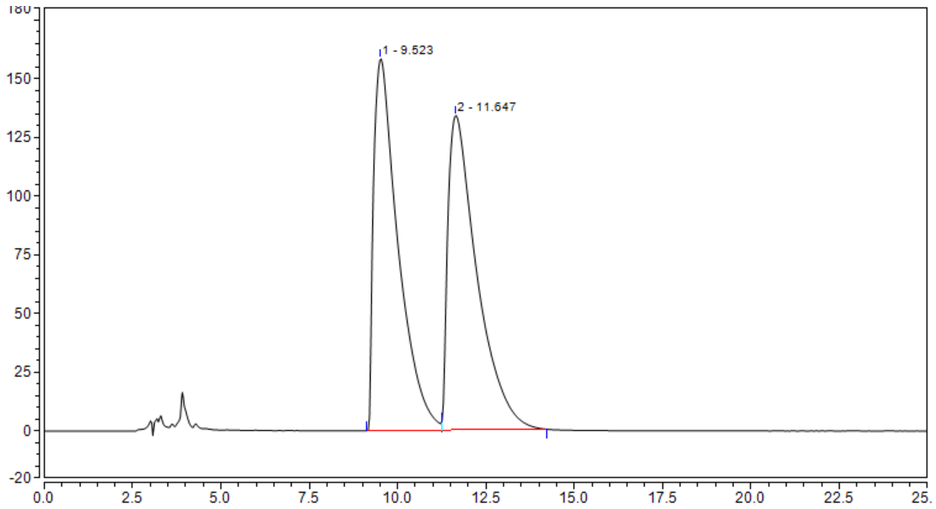

Ret. Time (min)	Area (mAU*min)	Height (mAU)	Area %	Height %
9.523	127.179	158.469	49.88	54.18
11.647	127.804	134.003	50.12	45.82
	**254.983**	**292.472**	**100.00**	**100.00**

Supplementary Fig. 149. HPLC spectrum of racemic 2f.

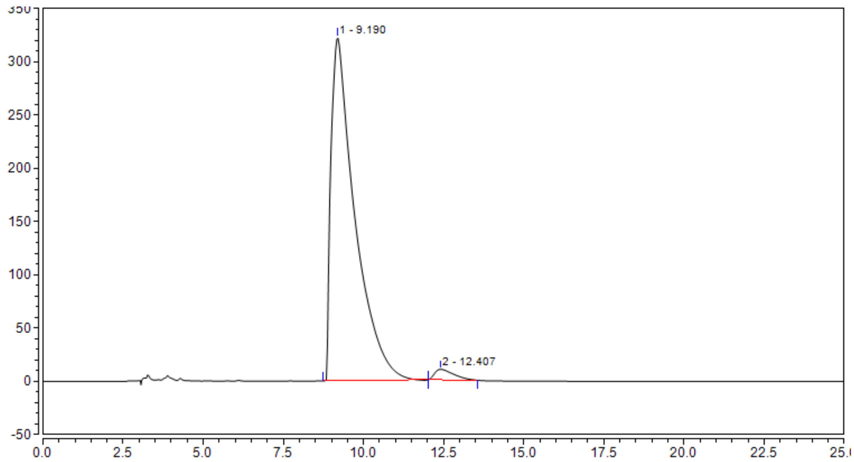

Ret. Time (min)	Area (mAU*min)	Height (mAU)	Area %	Height %
9.190	276.815	322.149	97.58	97.00
12.407	6.855	9.952	2.42	3.00
	**283.671**	**332.101**	**100.00**	**100.00**

Supplementary Fig. 150. HPLC spectrum of chiral 2f.

2g, 4-(2-((4-methoxybenzyl)oxy)naphthalen-1-yl)-2*H*-quinolizin-2-one

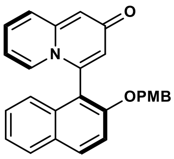

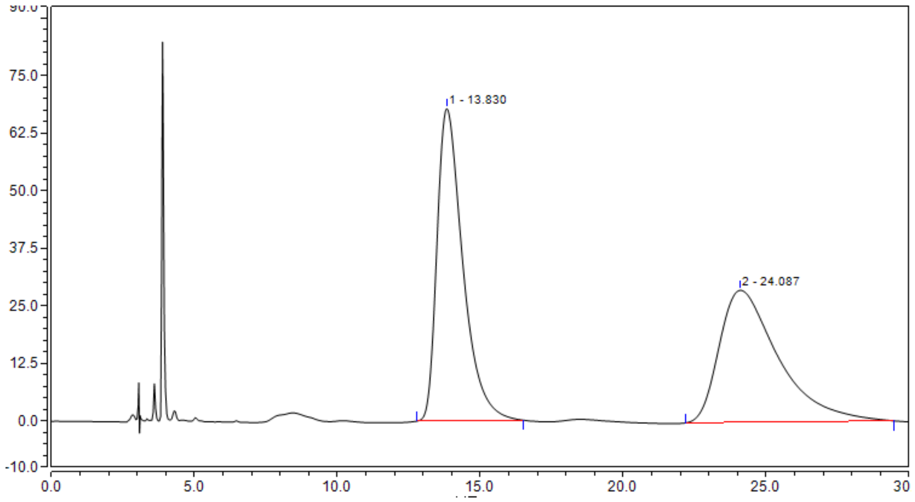

Ret. Time (min)	Area (mAU*min)	Height (mAU)	Area %	Height %
13.830	71.014	67.803	50.79	70.27
24.087	68.808	28.686	49.21	29.73
	**139.822**	**96.489**	**100.00**	**100.00**

Supplementary Fig. 151. HPLC spectrum of racemic 2g.

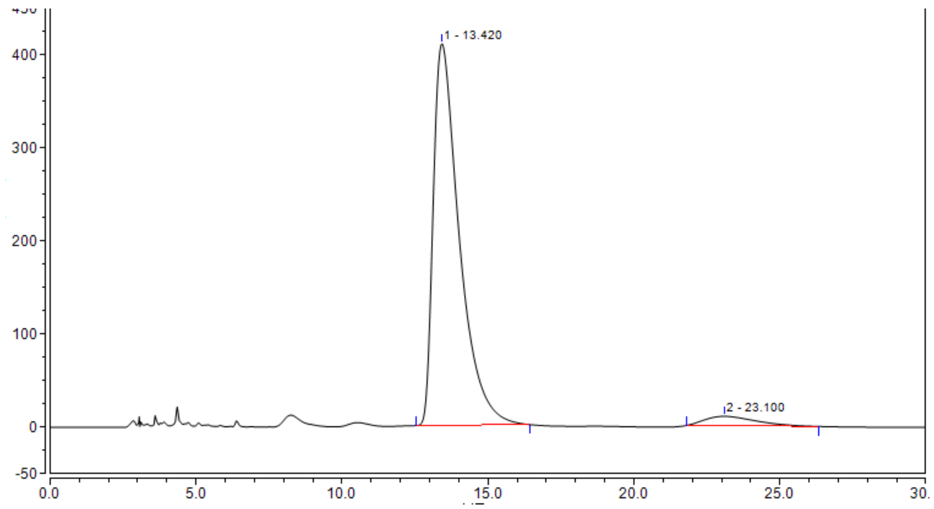

Ret. Time (min)	Area (mAU*min)	Height (mAU)	Area %	Height %
13.420	423.151	410.141	95.35	97.59
23.100	20.645	10.117	4.65	2.41
	**443.796**	**420.258**	**100.00**	**100.00**

Supplementary Fig. 152. HPLC spectrum of chiral 2g.

2h, 4-(2-(dimethylamino)naphthalen-1-yl)-2*H*-quinolizin-2-one

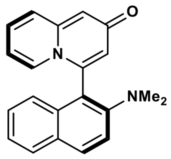

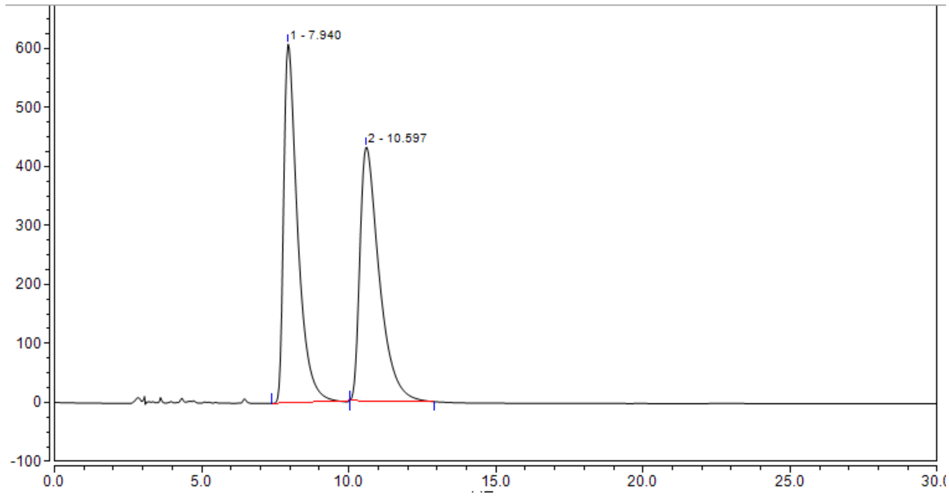

Ret. Time (min)	Area (mAU*min)	Height (mAU)	Area %	Height %
7.940	333.945	608.182	50.51	58.54
10.597	327.255	430.787	49.49	41.46
	661.200	1038.969	100.00	100.00

Supplementary Fig. 153. HPLC spectrum of racemic 2h.

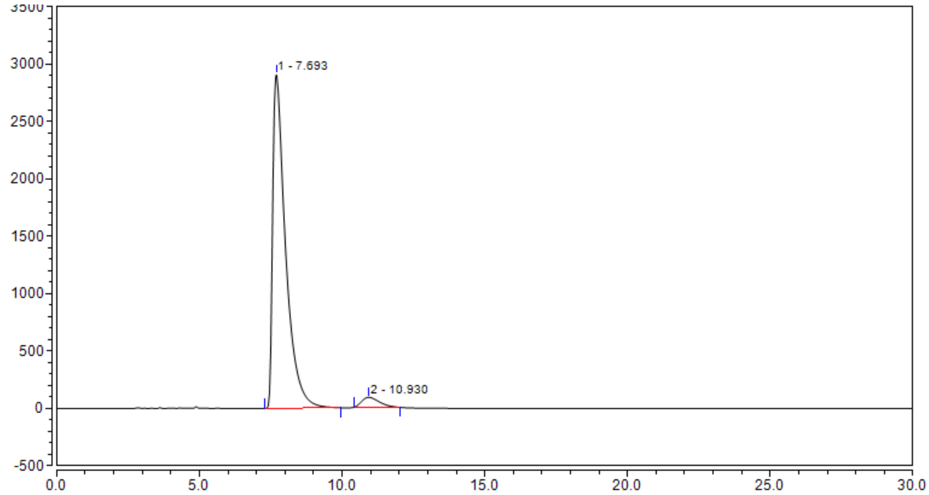

Ret. Time (min)	Area (mAU*min)	Height (mAU)	Area %	Height %
7.693	1523.968	2907.582	96.20	97.16
10.930	60.133	85.017	3.80	2.84
	1584.101	2992.599	100.00	100.00

Supplementary Fig. 154. HPLC spectrum of chiral 2h.

2i, 4-(2-(dibenzylamino)naphthalen-1-yl)-2*H*-quinolizin-2-one

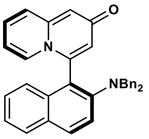

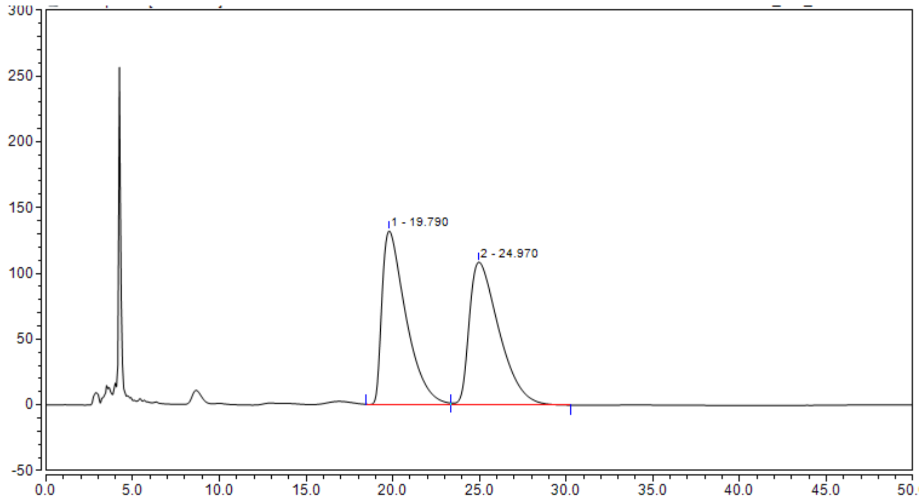

Ret. Time (min)	Area (mAU*min)	Height (mAU)	Area %	Height %
19.790	211.241	131.896	49.52	54.87
24.970	215.297	108.462	50.48	45.13
	**426.538**	**240.358**	**100.00**	**100.00**

Supplementary Fig. 155. HPLC spectrum of racemic 2i.

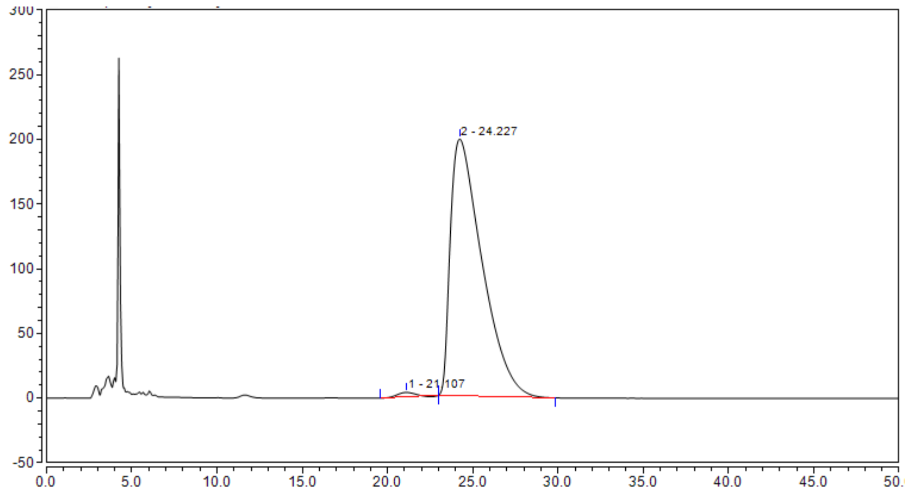

Ret. Time (min)	Area (mAU*min)	Height (mAU)	Area %	Height %
21.107	3.522	3.429	0.82	1.70
24.227	427.313	198.465	99.18	98.30
	**430.835**	**201.894**	**100.00**	**100.00**

Supplementary Fig. 156. HPLC spectrum of chiral 2i.

2j, 4-(2-methylnaphthalen-1-yl)-2*H*-quinolizin-2-one

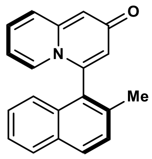

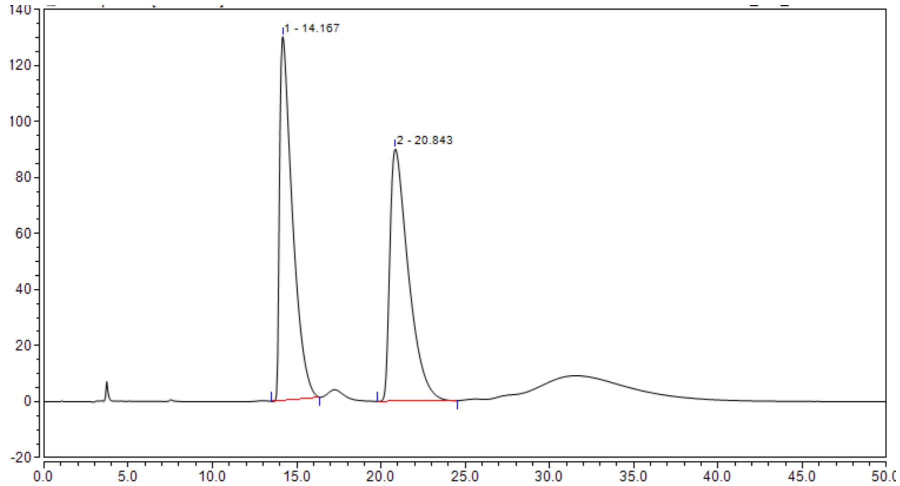

Ret. Time (min)	Area (mAU*min)	Height (mAU)	Area %	Height %
14.167	115.138	129.962	50.10	59.04
20.843	114.667	90.147	49.90	40.96
	**229.805**	**220.109**	**100.00**	**100.00**

Supplementary Fig. 157. HPLC spectrum of racemic 2j.

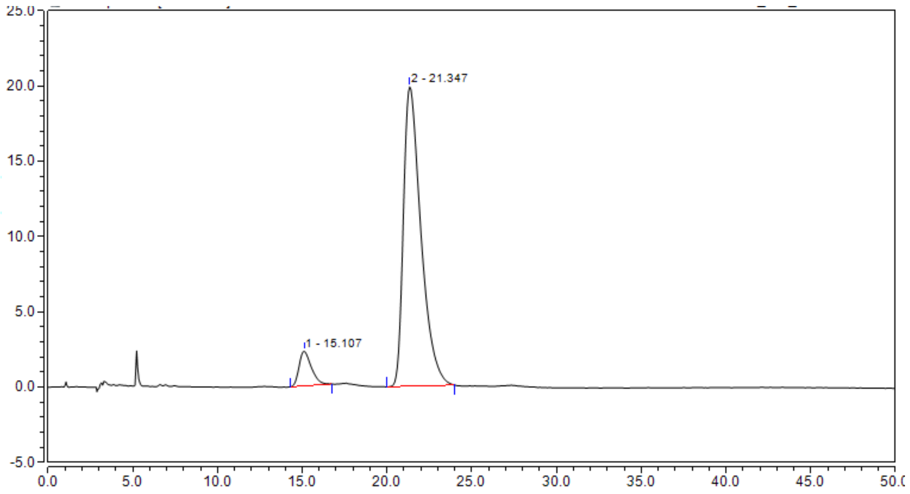

Ret. Time (min)	Area (mAU*min)	Height (mAU)	Area %	Height %
15.107	1.953	2.307	7.58	10.39
21.347	23.828	19.893	92.42	89.61
	**25.782**	**22.200**	**100.00**	**100.00**

Supplementary Fig. 158. HPLC spectrum of chiral 2j.

2k, 4-(2-ethylnaphthalen-1-yl)-2*H*-quinolizin-2-one

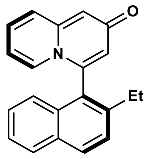

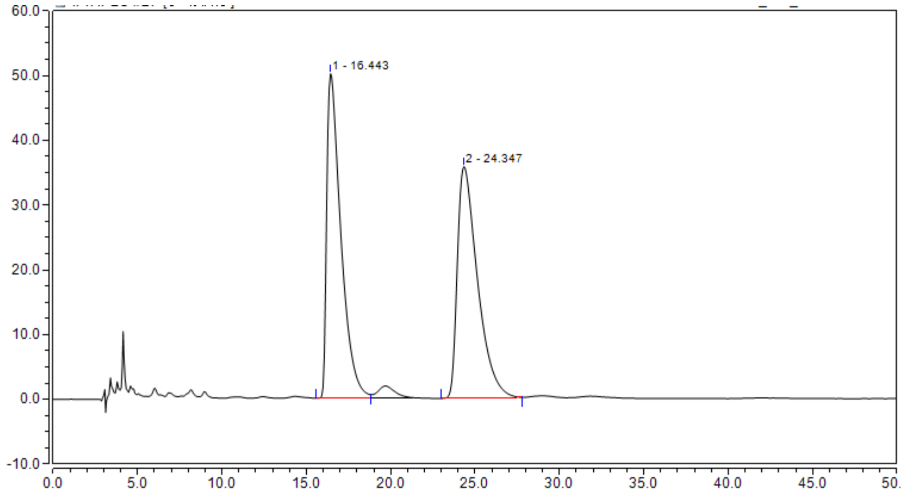

Ret. Time (min)	Area (mAU*min)	Height (mAU)	Area %	Height %
16.443	48.720	50.120	49.70	58.35
24.347	49.304	35.774	50.30	41.65
	**98.024**	**85.894**	**100.00**	**100.00**

Supplementary Fig. 159. HPLC spectrum of racemic 2k.

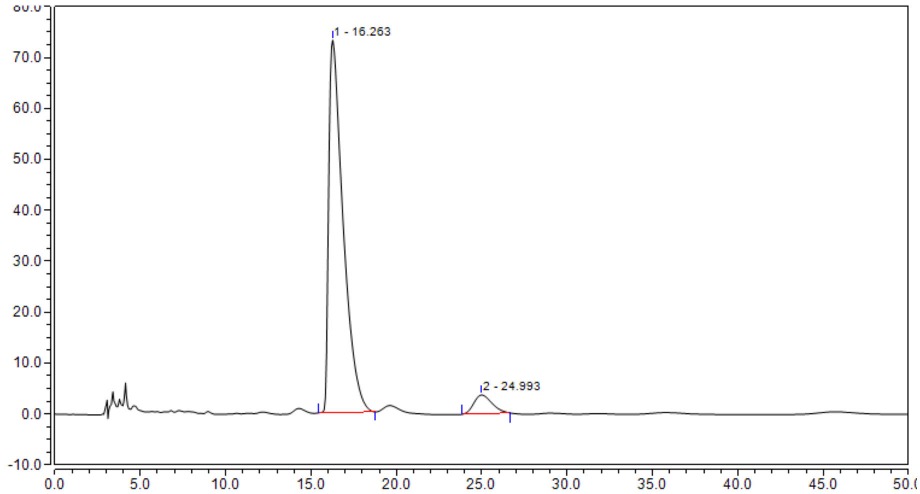

Ret. Time (min)	Area (mAU*min)	Height (mAU)	Area %	Height %
16.263	71.652	73.144	94.02	95.18
24.993	4.558	3.701	5.98	4.82
	**76.210**	**76.845**	**100.00**	**100.00**

Supplementary Fig. 160. HPLC spectrum of chiral 2k.

2l, 4-(2-butylnaphthalen-1-yl)-2*H*-quinolizin-2-one

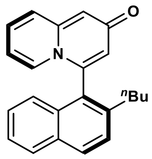

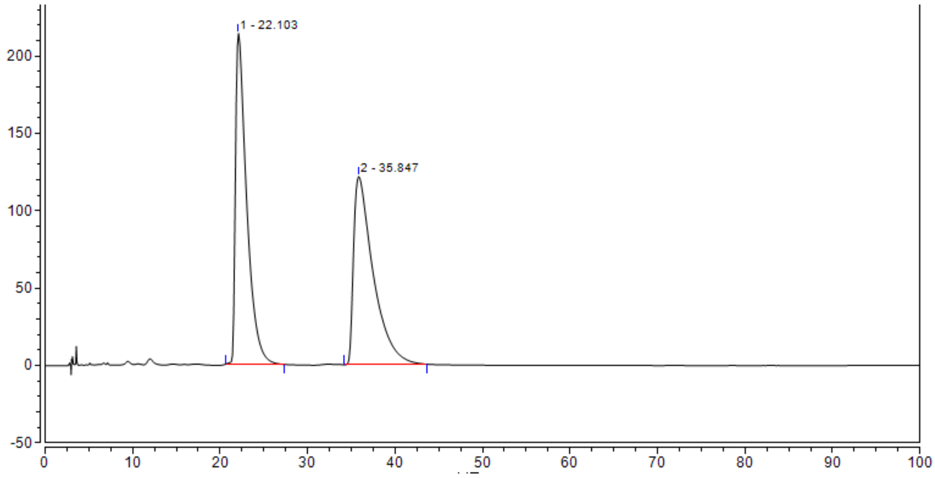

Ret. Time (min)	Area (mAU*min)	Height (mAU)	Area %	Height %
22.103	316.775	213.669	50.13	63.71
35.847	315.075	121.695	49.87	36.29
	**631.849**	**335.364**	**100.00**	**100.00**

Supplementary Fig. 161. HPLC spectrum of racemic 2l.

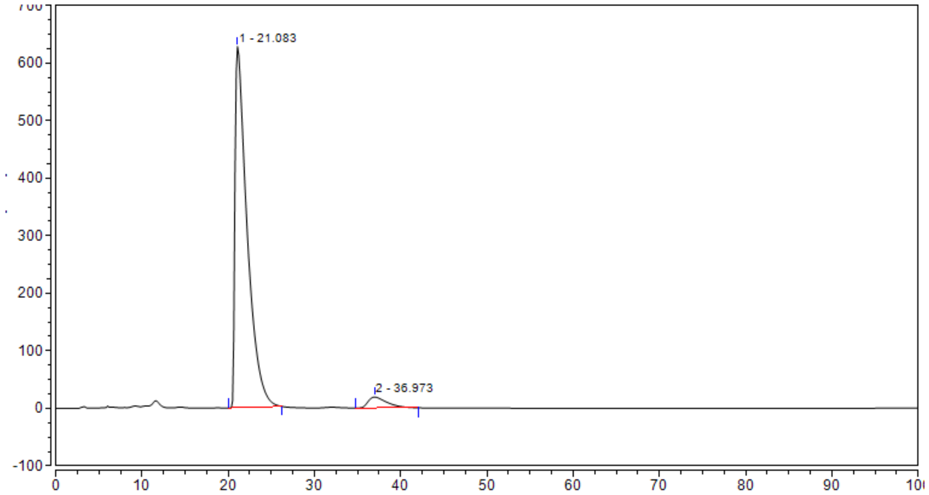

Ret. Time (min)	Area (mAU*min)	Height (mAU)	Area %	Height %
21.083	1024.229	628.384	95.45	97.06
36.973	48.809	19.049	4.55	2.94
	**1073.038**	**647.433**	**100.00**	**100.00**

Supplementary Fig. 162. HPLC spectrum of chiral 2l.

2m, 4-(2-isopropylnaphthalen-1-yl)-2*H*-quinolizin-2-one

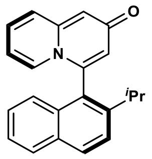

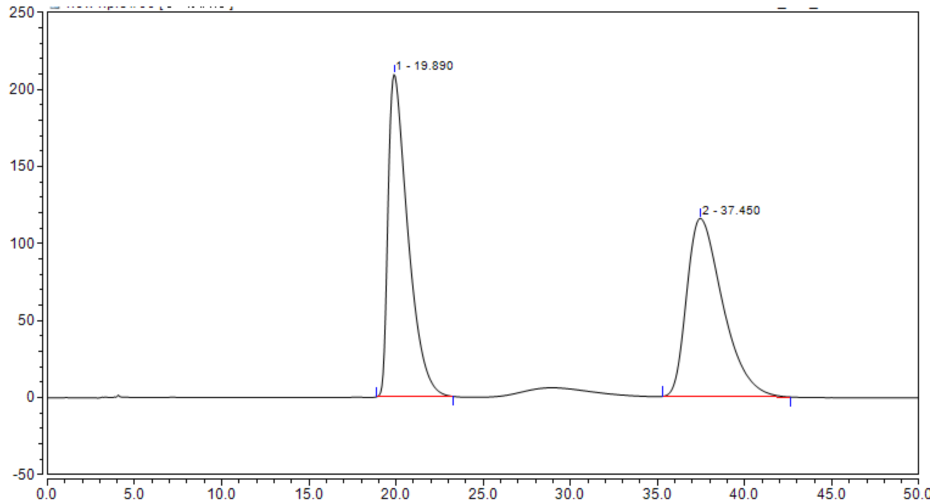

Ret. Time (min)	Area (mAU*min)	Height (mAU)	Area %	Height %
19.890	275.422	209.290	49.80	64.40
37.450	277.619	115.686	50.20	35.60
	**553.041**	**324.977**	**100.00**	**100.00**

Supplementary Fig. 163. HPLC spectrum of racemic 2m.

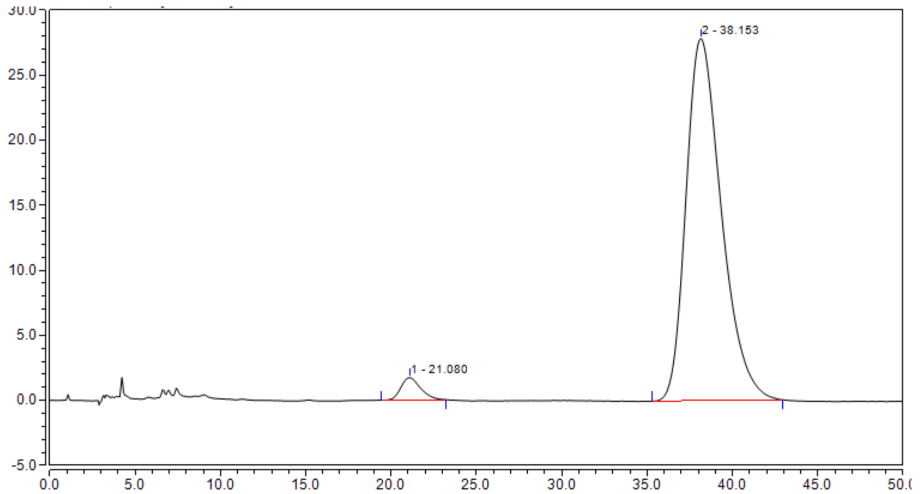

Ret. Time (min)	Area (mAU*min)	Height (mAU)	Area %	Height %
21.080	2.308	1.727	3.34	5.84
38.153	66.764	27.833	96.66	94.16
	**69.072**	**29.559**	**100.00**	**100.00**

Supplementary Fig. 164. HPLC spectrum of chiral 2m.

2n, 4-(2-phenylnaphthalen-1-yl)-2*H*-quinolizin-2-one

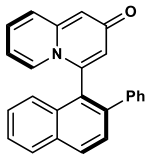

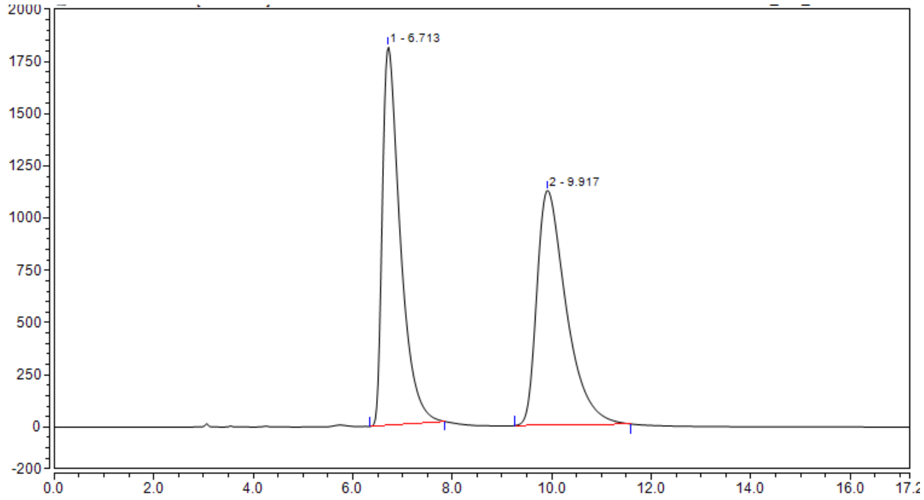

Ret. Time (min)	Area (mAU*min)	Height (mAU)	Area %	Height %
6.713	756.574	1811.137	49.64	61.71
9.917	767.576	1123.857	50.36	38.29
	**1524.150**	**2934.994**	**100.00**	**100.00**

Supplementary Fig. 165. HPLC spectrum of racemic 2n.

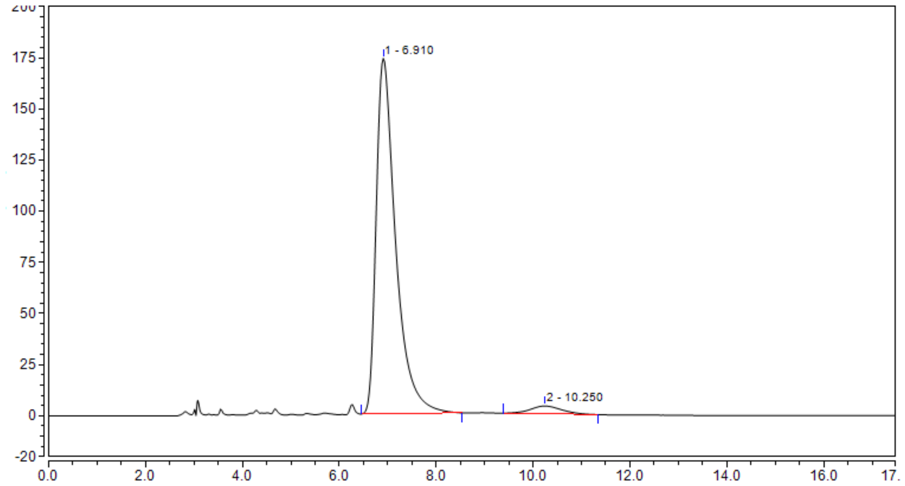

Ret. Time (min)	Area (mAU*min)	Height (mAU)	Area %	Height %
6.910	80.853	173.770	96.52	97.85
10.250	2.911	3.826	3.48	2.15
	**83.764**	**177.596**	**100.00**	**100.00**

Supplementary Fig. 166. HPLC spectrum of chiral 2n.

2o, 4-([1,2'-binaphthalen]-1'-yl)-2*H*-quinolizin-2-one

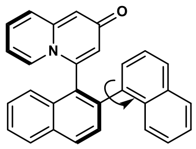

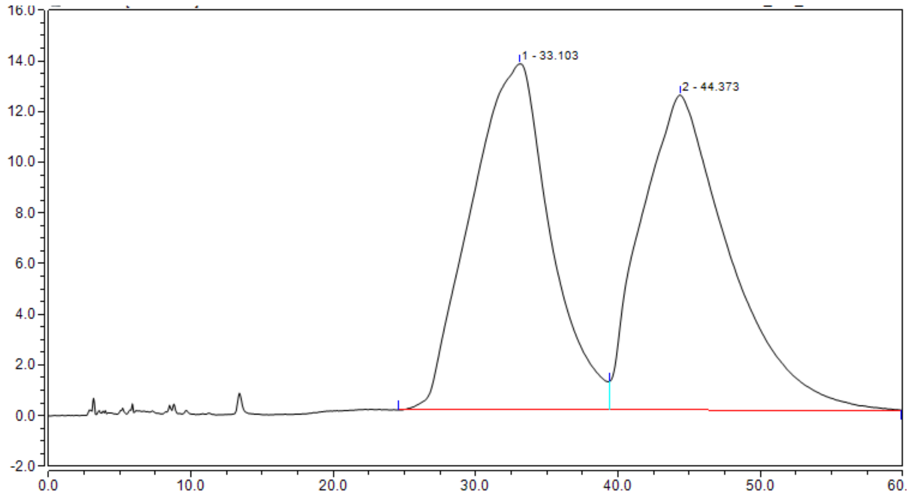

Ret. Time (min)	Area (mAU*min)	Height (mAU)	Area %	Height %
33.103	88.146	13.658	49.75	52.35
44.373	89.022	12.429	50.25	47.65
	**177.167**	**26.088**	**100.00**	**100.00**

Supplementary Fig. 167. HPLC spectrum of racemic 2o.

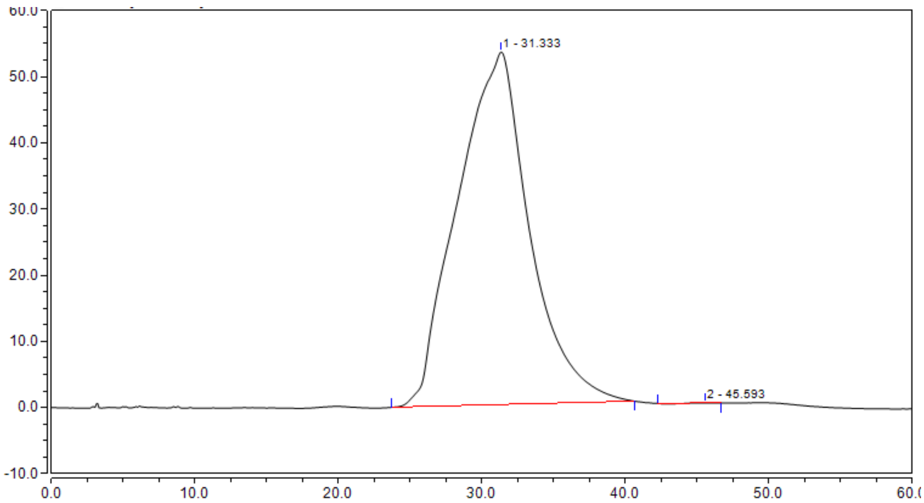

Ret. Time (min)	Area (mAU*min)	Height (mAU)	Area %	Height %
31.333	310.095	53.307	99.97	99.93
45.593	0.087	0.036	0.03	0.07
	**310.182**	**53.344**	**100.00**	**100.00**

Supplementary Fig. 168. HPLC spectrum of chiral 2o.

2p, 4-(2-bromonaphthalen-1-yl)-2*H*-quinolizin-2-one

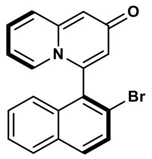

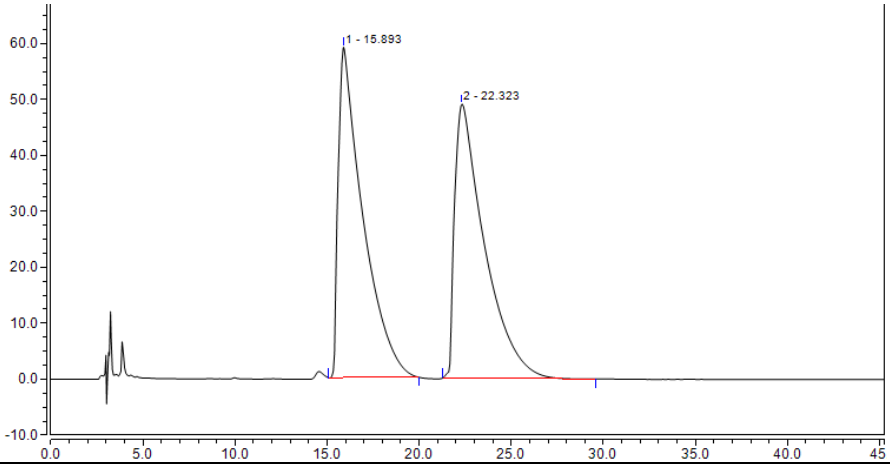

Ret. Time (min)	Area (mAU*min)	Height (mAU)	Area %	Height %
15.893	91.130	59.077	49.87	54.67
22.323	91.587	48.975	50.13	45.33
	**182.717**	**108.052**	**100.00**	**100.00**

Supplementary Fig. 169. HPLC spectrum of racemic 2p.

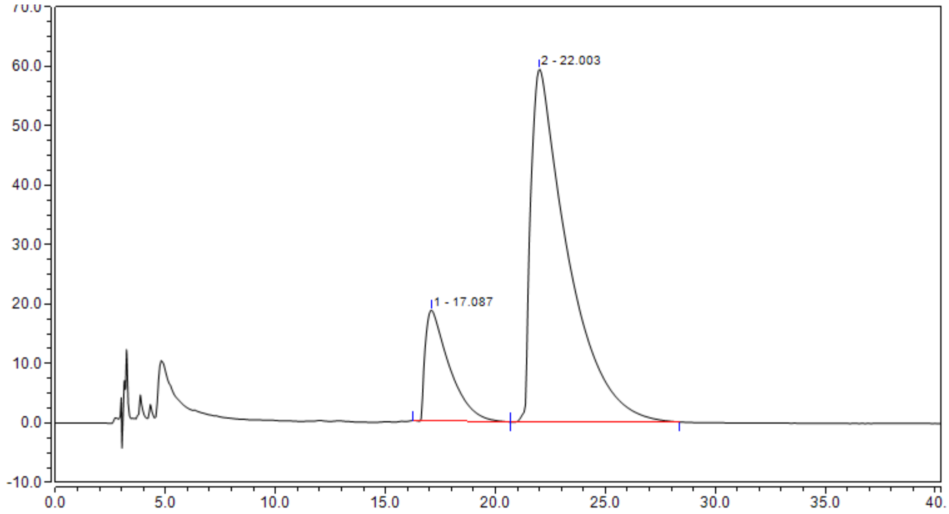

Ret. Time (min)	Area (mAU*min)	Height (mAU)	Area %	Height %
17.087	23.639	18.618	16.95	23.87
22.003	115.808	59.382	83.05	76.13
	**139.447**	**78.000**	**100.00**	**100.00**

Supplementary Fig. 170. HPLC spectrum of chiral 2p.

2q, 4-(2-chloronaphthalen-1-yl)-2*H*-quinolizin-2-one

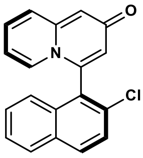

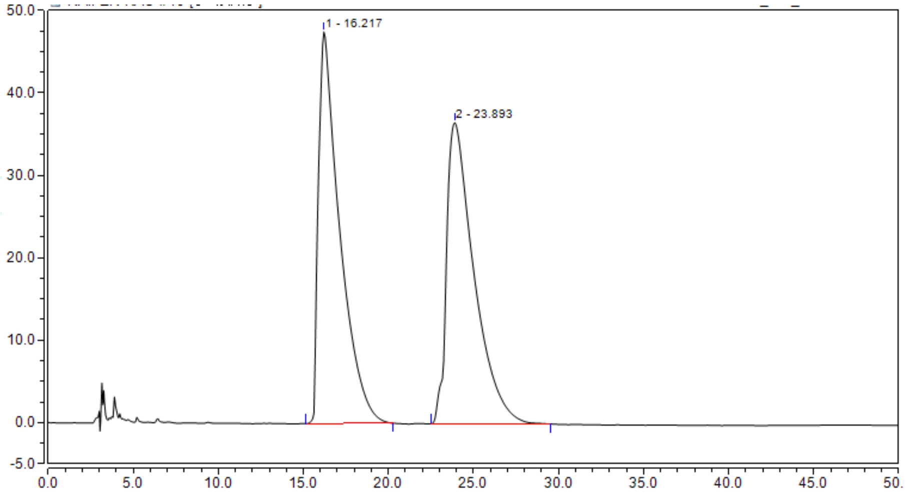

Ret. Time (min)	Area (mAU*min)	Height (mAU)	Area %	Height %
16.217	68.875	47.539	50.28	56.54
23.893	68.120	36.535	49.72	43.46
	**136.995**	**84.074**	**100.00**	**100.00**

Supplementary Fig. 171. HPLC spectrum of racemic 2q.

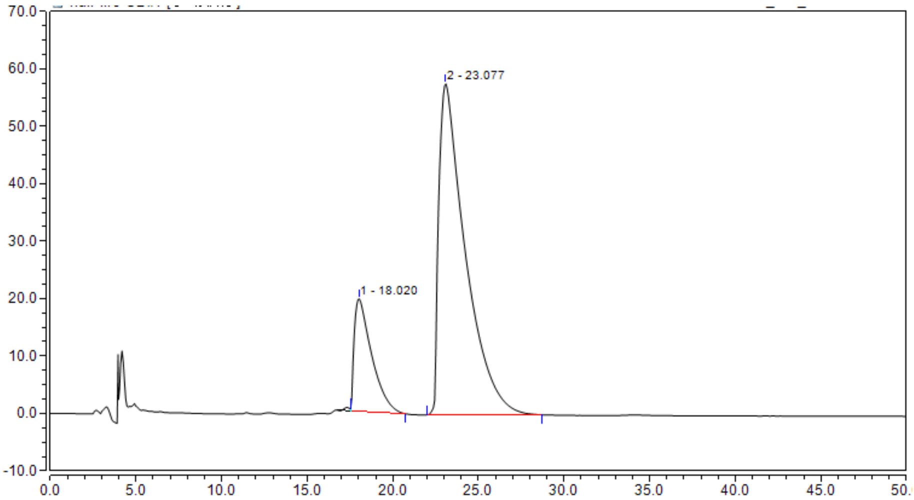

Ret. Time (min)	Area (mAU*min)	Height (mAU)	Area %	Height %
18.020	23.196	19.631	18.32	25.39
23.077	103.452	57.684	81.68	74.61
	**126.648**	**77.315**	**100.00**	**100.00**

Supplementary Fig. 172. HPLC spectrum of chiral 2q.

2r, 9-bromo-4-(2-ethoxynaphthalen-1-yl)-2*H*-quinolizin-2-one

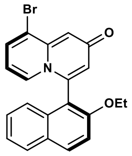

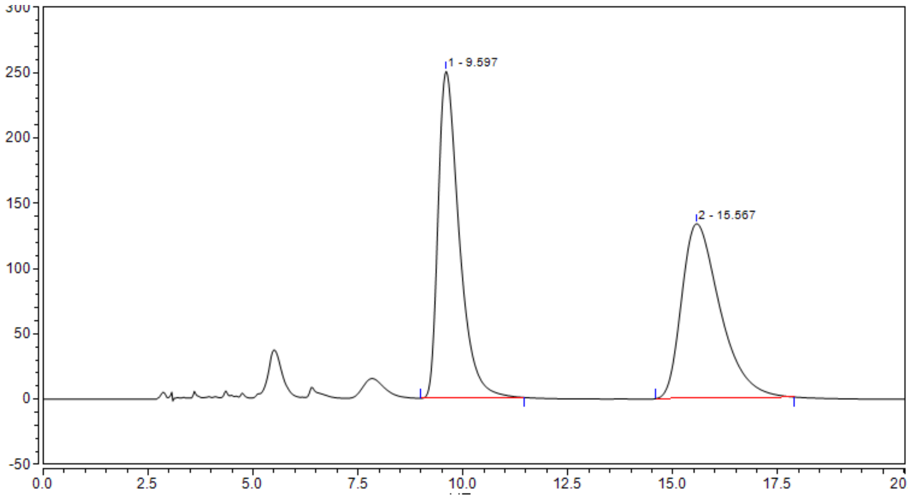

Ret. Time (min)	Area (mAU*min)	Height (mAU)	Area %	Height %
9.597	145.641	250.255	50.67	65.20
15.567	141.791	133.587	49.33	34.80
	**287.432**	**383.843**	**100.00**	**100.00**

Supplementary Fig. 173. HPLC spectrum of racemic 2r.

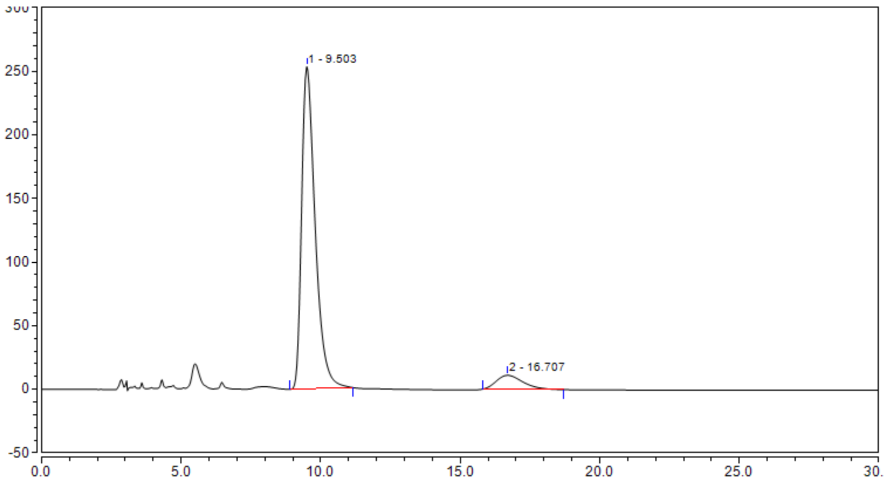

Ret. Time (min)	Area (mAU*min)	Height (mAU)	Area %	Height %
9.503	142.233	253.223	92.07	95.83
16.707	12.245	11.024	7.93	4.17
	**154.478**	**264.247**	**100.00**	**100.00**

Supplementary Fig. 174. HPLC spectrum of chiral 2r.

2s, 8-bromo-4-(2-ethoxynaphthalen-1-yl)-2*H*-quinolizin-2-one

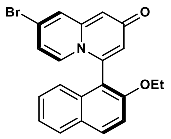

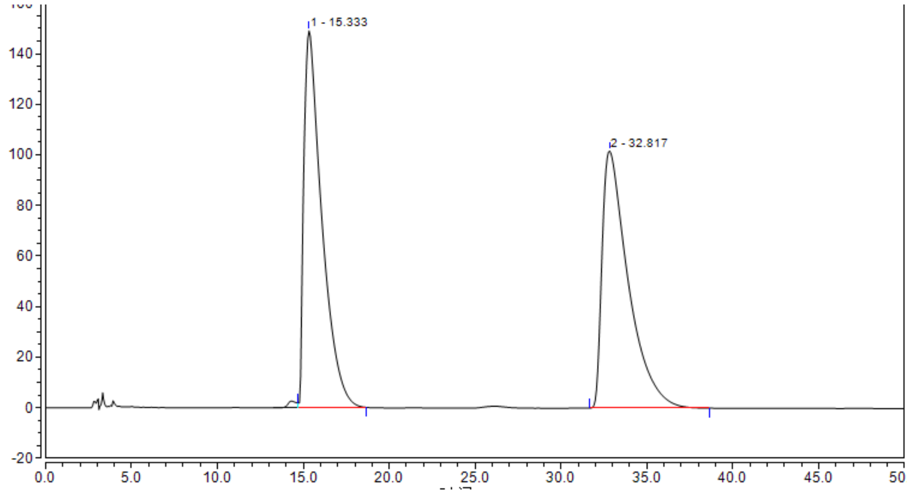

Ret. Time (min)	Area (mAU*min)	Height (mAU)	Area %	Height %
15.333	180.769	149.079	50.21	59.44
32.817	179.221	101.727	49.79	40.56
	359.990	250.805	100.00	100.00

Supplementary Fig. 175. HPLC spectrum of racemic 2s.

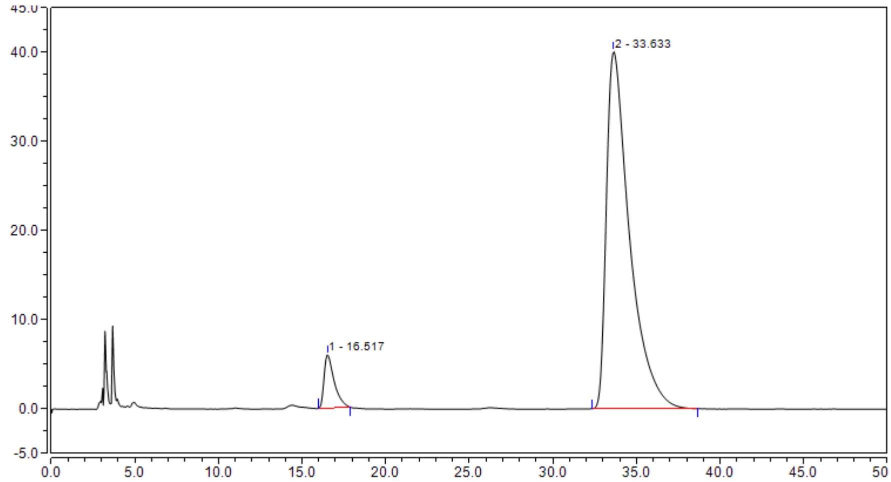

Ret. Time (min)	Area (mAU*min)	Height (mAU)	Area %	Height %
16.517	4.213	5.999	6.16	13.03
33.633	64.233	40.059	93.84	86.97
	68.447	46.059	100.00	100.00

Supplementary Fig. 176. HPLC spectrum of chiral 2s.

2t, 8-chloro-4-(2-ethoxynaphthalen-1-yl)-2*H*-quinolizin-2-one

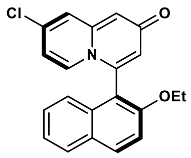

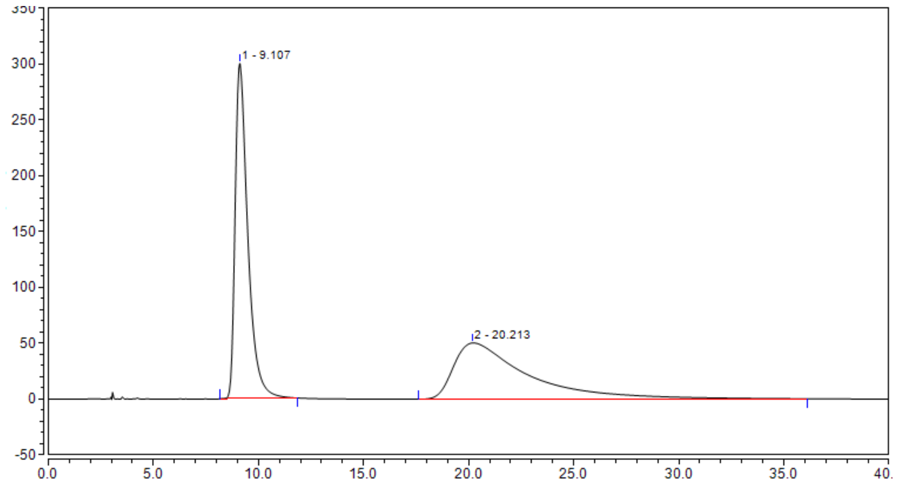

Ret. Time (min)	Area (mAU*min)	Height (mAU)	Area %	Height %
9.107	209.491	300.214	50.65	85.63
20.213	204.145	50.379	49.35	14.37
	**413.637**	**350.594**	**100.00**	**100.00**

Supplementary Fig. 177. HPLC spectrum of racemic 2t.

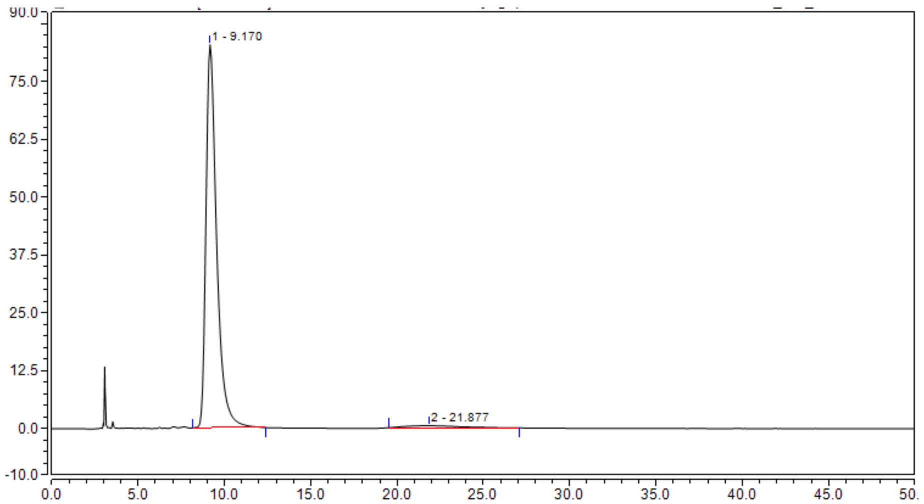

Ret. Time (min)	Area (mAU*min)	Height (mAU)	Area %	Height %
9.170	59.532	82.800	97.32	99.46
21.877	1.639	0.452	2.68	0.54
	**61.172**	**83.253**	**100.00**	**100.00**

Supplementary Fig. 178. HPLC spectrum of chiral 2t.

2u, 7-bromo-4-(2-ethoxynaphthalen-1-yl)-2*H*-quinolizin-2-one

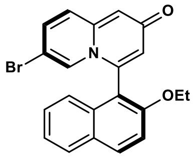

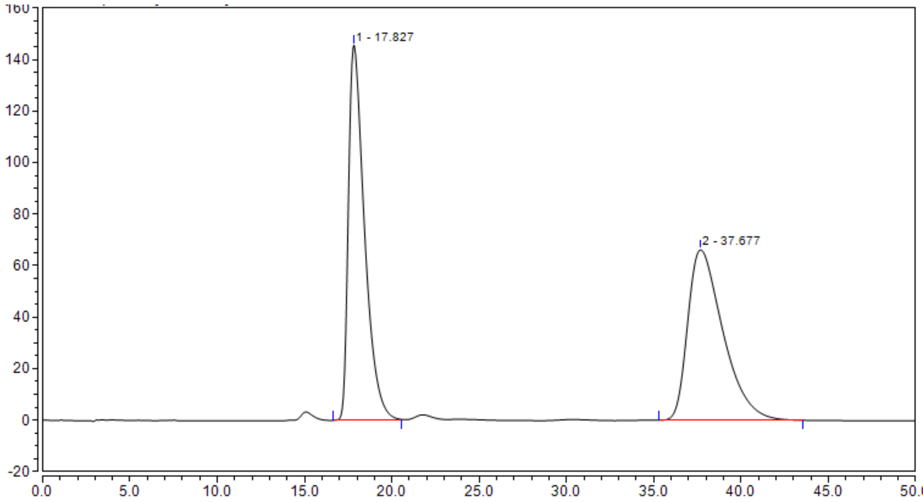

Ret. Time (min)	Area (mAU*min)	Height (mAU)	Area %	Height %
17.827	154.963	145.585	50.01	68.76
37.677	154.889	66.157	49.99	31.24
	**309.852**	**211.742**	**100.00**	**100.00**

Supplementary Fig. 179. HPLC spectrum of racemic 2u.

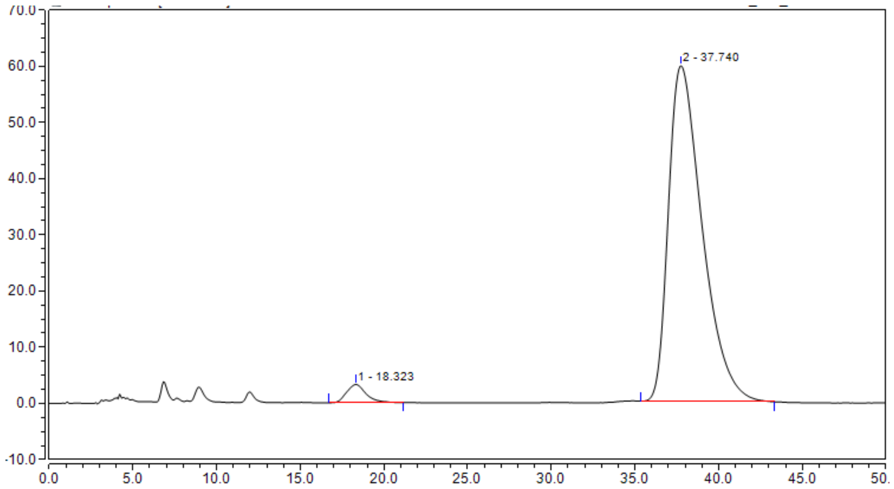

Ret. Time (min)	Area (mAU*min)	Height (mAU)	Area %	Height %
18.323	4.387	3.248	3.05	5.16
37.740	139.504	59.748	96.95	94.84
	**143.891**	**62.997**	**100.00**	**100.00**

Supplementary Fig. 180. HPLC spectrum of chiral 2u.

2v, 4-(2-ethoxynaphthalen-1-yl)-7-methyl-2*H*-quinolizin-2-one

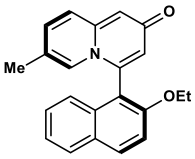

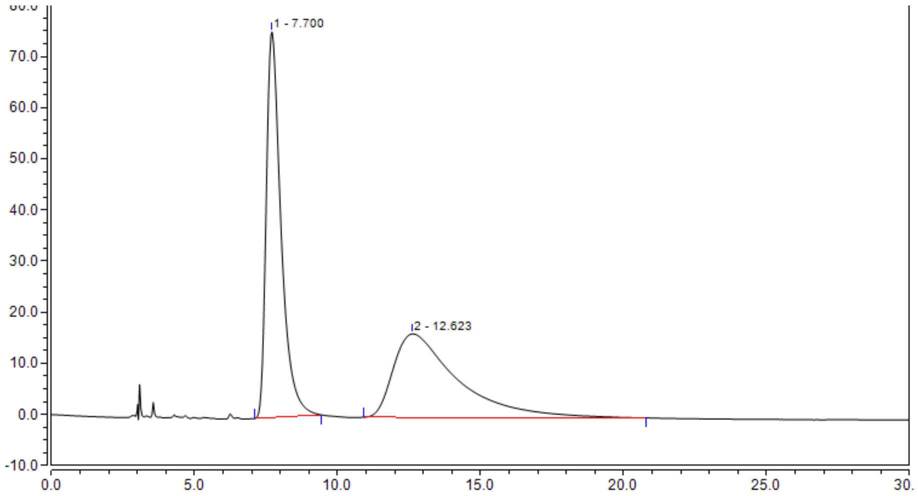

Ret. Time (min)	Area (mAU*min)	Height (mAU)	Area %	Height %
7.700	45.202	75.400	51.63	82.20
12.623	42.343	16.331	48.37	17.80
	**87.545**	**91.731**	**100.00**	**100.00**

Supplementary Fig. 181. HPLC spectrum of racemic 2v.

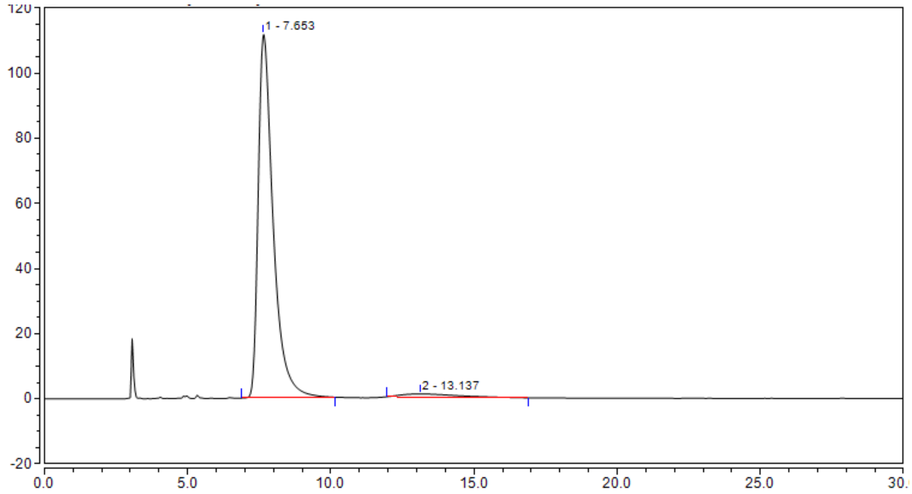

Ret. Time (min)	Area (mAU*min)	Height (mAU)	Area %	Height %
7.653	67.655	111.631	96.93	99.15
13.137	2.143	0.955	3.07	0.85
	**69.797**	**112.586**	**100.00**	**100.00**

Supplementary Fig. 182. HPLC spectrum of chiral 2v.

2w, 6-(2-ethoxynaphthalen-1-yl)-8*H*-pyrido[1,2-*a*]pyrazin-8-one

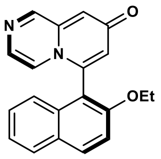

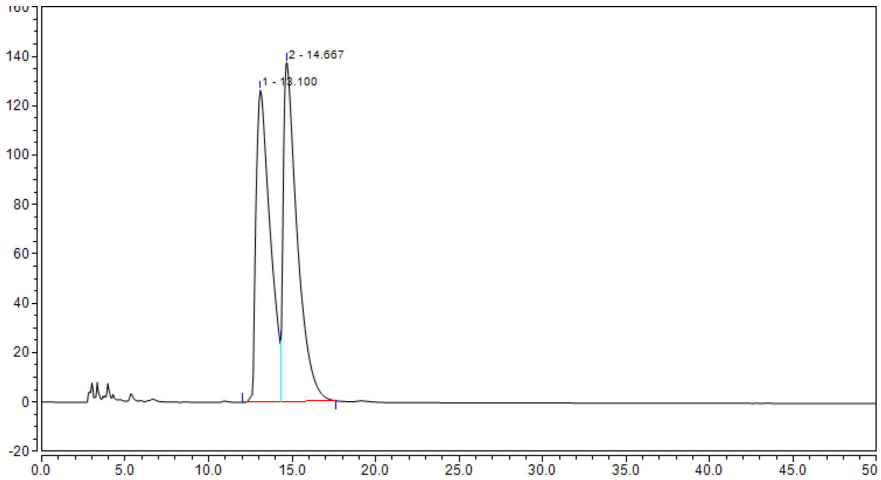

Ret. Time (min)	Area (mAU*min)	Height (mAU)	Area %	Height %
13.100	123.929	126.442	48.21	47.93
14.667	133.146	137.361	51.79	52.07
	**257.076**	**263.802**	**100.00**	**100.00**

Supplementary Fig. 183. HPLC spectrum of racemic 2w.

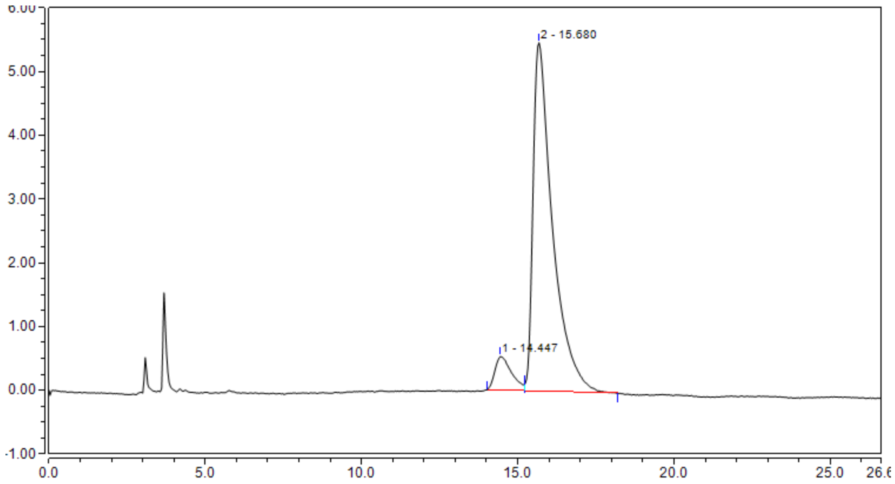

Ret. Time (min)	Area (mAU*min)	Height (mAU)	Area %	Height %
14.447	0.324	0.529	7.58	8.83
15.680	3.951	5.467	92.42	91.17
	**4.275**	**5.996**	**100.00**	**100.00**

Supplementary Fig. 184. HPLC spectrum of chiral 2w.

3, di-*tert*-butyl 1-(4-(2-ethoxynaphthalen-1-yl)-2-oxo-2*H*-quinolizin-1-yl)-hydrazine-1,2-dicarboxylate

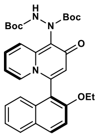

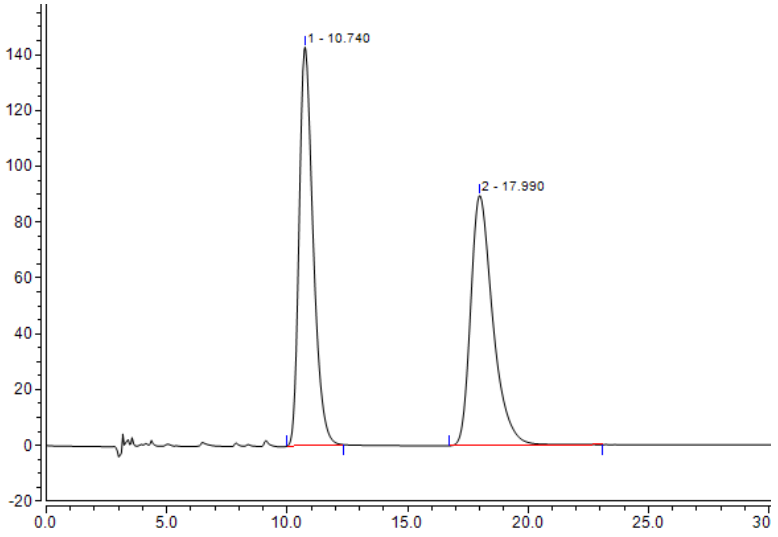

Ret. Time (min)	Area (mAU*min)	Height (mAU)	Area %	Height %
10.740	96.163	142.799	50.09	61.44
17.990	95.817	89.615	49.91	38.56
	**191.980**	**232.414**	**100.00**	**100.00**

Supplementary Fig. 185. HPLC spectrum of racemic 3.

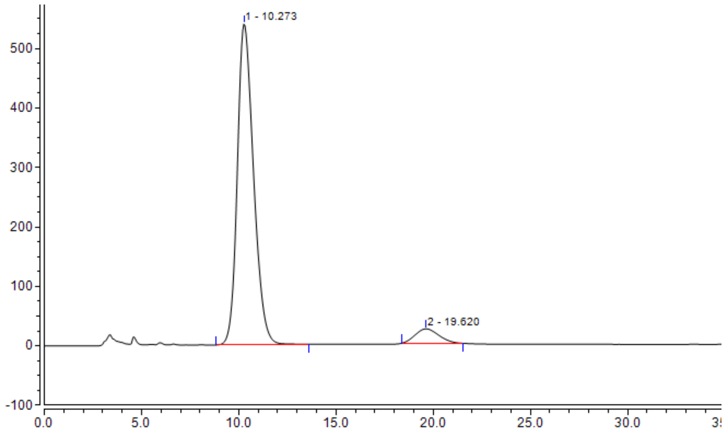

Ret. Time (min)	Area (mAU*min)	Height (mAU)	Area %	Height %
10.273	521.843	539.849	93.89	95.71
19.620	33.973	24.203	6.11	4.29
	**555.816**	**564.052**	**100.00**	**100.00**

Supplementary Fig. 186. HPLC spectrum of chiral 3.

4, 4-(2-hydroxynaphthalen-1-yl)-2*H*-quinolizin-2-one

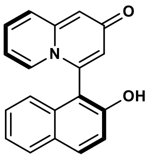

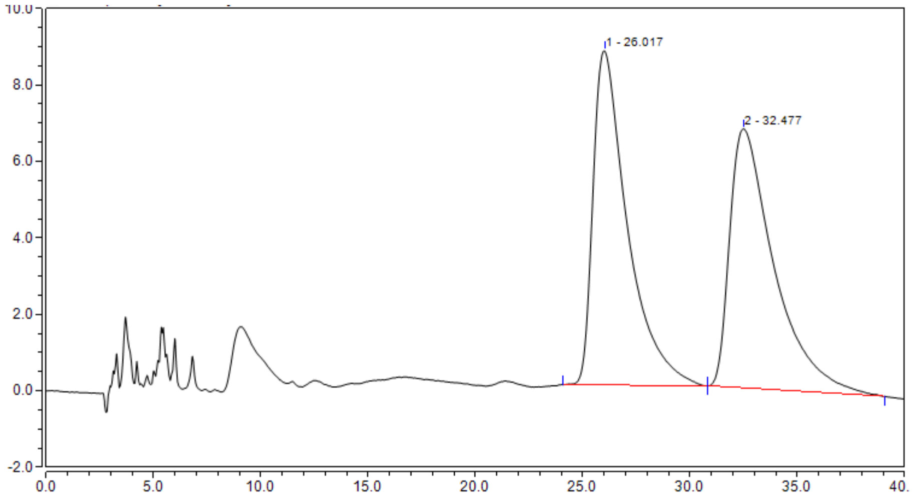

Ret. Time (min)	Area (mAU*min)	Height (mAU)	Area %	Height %
26.017	16.249	8.753	50.07	56.36
32.477	16.203	6.778	49.93	43.64
	**32.452**	**15.531**	**100.00**	**100.00**

Supplementary Fig. 187. HPLC spectrum of racemic 4.

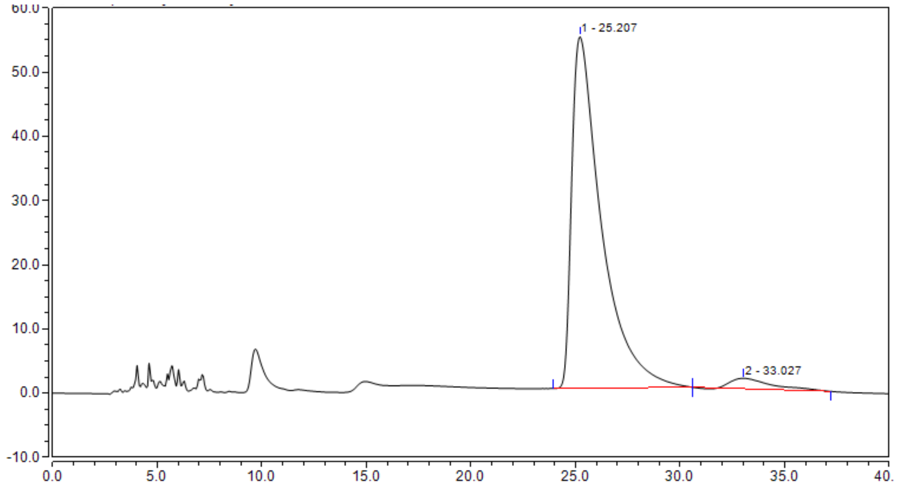

Ret. Time (min)	Area (mAU*min)	Height (mAU)	Area %	Height %
25.207	90.276	54.790	96.06	97.11
33.027	3.703	1.632	3.94	2.89
	**93.979**	**56.422**	**100.00**	**100.00**

Supplementary Fig. 188. HPLC spectrum of chiral 4.

5, 4-(2-((diphenylphosphanyl)oxy)naphthalen-1-yl)-2*H*-quinolizin-2-one

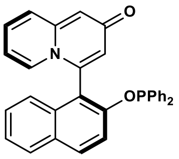

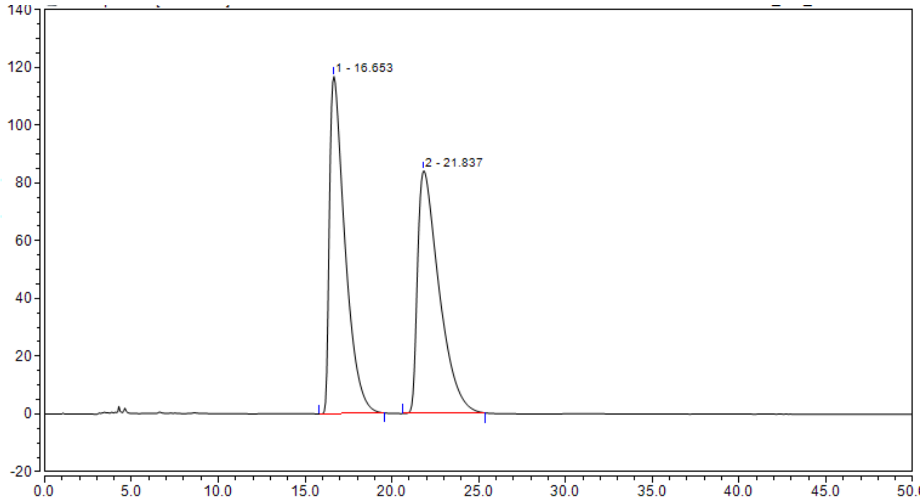

Ret. Time (min)	Area (mAU*min)	Height (mAU)	Area %	Height %
16.653	121.084	116.870	50.10	58.17
21.837	120.599	84.025	49.90	41.83
	**241.684**	**200.895**	**100.00**	**100.00**

Supplementary Fig. 189. HPLC spectrum of racemic 5.

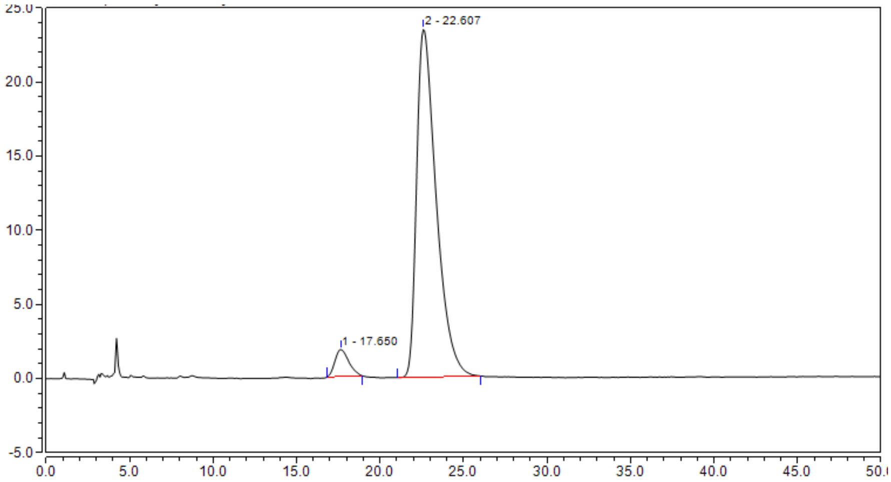

Ret. Time (min)	Area (mAU*min)	Height (mAU)	Area %	Height %
17.650	1.762	1.832	5.23	7.24
22.607	31.934	23.490	94.77	92.76
	**33.696**	**25.323**	**100.00**	**100.00**

Supplementary Fig. 190. HPLC spectrum of chiral 5.

6,1-(2-oxo-2*H*-quinolizin-4-yl)naphthalen-2-yl trifluoromethanesulfonate

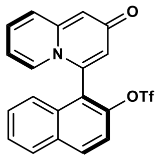

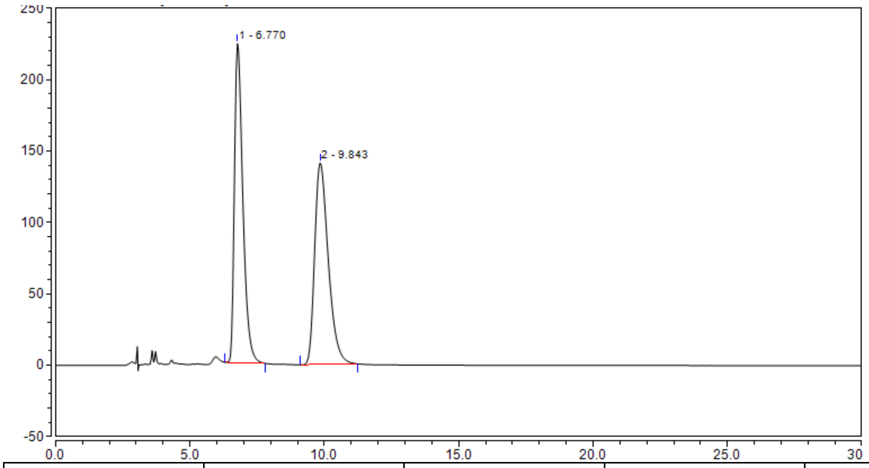

Ret. Time (min)	Area (mAU*min)	Height (mAU)	Area %	Height %
6.770<br>9.843	81.415<br>82.699	223.591<br>141.292	49.61<br>50.39	61.28<br>38.72
	**164.113**	**364.883**	**100.00**	**100.00**

Supplementary Fig. 191. HPLC spectrum of racemic 6.

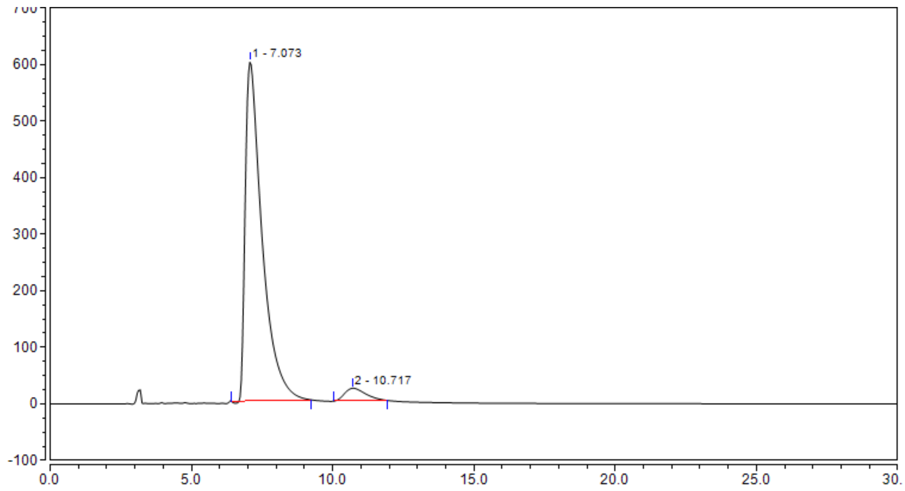

Ret. Time (min)	Area (mAU*min)	Height (mAU)	Area %	Height %
7.073<br>10.717	413.948<br>19.351	600.035<br>22.392	95.53<br>4.47	96.40<br>3.60
	**433.299**	**622.427**	**100.00**	**100.00**

Supplementary Fig. 192. HPLC spectrum of chiral 6.

7, 4-(2-(benzylamino)naphthalen-1-yl)-2*H*-quinolizin-2-one

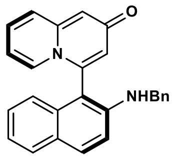

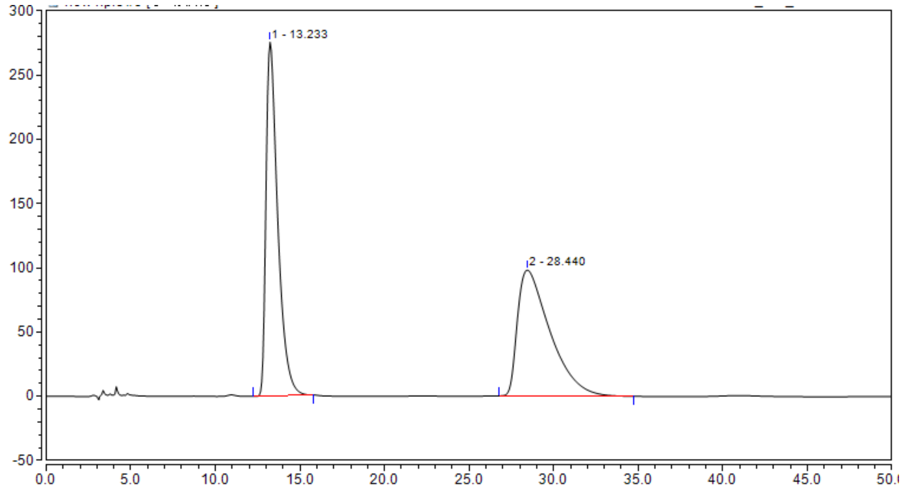

Ret. Time (min)	Area (mAU*min)	Height (mAU)	Area %	Height %
13.233	220.618	275.523	50.21	73.76
28.440	218.735	97.997	49.79	26.24
	**439.354**	**373.520**	**100.00**	**100.00**

Supplementary Fig. 193. HPLC spectrum of racemic 7.

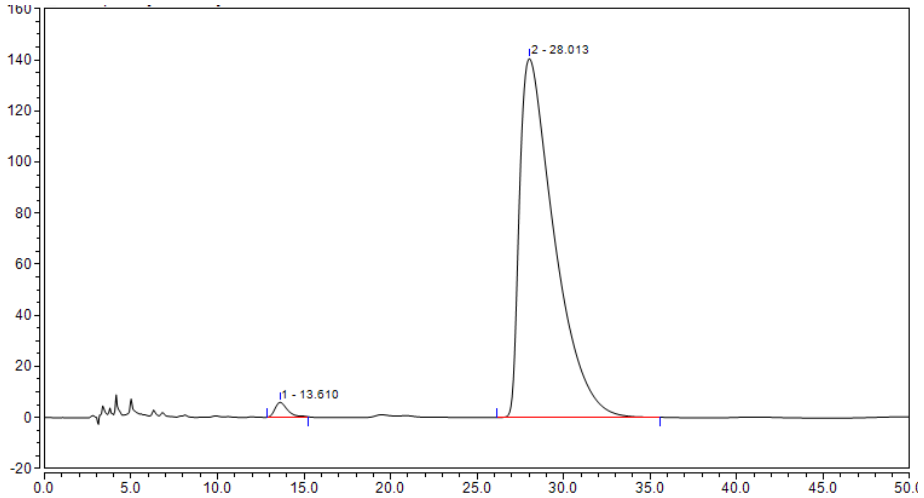

Ret. Time (min)	Area (mAU*min)	Height (mAU)	Area %	Height %
13.610	4.804	5.781	1.45	3.95
28.013	325.536	140.477	98.55	96.05
	**330.341**	**146.257**	**100.00**	**100.00**

Supplementary Fig. 194. HPLC spectrum of chiral 7.

8, 4-(2-aminonaphthalen-1-yl)-6,7,8,9-tetrahydro-2*H*-quinolizin-2-one

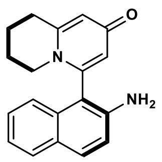

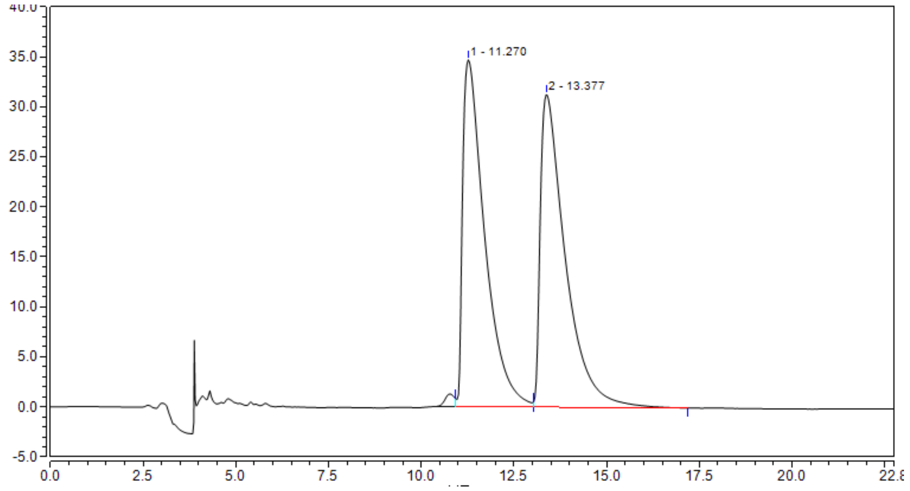

Ret. Time (min)	Area (mAU*min)	Height (mAU)	Area %	Height %
11.270	23.881	34.686	49.44	52.59
13.377	24.420	31.269	50.56	47.41
	**48.302**	**65.955**	**100.00**	**100.00**

Supplementary Fig. 195. HPLC spectrum of racemic 8.

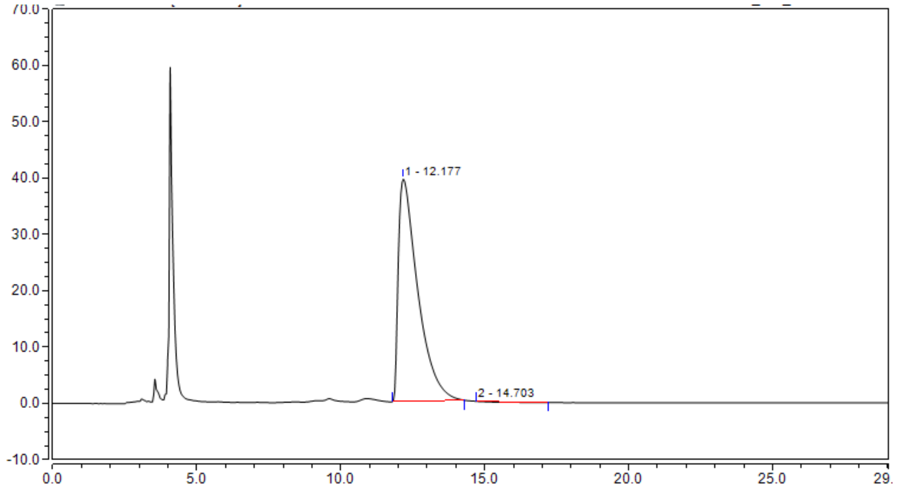

Ret. Time (min)	Area (mAU*min)	Height (mAU)	Area %	Height %
12.177	30.930	39.505	99.63	100.00
14.703	0.114	0.000	0.37	0.00
	**31.044**	**39.505**	**100.00**	**100.00**

Supplementary Fig. 196. HPLC spectrum of chiral 8.

9, 4-(2-aminonaphthalen-1-yl)-2*H*-quinolizin-2-one

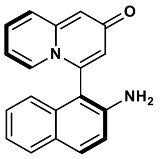

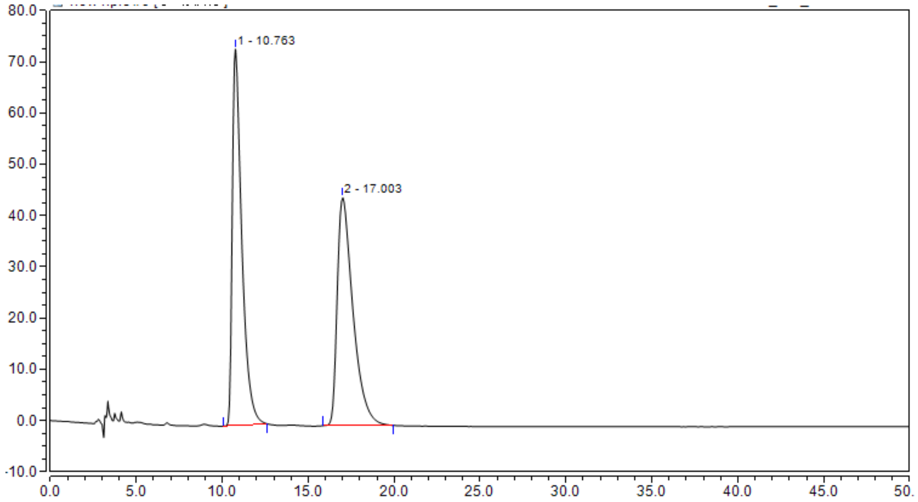

Ret. Time (min)	Area (mAU*min)	Height (mAU)	Area %	Height %
10.763	47.018	73.435	49.94	62.28
17.003	47.122	44.473	50.06	37.72
	**94.140**	**117.908**	**100.00**	**100.00**

Supplementary Fig. 197. HPLC spectrum of racemic 9.

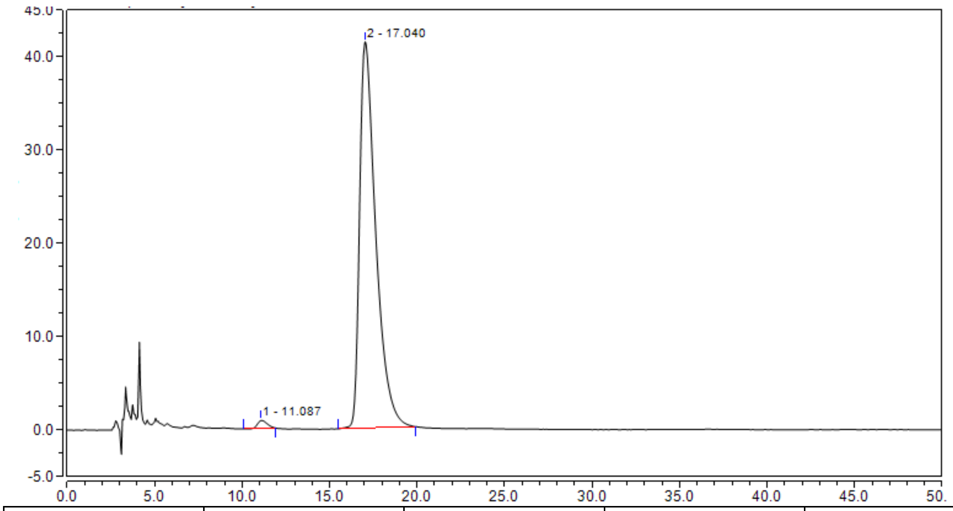

Ret. Time (min)	Area (mAU*min)	Height (mAU)	Area %	Height %
11.087	0.539	0.858	1.20	2.03
17.040	44.358	41.398	98.80	97.97
	**44.897**	**42.257**	**100.00**	**100.00**

Supplementary Fig. 198. HPLC spectrum of chiral 9.

10, 1-(3,5-bis(trifluoromethyl)phenyl)-3-(1-(2-oxo-6,7,8,9-tetrahydro-2*H*-quinolizin-4-yl)naphthalen-2-yl)thiourea

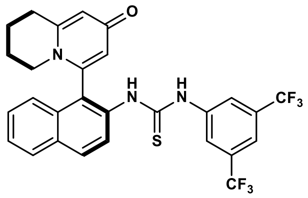

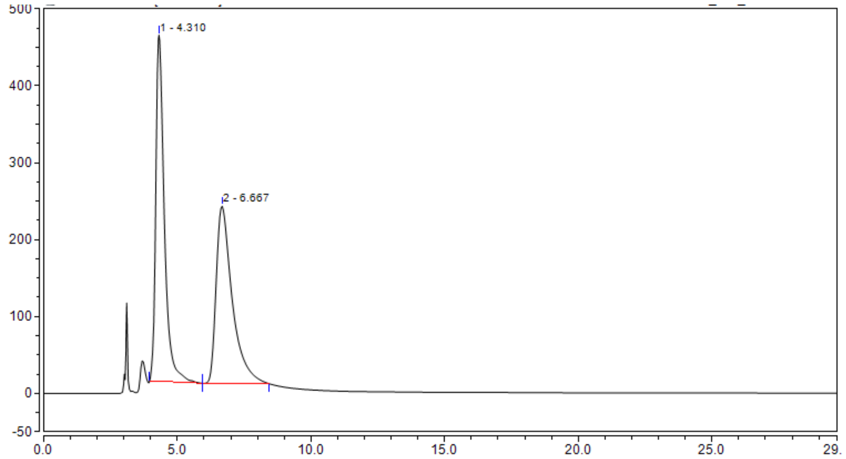

Ret. Time (min)	Area (mAU*min)	Height (mAU)	Area %	Height %
4.310	166.963	450.204	50.93	66.13
6.667	160.865	230.615	49.07	33.87
	**327.828**	**680.819**	**100.00**	**100.00**

Supplementary Fig. 199. HPLC spectrum of racemic 10.

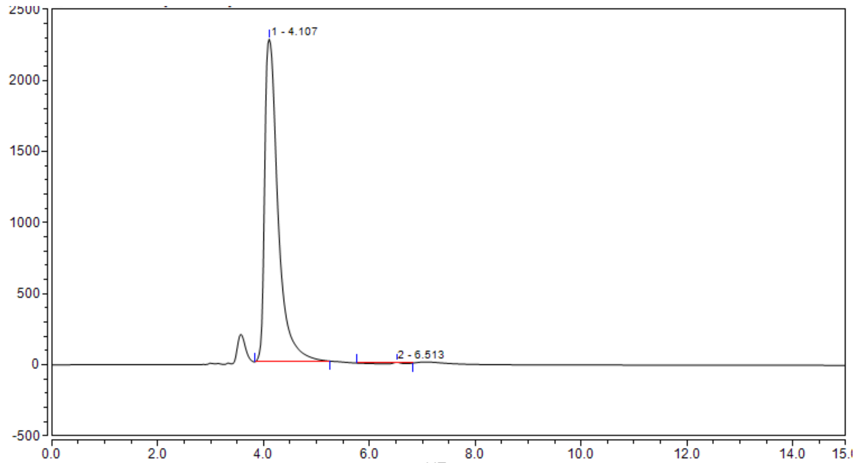

Ret. Time (min)	Area (mAU*min)	Height (mAU)	Area %	Height %
4.107	663.051	2269.579	99.61	99.75
6.513	2.575	5.714	0.39	0.25
	**665.626**	**2275.293**	**100.00**	**100.00**

Supplementary Fig. 200. HPLC spectrum of chiral 10.

11, 1-(3,5-bis(trifluoromethyl)phenyl)-3-(1-(2-oxo-2**H**-quinolizin-4-yl) naphthalen-2-yl)thiourea

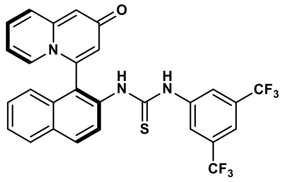

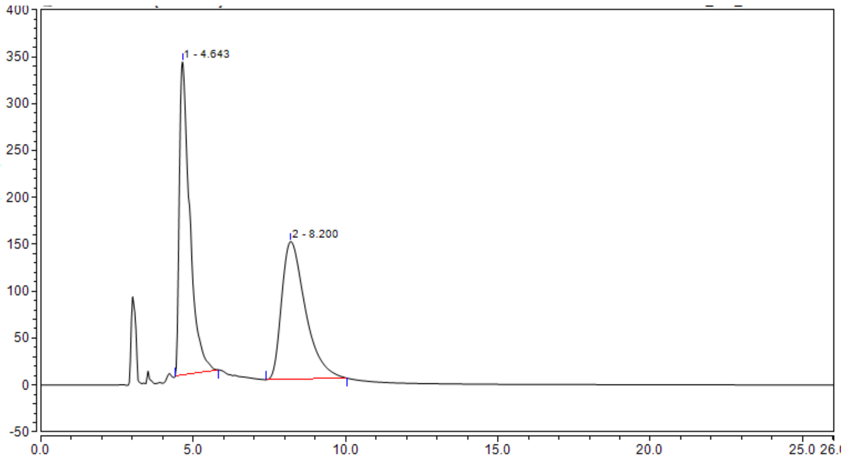

Ret. Time (min)	Area (mAU*min)	Height (mAU)	Area %	Height %
4.643	132.931	333.893	49.98	69.45
8.200	133.023	146.847	50.02	30.55
	**265.953**	**480.739**	**100.00**	**100.00**

Supplementary Fig. 201. HPLC spectrum of racemic 11.

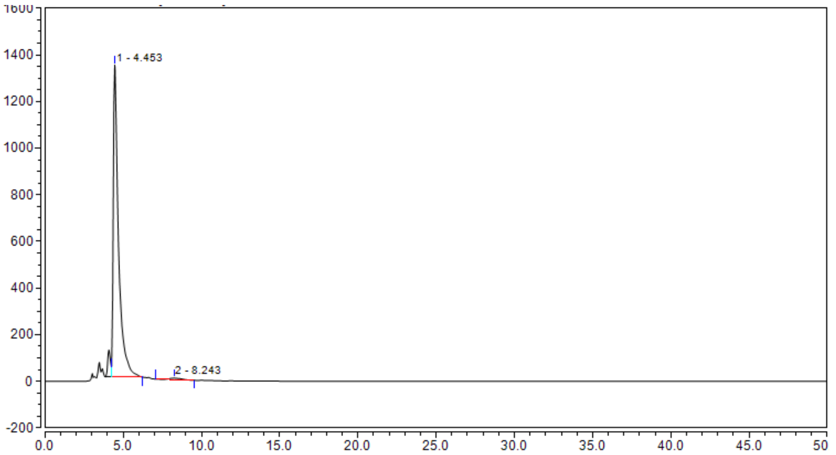

Ret. Time (min)	Area (mAU*min)	Height (mAU)	Area %	Height %
4.453	515.280	1336.940	98.89	99.51
8.243	5.809	6.546	1.11	0.49
	**521.089**	**1343.486**	**100.00**	**100.00**

Supplementary Fig. 202. HPLC spectrum of chiral 11.

(*S*)-3-(*p*-tolylthio)cyclohexan-1-one

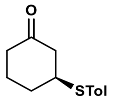

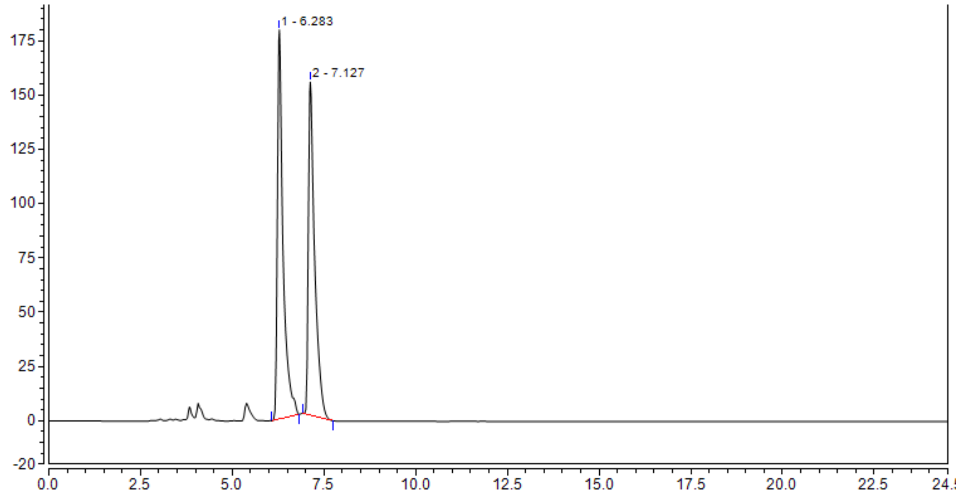

Ret. Time (min)	Area (mAU*min)	Height (mAU)	Area %	Height %
6.283	32.204	179.144	51.39	53.88
7.127	30.467	153.367	48.61	46.12
	**62.671**	**332.511**	**100.00**	**100.00**

Supplementary Fig. 203. HPLC spectrum of racemic 3-(p-tolylthio)cyclohexan-1-one

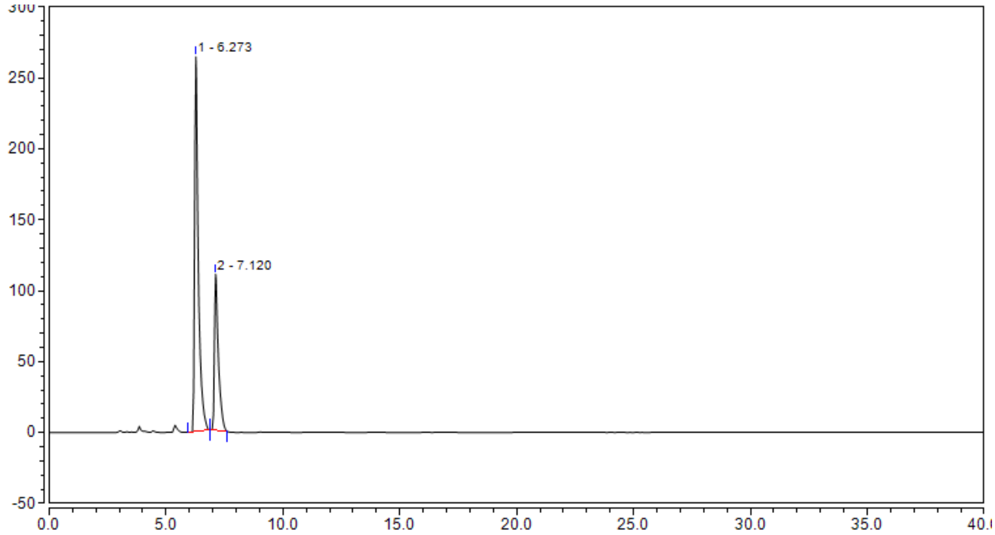

Ret. Time (min)	Area (mAU*min)	Height (mAU)	Area %	Height %
6.273	48.199	264.218	69.38	70.58
7.120	21.276	110.129	30.62	29.42
	**69.475**	**374.347**	**100.00**	**100.00**

Supplementary Fig. 204. HPLC spectrum of chiral 3-(p-tolylthio)cyclohexan-1-one

Supplementary References

1. Min, X.-L., Sun, C., & He, Y. Synthesis of 1-amino-2*H*-quinolizin-2-one scaffolds by tandem silver catalysis. *Org. Lett.* **21**, 724-728 (2019).

2. Ageshina, A. A., Chesnokov, G. A., Topchiy, M. A., Alabugin, I. V., Nechaev, M. S., & Asachenke, A. F. Making endo-cyclizations favorable again: A conceptually new synthetic approach to benzotriazoles *via* azide group directed lithiation/cyclization of 2-azidoaryl bromides. *Org. Biomol. Chem.* **17**, 4523-4534 (2019).

3. Li S.-L., Yang C., Wu Q., Zheng H.-L., Li X & Cheng J.-P. Atroposelective catalytic asymmetric allylic alkylation reaction for axially chiral anilides with achiral Morita–Baylis–Hillman carbonates. *J. Am. Chem. Soc.* **140,** 12836–12843(2018).

4. Li, Y., Manickam, G., Ghoshal, A., & Subramaniam, P. More efficient palladium catalyst for hydrogenolysis of benzyl groups. *Synth. Commun.* **36**, 925-928 (2006).

5. Rana, N. K. Selvakumar, S. & Singh, V. K. Highly enantioselective organocatalytic dulfa-Michael sddition to *α, β*-unsaturated ketones. *J. Org. Chem.* **75**, 2089-2091 (2010).

6. Adams, R., & Yuan, H. C. The stereochemistry of diphenyls and analogous compounds. *Chem. Rev.* **12**, 261-338 (1933).

7. Winstein, S., & Holness, N. J. Neighboring carbon and hydrogen. XIX. *t*-Butylcyclohexyl derivatives. Quantitative conformational analysis. *J. Am. Chem. Soc.* **77**, 5562-5578 (1955).

8. Charton, M. Steric effects. II. Base-catalyzed ester hydrolysis. *J. Am. Chem. Soc.* **97**, 3691-3693 (1975).

9. Charton, M. Steric effects. 7. Additional V constants. *J. Org. Chem.* **41**, 2217-2220 (1976).

10. Bott, G., Field, L. D., & Sternhell, S. Steric effects. A study of a rationally designed system. *J. Am. Chem. Soc*. **102**, 5618-5626 (1980).
